# Establishing the phylogeny of Prochlorococcus with a new alignment‐free method

**DOI:** 10.1002/ece3.3535

**Published:** 2017-11-15

**Authors:** Xin Zhao, Kun Tian, Rong L. He, Stephen S.‐T. Yau

**Affiliations:** ^1^ Department of Mathematical Sciences Tsinghua University Beijing China; ^2^ Department of Biological Sciences Chicago State University Chicago IL USA

**Keywords:** natural vector, phylogenetic analysis, *Prochlorococcus*, sequence analysis

## Abstract

*Prochlorococcus* marinus, one of the most abundant marine cyanobacteria in the global ocean, is classified into low‐light (LL) and high‐light (HL) adapted ecotypes. These two adapted ecotypes differ in their ecophysiological characteristics, especially whether adapted for growth at high‐light or low‐light intensities. However, some evolutionary relationships of *Prochlorococcus* phylogeny remain to be resolved, such as whether the strains SS120 and MIT9211 form a monophyletic group. We use the Natural Vector (NV) method to represent the sequence in order to identify the phylogeny of the *Prochlorococcus*. The natural vector method is alignment free without any model assumptions. This study added the covariances of amino acids in protein sequence to the natural vector method. Based on these new natural vectors, we can compute the Hausdorff distance between the two clades which represents the dissimilarity. This method enables us to systematically analyze both the dataset of ribosomal proteomes and the dataset of 16s‐23s rRNA sequences in order to reconstruct the phylogeny of *Prochlorococcus*. Furthermore, we apply classification to inspect the relationship of SS120 and MIT9211. From the reconstructed phylogenetic trees and classification results, we may conclude that the SS120 does not cluster with MIT9211. This study demonstrates a new method for performing phylogenetic analysis. The results confirm that these two strains do not form a monophyletic clade in the phylogeny of *Prochlorococcus*.

## 1 INTRODUCTION

The oceans play an important role in global nutrient cycling and climate regulation. The genus *Prochlorococcus* is a marine cyanobacteria that dominates most tropical and temperate oceans (Kettler et al., 2007; Moore, Rocap, & Chisholm, 1998; Murata et al., 2017; Partensky, Hess, & Vaulot, 1999). As the smallest (<1 μm diameter) and most abundant (3 × 10^27^ cells) photosynthetic organism on the planet, *Prochlorococcus* plays a key role in the microbial world (Biller et al., 2014; Murphy et al., 2017). *Prochlorococcus* group consists of two major ecotypes: high‐light (HL)‐adapted and low‐light (LL)‐adapted. These two ecotypes are genetically and physiologically distinct (Aharonovich & Sher, 2016; Biller, Coe, & Chisholm, 2016; Gómez‐Baena, Rangel, López‐Lozano, García‐ Fernández, & Diez, 2009; Kettler et al., 2007). High‐light‐adapted ecotypes occupy the upper, well illuminated but nutrient‐poor 100‐m layer of the water column, whereas low‐light‐adapted ecotypes preferentially thrive at the bottom of the euphotic zone (80–200 m) at dimmer light but in a nutrient‐rich environment (Avrani & Lindell, 2015; Casey, Mardinoglu, Nielsen, & Karl, 2016; Dufresne et al., 2003). *Prochlorococcus* have the smallest genomes of any known free‐living photosynthetic cell, ranging from 1.6 to 2.7 Mbp. Despite the ecotype differentiation, this group has at least 97% 16S rRNA similarity (Biller et al., 2014). Past phylogenetic studies of *Prochlorococcus* were mainly based on nucleotide sequence data (Kettler et al., 2007; Luo, Shi, Arndt, Tang, & Friedman, 2008; Moore et al., 1998). However, some evolutionary relationships of *Prochlorococcus* phylogeny were still unresolved. For example, whether the low‐light‐adapted *Prochlorococcus* marinus type strains SS120 (also known as CCMP1375) and MIT9211 form a monophyletic group remained unclear (Kettler et al., 2007; Luo et al., 2008). Figures in Kettler's study (Kettler et al., 2007) show two alternative phylogenetic relationships of *Prochlorococcus*. In one figure, SS120 does not cluster with MIT9211 and the other figures show SS120 and MIT9211 forming a separate clade.

The long computation time of alignment based method makes it difficult to do phylogeny analyses on species containing large number of sequences, such as virus and bacteria. The alignment‐free method is useful as it can handle large number of sequences easily and quickly. Tens of thousands of whole genomes or proteomes could be compared simultaneously in a short time. In previous researches, the alignment‐free natural vector method has been widely used in studying the evolutionary of virus and bacteria (Deng, Yu, Liang, He, & Yau, 2011; Povolotskaya & Kondrashov, 2010; Tian et al., 2015; Yau, Yu, & He, 2008; Yu, Cheng, He, & Yau, 2011; Yu, He, & Yau, 2013c; Yu et al., 2013a,b; Zhao, Wan, He, & Yau, 2016). This method is based on the normalized distribution of amino acids in protein sequence without any model assumption. The correspondence between protein sequences and their 60‐dimensional natural vectors is one‐to‐one (Deng et al., 2011). In this study, we develop a new natural vector method which adds the covariances of amino acids to the existing natural vector method (Deng et al., 2011) and use it infer the phylogeny of Prochlorococcus with increased accuracy. We aim at investigating the phylogeny and resolving the phylogenetic relationship of SS120 and MIT9211. We apply the Hausdorff distance in the protein space to measure the dissimilarity distance between pairs of strains of *Prochlorococcus*. In order to illustrate the results clearly, we add the classification of the 12 *Prochlorococcus* strains to analyze the similarity of SS120 and MIT9211.

## 2 MATERIALS AND METHODS

### 2.1 Datasets

In this study, we chose both 16s‐23s rRNA sequences and a full set of ribosomal protein sequences of *Prochlorococcus* as the datasets. Both datasets were downloaded from NCBI database. The ribosomal protein dataset which contained 12 *Prochlorococcus* strains is listed in Table 1. Datasets used in this study could be obtained from S1 Dataset and S2 Dataset. We did not trim or align the rRNA and protein sequences, as we consider this type operation to be artificial. This operation may lead to the result not as real and reliable as using the original dataset.

**Table 1:** The strain names and number of the proteins in the ribosomal protein dataset

Strain names	Light adaptation	No. of ribosomal proteins
MED4	HL	118
MIT9515	HL	114
MIT9312	HL	114
AS9601	HL	106
MIT9301	HL	107
MIT9215	HL	109
SS120	LL	188
MIT9211	LL	105
NATL2A	LL	113
NATL1A	LL	106
MIT9303	LL	114
MIT9313	LL	129

### 2.2 Natural vector

We use natural vector to represent the features of proteins in the dataset. The natural vector method is alignment free, which does not depend on any assumptions. The natural vector method for protein is defined as follows (Deng et al., 2011; Yu et al., 2013a).

Let $S=\left(s_{1},s_{2},s_{3},\cdots ,s_{N}\right)$ be a protein sequence of length N, where $s_{i}\in \left\{A,R,N,D,C,E,Q,G,H,I,L,K,M,F,P,S,T,W,Y,V\right\},\;i=1,2,3,\cdots N$.

When k is one of the 20 amino acids, define

$w_{k}\left(\cdot \right):\left\{A,R,N,D,C,E,Q,G,H,I,L,K,M,F,P,S,T,W,Y,V\right\}\to \left\{0,1\right\}$

such that $w_{k}\left(s_{i}\right)=1$ if $s_{i}=k$ and otherwise $w_{k}\left(s_{i}\right)=0$.

1. Let $n_{k}=\sum_{i=1}^{N}w_{k}\left(s_{i}\right)$ denote the occurrence of the number of amino acid k in the protein sequence S.
2. Let $T_{k}=\sum_{i=1}^{N}i\cdot w_{k}\left(s_{i}\right)$ be the total distance for each set of 20 amino acids.
3. We then take $u_{k}=\frac{T_{k}}{n_{k}}$ as the mean position of amino acid k.
4. Finally, we define the normalized central moments as follows:

$D_{j}^{k}=\sum_{i=1}^{N}\frac{{\left(i-\mu_{k}\right)}^{j}w_{k}\left(s_{i}\right)}{n_{k}^{\;j-1}N^{j-1}},\;j=1,2,3,\cdots ,n_{k},$

where *k* represents the twenty amino acids.

For *j* = 1, note that

$$\begin{array}{cc} D_{1}^{k} & =\sum_{i=1}^{N}\left(i-\mu_{k}\right)w_{k}\left(s_{i}\right)=\sum_{i=1}^{N}i\cdot w_{k}\left(s_{i}\right)-\mu_{k}\sum_{i=1}^{N}w_{k}\left(s_{i}\right)\\ & =T_{k}-\mu_{k}n_{k}=0. \end{array}$$

Therefore, the first order moments can be ignored. The natural vector N(S) of a protein sequences S is given as follows,

$\left(n_{A},n_{R},\cdots ,n_{V},\mu_{A},\mu_{R},\cdots ,\mu_{V},D_{2}^{A},\cdots D_{n_{A}}^{A},D_{2}^{R},\cdots D_{n_{R}}^{R},\cdots ,D_{2}^{V},\cdots D_{n_{V}}^{V}\right).$

We can prove mathematically that the correspondence between protein sequences and their natural vectors is one‐to‐one (Deng et al., 2011).

As for natural vector of DNA sequences, we define $s_{i}\in \left\{A,C,G,T\right\},\;i=1,2,3,\cdots N$. We calculate natural vectors the same way as we calculate that of protein. The natural vector N(S) of a DNA sequence S is given as follows,

$\left(n_{A},n_{C},n_{G},n_{T},\mu_{A},\mu_{C},\mu_{G},\mu_{T},D_{2}^{A},\cdots D_{n_{A}}^{A},D_{2}^{C},\cdots D_{n_{C}}^{C},D_{2}^{G},\cdots D_{n_{G}}^{G},D_{2}^{T},\cdots D_{n_{T}}^{T}\right).$

The 12‐dimensional natural vector with *j* = 2 in $D_{j}^{k}$ is usually used to represent DNA sequences, and the 60‐dimensional natural vector with *j* = 2 represents the proteins. In this study, we introduce the 18‐dimensional natural vector and 250‐dimension natural vector with covariance to make further investigation on DNA and proteins, respectively. The 250‐dimensional natural vector can be explained as the following.

Let $\;A=\left\{a_{1},a_{2},\cdots ,a_{n}\right\}$ and $B=\left\{b_{1},b_{2},\cdots ,b_{m}\right\}$ be two finite point sets in R, where $a_{1}<a_{2}<\cdots <a_{n}$ and $b_{1}<b_{2}<\cdots <b_{m}$. We need to calculate the covariance between A and B.

1. If m=n, then $\mathrm{Cov}\left(A,B\right)=\sum_{i=1}^{m}\left(a_{i}-u_{A}\right)\left(b_{i}-u_{B}\right)/m$, where $u_{A}=\sum_{i=1}^{n}\frac{a_{i}}{n},\;u_{B}=\sum_{i=1}^{m}\frac{b_{i}}{m}$.
2. If m≠n, we can assume that n>m. We then choose m numbers from set A, which satisfy$a_{i_{1}}<a_{i_{2}}<\cdots <a_{i_{m}},\;1\leq i_{1}<i_{2}<\cdots <i_{m}\leq n$, and here are $C_{n}^{m}\;$choices in total. We compute the covariance between the m numbers and set B, then take the average value of these $C_{n}^{m}$ results as final covariance between the point sets A and B written as I. Then the final result is

$I=\frac{1}{mC_{n}^{m}}{\text{BDA}}^{T}-\mu_{A}\mu_{B},$

where $\mu_{A}=\sum_{i=1}^{n}\frac{a_{i}}{n},\;\mu_{B}=\sum_{i=1}^{m}\frac{b_{i}}{m}$, $A^{T}={\left\{a_{1},a_{2},\cdots ,a_{n}\right\}}^{T}$ represents an n×1 column vector, and D is an m×n matrix written as ${\left(D_{ij}\right)}_{m\times n}$,

$\text{if}\;i=1,D_{ij}=\left\{\begin{array}{c} C_{n-j}^{m-1},\;1\leq j\leq n-m+1\;\\ 0,\;n-m+2\leq j\leq n \end{array}\right.$,

$\text{if}\;2\leq i\leq m-1,\;D_{ij}=\left\{\begin{array}{c} 0,\;1\leq j\leq i-1\\ C_{j-1}^{i-1}C_{n-j}^{m-i},i\leq j\leq n-m+i\\ 0,\;n-m+i+1\leq j\leq n \end{array}\right.$,

$\text{if}\;i=m,D_{ij}=\left\{\begin{array}{c} 0,\;1\leq j\leq i-1\;\\ C_{j-1}^{m-1},\;i\leq j\leq n \end{array}\right.$.

For a sequence S of length N, we want to compute the covariance between any pair of nucleotides or amino acids X and Y. Assume that position of X appeared in the sequence S is $A=\left\{a_{1},a_{2},\cdots ,a_{n}\right\}$, and the position of Y is $B=\left\{b_{1},b_{2},\cdots ,b_{m}\right\}$. Then the covariance between X and Y is defined as Cov(A, B)/N.

For example, given a DNA sequence ACACACGTGT, we first compute the covariance between nucleotides A and C. The position of A appeared in the sequence is {1,3,5}, and the position of C is {2,4,6}. We could calculate $u_{A}=3$ and $u_{C}=4$. Then the covariance between nucleotides A and C is $\left[\left(1-3\right)\left(2-4\right)/3+\left(3-3\right)\left(4-4\right)/3+\left(5-3\right)\left(6-4\right)/3\right]/10=4/15.$ Secondly, we calculate the covariance between A and G. The position of G is {7,9} and $u_{G}=8$. The covariance between A and G is {[(1−2)(7−8)/2+(3−2)(9−8)/2]+[(1−3)(7−8)/2+(5−3) (9−8)/2]+[(3−4)(7−8)/2+(5−4)(9−8)/2]}/(3×10)=2/15. The covariances between the other pairs of nucleotides could be calculated in the same way.

After we get the covariances between the pairs of nucleotides or amino acids, we add the covariances to the original natural vector of the sequence S. The number of pairs of nucleotides acids is $C_{4}^{2}=6$ and the number of pairs of amino acids is $C_{20}^{2}=190$. Thus, the dimension of the natural vector of DNA is extended from 12 to 18 while the dimension of protein is extended from 60 to 250. We then obtain a new type of natural vector which reflects natural statistic information for sequences.

### 2.3 Hausdorff distance

In mathematics, the Hausdorff distance measures the degree of dissimilarity between two sets.

Let X and Y be two finite point sets of a metric space such as $X=\left\{x_{1},x_{2},\cdots ,x_{n}\right\}$ and $Y=\left\{y_{1},y_{2},\cdots ,y_{m}\right\}$. The Hausdorff distance between X and Y is defined by

$H\left(X,Y\right)=\max_{\;}\left\{\max_{x\in X}\min_{y\in Y}d\left(x,y\right),\max_{y\in Y}\min_{x\in X}d\left(x,y\right)\right\}$

where d(*x*,* y*) means underlying norm between x in X and y in Y (Huttenlocher, Klanderman, & Rucklidge, 1993), such as the Euclidean distance and the Manhattan distance. The Hausdorff distance is a true metric and it satisfies the triangle inequality

$H\left(X,Y\right)\leq H\left(X,Z\right)+H\left(Z,Y\right)$

Here, X, Y, Z represent non‐empty sets, respectively.

The Hausdorff distance is defined as the distance between the point in one set that is the farthest from any point of the other set and vice versa. Presently, the most useful criterion to measure the similarity between two point sets is the Hausdorff distance. This distance can be used to determine the degree of resemblance between two point sets that are superimposed on one another. It can be used to compare any two species for which various DNA or protein sequences are available. As we use a natural vector to represent a DNA or protein sequence and each *Prochlorococcus* strain contains a set of DNA or proteins, then each strain corresponds to a set of natural vectors. Common metrics such as the Euclidean distance and the Manhattan distance are used to measure the distance between two points, while the Hausdorff distance is able to measure the dissimilarity of the two sets of natural vectors.

### 2.4 Classification

We propose a classification method to reveal the phylogenetic relationship on *Prochlorococcus* further. The classification rules are as follows. The ribosomal proteins from 12 strains are used as the feature database forming a set S, which is a union of 12 subsets.

S=S_1∪S_2∪⋯∪S_12.

For the query protein X to be predicted, we are trying to find the similarities between the query protein X and the protein family S_k. These similarities could be measured by the least Euclidean distance between the X and the proteins of the family S_k.

$D\left(X,S\_k\right)=\min_{X\neq X_{k}^{\xi }}\left\{D_{E}\left(X,X_{k}^{\xi }\right)\right\}$

where $X_{k}^{\xi }$ represents the xith protein in the subset S_k and $D_{E}\left(X,X_{k}^{\xi }\right)$ means the Euclidean distance between X and${\;X}_{k}^{\xi }$ .

The shorter distance between the protein X and the family S_k represents more similarities. The classification rule is to find the least distance $D\left(X,S\_k\right)$ which the query protein belongs to.

## 3 RESULTS AND DISCUSSION

### 3.1 Phylogeny of *Prochlorococcus*

As introduced in the materials and methods section, we first calculated the 250‐dimensional natural vectors of the ribosomal proteins of each strain and the pairwise distances between these natural vectors. We then obtained the Hausdorff distances between the 12 *Prochlorococcus* strains. The phylogenetic tree is reconstructed by single‐linkage method (Gower & Ross, 1969) based on the Hausdorff distance.

**Figure 1:**
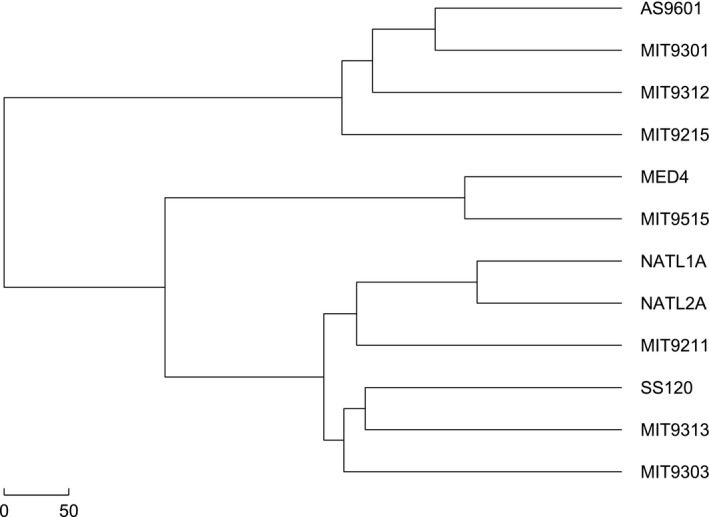
Phylogenetic tree reconstructed by the Euclidean distance and the Hausdorff distance based on the natural vectors of ribosomal proteins

*Prochlorococcus* is classified into low‐light (LL) and high‐light (HL) adapted ecotypes. These two adapted ecotypes differ by their ecophysiological characteristics, including whether adapted for growth at high‐light or low‐light intensities. The 6 HL strains form a cluster while the 6LL strains form another one as shown in Figure 1. In the HL cluster, the MED4 and MIT9515 cluster into one clade, remaining 4 HL strains form another one. Although 6 LL strains form a cluster, the SS120 and MIT9211 do not form a separate clade.

**Figure 2:**
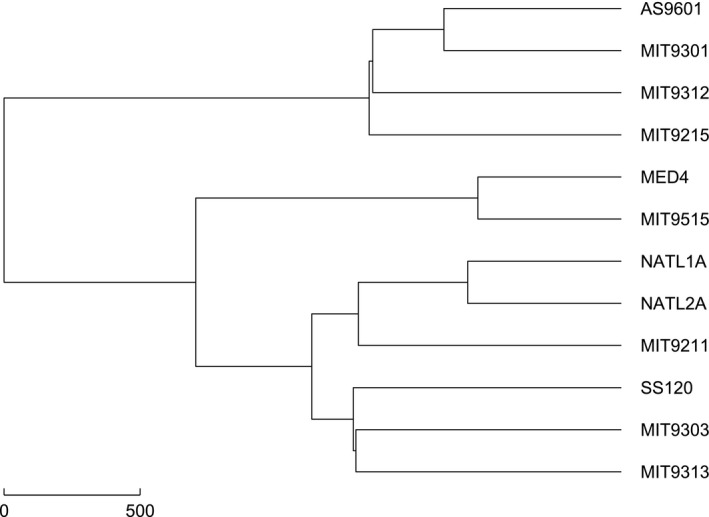
Phylogenetic tree reconstructed by the Manhattan distance and the Hausdorff distance based on the natural vectors of ribosomal proteins

In order to further prove this point, different methods and datasets are used. We applied the Manhattan distance instead of the Euclidean distance to measure the similarity between two natural vectors. From the phylogeny of *Prochlorococcus* reconstructed by the Manhattan distance as shown in Figure 2, we can find out that SS120 and MIT9211 still do not form a separate clade, which is consistent with the previous results in Figure 1.

**Figure 3:**
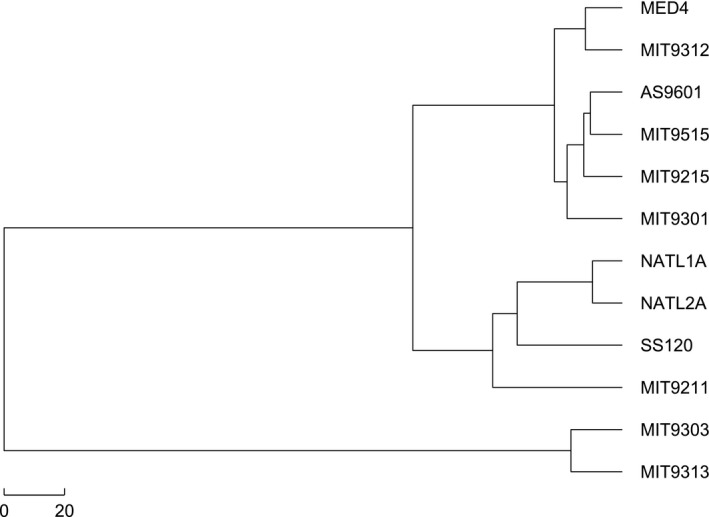
Phylogenetic tree reconstructed by the Euclidean distance based on the natural vectors of 16s‐23s rRNA sequences

From the phylogeny of the protein information, we conclude that SS120 and MIT9211 do not form a monophyletic clade, but we are further concerned about the phylogenetic relationship at the genome level. To resolve this issue, we analyzed the 16s‐23s rRNA dataset. Firstly, we got the 18‐dimensional natural vectors of the 16s‐23s rRNA of the 12 *Prochlorococcus*. We then calculated the pairwise distance between every two strains using both the Euclidean distance and the Manhattan distance. The phylogenetic tree was reconstructed by single‐linkage method based on the distance calculated above. In the 16s‐23s phylogenetic tree shown in Figure 3, the 6 HL strains clustered into a clade while the 6 LL strains formed another clade. The evolutionary relationship between SS120 and MIT9211 is consistent with the previous results (Kettler et al., 2007), indicating that these two strains do not form a separate clade.

To confirm that using our natural vector method with the Hausdorff distance is reasonable, we compared it with other methods and metrics on the same dataset. We used the full set of ribosomal proteins to make the comparisons.

**Figure 4:**
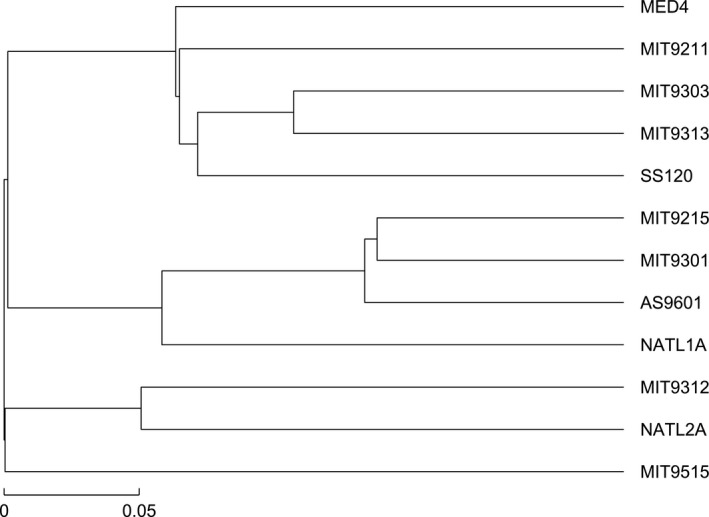
Phylogenetic tree reconstructed by 3‐mer amino acid composition method based on the full set of ribosomal proteins

The k‐mer method has been extensively applied to perform phylogenetic analyses of organisms (Vinga & Almeida, 2003). We applied this method with the Euclidean distance to our data, and the resulting phylogenetic tree is shown in Figure 4. From the phylogeny of *Prochlorococcus*, the Figure 4 did not separate the high‐light strains and low‐light strains, and we can see that the evolutionary tree reconstructed by the natural vector method with the Hausdorff distance is better than that. We conclude that the natural vector method with the Hausdorff distance outperforms other two approaches.

**Figure 5:**
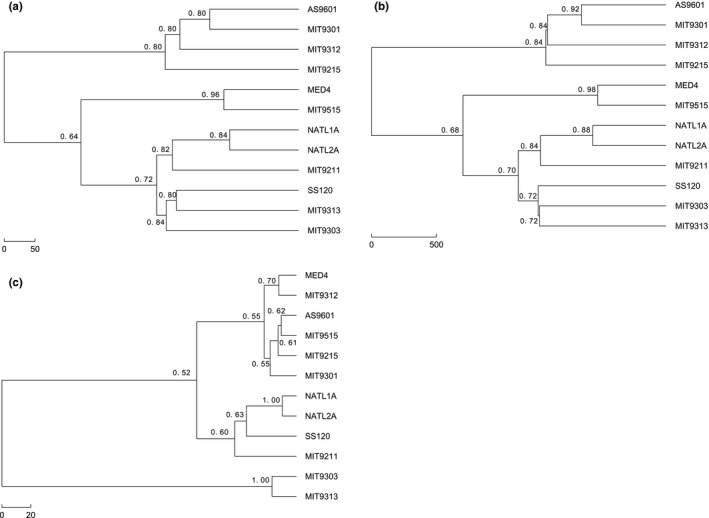
Bootstrap values on three phylogenetic trees for *Prochlorococcus* using natural vector method and single‐linkage method. (a) Phylogenetic tree reconstructed by the Euclidean distance and the Hausdorff distance based on the natural vectors of ribosomal proteins. (b) Phylogenetic tree reconstructed by the Manhattan distance and the Hausdorff distance based on the natural vectors of ribosomal proteins. (c) Phylogenetic tree reconstructed by the Euclidean distance based on the natural vectors of 16s‐23s rRNA sequences

We also used the bootstrapping method to calculate the confidence probabilities on our phylogenetic trees as shown in Figure 5. The bootstrapping protein sequences are taken from the original protein sequences using sampling with replacement. We then compared the new subtrees with the original subtree and obtained the confidence probability of the original tree. Overall, the bootstrap values in Figure 5a,b are higher than Figure 5c. The bootstrap values about strains MIT9311, MIT3013, NATL1A, and NATL2A are 100% which are higher than that in the other two figures. The bootstrap values which are related to SS120 and MIT9211 are above 70% shown in Figure 5a,b. Previous studies showed that bootstrap proportions of 70% usually correspond to a probability of 95%, which indicates that the corresponding clade is real (Hillis & Bull, 1993). These results prove that our method applied on these datasets is convincing.

### 3.2 Classification

We reconstructed the phylogeny of *Prochlorococcus* and the results indicated that SS120 and MIT9211 do not cluster into a clade. In order to validate this point, we applied classification on *Prochlorococcus*. According to the classification rules in materials and methods section, the ribosomal protein might be classified to wrong strain if the distance between two strains is very close. The dataset used in classification is a full set of ribosomal protein sequences of 12 Prochlorococcus strains shown in Table 1. The classification results of the ribosomal protein sequences are shown in Table 2.

**Table 2:** Classification result of 12 *Prochlorococcus* strains

Strains	Accuracy
AS9601	0.8868
MED4	0.8983
MIT9211	0.8286
MIT9215	0.6881
MIT9301	0.6168
MIT9303	0.8509
MIT9312	0.7982
MIT9313	0.6589
MIT9515	0.8070
NATL1A	0.8962
NATL2A	0.5310
SS120	0.9787
Total	0.7866

We can see that the classification accuracy of the 12 *Prochlorococcus* strains is from 0.6 to 0.98 and the total accuracy is 0.7866 which indicates that this classification is valid. In the classification of the ribosomal proteins, the strains which form a separate clade have a low accuracy such as MIT9303 and MIT9313, NATL1A, and NATL2A. However, the high accuracies of MIT9211 and SS120 are 0.8286 and 0.9787, respectively. This indicates that the similarity between MIT9211 and SS120 is not striking.

For each strain, we counted the number of proteins which are classified into other 11 strains by mistake. We then calculated the corresponding error rates. The strain with the highest error rate is called “most wrong strain” and the error rate is called “most error rate” which are listed in Table 3. For example, the number of ribosomal proteins for the strain MED4 is 118, and the accuracy of classification is 0.8983. We could calculate the total number of sequences which have been classified by mistake. For MED4, the number of sequences which have been classified to other 11 strains is twelve. Among the twelve sequences, there are six sequences classified to AS9601 and two sequences are classified to MIT9515. The remaining four sequences are classified to MIT9211, MIT9301, MIT9313, and NATL1A, respectively. We called AS9601 the “most wrong strain.” The “most error rate” for MED4 is 5.08% (6/118).

**Table 3:** Most wrong strains and most error rates in classification

Strains	Most wrong strain	Most error rate, %
AS9601	MIT9215	4.72
MED4	AS9601	5.08
MIT9211	SS120	3.82
MIT9215	AS9601	22.94
MIT9301	AS9601	26.17
MIT9303	MIT9313	12.28
MIT9312	AS9601	12.28
MIT9313	MIT9303	31.78
MIT9515	MED4	10.53
NATL1A	NATL2A	10.38
NATL2A	NATL1A	46.02
SS120	MIT9313	1.60

We can see that the strains which form a separate clade are likely to be classified wrongly. MIT9303 and MIT9313 form a clade while the most wrong strain of them is each other and the most error rates are 12.28% and 31.78%, respectively, which is very high. Similarly, NATL1A and NATL2A form a cluster and the most error rates are also high (10.38% and 46.02%). However, the most wrong strain of SS120 is MIT9313 but not MIT9211. Although the most wrong strain of MIT9211 is SS120 but the most error rate is only 3.82%, which is very low. This showed that the similarity between SS120 and MIT9211 is insignificant and we have reasons to believe these two *Prochlorococcus* do not form a separate clade.

### 3.3 Characteristics of SS120 and MIT9211

These two *Prochlorococcus* strains differ in a few features. Firstly, the location of SS120 is in Sargasso Sea which is located entirely in the Atlantic Ocean while MIT9211 is located in Equatorial Pacific (Rocap, Distel, Waterbury, & Chisholm, 2002). Some strains which have close locations form a monophyletic clade such as NATL1A and NATL2A, MIT9303, and MIT9313. The locations of NATL1A and NATL2A are both from the North Atlantic. The strain MIT9303 is located in Sargasso Sea and MIT9313 is located in Gulf Stream which are also close (Berube, Biller, & Kent, 2015; Kent, Dupont, Yooseph, & Martiny, 2016). Secondly, the 16s‐23s ribosomal DNA internal transcribed spacer sequences identity of the strains which could form a separate clade confirmed this point. We pay close attention to the LL strains while both SS120 and MIT9211 belong to this ecotype. For example, NATL1A and NATL2A have a high sequence identity (97%), while SS120 and MIT9211 only have a lower sequence identity of 80%. However, the sequence identity of MIT9303 and MIT9313 is very high (99%) and these two *Prochlorococcus* form a separate clade. This is also consistent with the results of 16s rDNA in this ecotype (Rocap et al., 2002).

## 4 CONCLUSION

This paper presents an effective method to analyze the evolutionary origin of *Prochlorococcus*. Our mathematical approach characterizes the protein sequence as a new natural vector according to the information in the sequence. The new vector contains more useful statistics information of sequences than the old natural vector, which could be used to get more precise results in phylogenetic analysis. In addition, we use the Hausdorff distance to measure the biological distance between the pairs of species of *Prochlorococcus*. This has turned out to be a good metric for differentiating between species and clades of *Prochlorococcus*. Comparing with multiple alignment method, the new natural vector method is alignment free and the computation time is much shorter than multiple alignment method. In this study, we also make comparisons with k‐mer method, and we conclude evolutionary tree reconstructed by the natural vector method with the Hausdorff distance is better than that. The strains MIT9211 and SS120 do not form a separate clade in the phylogeny. To confirm that the results are reasonable, we apply the classification on *Prochlorococcus*. The pairs of *Prochlorococcus* strains which form a separate clade have a high probability to be classified wrongly to each other, while MIT9211 and SS120 do not. Although MIT9211 and SS120 have a high similarity of genome content, we need to have a close look at detailed information of the genome and protein sequences, such as the distribution of nucleotides and amino acids. Using our method, we are able to analyze the phylogenetic relationship between SS120 and MIT9211. These evidences support the conclusion that these two *Prochlorococcus* strains do not form a separate clade, which implies they have undergone genome reduction independently.

## CONFLICT OF INTEREST

None declared.

## AUTHORS' CONTRIBUTIONS

XZ, KT, HL, and SSTY conceived the ideas and designed methodology; XZ collected the data; XZ and KT analyzed the data; XZ, KT, and SSTY led the writing of the manuscript. All authors contributed critically to the drafts and gave final approval for publication.

## DATA ACCESSIBILITY

The datasets used in this paper are available as Data S1 and Data S2.
