# Supplementary material for: Establishing the phylogeny of Prochlorococcus with a new alignment‐free method
Source: Ecol Evol. 2017 Nov 15;7(24):11057–65. doi: 10.1002/ece3.3535 (PMC5743538; doi:10.1002/ece3.3535)
Supplement: Supplementary file 1 [file ECE3-7-11057-s001.docx]

>gi|18462994|gb|AF397677.1| Prochlorococcus marinus str. AS9601 16S ribosomal RNA gene, partial sequence; internal transcribed spacer, complete sequence; tRNA-Ile and tRNA-Ala genes, complete sequence; and 23S ribosomal RNA gene, partial sequence

AGCTAGCAAATCCCATAAACCGTGGCTCAGTTCAGATCGTAGGCTGCAACTCGCCTACGTGAAGTAGGAA

TCGCTAGTAATCGCAGGTCAGCATACTGCGGTGAATACGTTCCCGGGCCTTGTACACACCGCCCGTCACA

CCATGGAAGTTGGCCATGCCCGAAGTCGTTACTCCAACCCTTGTGGAGGAGGACGCCGAAGGTGGGGCTA

ATGACTGGGGTGAAGTCGTAACAAGGTAGCCGTACCGGAAGGTGCGGCTGGATCACCTCCTAACAGGGAG

ACAACAAAATGTTTAATATTATTATTTTGTCACCTTAGGTCGATCGGTATCTCACGTTCTTAATTTATTA

AGAATTTCATTTCCTAAGTTTTTCTAGGTCACACCCATTATTTTCCTGGGCCATTAGCTCAGGTGGTTAG

AGCGCACCCCTGATAAGGGTGAGGTCCCTGGTTCAAGTCCAGGATGGCCCATATCTCTATGTTGGGGGTA

TAGCTCAGTTGGTAGAGCGCCTGCTTTGCAAGCAGGATGTCAGCGGTTCGAGTCCGCTTACCTCCACTGA

TTACCACCTCTTCCATAATCTATAGAAAGGAAAAATTTTGAGTTGTGATCAAATTTCTGAACGAATCTAG

CTTCCTAATGAAACCATTAAGATGCTGGACTCATTGAATTAATTTCATTGTTTTCAGAGAACCTTGACAA

CTGCATAGATTATTTTTAAAGACAATAAGCATCTTTAATGATGCAGATTTATTATTTGTGCAAATGCATT

AATAACAATTCTATTAAGCTGATGCTTCAATTATGAAGTTATTGGTCAAGCTACAAAGGGCTCACGGAGG

ATACCTAGGCACACAGAGGCGATGAAGGACGTGGTTACCTGCGATAAGTCTCGGGGAGTTGGAAGCACAC

TTTGATCCGGGAATTTCCGAATGGGGCAACCCCATGTACGGCCAACTGAATATATAGGTTGGTGCGAGCT

AACCCAGCGAACTGAAACATCTTA

>gi|18462990|gb|AF397673.1| Prochlorococcus marinus subsp. pastoris str. CCMP1378 16S ribosomal RNA gene, partial sequence; internal transcribed spacer, complete sequence; tRNA-Ile and tRNA-Ala genes, complete sequence; and 23S ribosomal RNA gene, partial sequence

ACGGACAAAGGGCAGCAAACTCGCGAGAGCTAGCAAATCCCATAAACCGTGGCTCAGTTCAGATCGTAGG

CTGCAACTCGCCTACGTGAAGTAGGAATCGCTAGTAATCGCAGGTCAGCATACTGCGGTGAATACGTTCC

CGGGCCTTGTACACACCGCCCGTCACACCATGGAAGTTGGCCATGCCCGAAGTCGTTACTCCAACCCTTG

TGGAGGAGGACGCCGAAGGTGGGGCTAATGACTGGGGTGAAGTCGTAACAAGGTAGCCGTACCGGAAGGTGCGGCTGGATCACCTCCTAACAGGGAGACAACAAAATGTAAATATTAATTTTGTCACCTTAGGTCGATCG

GTATCTCAGATTCTTATTTTTTAAGAATTTCAGTTTCTAAGTTTTTTCTAGGTCACACCCATCACTTTTT

CCTGGGCCATTAGCTCAGGTGGTTAGAGCGCACCCCTGATAAGGGTGAGGTCCCTGGTTCAAGTCCAGGA

TGGCCCATATCTCTATGTTGGGGGTATAGCTCAGTTGGTAGAGCGCCTGCTTTGCAAGCAGGATGTCAGC

GGTTCGAGTCCGCTTACCTCCACTGAATACCACCTCTTACATATTTAAAGAGGGAAATTGTTTTGAGTCG

TGTCCTAATTTGCTCTGAGCGAATCTAGCTTCCTATTTTATTCAATAAATAAGATGCTGGACTCATTGAA

TTTTTTTTCATTGTTTTCAGAGAACCTTGACAACTGCATAGATTATTTTTGAAGACAATAAGCATCTTTA

ATGATGCAGATTTATTATTTGTGCGAATGCATTAAATAGCAATTCTATTAAGCTGATGCTTCAATTATTG

AAGTTATTGGTCAAGCTACAAAGGGCTCACGGAGGATACCTAGGCACACAGAGGCGATGAAGGACGTGGT

TACCTGCGATAAGTCTCGGGGAGTTGGAAGCACACTTTGATCCGGGAATTTCCGAATGGGGCAACCCCTT

GAACGACCAACTGAATATATAGGTTGGTGCGAGCTAACCCAGCGAACTGAAACATCTTA

>gi|18463019|gb|AF397702.1| Prochlorococcus marinus str. MIT 9211 16S ribosomal RNA gene, partial sequence; internal transcribed spacer, complete sequence; tRNA-Ile and tRNA-Ala genes, complete sequence; and 23S ribosomal RNA gene, partial sequence

CAAGCAAATCCCATAAACCGTGGCTCAGTTCAGATCGTAGGCTGCAACTCGCCTACGTGAAGTAGGAATC

GCTAGTAATCGCAGGTCAGCATACTGCGGTGAATACGTTCCCGGGCCTTGTACACACCGCCCGTCACACC

ATGGAAGTTGGCCACGCCCGAAGTCGTTACTCCAACCCTTGTGGAGGAGGACGCCGAAGGTGGGGCTAAT

GACTGGGGTGAAGTCGTAACAAGGTAGCCGTACCGGAAGGTGCGGCTGGATCACCTCCTAACAGGGAGAC

AAAACTGATTATGATGTTTGGATAAAAAGTTCAGGCCATAATCCTGTCACCTTAGGTCGATCGGTACCTC

AGGTTGAGAACTATACAAATCATGAGAAATCATTGATTCTTTAGTTTTTAATTTCAGTTCCTAAGCTTGT

CTAGGTCACACCCAACAAAACTTTCTTCTGGGCTATTAGCTCAGGTGGTTAGAGCGCACCCCTGATAAGG

GTGAGGTCCCTGGTTCAAGTCCAGGATGGCCCATTCGTTGTTGGGGGTATAGCTCAGTTGGTAGAGCGCC

TGCTTTGCAAGCAGGATGTCAGCGGTTCGAGTCCGCTTACCTCCACTGATCCACTGCCTGATAACTCAAT

TTATGGAGATTGATATTTGTTAAATGTGATTTAGACGTTGTTCAACTGATTGAACCTAGCTTCCTATTAC

CCTTTTTTATAAAGGCTGGTAAGACGCTGGGCTCACTGTCCTTCTAAAGGACAGTTAAGTTCAGTAGAAC

CTTGACAACTGCATATGTGAGTCTGGAAAGAATTAAAGCATCTAAATAGATCCATAAATTCTATTGATTC

TATTAATTGAATTTATGTTCAATTCTAATAATTAAGAGCCGAGAAATTTATTTGTTTATTACTTCTATAG

ATGGAAAATATTCATTTATAAAAGTCTAATAACCATCAAATAAATTAACTAAATGGTCAAGCTACAAAGG

GCTCACGGTGGATACCTTGGCACACAGAGGCGATGAAGGACGTGGTTACCTGCGATAAGTCTCGGGGAGC

TGGAAACACGCTTTGATCCGGGAATTTCCGAATGGGGCAACCCTTAGAACGACCAGCTGAATTCATAGGC

TGGAACGAGCCAACCCAGCGAACTGAAACATCTTA

>gi|18463001|gb|AF397684.1| Prochlorococcus marinus str. MIT 9215 16S ribosomal RNA gene, partial sequence; internal transcribed spacer, complete sequence; tRNA-Ile and tRNA-Ala genes, complete sequence; and 23S ribosomal RNA gene, partial sequence

CAAACTCGCGAGAGCTAGCAAATCCCATAAACCGTGGCTCAGTTCAGATCGTAGGCTGCAACTCGCCTAC

GTGAAGTAGGAATCGCTAGTAATCGCAGGTCAGCATACTGCGGTGAATACGTTCCCGGGCCTTGTACACA

CCGCCCGTCACACCATGGAAGTTGGCCATGCCCGAAGTCGTTACTCCAACCCTTGTGGAGGAGGACGCCG

AAGGTGGGGCTAATGACTGGGGTGAAGTCGTAACAAGGTAGCCGTACCGGAAGGTGCGGCTGGATCACCT

CCTAACAGGGAGACAACAAAATGTTTAATATTATTATTTTGTCACCTTAGGTCGATCGGTATCTCACATT

CTTAATTTATTAAGAATTTCAACTCCTAAGTTTTTCTAGGTCACACCCATTATTTCCCTGGGCCATTAGC

TCAGGTGGTTAGAGCGCACCCCTGATAAGGGTGAGGTCCCTGGTTCAAGTCCAGGATGGCCCATATCTCT

ATGCTGGGGGTATAGCTCAGTTGGTAGAGCGCCTGCTTTGCAAGCAGGATGTCAGCGGTTCGAGTCCGCT

TACCTCCACTGAATACCACCTCTGCCATATTTTGTAAAAGGGAAATATTATGAGTTGTGATCAAATTCTG

AACGAATCTAGCTTCCTAATGAAATCATTAAGATGCTGGACTCATTGAATTAATTTCATTGTTTTCAGAG

AACCTTGACAACTGCATAGATTATTTTTAAAGACAATAAGCATCTTTAATGATGCAGATTTATTATTTGT

GCGAATGCATTAATAACAATTCTATTAAGCTGATGCTTCAATTTTGAAGTTATTGGTCAAGCTACAAAGG

GCTCACGGAGGATACCTAGGCACACAGAGGCGATGAAGGACGTGGTTACCTGCGATAAGTCTCGGGGAGT

TGGAAGCACACTTTGATCCGGGAATTTCCGAATGGGGCAACCCCATGTACGGCCAACTGAATATATAGGT

TGGTGCGAGCTAACCCAGCGAACTGAAACATCTTA

>gi|18463002|gb|AF397685.1| Prochlorococcus marinus str. MIT 9301 16S ribosomal RNA gene, partial sequence; internal transcribed spacer, complete sequence; tRNA-Ile and tRNA-Ala genes, complete sequence; and 23S ribosomal RNA gene, partial sequence

TAAACCGTGGCTCAGTTCAGATCGTAGGCTGCAACTCGCCTACGTGAAGTAGGAATCGCTAGTAATCGCA

GGTCAGCATACTGCGGTGAATACGTTCCCGGGCCTTGTACACACCGCCCGTCACACCATGGAAGTTGGCC

ATGCCCGAAGTCGTTACTCCAACCCTTGTGGAGGAGGACGCCGAAGGTGGGGCTAATGACTGGGGTGAAG

TCGTAACAAGGTAGCCGTACCGGAAGGTGCGGCTGGATCACCTCCTAACAGGGAGACAACAAAATGTTTA

ATATTATTATTTTGTCACCTTAGGTCGATCGGTATCTCACGTTTTTAATTTATTAAGAATTTCATTTCCT

AAGTTTTTCTAGGTCACACCCATTATTTTTCCTGGGCCATTAGCTCAGGTGGTTAGAGCGCACCCCTGAT

AAGGGTGAGGTCCCTGGTTCAAGTCCAGGATGGCCCATATCTCTATGTTGGGGGTATAGCTCAGTTGGTA

GAGCGCCTGCTTTGCAAGCAGGATGTCAGCGGTTCGAGTCCGCTTACCTCCACTGATTACCACCTCTTCC

ATAACCGGTAGAAAGGAAATGTTTTGAGTTGTGATCAAATTCTGAAAGAATCTAGCTTCCTAATGAAATC

ATTAAGATGCTGGACTCATTGAATTAATTTCATTGTTTTCAGAGAACCTTGACAACTGCATAGATTATTT

TTAAAGACAATAAGCATCTTTAATGATGCAGATTTATTATTTGTGCGAATGCATTAAATAACAATTCTAT

TAAGCTGATGCTTCAATTTTTGAAGTTATTGGTCAAGCTACAAAGGGCTCACGGAGGATACCTAGGCACA

CAGAGGCGATGAAGGACGTGGTTACCTGCGATAAGTCTCGGGGAGTTGGAAGCACACTTTGATCCGGGAA

TTTCCGAATGGGGCAACCCCATGTACGGCCAACTGAATATATAGGTTGGTGCGAGCTAACCCAGCGAACT

GAAACATCTTA

>gi|18463020|gb|AF397703.1| Prochlorococcus marinus str. MIT 9303 16S ribosomal RNA gene, partial sequence; internal transcribed spacer, complete sequence; tRNA-Ile and tRNA-Ala genes, complete sequence; and 23S ribosomal RNA gene, partial sequence

ACGGACAAAGGGCAGCAAGTTCGCGAGGACAAGCAAATCCCATAAACCGTGGCTCAGTTCAGATCGTAGG

CTGCAACTCGCCTACGTGAAGAAGGAATCGCTAGTAATCGCAGGTCAGCATACTGCGGTGAATACGTTCC

CGGGCCTTGTACACACCGCCCGTCACACCATGGAAGTTGGCCACGCCCGAAGTCGTTACTCCAACCCTTG

TGGAGGAGGACGCCGAAGGTGGGGCTGATGACTGGGGTGAAGTCGTAACAAGGTATCCGTACCGGAAGGT

GCGGATGGATCACCTCCTAACAGGGAGACAACACAATGATTTTGATGTCTGAGTATTTTAATTCTTAGGC

CGAAATCCTGTCACCTTAGGTCGATCGGTACCTCAAGTTGAGAATCAAAGAAAAGCTGAGTAATCAGCTT

GGAGATTGATGATTGATTTTAGTTCCTAAACTTGTCTAGGTCACACCCCGCAAGGGTTACTCCTGGGCCA

TTAGCTCAGGTGGTTAGAGCGCACCCCTGATAAGGGTGAGGTCCCTGGTTCAAGTCCAGGATGGCCCATT

CGGTGTTGGGGGTTTAGCTCAGTTGGTAGAGCGCCTGCTTTGCAAGCAGGATGTCAGCGGTTCGAGTCCG

CTAACCTCCACTGACTGATCTCCTACAACGAGCCAATGGTGAGAACCGTTGTGTGATGTGATTTAGAATT

TAAGTTTGCTGGAAGACCCTAGCTTCTTATCATTCCAGGCCTCAATTAATTGAGGTTAGGTTGATAGGAT

GCTGGGCTCACTGTAATTCGCAAGAATTGCAGAGATGTTCAGCAGAACCTTGACAACTGCATAGGTAAGT

CTGGAAAGAATAAAGCATCTTCATGGATGCATGATTCTTGGGAATCAGTTAATGCAATCTGCGTCAGTAG

AGAGCTGAGGTTGATGAACGAGAATGATGTTTGATTCTTGAGTCAAGAGCCGAGACTCTATAGCGTTCTT

TCGATGTCATCGAGGCATCAAGAGTTTGATTGGTCATTCAAACCATCTTCAATGACAAACAATTCAGTTT

AACTGGAGAAGTTTGAGATTGAAGCCAGGTGAATTGCTAAGAATACGTATTCAACTACGTGAAGCGTTAT

AGAGATTAATTGGTCAAGCTACAAAGGGCTCATGGTGGATACCTTGGCACACAGAGGCGATGAAGGACGT

AGTTACCTGCGATAAGTCTCGGGGAGCTGGACACACGCTTTGATCCGGGAATTTCCGAATGGGGCAACCC

CTAGTACGGCCAGCTGAATCCATAGGCTGGTGCGAGCCAACCCAGCGAACTGAAACATCTTA

>gi|18463005|gb|AF397688.1| Prochlorococcus marinus str. MIT 9312 16S ribosomal RNA gene, partial sequence; internal transcribed spacer, complete sequence; tRNA-Ile and tRNA-Ala genes, complete sequence; and 23S ribosomal RNA gene, partial sequence

ACGGACAAAGGGCAGCAAACTCGCAAGAGCTAGCAAATCCCATAAACCGTGGCTCAGTTCAGATCGTAGG

CTGCAACTCGCCTACGTGAAGTAGGAATCGCTAGTAATCGCAGGTCAGCATACTGCGGTGAATACGTTCC

CGGGCCTTGTACACACCGCCCGTCACACCATGGAAGTTGGCCATGCCCGAAGTCGTTACTCCAACCCTTG

TGGAGGAGGACGCCGAAGGTGGGGCTAATGACTGGGGTGAAGTCGTAACAAGGTAGCCGTACCGGAAGGT

GCGGCTGGATCACCTCCTAACAGGGAGACAACAAAATGTTTAATATTATTATTTTGTCACCTTAGGTCGA

TCGGTATCTCACATTCTCGATTTATTGAGGATTTCATTTCCTAAGTTTTTCTAGGTCACACCCATTATTT

TCCCTGGGCCATTAGCTCAGGTGGTTAGAGCGCACCCCTGATAAGGGTGAGGTCCCTGGTTCAAGTCCAG

GATGGCCCATATCTCTATGTTGGGGGTATAGCTCAGTTGGTAGAGCGCCTGCTTTGCAAGCAGGATGTCA

GCGGTTCGAGTCCGCTTACCTCCACTGATCACCACCTCTTCCATAATTTATAGAAGGGAATGTTTTGGAG

TTGTGATCAAATCTGAACGAATCTAGCTTCCTAATGAAATCATTAAGATGCTGGACTCATTGAATTAATT

TCATTGTTTTCAGAGAACCTTGACAACTGCATAGATTATTTTTAAAGACAATAAGCATCTTTAATGATGC

AGATTTATTATTTGTGCGAATGCATTAATAACAATTCTATTAAGCTGATGCTTCAATTTTTGAAGTTATT

GGTCAAGCTACAAAGGGCTCACGGAGGATACCTAGGCACACAGAGGCGATGAAGGACGTGGTTACCTGCG

ATAAGTCTCGGGGAGTTGGAAGCACACTTTGATCCGGGAATTTCCGAATGGGGCAACCCCATGTACGGCC

AACTGAATATATAGGTTGGTGCGAGCTAACCCAGCGAACTGAAACATCTTA

>gi|18463021|gb|AF397704.1| Prochlorococcus marinus str. MIT 9313 16S ribosomal RNA gene, partial sequence; internal transcribed spacer, complete sequence; tRNA-Ile and tRNA-Ala genes, complete sequence; and 23S ribosomal RNA gene, partial sequence

ACGGACAAAGGGCAGCGAACTCGCGAGGGCAAGCAAATCCCATAAACCGTGGCTCAGTTCAGATCGTAGG

CTGCAACTCGCCTACGTGAAGAAGGAATCGCTAGTAATCGCAGGTCAGCATACTGCGGTGAATACGTTCC

CGGGCCTTGTACACACCGCCCGTCACACCATGGAAGTTGGCCACGCCCGAAGTCGTTACTCCAACCCTTG

TGGAGGAGGACGCCGAAGGTGGGGCTGATGACTGGGGTGAAGTCGTAACAAGGTAGCCGTACCGGAAGGT

GCGGCTGGATCACCTCCTAACAGGGAGACAACAACTGATCGTGATGTCTGAGTTATTGATACTTAGGCCA

TGATCCTGTCACCTTAGGTCGATCGGTACCTCAAGTTGAGAATCAAAGAAAAGCTGAGTAATCAGCTTGG

AGATTGATGATTGATTTTAGTTCCTAAACTTGTCTAGGTCACACCCCGCAAGGGTTACTCCTGGGCCATT

AGCTCAGGTGGTTAGAGCGCACCCCTGATAAGGGTGAGGTCCCTGGTTCAAGTCCAGGATGGCCCATTCG

GTGTTGGGGGTTTAGCTCAGTTGGTAGAGCGCCTGCTTTGCAAGCAGGATGTCAGCGGTTCGAGTCCGCT

AACCTCCACTGACGAATCTTCTGACAACGAGCCAATGGTGAGAACCGTTGTGTGATGTGATTTAGAATTT

AAGTTTGCTGGCAGACCCTAGCTTCTTATCATTCCAGGACTCAATCAATTGAGATGAGGTTGATAGGACG

CTGGGCTCACTGTAATTCGCAAGAATTGCAGAGATGTTCAGCAGAACCTTGACAACTGCATAGGTAAGTC

TGGAAAGAATAAAGCATCTCATGGATGCATGATTCTTGGGAATCAGCTGATGCAATCTGCGCGAGCAGAG

AGCTGAGGTTGATCAACGAGAATGATGTTTGATTCTTGAGTCAAGAGCCGAGACTCTATAGCGTTCTTTC

GATGTCATCGAGGCATCGAGAGTTTGATTGGTGATTCAAACCATCTTCAATGACAAACAATTCAGTTTAA

CTGGAGAAGTTTGAGATTGAAGCCAGGTAAATTGCTAAGAATACGTATTCAACTACGTGAAGCGTTATAG

AGATTAATTGGTCAAGCTACAAAGGGCTCATGGTGGATACCTTGGCACACAGAGGCGATGAAGGACGTAG

TTACCTGCGATAAGTCTCGGGGAGCTGGACACACGCTTTGATCCGGGAATTTCCGAATGGGGCAACCCCT

AGTACGGCCAGCTGAATCCATAGGCTGGTGCGAGCCAACCCAGCGAACTGAAACATCTTA

>gi|18462992|gb|AF397675.1| Prochlorococcus marinus str. MIT 9515 16S ribosomal RNA gene, partial sequence; internal transcribed spacer, complete sequence; tRNA-Ile and tRNA-Ala genes, complete sequence; and 23S ribosomal RNA gene, partial sequence

CTAGCAAATCCCATAAACCGTGGCTCAGTTCAGATCGTAGGCTGCAACTCGCCTACGTGAAGTAGGAATC

GCTAGTAATCGCAGGTCAGCATACTGCGGTGAATACGTTCCCGGGCCTTGTACACACCGCCCGTCACACC

ATGGAAGTTGGCCATGCCCGAAGTCGTTACTCCAACCCTTGTGGAGGAGGACGCCGAAGGTGGGGCTAAT

GACTGGGGTGAAGTCGTAACAAGGTAGCCGTACCGGAAGGTGCGGCTGGATCACCTCCTAACAGGGAGAC

AACAAAATGTAAATATTAATTTTGTCACCTTAGGTCGATCGGTATCTCAGGTTCTTAATTTTAAGAATTT

CAGTTTCTAAGTTTTTTCTAGGTCACACCCATCACTTTTTCCTGGGCCATTAGCTCAGGTGGTTAGAGCG

CACCCCTGATAAGGGTGAGGTCCCTGGTTCAAGTCCAGGATGGCCCATATCTCTATGTTGGGGGTATAGC

TCAGTTGGTAGAGCGCCTGCTTTGCAAGCAGGATGTCAGCGGTTCGAGTCCGCTTACCTCCACTGAATAC

CACCTCTTACATATAAATAAAGAGGGAAATTGTTTTGAGTCGTGTCCTATAGCTCTGAGCGAATCTAGCT

TCCTATTTCTTTAATTAAATAAGATGCTGGACTCATTGAATTATTATTCATTGTTTTCAGAGAACCTTGA

CAACTGCATAGATTATTTTTGAAGACAATAAGCATCTTTAATGATGCAGATTTATTATTTGTGCGAATGC

ATCAAATAGCAATTCTATTAAGCTGATGCTTCAATTATTGAAGTTATTGGTCAAGCTACAAAGGGCTCAC

GGAGGATACCTAGGCACACAGAGGCGATGAAGGACGTGGTTACCTGCGATAAGTCTCGGGGAGTTGGAAG

CACACTTTGATCCGGGAATTTCCGAATGGGGCAACCCCTTGAACGACCAACTGAATATATAGGTTGGTGC

GAGCTAACCCAGCGAACTGAAACATCTTA

>gi|18463011|gb|AF397694.1| Prochlorococcus marinus str. NATL1A 16S ribosomal RNA gene, partial sequence; internal transcribed spacer, complete sequence; tRNA-Ile and tRNA-Ala genes, complete sequence; and 23S ribosomal RNA gene, partial sequence

CTAGCAAATCCCATAAACCGTGGCTCAGTTCAGATCGTAGGCTGCAACTCGCCTACGTGAAGTAGGAATC

GCTAGTAATCGCAGGTCAGCATACTGCGGTGAATACGTTCCCGAGCCTTGTACACACCGCCCGTCACACC

ATGGAAGTTGGCCACGCCCGAAGTCGTTACTTTAACCCTTGTGGAGAAGGACGCCGAAGGTGGGGCTGAT

GACTGGGGTGAAGTCGTAACAAGGTAGCCGTACCGGAAGGTGCGGCTGGATCACCTCCTAACAGGGAGAC

AATAAATTGATTGTGATGTCTAAGTTATTTATTCTTAGGCCGCAATCCTGTCACCTTAAGGTCGATCGGT

ACCTCAGATTTTTGAATTAGTTTTACAATTGATTCTTTGATTTCAGTTCCTAAACTTGTCTAGGTCACAC

CCAACAAAGGTTTCTCCTGGGCCATTAGCTCAGGTGGTTAGAGCGCACCCCTGATAAGGGTGAGGTCCCT

GGTTCAAGTCCAGGATGGCCCATTCGTTGTTGGGGGTATAGCTCAGTTGGTAGAGCGCCTGCTTTGCAAG

CAGGATGTCAGCGGTTCGAGTCCGCTTACCTCCACTGAAAACTATCCTGATAACTAAATTTCTGGAGAAA

AAAATTGTAGATGTGGCTTAGAATTGTCTATTTGAAAAGAACCTAGCTTCTTGTCATCTTTTTATAGTTG

ATAAAATGCTGGGCTCGCATGGATTATTTCCATGAGAATTCAGTAGAACCTTGAAAACTGCATAGATTAG

AAAGAATAAAGCATCTCATGGATGCATAGTTCTGTTTTTATAATTTAGCCTTAAAACTAAATTATTTAAT

TCTAGAATTCATGTTTAATTCTTGAGTAATAGCCGAGCACAATTGATTTTGTGATTGTATTAAAGGTCAA

GCTACAAAGGGCTCATGGCGGATACCTTGGCACACAGAGGCGATGAAGGACGTGGTTACCTGCGATATGT

CTCGGGGAGCTGGATACACGCTTTGATCCGGGAATTTCCGAATGGGGCAACCCTTAGAACGGCCAGCTGA

ATACATAGGCTGGCGCGAGCCAACCCAGCGAATTGAAACATCTTA

>gi|18463012|gb|AF397695.1| Prochlorococcus marinus str. NATL2A 16S ribosomal RNA gene, partial sequence; internal transcribed spacer, complete sequence; tRNA-Ile and tRNA-Ala genes, complete sequence; and 23S ribosomal RNA gene, partial sequence

CTAGCAAATCCCATAAACCGTGGCTCAGTTCAGATCGTAGGCTGCAACTCGCCTACGTGAAGTAGGAATC

GCTAGTAATCGCAGGTCAGCATACTGCGGTGAATACGTTCCCGGGCCTTGTACACACCGCCCGTCACACC

ATGGAAGTTGGCCACGCCCGAAGTCGTTACTTTAACCCTTGTGGAGAAGGACGCCGAAGGTGGGGCTGAT

GACTGGGGTGAAGTCGTAACAAGGTAGCCGTACCGGAAGGTGCGGCTGGATCACCTCCTAACAGGGAGAC

AATAAATTGATTGTGATGTCTAAGTCATTTATTCTTAGGCCACAATCCTGTCACCTTAAGGTCGATCGGT

ACCTCAGGTTTTTGAATTAGTTTTATAATTAATTCTTTGATTTCAGTTCCTAAACTTGTCTAGGTCACAC

CCAACAAAGGTTTCTCCTGGGCCATTAGCTCAGGTGGTTAGAGCGCACCCCTGATAAGGGTGAGGTCCCT

GGTTCAAGTCCAGGATGGCCCATTCGTTGTTGGGGGTATAGCTCAGTTGGTAGAGCGCCTGCTTTGCAAG

CAGGATGTCAGCGGTTCGAGTCCGCTTACCTCCACTGAAAACTATCCTGATAACTAAATTTCGGAGAAAA

ATTTGTAGAAGTGGCTTAGAATTGTCTGTCTGAAAAGAACCTAGCTTCTTGTCATCTTTTTATAGTTGAT

AACATGCTGGGCTCGCATGGATTAATTCCATGAGAAATCAGTAGAACCTTGAAAACTGCATAGATTAGAA

AGAATAAAGCATCTCATGGATGCATAATTCTGATTTTTTTAATTTAGCCTCAAAACTAAATTACTTAGAT

CTAGAATTCATGTTTAATTCTTGAGTAAAAGCCGAGCACAATTGATTTTGTGATTGTATTTAAGGTCAAG

CTACAAAGGGCTCATGGCGGATACCTTGGCACACAGAGGCGATGAAGGACGTGGTTACCTGCGATATGTC

TCGGGGAGCTGGATACACGCTTTGATCCGGGAATTTCCGAATGGGGCAACCCTTAGAACGGCCAGCTGAA

TATATAGGCTGGCACGAGCCAACCCAGCGAACTGAAACATCTTA

>gi|18463018|gb|AF397701.1| Prochlorococcus marinus subsp. marinus str. CCMP1375 16S ribosomal RNA gene, partial sequence; internal transcribed spacer, complete sequence; tRNA-Ile and tRNA-Ala genes, complete sequence; and 23S ribosomal RNA gene, partial sequence

TCCCATAAACCGTGGCTCAGTTCAGATCGTAGGCTGCAACTCGCCTACGTGAAGCAGGAATCGCTAGTAA

TCGCAGGTCAGCATACTGCGGTGAATACGTTCCCGGGCCTTGTACACACCGCCCGTCACACCATGGAAGT

TGGCCACGCCCGAAGTCGTTACTCCAACCCTTGTGGAGGAGGACGCCGAAGGTGGGGCTGATGACTGGGG

TGAAGTCGTAACAAGGTAGCCGTACCGGAAGGTGCGGCTGGATCACCTCCTAACAGGGAGACAACAATCG

ATTGTGATGTCTAATTTTCAAAATTTAGGCCATAATCCTGTCACCTTAGGTCGATCGGTACCTCAAAGTA

ATAACTTAAAAAATTGAGCAATCACTTTATAAATAGTTTTTATTTTCAGTTCCTAAACTTGTCTAGGTCA

CACCCAACAAACTTTCTCCTGGGCTATTAGCTCAGGCGGTTAGAGCGCACCCCTGATAAGGGTGAGGTCC

CTGGTTCAAGTCCAGGATGGCCCATTCGTTGTTGGGGGTATAGCTCAGTTGGTAGAGCGCCTGCTTTGCA

AGCAGGATGTCAGCGGTTCGAGTCCGCTTACCTCCACTGATCAACTACCTGATAACTCAATTTGGAGATT

ATCGTTTGTTTAATGTGACTTAGAGTTGTTCGTCTGAATGAACCTAGCTTCCTATCATTCCTTTCTTTTA

GGCCGATAAGACGCTGGGCTCAACTAATATTCTTCAGATTATTAGTTTGATTCAGCAGAACCTTGACAAC

TGCATAGGAAAAGTCTGGAAAGAATAAAGCATCTCATAGATGCACTGAAATTTACTTATTAAGCAAAGCT

AAATAAAGAGATTTCAATGCTTAATTCTTGAGTTAGAGCATATTTACTAAAATTTAGTTATTTCAAATAA

CTTTTTAGTGATTATTAATGGTCAAGCTACAAAGGGCTCACGGTGGATACCTTGGCACACAGAGGCGATG

AAGGACGTGGTTACCTGCGATAAGTCTCGGGGAGCTGGAAGCACGCTTTGATCCGGGAATTTCCGAATGG

GGCAACCCTTAATACGGCCAGCTGAATATATAGGCTGGAACGAGCCAACCCAGCGAACTGAAACATCTTA
