# Supplementary material for: Establishing the phylogeny of Prochlorococcus with a new alignment‐free method
Source: Ecol Evol. 2017 Nov 15;7(24):11057–65. doi: 10.1002/ece3.3535 (PMC5743538; doi:10.1002/ece3.3535)
Supplement: Supplementary file 2 [file ECE3-7-11057-s002.docx]

>gi|123197983|gb|ABM69624.1| 30S ribosomal protein S1, protein A [Prochlorococcus marinus str. AS9601]

MNENSSQTIKEISEDQEIKNSSELDNNSASQNEEDLSFEKSDIPQADSSSSRTNTDFDNAGFTQEEFASL

LGKYDYNFKPGDLVKGTVFALEPKGAMIDIGAKTAAFMPVQEVSINRVEGLNDVLQPSESREFFIMSEEN

EDGQLALSIRRIEYQRAWERVRQLQKEDATIYSEVFATNRGGALVRVEGLRGFIPGSHISARRIKDDLEG

EYLPLKFLEVDEERNRLVLSHRRALVEKKMNRLEVGEVVVGSVKGIKPYGAFIDIGGVSGLLHISEISHE

HIETPHNVLNVSDQMKVMIIDLDSERGRISLSTKALEPEPGDMLTDPQKVFSKAEEMAAKYKQMLFEQTD

ENEEIATASAETL

>gi|123198231|gb|ABM69872.1| 30S ribosomal protein S1 protein B, putative Nbp1 [Prochlorococcus marinus str. AS9601]

MGASNKNAQDNIQPKGNKKPLQVLHISKKDSQEINNEQNNSQEDIKKENIAIKPQIIKDNSVKEIEDSNE

KTKDFDISQQDLTQQDLNRPLNFSEQKIDFQLERTVDEFDFDESAFLEALNANEPIGATGETISGKVIAI

ESDGLYVDIGGKAPGYMPKKECGLGVITNFKEKFSIGLEMEVLVIKEQNADGMVTVSARALILRQSWEKV

SNSAKNGELINVLINGFNRGGLTCDVDGLRGFIPRSQLEDGQDYQSFVGKNLKVAFLEVNPESRKLVLSE

KKASLVSKLTSLELGQLIEGEVLAVKPYGFFIDLGGASGLLHQSSLTNGSIRSLREVFREGEVIKALISE

IDLEKGRIGLNTALLENSAGELIIDKQKVMQEATERALKTKALFDKKEQDK

>gi|123199524|gb|ABM71165.1| 50S ribosomal protein L9 [Prochlorococcus marinus str. AS9601]

MAKRVQVALTESIASLGKEGDLVDVAPGYARNFLLPYGKAMNVTPAVLKQIERKKEKEKIAAGKLKQEAL

DFQTALSTIGRFTIKKQVGEDGVLFGTVTNGDVAEAIEAATKKEIDRRNITVPDIHNLGAFTAKIKLHPE

VNAEVNIEVTS

>gi|123199393|gb|ABM71034.1| 50S ribosomal protein L6 [Prochlorococcus marinus str. AS9601]

MSRIGKTPVLIPEKVTVDFDGLTVTVKGPKGELKRLMPEGVSFDKKDNTVVVSPTTTKIHSRQRHGLCRA

LIANMVEGVTQGFSKKLEIVGVGSRAQVKGKNLVVSAGYSHPVEMIPPDGITYKVESNTNVTVSGIDKEI

VGNEAAKIRSIRPPEPYKGKGIKYHDERILRKAGKSGKK

>gi|123199391|gb|ABM71032.1| 30S ribosomal protein S5 [Prochlorococcus marinus str. AS9601]

MTDTPTKQEIQSKNDNVPAATPVEQKKNNRNDRKRNRRGDSKNLERDSDWQERVVQIRRVSKTVKGGKKM

SFRAIVVVGNEKGQVGVGVGKAGDVIGAVRKGVSDGKKNLVRVPLTPNNSIPTLSLGSDGAANVLIRPAA

PGTGVIAGGSIRTVLELAGIKNVLAKRLGSKTPLNNARAAMVALSQLRTHKSVSRERGISLEQLYS

>gi|123197869|gb|ABM69510.1| 50S ribosomal protein L11 [Prochlorococcus marinus str. AS9601]

MAKKIVAVIKLALQAGKANPAPPVGPALGQHGVNIMAFCKEYNARTQDKAGFVIPVEISVFEDRSFTFIT

KTPPASVLITKAAGIEKGSGESAKGSVGNISKAQLEEIAKTKLPDLNCSSVESAMKVIEGTARNMGVSIT

D

>gi|123197867|gb|ABM69508.1| 50S ribosomal protein L10 [Prochlorococcus marinus str. AS9601]

MGRTLENKQKIVTEIKSLLDDSEMAVVLDYKGLTIKEMSDLRSRLQTTNGICKVTKNSLMRKAIDGDSNW

NDLESLLTGTNAFVLIKEDVGGAVKAIQSFQKDTKKSETKGALFEGRLLSDSEIKEIASLPSKEVLMAKI

AGALNGVATKIAISINEVPSGLARSLKQHSEKSES

>gi|123199402|gb|ABM71043.1| 50S ribosomal protein L22 [Prochlorococcus marinus str. AS9601]

MTKTPETTKKAIAHGNYVRGSASKVRRVLDQIRGRSYRDALIMLEFMPYRSTDPITKVLRSAVANAEHNL

GMDPSTLVISSAWANSGPVMKRYRPRAQGRAFSIKKQTCHISISVESAPTETNAEVQN

>gi|123199401|gb|ABM71042.1| 30S ribosomal protein S3 [Prochlorococcus marinus str. AS9601]

MGHKIHPSGLRLGITQEHRSKWFATSKTYPILLQEDFKIRTFIQKKYGAAGISDVLIARKADQLELELKT

ARPGVIVGRQGSGIEELRSGIQKTIGDRTRQVRINVVEVERVDADAFLLAEYIAQQLEKRVAFRRTIRMA

LQRAQRAGVLGLKIQVGGRLNGAEIARTEWTREGRVPLHTLRAEIDYATREANTTYGVLGIKVWVFKGEV

LPKEEQTIPVGASPKRKASRRPQQFEDRSNENS

>gi|123199395|gb|ABM71036.1| 50S ribosomal protein L5 [Prochlorococcus marinus str. AS9601]

MTLKNRYKESIRPKLLKDLGLKNIHQVPKVVKVNVNRGLGEAASNSKALEASLNEMATITGQKALVTRAK

KAIAGFKIREGMPIGCTVTLRGDRMYSFLERFINLALPRIRDFRGVNPKSFDGRGNYTVGVKEQLIFPEI

SFDKIDSIRGMDITIVTSAKSDQEGKALLQELGMPFSKN

>gi|123198107|gb|ABM69748.1| 30S ribosomal protein S4 [Prochlorococcus marinus str. AS9601]

MSRYRGPRLRVTRRLGELPGLTRKASKKSNPPGQHGQARRKRSEYAIRLEEKQKLRFNYGVSEKQLVRYV

KKARAQEGSTGTNLLRLLENRLDNVCFRLGFGGTIPGSRQLVNHGHVTVNGKVLDIAGYQCKSGDVIGIK

ENKASKKLVEGNIEFPGLANVPPHLDLDKPKLTGKINGKCDREWVALEINELLVVEYYSRKV

>gi|123197868|gb|ABM69509.1| 50S ribosomal protein L1 [Prochlorococcus marinus str. AS9601]

MKKLSKRMAALSTKIEDRIYAPLEALSIIKENANAKFDETIEAHIRLGIDPKYTDQQLRTTVVLPHGTGQ

SIKIAVITSGENVSKAKAAGADLFGEEDLVESINKGNMEFDLLIATPDMMPKVAKLGRVLGPRGLMPNPK

AGTVTNDIANAIKEFKAGKLEFRADKAGIVHVRFGKASFTKEALFDNLKTLQESIDKNKPSGAKGKYWKT

FYVTSTMGPSVQLDINAVQDYQPEG

>gi|123197866|gb|ABM69507.1| 50S ribosomal protein L7/L12 [Prochlorococcus marinus str. AS9601]

MSAKTEEILESLKSLSLLEASELVKQIEEAFGVSAAASAGVVMAAPGAAGGDADGGAAEEKTEFDVVLES

FDAAAKIKVLKVVRNATGLGLGDAKALVESAPKTVKEGIAKADAESLKKEIEEAGGKVTLK

>gi|123199558|gb|ABM71199.1| 30S ribosomal protein S6 [Prochlorococcus marinus str. AS9601]

MVSNEQTLITSIDEPKTLSETMTDQQSYYETMYILRPDIAEDEVTNHIDKYNKLLEEFGGTILDSQMRGK

RRLAYQIAKHREGIYVQLSHQGDGQHIFKIEKAMRLSEDVIRYMTVKQEGPLPTPRPSNKSTSQSENNDN

PDAKVESKEKQPVASADSSTSSKDDAGTKENAES

>gi|123199513|gb|ABM71154.1| 50S ribosomal protein L20 [Prochlorococcus marinus str. AS9601]

MARVKRGNIARKRRNKILNLAKGFRGGNKNLFRTANQRVMKALCNAYRDRRRRKRDFRRLWISRINASAR

INGTNYSKLINGMKNADIIINRKMLAQLAITDPKCFEKIVSSVSQ

>gi|123199512|gb|ABM71153.1| 50S ribosomal protein L35 [Prochlorococcus marinus str. AS9601]

MSKLKTRKSAAKRFKATATGKFMRRRAFHNHLLDHKSSKLKRHLSTKAVVDERDADNVRLMIPYA

>gi|123199407|gb|ABM71048.1| 50S ribosomal protein L3 [Prochlorococcus marinus str. AS9601]

MSIGILGKKLGMSQLFDDKGNSVPVTLIEAGPCRVTQLKTTALDGYTAVQIGYGLSKEKHLSKPEKGHLL

KSGEELLKHLKEYRVEETSSYEIGKQITVKNFEVGQKVDISGKSMGRGFAGYQKRHGFSRGPMSHGSKNH

RAPGSTGAGTTPGRIYPGKRMAGRYGGKQITTKGLLVLKIDDQKNLLVVKGSVPGKPGSIINIKPNNVVG

KKGGEKS

>gi|123199406|gb|ABM71047.1| 50S ribosomal protein L4 [Prochlorococcus marinus str. AS9601]

MTTLETLKWDGKKSGKVSLDLAVAKETSSADLIHRAVLRQLANKRQGTASTLTRSEVRGGGRKPYKQKGT

GRARQGSIRTPLRPGGGIIFGPKPRSYNLDMNRKERRLALRTALMSRVSDMKAVEDFGSTLKQPKTSDII

NGLARLGIQKTEKVLVILDSPSDIIKKSINNIEKVKLIAADQLNVFDILNANKLVIGQSAIDKIQEVYAS

>gi|123199405|gb|ABM71046.1| 50S ribosomal protein L23 [Prochlorococcus marinus str. AS9601]

MSKLFDSRLADVIRKPVITEKATNALDLNQYTFEVDHRAAKPQIKAAVEALFSVKVIGVNTMNPPRRTRR

VGKFSGKRSQVKKAIVRLAEGDKIQLFPES

>gi|123199404|gb|ABM71045.1| 50S ribosomal protein L2 [Prochlorococcus marinus str. AS9601]

MAIRKFKPYTPGTRQRVVTDFSEITSAKPERSLIVSKHRVKGRNNRGVITCRHRGGGHKRQYRLVDFRRD

KRNINAKVAAIHYDPHRNARLALLFYEDGEKRYIIAPAGVKVGQNVISGESVPIEDGNAMPLSVMPLGSS

VHCVELYAGRGAQMVRSAGASAQVMAKEGDYVALKLPSTEVRLVRKECYATLGEVGNSEIRNTSLGKAGR

RRWLGRRPQVRGSVMNPCDHPHGGGEGKAPIGRAGPVTPWGKPALGLKTRKKNKPSNKLVVRRRRRVSKR

SRGGRDS

>gi|123199403|gb|ABM71044.1| 30S Ribosomal protein S19 [Prochlorococcus marinus str. AS9601]

MGRSLKKGPFIADSLLKKVEKQNTDNDKSVIKTWSRSSTILPLMIGHTIAVHNGKTHIPVFITEQMIGHK

LGEFAPTRTYRGHIRDKKGAKS

>gi|123199398|gb|ABM71039.1| 30S Ribosomal protein S17 [Prochlorococcus marinus str. AS9601]

MALKERIGTVVSDKMDKTVVVAVINRYPHPTYKKIVSRTTRYKAHDPENTCVLGDRVKIRETRPLSAHKR

WAIEEILNKTSQAKEVKK

>gi|123199397|gb|ABM71038.1| 50S Ribosomal protein L14 [Prochlorococcus marinus str. AS9601]

MIQQETYLTVADNSGAKRLQCIRVLGSNRRYAHVGDVIVATVKDALPNMGVKKSEVVKAVIVRTKATLRR

NTGNSIRFDDNAAVLINEDKNPKGTRVFGPVARELRDKNYTKIVSLAPEVI

>gi|123199396|gb|ABM71037.1| 50S ribosomal protein L24 [Prochlorococcus marinus str. AS9601]

MLDSLKQKKNFQRIKMRIKTGDLVKVINGKDKGKTGEVLKTIPLENRVVVKGINLRTKHVKPTQEGETGR

ILTEEASLHASNVMFFSKDKNLTSKIEYFIDKEGVKKRRLKKTGEVID

>gi|123199394|gb|ABM71035.1| 30S ribosomal protein S8 [Prochlorococcus marinus str. AS9601]

MSNHDPISDMLTRIRNASQKKHTTTTIPGSKMSLSIAKVLQKEGFISDINEEGEGYKSQIILGLKYSGKN

KFPTIRSMQRVSKPGLRIYKNTRGLPKVLGGLGVAIISTSKGVMSDRDARKQGIGGEVLCYVY

>gi|123199392|gb|ABM71033.1| 50S ribosomal protein L18 [Prochlorococcus marinus str. AS9601]

MTKLSRKLQTQKRHRRLRRYLIGDATRPRLSVYRSNNHIYAQVIDDSAQTTICSASTVDKELKEKSEKLP

SDCNSSSIVGKLLAKRAIKKGIKQVIFDRGGNLYHGRVKALADAAREAGLEF

>gi|123199390|gb|ABM71031.1| 50S ribosomal protein L15 [Prochlorococcus marinus str. AS9601]

MTSTLNTLKSNSGSRKKKLRKGRGIAAGQGASCGFGMRGQKSRSGRPTRPGFEGGQMPLYRRVPKLKHFE

IINQKNFSIINLEKLNDFKDNDTVNIDSLVKKGLIFKPKFPLKILGNGKINVKLKVQAHSFTKVAKQKIE

DAGGSCELINNK

>gi|123199387|gb|ABM71028.1| 30S ribosomal protein S13 [Prochlorococcus marinus str. AS9601]

MARIAGIDIPREKRVEIALTYVYGIGLTRSKLILANTGVNPDTRVKDLSDGDVQKLRGATEEFTLEGDLR

RKEGMALKRLQDIGCVRGRRHRMSLPVRGQRTRTNARTRRGSRKTVAGRKK

>gi|123199386|gb|ABM71027.1| 30S ribosomal protein S11 [Prochlorococcus marinus str. AS9601]

MAATVKKTGSKKSKRNVPNGVVHIQSTFNNTIVSITDTSGHVISWSSAGASGFKGARKGTPFAAQTAAEA

AARRALDQGMRQIEVLVRGPGAGRETAIRALQVAGLEITLIRDVTPLPHNGCRRPKRRRV

>gi|123199384|gb|ABM71025.1| 50S ribosomal protein L17 [Prochlorococcus marinus str. AS9601]

MRHQLRIPLLSKPADQRKALLRGLTTQLIREGRVTTTKARAKALRNEAERMISLAKEGSLASRRRAIGYI

YDKKLVHSLFEKAKERYGDREGGYTRIVRTVSRKGDNAQMAIIELV

>gi|123199382|gb|ABM71023.1| 50S ribosomal protein L13 [Prochlorococcus marinus str. AS9601]

MNKTITPSLETIERNWFLVDAKDKTLGRLATEIATVLRGKNKPTYTPHLDTGDFVIVVNAEKVEVTGKKA

SQKLYRRHSGRPGGMKIEKFESLQERIPERIIEQAVKGMLPHNSLGRQQFKKLKVYKGADHPHAAQNPVL

LNS

>gi|123199381|gb|ABM71022.1| 30S ribosomal protein S9 [Prochlorococcus marinus str. AS9601]

MNSQIKNKAVYWGTGRRKTSVARVRLIPGNGLIKINGRSGDDYLNFNPLHLNSIKAPLQTLGLENSYDIL

VNVFGGGLTGQADAIKQGAARALCELSPDNRKPLKTEGHLSRDPRAKERRKYGLKKARKAPQFSKR

>gi|123199380|gb|ABM71021.1| 50S ribosomal protein L31 [Prochlorococcus marinus str. AS9601]

MPKSEIHPKWYPDAKVICNGEVVMTTGSTQPELHVDVWSGNHPFFTGTQKILDTEGRVDRFMKKYGMGSA

NSATSKEQKEEKDSKK

>gi|123199356|gb|ABM70997.1| 30S ribosomal protein S12 [Prochlorococcus marinus str. AS9601]

MPTISQLVGSERKRLTKKTKSPALKACPERRGVCTRVYTSTPKKPNSALRKVARVRLTSGFEVTAYIPGI

GHNLQEHSVVLLRGGRVKDLPGVRYHIIRGTLDTAGVKDRRQSRSKYGAKAPKD

>gi|123199355|gb|ABM70996.1| 30S ribosomal protein S7 [Prochlorococcus marinus str. AS9601]

MSRRNAAVKRPVLPDPQFNSRLASMMISRLMKHGKKSTAQRILSDAFSLISERTGGNAVELFETAVKNAT

PLVEVRARRVGGATYQVPMEVRQERGTAMALRWLVTFSRARNGKSMSQKLAGELMDAANETGSSVKKRED

THKMAEANKAFAHYRY

>gi|123199352|gb|ABM70993.1| 30S ribosomal protein S10 [Prochlorococcus marinus str. AS9601]

MTASIAQQKIRIRLKAFDRRMLDLSCDKIIQTADTTSASAIGPIPLPTKRKIYCVLRSPHVDKDSREHFE

TRTHRRIIDIYSPSAKTIDALMKLDLPSGVDIEVKL

>gi|123199333|gb|ABM70974.1| 30s Ribosomal protein S20 [Prochlorococcus marinus str. AS9601]

MANNKSAKKRIQIAERNRLMNKSYKSTVRTLTKKTLENCEKYKKNPNEDNKNLVKTSLNKAFSLIDKAVK

KNVLHKNNGANRKSKINNFVKTTLTTK

>gi|123199196|gb|ABM70837.1| putative methyltransferase for Ribosomal protein L11 [Prochlorococcus marinus str. AS9601]

MTIKDWYKLTFLIESDSEEIIIWKLNELGIFSFSFEYLIKNKNKKEVNIWLPVADWSESSRFGVEKIITK

LLNINAPTNQFFDWSIIKEEDWLTSWKKYWAPELVGNHFLILPCWINLNEKFKDKKIIKIDPGAAFGTGS

HPSTYLCLEKMDNILFSDKKILDIGSGSGILSVAARLLGAKEVCAVDNDYLAINATKSNFQLNFGNLNNL

NTYLGSFNEVILKNQLKQFDFVLCNILAEVIKEMIPNIYKCLRNNGEVIFSGILNSQKDEIIKILIQNDL

KLLDVSTRKDWACISAQKASDPT

>gi|123199188|gb|ABM70829.1| 50S ribosomal protein L27 [Prochlorococcus marinus str. AS9601]

MAHKKGTGSTRNGRDSNSKRLGVKAYGGEKVTAGSILIRQRGTSFLPGNNVGIGKDDTLFALKEGTVSFE

SIKRNLKNRKRVNIVI

>gi|123199187|gb|ABM70828.1| 50S ribosomal protein L21 [Prochlorococcus marinus str. AS9601]

MIAYLAKSNYKFEPDSVFKKMTNSKNSSSNSLKSNELFAIAETSGQQFWFEVNRYYDIDRLNAKEKDKIT

LEKVLLLKDKDSITVGKPYVKDAKIELEVVSHKRDKKILVYKMRPKKKTRRKMGHRQELTRVMVKSITIG

KDTPKSSSKKETIKKETKPKSEKSTN

>gi|123199126|gb|ABM70767.1| 30S Ribosomal protein S16 [Prochlorococcus marinus str. AS9601]

MIKLRLKRFGKKKEASFRIVACNSTSRRDGRPLQELGFYNPRTKETRLDTEALRTRLTQGAQPTDVVRTL

LEKGGLLEKTERPSIAIGKAKLEKEKLAKAKTKDEENDNSKVESEGNEAES

>gi|123199026|gb|ABM70667.1| 30S Ribosomal protein S14 [Prochlorococcus marinus str. AS9601]

MAKKSMIAREVKRKKLVKKYSAKRKALLDEFNAAKDPMERLEIHRKIQGLPRNSAPNRVRNRCWATGKPR

GVYRDFGLCRNQLRQRAHNGELPGVVKSSW

>gi|123198636|gb|ABM70277.1| 30S Ribosomal protein S18 [Prochlorococcus marinus str. AS9601]

MPNSIFKKQLSPIKPGDPIDYKDVELLKKFITERGKILPRRMTGLTSKQQRDLTLAVKRARIVALLPFVN

PEG

>gi|123198602|gb|ABM70243.1| 50S ribosomal protein L28 [Prochlorococcus marinus str. AS9601]

MSRACELTGAKANNGMAVSHSHIRTKKLQQVNLQKRRLWWEEGKKWVNIKISTKALKSIQKVGLDKFAKS

NGVDLKKF

>gi|123198560|gb|ABM70201.1| 30S Ribosomal protein S15 [Prochlorococcus marinus str. AS9601]

MSLDTAEKQKLIENHQVHPTDTGSVEVQVAMLSKRISKLSDHLQGNIHDFASRQGLLKMIGKRKRLLSYL

KDKNVQKYQELVKKIGIRG

>gi|123198459|gb|ABM70100.1| 30S ribosomal protein S2 [Prochlorococcus marinus str. AS9601]

MAVVSLSEMMEAGAHFGHQTRRWNPKMSKYIYCARNGVHIIDLVKTALCMNNAYKWTRNAAKSGKRFLFV

GTKKQASDVVAQEATRCGAAYVNQRWLGGMLTNWTTMKARIERLKDLERMESSGSIAMRPKKEAAVLRRE

LERLQKYLGGLKGMRRLPDVVVLVDQRRESNAVLEARKLDISLVSMLDTNCDPDLCEVPIPCNDDAVRSV

QLILGRLADAINEGRKGSNAERKN

>gi|123198177|gb|ABM69818.1| Ribosomal protein L19 [Prochlorococcus marinus str. AS9601]

MTKMAKEKQEKDLETGIKAEASVDVAVEQKEKNTVSETKQTLSASNLIREFENEQLKKELPEIYVGDTVK

VGVKITEGNKERVQPYEGVVIAKRHGGINQTITVRRIFQGIGVERVFMLHSPQVASLKVERRGKVRRAKL

FYLRDRVGKATRVKQRFDR

>gi|123199400|gb|ABM71041.1| 50S ribosomal protein L16 [Prochlorococcus marinus str. AS9601]

MLSPKRTKFRKQHRGRMRGVASKGNTIAFGQFALQAQDCGWVTARQIEASRRAMTRYIKRGGQIWIRIFP

DKPVTMRPAETRMGSGKGNPEFWVAVVKPGRILFEMGGEDITEETAKEAMRLAQYKLPVKTKFISIDKNL

ENSSQENTKDSKKSQEEVKQ

>gi|123199399|gb|ABM71040.1| 50S ribosomal protein L29 [Prochlorococcus marinus str. AS9601]

MKNSESLKEFKKLNSEQITEKIDQLRKDLFDLRFKQATRQLNETHKFKIIKKQVAQLLTLSKNQSASQTT

PD

>gi|123199019|gb|ABM70660.1| 50S ribosomal protein L34 [Prochlorococcus marinus str. AS9601]

MTKRTFGGTSRKRKRVSGFRVRMRSHTGRRVIKSRRQKGRERIAV

>gi|123198711|gb|ABM70352.1| 30S Ribosomal protein S21 [Prochlorococcus marinus str. AS9601]

MTQVTVGENEGIESALRRFKRQVSKSGIFADLKRLRHHETPIEKYKRKLQQRRKARRR

>gi|123198635|gb|ABM70276.1| 50S Ribosomal protein L33 [Prochlorococcus marinus str. AS9601]

MYNRKLYNKVLIKMAKKGTRVVVTLECTEARTSTDPKRSNGVSRYTTEKNRRNTTERLELKKFNPHLNRM

TIHKEIK

>gi|123198659|gb|ABM70300.1| 50S ribosomal protein L32 [Prochlorococcus marinus str. AS9601]

MAVPKKKKSKSKRNQRHAVWKGKAAIAAQKAISLGKSVLTGKAQGFVYPIEEEEEE

>gi|123199338|gb|ABM70979.1| N utilization substance protein A [Prochlorococcus marinus str. AS9601]

MALVILPGLNNLIEDISEEKKLPPNIVELALREALLKGYEKYRKTFYIGVNQDPFDEEYFSNFDVGLDLD

EEGYRILSSKIIVEEVESEDHQISLVEVKQVADDAQIGDTVVLDVTPEKEDFGRMAASTTKQVLAQKLRD

QQRKMIQEEFADLEDPVLTARVIRFERQSVIMGVSSGIGRPEVEAELPKRDQLPNDNYRANATFKVFLKE

VSEIARKGPQLFVSRANAGLVVYLFENEVPEIQEGTVKIVAVSREANPPSRAVGPRTKVAVDSVEEEVDP

VGACIGARGARIQQVVNELRGEKIDVIKWSSNPIQYILNSLSPAKVDQVRLVDPAGQHAHVLVPPDQLSL

AIGREGQNVRLAARLTGWKIDVKNSHEYDQEAEDAAVSELIIQREDEENLQREAELRLEAEQAERAAEDA

RLRELYPLPEDEEEYGEEQYEGVEFTDNDPLETVQDTETSAKEEKKR

>gi|123198263|gb|ABM69904.1| possible acetyltransferase [Prochlorococcus marinus str. AS9601]

MIFRNQGSLIKKSNSISKDELIDLYGLNSYEFTQTTKEEIFVCSKNKDLDLIELDQLLQTVGWSRRPIRR

VKRALDYSILVVGLWRHDDKFPRLVGFARCTGDGILEATVWDVAINPVYQGLGLGKEIMRYVLKELKNIG

ISKVTLFADAEVVSFYKRQGWILEPRGSKCAFWYAN

>gi|123197665|gb|ABM69306.1| Predicted GTPases [Prochlorococcus marinus str. AS9601]

MKTNSKYLGLVTKKFNDYFLVDLKNQENSLNSEKFLCKVKKSINFRDQLIYVGDEVAIEKIDLKGKRAVI

TSLKKRKNLLVRPSVANISNIYVTFSVEEPELNLSQVNRFLISAESIGVEVSLVLTKCDLISDKRRSYLL

DKFEKWGYQVITLNLQNSDCFKNLLADLKQKECSIFMGPSGVGKTTLLNMIIPGLENSTAPVSNKIKRGK

NTTRNVELFSISNQSYIVDTPGFNMQTLEIDIKLLPNLYSEIHKQIVEEGIKCKFRNCLHLKDEGCNLNK

SFERYSFYKEMIESSKNHYYQNQED

>gi|123197765|gb|ABM69406.1| possible Fe-S oxidoreductase [Prochlorococcus marinus str. AS9601]

MKQNSLNVKEKKLSKVAFSHVGCEKNLVDTEHMQGLLDKEGYEVDSNINDANVVVVNTCSFIETAREESI

RKILEYTNQGKEVIVAGCMAQHFKDELIREIPEIKGLVGTGDYQKIAKVLDRVEQGEIVNEVSKIPEFIA

DEEMPRFVDKNKFVAYLRIAEGCNYNCAFCIIPKLRGPQRSRTIESIVSEAKSLAKKGIQEIILISQITT

NYGQDIYGKPSLAKLLNELSKVSIPWIRIHYAYPTGLTDEVIRAFKDSKNIVPYFDLPLQHSHPDVLKSM

NRPWQASLNESILEKIREEIPSAVLRTSLIVGFPGEKKEHFEHLLQFLDRHKFDHVGVFIFSPEEGTTAF

HLPNKVSLEVAEARKDNVISVQQNISKDKNQTYVGSKMKILVEKISDNNELIGRSYNFAPEIDGTVILSV

KDKIDLKNYIGKFVEANITFADEYDLYGEIIKIL

>gi|189045477|sp|A2BTC1.1|RS5_PROMS RecName: Full=30S ribosomal protein S5

MTDTPTKQEIQSKNDNVPAATPVEQKKNNRNDRKRNRRGDSKNLERDSDWQERVVQIRRVSKTVKGGKKM

SFRAIVVVGNEKGQVGVGVGKAGDVIGAVRKGVSDGKKNLVRVPLTPNNSIPTLSLGSDGAANVLIRPAA

PGTGVIAGGSIRTVLELAGIKNVLAKRLGSKTPLNNARAAMVALSQLRTHKSVSRERGISLEQLYS

>gi|166230913|sp|A2BNZ9.1|RL11_PROMS RecName: Full=50S ribosomal protein L11

MAKKIVAVIKLALQAGKANPAPPVGPALGQHGVNIMAFCKEYNARTQDKAGFVIPVEISVFEDRSFTFIT

KTPPASVLITKAAGIEKGSGESAKGSVGNISKAQLEEIAKTKLPDLNCSSVESAMKVIEGTARNMGVSIT

D

>gi|166223844|sp|A2BTQ4.1|RL9_PROMS RecName: Full=50S ribosomal protein L9

MAKRVQVALTESIASLGKEGDLVDVAPGYARNFLLPYGKAMNVTPAVLKQIERKKEKEKIAAGKLKQEAL

DFQTALSTIGRFTIKKQVGEDGVLFGTVTNGDVAEAIEAATKKEIDRRNITVPDIHNLGAFTAKIKLHPE

VNAEVNIEVTS

>gi|166230050|sp|A2BNZ7.1|RL10_PROMS RecName: Full=50S ribosomal protein L10

MGRTLENKQKIVTEIKSLLDDSEMAVVLDYKGLTIKEMSDLRSRLQTTNGICKVTKNSLMRKAIDGDSNW

NDLESLLTGTNAFVLIKEDVGGAVKAIQSFQKDTKKSETKGALFEGRLLSDSEIKEIASLPSKEVLMAKI

AGALNGVATKIAISINEVPSGLARSLKQHSEKSES

>gi|215274855|sp|A2BTD2.1|RL22_PROMS RecName: Full=50S ribosomal protein L22

MTKTPETTKKAIAHGNYVRGSASKVRRVLDQIRGRSYRDALIMLEFMPYRSTDPITKVLRSAVANAEHNL

GMDPSTLVISSAWANSGPVMKRYRPRAQGRAFSIKKQTCHISISVESAPTETNAEVQN

>gi|166222195|sp|A2BNZ6.1|RL7_PROMS RecName: Full=50S ribosomal protein L7/L12

MSAKTEEILESLKSLSLLEASELVKQIEEAFGVSAAASAGVVMAAPGAAGGDADGGAAEEKTEFDVVLES

FDAAAKIKVLKVVRNATGLGLGDAKALVESAPKTVKEGIAKADAESLKKEIEEAGGKVTLK

>gi|166216367|sp|A2BTC5.1|RL5_PROMS RecName: Full=50S ribosomal protein L5

MTLKNRYKESIRPKLLKDLGLKNIHQVPKVVKVNVNRGLGEAASNSKALEASLNEMATITGQKALVTRAK

KAIAGFKIREGMPIGCTVTLRGDRMYSFLERFINLALPRIRDFRGVNPKSFDGRGNYTVGVKEQLIFPEI

SFDKIDSIRGMDITIVTSAKSDQEGKALLQELGMPFSKN

>gi|160166296|sp|A2BNZ8.1|RL1_PROMS RecName: Full=50S ribosomal protein L1

MKKLSKRMAALSTKIEDRIYAPLEALSIIKENANAKFDETIEAHIRLGIDPKYTDQQLRTTVVLPHGTGQ

SIKIAVITSGENVSKAKAAGADLFGEEDLVESINKGNMEFDLLIATPDMMPKVAKLGRVLGPRGLMPNPK

AGTVTNDIANAIKEFKAGKLEFRADKAGIVHVRFGKASFTKEALFDNLKTLQESIDKNKPSGAKGKYWKT

FYVTSTMGPSVQLDINAVQDYQPEG

>gi|152112248|sp|A2BPN7.1|RS4_PROMS RecName: Full=30S ribosomal protein S4

MSRYRGPRLRVTRRLGELPGLTRKASKKSNPPGQHGQARRKRSEYAIRLEEKQKLRFNYGVSEKQLVRYV

KKARAQEGSTGTNLLRLLENRLDNVCFRLGFGGTIPGSRQLVNHGHVTVNGKVLDIAGYQCKSGDVIGIK

ENKASKKLVEGNIEFPGLANVPPHLDLDKPKLTGKINGKCDREWVALEINELLVVEYYSRKV

>gi|152060895|sp|A2BTD1.1|RS3_PROMS RecName: Full=30S ribosomal protein S3

MGHKIHPSGLRLGITQEHRSKWFATSKTYPILLQEDFKIRTFIQKKYGAAGISDVLIARKADQLELELKT

ARPGVIVGRQGSGIEELRSGIQKTIGDRTRQVRINVVEVERVDADAFLLAEYIAQQLEKRVAFRRTIRMA

LQRAQRAGVLGLKIQVGGRLNGAEIARTEWTREGRVPLHTLRAEIDYATREANTTYGVLGIKVWVFKGEV

LPKEEQTIPVGASPKRKASRRPQQFEDRSNENS

>gi|166199809|sp|A2BS99.1|RL34_PROMS RecName: Full=50S ribosomal protein L34

MTKRTFGGTSRKRKRVSGFRVRMRSHTGRRVIKSRRQKGRERIAV

>gi|166231013|sp|A2BTB2.1|RL13_PROMS RecName: Full=50S ribosomal protein L13

MNKTITPSLETIERNWFLVDAKDKTLGRLATEIATVLRGKNKPTYTPHLDTGDFVIVVNAEKVEVTGKKA

SQKLYRRHSGRPGGMKIEKFESLQERIPERIIEQAVKGMLPHNSLGRQQFKKLKVYKGADHPHAAQNPVL

LNS

>gi|226731536|sp|A2BSA6.1|RS14_PROMS RecName: Full=30S ribosomal protein S14

MAKKSMIAREVKRKKLVKKYSAKRKALLDEFNAAKDPMERLEIHRKIQGLPRNSAPNRVRNRCWATGKPR

GVYRDFGLCRNQLRQRAHNGELPGVVKSSW

>gi|226708155|sp|A2BTB0.1|RL31_PROMS RecName: Full=50S ribosomal protein L31

MPKSEIHPKWYPDAKVICNGEVVMTTGSTQPELHVDVWSGNHPFFTGTQKILDTEGRVDRFMKKYGMGSA

NSATSKEQKEEKDSKK

>gi|218547108|sp|A2BTC6.1|RL24_PROMS RecName: Full=50S ribosomal protein L24

MLDSLKQKKNFQRIKMRIKTGDLVKVINGKDKGKTGEVLKTIPLENRVVVKGINLRTKHVKPTQEGETGR

ILTEEASLHASNVMFFSKDKNLTSKIEYFIDKEGVKKRRLKKTGEVID

>gi|166987300|sp|A2BTD5.1|RL23_PROMS RecName: Full=50S ribosomal protein L23

MSKLFDSRLADVIRKPVITEKATNALDLNQYTFEVDHRAAKPQIKAAVEALFSVKVIGVNTMNPPRRTRR

VGKFSGKRSQVKKAIVRLAEGDKIQLFPES

>gi|166234500|sp|A2BTC0.1|RL15_PROMS RecName: Full=50S ribosomal protein L15

MTSTLNTLKSNSGSRKKKLRKGRGIAAGQGASCGFGMRGQKSRSGRPTRPGFEGGQMPLYRRVPKLKHFE

IINQKNFSIINLEKLNDFKDNDTVNIDSLVKKGLIFKPKFPLKILGNGKINVKLKVQAHSFTKVAKQKIE

DAGGSCELINNK

>gi|166234345|sp|A2BQZ0.1|RS15_PROMS RecName: Full=30S ribosomal protein S15

MSLDTAEKQKLIENHQVHPTDTGSVEVQVAMLSKRISKLSDHLQGNIHDFASRQGLLKMIGKRKRLLSYL

KDKNVQKYQELVKKIGIRG

>gi|166233179|sp|A2BTD7.1|RL3_PROMS RecName: Full=50S ribosomal protein L3

MSIGILGKKLGMSQLFDDKGNSVPVTLIEAGPCRVTQLKTTALDGYTAVQIGYGLSKEKHLSKPEKGHLL

KSGEELLKHLKEYRVEETSSYEIGKQITVKNFEVGQKVDISGKSMGRGFAGYQKRHGFSRGPMSHGSKNH

RAPGSTGAGTTPGRIYPGKRMAGRYGGKQITTKGLLVLKIDDQKNLLVVKGSVPGKPGSIINIKPNNVVG

KKGGEKS

>gi|166232685|sp|A2BTC7.1|RL14_PROMS RecName: Full=50S ribosomal protein L14

MIQQETYLTVADNSGAKRLQCIRVLGSNRRYAHVGDVIVATVKDALPNMGVKKSEVVKAVIVRTKATLRR

NTGNSIRFDDNAAVLINEDKNPKGTRVFGPVARELRDKNYTKIVSLAPEVI

>gi|166231312|sp|A2BT82.1|RS10_PROMS RecName: Full=30S ribosomal protein S10

MTASIAQQKIRIRLKAFDRRMLDLSCDKIIQTADTTSASAIGPIPLPTKRKIYCVLRSPHVDKDSREHFE

TRTHRRIIDIYSPSAKTIDALMKLDLPSGVDIEVKL

>gi|166229588|sp|A2BTB1.1|RS9_PROMS RecName: Full=30S ribosomal protein S9

MNSQIKNKAVYWGTGRRKTSVARVRLIPGNGLIKINGRSGDDYLNFNPLHLNSIKAPLQTLGLENSYDIL

VNVFGGGLTGQADAIKQGAARALCELSPDNRKPLKTEGHLSRDPRAKERRKYGLKKARKAPQFSKR

>gi|166228462|sp|A2BT85.1|RS7_PROMS RecName: Full=30S ribosomal protein S7

MSRRNAAVKRPVLPDPQFNSRLASMMISRLMKHGKKSTAQRILSDAFSLISERTGGNAVELFETAVKNAT

PLVEVRARRVGGATYQVPMEVRQERGTAMALRWLVTFSRARNGKSMSQKLAGELMDAANETGSSVKKRED

THKMAEANKAFAHYRY

>gi|166226602|sp|A2BR32.1|RL28_PROMS RecName: Full=50S ribosomal protein L28

MSRACELTGAKANNGMAVSHSHIRTKKLQQVNLQKRRLWWEEGKKWVNIKISTKALKSIQKVGLDKFAKS

NGVDLKKF

>gi|166225297|sp|A2BQN9.1|RS2_PROMS RecName: Full=30S ribosomal protein S2

MAVVSLSEMMEAGAHFGHQTRRWNPKMSKYIYCARNGVHIIDLVKTALCMNNAYKWTRNAAKSGKRFLFV

GTKKQASDVVAQEATRCGAAYVNQRWLGGMLTNWTTMKARIERLKDLERMESSGSIAMRPKKEAAVLRRE

LERLQKYLGGLKGMRRLPDVVVLVDQRRESNAVLEARKLDISLVSMLDTNCDPDLCEVPIPCNDDAVRSV

QLILGRLADAINEGRKGSNAERKN

>gi|166225033|sp|A2BSR8.1|RL27_PROMS RecName: Full=50S ribosomal protein L27

MAHKKGTGSTRNGRDSNSKRLGVKAYGGEKVTAGSILIRQRGTSFLPGNNVGIGKDDTLFALKEGTVSFE

SIKRNLKNRKRVNIVI

>gi|166224054|sp|A2BRE1.1|RS21_PROMS RecName: Full=30S ribosomal protein S21

MTQVTVGENEGIESALRRFKRQVSKSGIFADLKRLRHHETPIEKYKRKLQQRRKARRR

>gi|166223969|sp|A2BT63.1|RS20_PROMS RecName: Full=30S ribosomal protein S20

MANNKSAKKRIQIAERNRLMNKSYKSTVRTLTKKTLENCEKYKKNPNEDNKNLVKTSLNKAFSLIDKAVK

KNVLHKNNGANRKSKINNFVKTTLTTK

>gi|166219722|sp|A2BTC8.1|RS17_PROMS RecName: Full=30S ribosomal protein S17

MALKERIGTVVSDKMDKTVVVAVINRYPHPTYKKIVSRTTRYKAHDPENTCVLGDRVKIRETRPLSAHKR

WAIEEILNKTSQAKEVKK

>gi|166219680|sp|A2BTP3.1|RL20_PROMS RecName: Full=50S ribosomal protein L20

MARVKRGNIARKRRNKILNLAKGFRGGNKNLFRTANQRVMKALCNAYRDRRRRKRDFRRLWISRINASAR

INGTNYSKLINGMKNADIIINRKMLAQLAITDPKCFEKIVSSVSQ

>gi|166218836|sp|A2BTC2.1|RL18_PROMS RecName: Full=50S ribosomal protein L18

MTKLSRKLQTQKRHRRLRRYLIGDATRPRLSVYRSNNHIYAQVIDDSAQTTICSASTVDKELKEKSEKLP

SDCNSSSIVGKLLAKRAIKKGIKQVIFDRGGNLYHGRVKALADAAREAGLEF

>gi|166216204|sp|A2BTB4.1|RL17_PROMS RecName: Full=50S ribosomal protein L17

MRHQLRIPLLSKPADQRKALLRGLTTQLIREGRVTTTKARAKALRNEAERMISLAKEGSLASRRRAIGYI

YDKKLVHSLFEKAKERYGDREGGYTRIVRTVSRKGDNAQMAIIELV

>gi|166201335|sp|A2BSK6.1|RS16_PROMS RecName: Full=30S ribosomal protein S16

MIKLRLKRFGKKKEASFRIVACNSTSRRDGRPLQELGFYNPRTKETRLDTEALRTRLTQGAQPTDVVRTL

LEKGGLLEKTERPSIAIGKAKLEKEKLAKAKTKDEENDNSKVESEGNEAES

>gi|166199904|sp|A2BTD3.1|RS19_PROMS RecName: Full=30S ribosomal protein S19

MGRSLKKGPFIADSLLKKVEKQNTDNDKSVIKTWSRSSTILPLMIGHTIAVHNGKTHIPVFITEQMIGHK

LGEFAPTRTYRGHIRDKKGAKS

>gi|166199844|sp|A2BTD6.1|RL4_PROMS RecName: Full=50S ribosomal protein L4

MTTLETLKWDGKKSGKVSLDLAVAKETSSADLIHRAVLRQLANKRQGTASTLTRSEVRGGGRKPYKQKGT

GRARQGSIRTPLRPGGGIIFGPKPRSYNLDMNRKERRLALRTALMSRVSDMKAVEDFGSTLKQPKTSDII

NGLARLGIQKTEKVLVILDSPSDIIKKSINNIEKVKLIAADQLNVFDILNANKLVIGQSAIDKIQEVYAS

>gi|166199820|sp|A2BTP2.1|RL35_PROMS RecName: Full=50S ribosomal protein L35

MSKLKTRKSAAKRFKATATGKFMRRRAFHNHLLDHKSSKLKRHLSTKAVVDERDADNVRLMIPYA

>gi|160358608|sp|A2BTD4.1|RL2_PROMS RecName: Full=50S ribosomal protein L2

MAIRKFKPYTPGTRQRVVTDFSEITSAKPERSLIVSKHRVKGRNNRGVITCRHRGGGHKRQYRLVDFRRD

KRNINAKVAAIHYDPHRNARLALLFYEDGEKRYIIAPAGVKVGQNVISGESVPIEDGNAMPLSVMPLGSS

VHCVELYAGRGAQMVRSAGASAQVMAKEGDYVALKLPSTEVRLVRKECYATLGEVGNSEIRNTSLGKAGR

RRWLGRRPQVRGSVMNPCDHPHGGGEGKAPIGRAGPVTPWGKPALGLKTRKKNKPSNKLVVRRRRRVSKR

SRGGRDS

>gi|158706263|sp|A2BTB7.1|RS13_PROMS RecName: Full=30S ribosomal protein S13

MARIAGIDIPREKRVEIALTYVYGIGLTRSKLILANTGVNPDTRVKDLSDGDVQKLRGATEEFTLEGDLR

RKEGMALKRLQDIGCVRGRRHRMSLPVRGQRTRTNARTRRGSRKTVAGRKK

>gi|156637364|sp|A2BT86.1|RS12_PROMS RecName: Full=30S ribosomal protein S12

MPTISQLVGSERKRLTKKTKSPALKACPERRGVCTRVYTSTPKKPNSALRKVARVRLTSGFEVTAYIPGI

GHNLQEHSVVLLRGGRVKDLPGVRYHIIRGTLDTAGVKDRRQSRSKYGAKAPKD

>gi|152060809|sp|A2BTB6.1|RS11_PROMS RecName: Full=30S ribosomal protein S11

MAATVKKTGSKKSKRNVPNGVVHIQSTFNNTIVSITDTSGHVISWSSAGASGFKGARKGTPFAAQTAAEA

AARRALDQGMRQIEVLVRGPGAGRETAIRALQVAGLEITLIRDVTPLPHNGCRRPKRRRV

>gi|148841163|sp|A2BTC4.1|RS8_PROMS RecName: Full=30S ribosomal protein S8

MSNHDPISDMLTRIRNASQKKHTTTTIPGSKMSLSIAKVLQKEGFISDINEEGEGYKSQIILGLKYSGKN

KFPTIRSMQRVSKPGLRIYKNTRGLPKVLGGLGVAIISTSKGVMSDRDARKQGIGGEVLCYVY

>gi|218551742|sp|A2BR65.2|RL33_PROMS RecName: Full=50S ribosomal protein L33

MAKKGTRVVVTLECTEARTSTDPKRSNGVSRYTTEKNRRNTTERLELKKFNPHLNRMTIHKEIK

>gi|166220974|sp|A2BR66.1|RS18_PROMS RecName: Full=30S ribosomal protein S18

MPNSIFKKQLSPIKPGDPIDYKDVELLKKFITERGKILPRRMTGLTSKQQRDLTLAVKRARIVALLPFVN

PEG

>gi|166228243|sp|A2BTC9.1|RL29_PROMS RecName: Full=50S ribosomal protein L29

MKNSESLKEFKKLNSEQITEKIDQLRKDLFDLRFKQATRQLNETHKFKIIKKQVAQLLTLSKNQSASQTT

PD

>gi|166199705|sp|A2BTD0.1|RL16_PROMS RecName: Full=50S ribosomal protein L16

MLSPKRTKFRKQHRGRMRGVASKGNTIAFGQFALQAQDCGWVTARQIEASRRAMTRYIKRGGQIWIRIFP

DKPVTMRPAETRMGSGKGNPEFWVAVVKPGRILFEMGGEDITEETAKEAMRLAQYKLPVKTKFISIDKNL

ENSSQENTKDSKKSQEEVKQ

>gi|156630854|sp|A2BR89.1|RL32_PROMS RecName: Full=50S ribosomal protein L32

MAVPKKKKSKSKRNQRHAVWKGKAAIAAQKAISLGKSVLTGKAQGFVYPIEEEEEE

>gi|238066469|sp|A2BNP5.1|RIMO_PROMS RecName: Full=Ribosomal protein S12 methylthiotransferase RimO; Short=S12 MTTase; Short=S12 methylthiotransferase; AltName: Full=Ribosomal protein S12 (aspartate-C(3))-methylthiotransferase; AltName: Full=Ribosome maturation factor RimO

MKQNSLNVKEKKLSKVAFSHVGCEKNLVDTEHMQGLLDKEGYEVDSNINDANVVVVNTCSFIETAREESI

RKILEYTNQGKEVIVAGCMAQHFKDELIREIPEIKGLVGTGDYQKIAKVLDRVEQGEIVNEVSKIPEFIA

DEEMPRFVDKNKFVAYLRIAEGCNYNCAFCIIPKLRGPQRSRTIESIVSEAKSLAKKGIQEIILISQITT

NYGQDIYGKPSLAKLLNELSKVSIPWIRIHYAYPTGLTDEVIRAFKDSKNIVPYFDLPLQHSHPDVLKSM

NRPWQASLNESILEKIREEIPSAVLRTSLIVGFPGEKKEHFEHLLQFLDRHKFDHVGVFIFSPEEGTTAF

HLPNKVSLEVAEARKDNVISVQQNISKDKNQTYVGSKMKILVEKISDNNELIGRSYNFAPEIDGTVILSV

KDKIDLKNYIGKFVEANITFADEYDLYGEIIKIL

>gi|166223430|sp|A2BSS6.1|PRMA_PROMS RecName: Full=Ribosomal protein L11 methyltransferase; Short=L11 Mtase

MTIKDWYKLTFLIESDSEEIIIWKLNELGIFSFSFEYLIKNKNKKEVNIWLPVADWSESSRFGVEKIITK

LLNINAPTNQFFDWSIIKEEDWLTSWKKYWAPELVGNHFLILPCWINLNEKFKDKKIIKIDPGAAFGTGS

HPSTYLCLEKMDNILFSDKKILDIGSGSGILSVAARLLGAKEVCAVDNDYLAINATKSNFQLNFGNLNNL

NTYLGSFNEVILKNQLKQFDFVLCNILAEVIKEMIPNIYKCLRNNGEVIFSGILNSQKDEIIKILIQNDL

KLLDVSTRKDWACISAQKASDPT

>gi|166226465|sp|A2BTI7.1|RIMM_PROMS RecName: Full=Ribosome maturation factor RimM

MIIKNEWLIVGFITSCHGINGQLKVKSLSDFEERFLKPGMRWLQKESEHPSKIELISGFKQPGKEIFIVK

FKGINTRNHAEQLKKCKLLVKSDKLPKLKKEEFHLLELIDLEVKTLENDELKIIGKVINLENEKNNLLII

ELFKNQKKVLIPFVKEIVPLVDIKNNFLIINPPNGLLEL

>gi|33639819|emb|CAE18989.1| 30S ribosomal protein S1 homolog B, putative Nbp1 [Prochlorococcus marinus subsp. pastoris str. CCMP1986]

MKGVSDKDAQNNKKIKGDKNNLKKPLQVLHISKKDTISNKEEVFDGHQNSSKEVKTDISAVKPKFIEAPI

DEVKESYSNNINFENISYKELEKPLTFQDEDEDFIIERKVDEFDFDESAFLEALNENEPIGATGETIKGK

VIALESDGLYIDIGGKAPGFMPKKECGLGVITNFKEKFTIDLEMEVLVIKEQNADGMVTVSARALILRQS

WEKVASSAKNGELIQVTINGFNRGGLTCDVDGLRGFIPRSQLENGQDYQSLVSKNLKVAFLEVNPETRKL

VLSEKKALLVSKFADLKFGQLIEGEVLAIKPYGFFVDLGGASGLLHQSSITNGSIRNLREIFREGEVIKA

LITEIDLERGRIGLNTALLENTPGELIIDKEKVMIEASERSLKTKSLFDKKDLEK

>gi|33633812|emb|CAE18771.1| 30S ribosomal protein S1, homolog A [Prochlorococcus marinus subsp. pastoris str. CCMP1986]

MIENPSQIVKEISDEKEIENSTIEENTSDTPKEEDLSFDHKDIPSADSSSSRRNNDLDTAGFTQEEFASL

LGKYDYNFKPGDLVKGTVFALEPKGAMIDIGAKTAAFMPMQEVSINRVEGLSDVLQPSESREFFIMSEEN

EDGQLALSIRRIEYQRAWERVRQLQKEDATIYSEVFATNRGGALVRVEGLRGFIPGSHISARKIKEDLEG

EYLPLKFLEVDEERNRLVLSHRRALVEKKMNRLEVGEVVIGSVKGIKPYGAFIDIGGVSGLLHISEISHE

HIETPHNVLNVNDQMKVMIIDLDSERGRISLSTKALEPEPGDMLTDPQKVFNKAEEMAAKYKQMLLEQTD

ENEEQTVEIAESV

>gi|33634447|emb|CAE20132.1| 50S ribosomal protein L9 [Prochlorococcus marinus subsp. pastoris str. CCMP1986]

MAKRVKVVLTESVATLGRDGDVVEVAPGYARNFLLPFGKAANVTPSILKQIERKRAKEKIAAEKVKQEAI

DFKTALATIGRFTIKKQVGEDGVLFGTVTNGDVAEAIEAATKKDIDRRDITVPDIHNLGSFVAKIKLHQE

VSAEVNIEVTS

>gi|33634318|emb|CAE20003.1| 50S ribosomal protein L6 [Prochlorococcus marinus subsp. pastoris str. CCMP1986]

MSRIGKSPVQIPEKVSVDIKGLSITVKGPKGELKRLMPEGVNFDQKENQIVVTPATTKRYSRERHGLCRT

LISNMVQGVTEGYEKKLEIVGVGSRAQVKGKNLVVSAGYSHPVEMTPPDGITYKVESNTNVTVSGIDKEI

VGNEAAKIRSIRPPEPYKGKGIKYQDERIIRKAGKSGKK

>gi|33634316|emb|CAE20001.1| 30S ribosomal protein S5 [Prochlorococcus marinus subsp. pastoris str. CCMP1986]

MTDTPTKQENQSKTENPPSSNANEQRRGNRNNDRKRNRRGDSKNERDSEWQERVVQIRRVSKTVKGGKKM

SFRAIVVVGNEKGQVGVGVGKAGDVIGAVRKGVSDGKKHLVRVPLTPNNSIPTLSKGRDGAANVLIRPAA

PGTGVIAGGSIRTVLELAGIKNVLAKRLGSKTPLNNARAAMVALSQLRTHKSASRERGISLEQLYS

>gi|33633706|emb|CAE18663.1| 50S ribosomal protein L11 [Prochlorococcus marinus subsp. pastoris str. CCMP1986]

MAKKIVAVIKLALQAGKANPAPPVGPALGQHGVNIMAFCKEYNARTQDKAGFVIPVEISVFEDRSFTFIT

KTPPASVLITKAAGIEKGAGESSKGSVGNISKSQLEEIAKTKLPDLNCTSIESAMKVIEGTARNMGVSIT

E

>gi|33633704|emb|CAE18661.1| 50S ribosomal protein L10 [Prochlorococcus marinus subsp. pastoris str. CCMP1986]

MGRTIENKQKIVTEIKSLLDDSEMAVVLDYKGLTIKEMSDLRSRLQTNNGICKVTKNSLMRKAIDGNSNW

TDLESLLTGTNAFVLIKEDVGGAVKAIQSFQKETKKSETKGALFEGRLLSESEIKEIASLPSREVLMAKI

AGALNGVATKIAISINEVPSGIARSLKQHSEKSES

>gi|33640209|emb|CAE19744.1| 30S Ribosomal protein S16 [Prochlorococcus marinus subsp. pastoris str. CCMP1986]

MIKLRLKRFGKKKEASFRIVACNSTSRRDGRPLQELGFYNPRTKETRLDTEALRIRLTQGAQPTDVVRTL

LEKGGLLEKKVRPSIAIGKAKLEKEKIAKAKSKEAESDSKEAES

>gi|33639699|emb|CAE18869.1| 30S ribosomal protein S4 [Prochlorococcus marinus subsp. pastoris str. CCMP1986]

MSRYRGPRLRVTRRLGELPGLTRKASKKSNPPGQHGQARRKRSEYAIRLEEKQKLRFNYGVSERQLVRYV

KKARAQEGSTGTNLLRLLENRLDNVCFRLGFGGTIPGSRQLVNHGHVTINGKVLDIAGYQCKPGDVISIK

ENKASKKLVEGNIEFPGLANVPPHIELDKPKLTGKINGKCDREWVALEINELLVVEYYSRKV

>gi|33634327|emb|CAE20012.1| 50S ribosomal protein L22 [Prochlorococcus marinus subsp. pastoris str. CCMP1986]

MTKTPEMTKTAIAHGKYIRGSASKVRRVLDQIRGKSYRDALIMLEFMPYRSTDPITKVLRSAVANAEHNL

GMDPSSLVISSASADNGPVMKRFRPRAQGRAFSIKKQTCHISISVESAPNQTNTEAQN

>gi|33634326|emb|CAE20011.1| 30S ribosomal protein S3 [Prochlorococcus marinus subsp. pastoris str. CCMP1986]

MGNKINPTGFRLGITQEHRSKWFATSKTYPTLLQEDDKIRTFIQKKYSSAGISDVLIARKADQLELELKT

ARPGVIVGRQGSGIEELRSGIQKTIGDRTRQVRINVVEVERVDADAYLLAEYIAQQLEKRVAFRRTIRMA

LQRAQRAGVLGLKVQVGGRLNGAEIARTEWTREGRVPLHTLRAEVDYALREANTTYGVLGIKVWVFKGEV

LPKEEQTIPVGAIPRRKGSRKPQQFEDRSNENS

>gi|33634320|emb|CAE20005.1| 50S ribosomal protein L5 [Prochlorococcus marinus subsp. pastoris str. CCMP1986]

MTLKTRYKEAIRPKLLKDLGLKNIHQVPKVIKVNVNRGLGEAASNSKALEASLNEMATITGQKALVTRSK

KAIAGFKIREGMAIGCTVTLRGDRMYSFLERFINLALPRIRDFRGVNPKSFDGRGNYTLGVKEQLIFPEI

SFDKIDSIRGMDITIVTSASTDQEGKALLKELGMPFSN

>gi|33633705|emb|CAE18662.1| 50S ribosomal protein L1 [Prochlorococcus marinus subsp. pastoris str. CCMP1986]

MKKLSKRMKALSTKIEDRTYAPLEALGIVKENANAKFDETIEAHIRLGIDPKYTDQQLRTTVALPNGTGQ

SIKIAVITSGENVAKAKSAGADLFGEEDLVESINKGNMDFDLLIATPDMMPKVAKLGRVLGPRGLMPNPK

AGTVTGDIASAIKEFKAGKLEFRADKAGIVHVRFGKASFTENALFENLKTLQESIDKNKPSGAKGKYWRS

FYLTSTMGPSVQVDINALQDYQPES

>gi|33633703|emb|CAE18660.1| 50S ribosomal protein L7/L12 [Prochlorococcus marinus subsp. pastoris str. CCMP1986]

MTAKTEEILDSLKSLSLLEASELVKQIEEAFGVSAAASAGVVMAAPGAGGGDGDGGAAEEKTEFDVILES

FDAAAKIKVLKVVRNATGLGLGDAKALVESAPKTVKEGIAKADAETLKKEIEEAGGKVTLK

>gi|33640277|emb|CAE19812.1| putative methyltransferase for Ribosomal protein L11 [Prochlorococcus marinus subsp. pastoris str. CCMP1986]

MEIKNWYELTFEIETNLEEIIIWKLNELGISSYAFEILLNNKNNKKVIIWLPHLNWPESLRIKLVRNIKE

VLDKNNYQTNCFEWNLIEQEDWISSWKKYWGPEIVGDNLLILPCWLELPEEYKNKKVIKIDPGAAFGTGS

HPTTSLCLEELEKISLSSKKILDIGSGSGILSIAARSFGASKIYSIDNDYLAINSTESNFRLNFGNLDNL

KTYLGSFDGLVSKYTLKNFDFILCNILAEVIKGIIPDIRNCLKIDGEVILSGILNSQKDEIIKLLKASNL

RINDVSSKKDWVCITAQKIP

>gi|33640269|emb|CAE19804.1| 50S ribosomal protein L27 [Prochlorococcus marinus subsp. pastoris str. CCMP1986]

MAHKKGTGSTRNGRDSNSKRLGVKAYGGEKVSAGSIIIRQRGTSFLPGINVGKGKDDTLFALKEGTVSFD

SIKRNLRNRKRVNVVL

>gi|33640268|emb|CAE19803.1| 50S ribosomal protein L21 [Prochlorococcus marinus subsp. pastoris str. CCMP1986]

MTSSKKPSNSSAKNENLYAIAETSGQQFWFEVDKYYDIDRLNAKEKDKITIDKILLIKDKDNISLGQPYV

KNAKIELEVVSHKRDKKIIVYKMRPKKKTRRKMGHRQELTRVMVKSISITNSTPKTSSKTEVKKKSTSPK

ASNPEN

>gi|33640114|emb|CAE19649.1| 30S Ribosomal protein S14 [Prochlorococcus marinus subsp. pastoris str. CCMP1986]

MAKKSMIAREVKRKKLVKKYATKRKSLLDEFNAAKDPMERLEIHRKIQGLPRNSAPTRVRNRCWATGKPR

GVYRDFGLCRNQLRLRAHNGELPGVVKSSW

>gi|33640107|emb|CAE19642.1| 50S ribosomal protein L34 [Prochlorococcus marinus subsp. pastoris str. CCMP1986]

MTKRTFGGTSRKRKRVSGFRVRMRSHTGRRVIKSRRKRGRERIAV

>gi|33639764|emb|CAE18934.1| Ribosomal protein L19 [Prochlorococcus marinus subsp. pastoris str. CCMP1986]

MIIEHKMAKEKQETELEITNETDTTTELTVEKQGKELIAQTNLSSSNLIKEFEREQLKKQLPEIYVGDTV

KVGVKITEGNKERVQPYEGVVIAKRHGGLHQTITVRRIFQGIGVERVFMLHSPQVASLKVERRGKVRRAK

LFYLRDRVGKATRVKQRFDR

>gi|33634480|emb|CAE20165.1| 30S ribosomal protein S6 [Prochlorococcus marinus subsp. pastoris str. CCMP1986]

MTDQIYYETMYILRPDIAEDEVTNHIDKYNKLLEESGGKILDSQMRGKRRLAYQIAKHREGIYVQLSHQG

DGQHIFKIEKAMRLSEDVIRYLTVKQEGPLPTPRSSNKGYNQSEKKDIESIDSTNKSEFKEEANDKKTAT

SESTSSQGKESQKS

>gi|33634436|emb|CAE20121.1| 50S ribosomal protein L20 [Prochlorococcus marinus subsp. pastoris str. CCMP1986]

MARVKRGNIARKRRNKILNLAKGFRGGNKNLFRTANQRVMKALCNAYRDRRRRKRDFRRLWISRINASAR

INGTNYSRLINGMKNSEIIINRKMLAQLALSDPQCFEKIVSTVNN

>gi|33634435|emb|CAE20120.1| 50S ribosomal protein L35 [Prochlorococcus marinus subsp. pastoris str. CCMP1986]

MSKLKTRKSAAKRFKATATGKFTRRRAFHNHLLDHKSSKLKRHLKTKAVVDERDADNVKLMIPYA

>gi|33634332|emb|CAE20017.1| 50S ribosomal protein L3 [Prochlorococcus marinus subsp. pastoris str. CCMP1986]

MSIGILGKKLGMSQLFDKDGNAVPVTLIEAGPCRVTQLKTQPLDGYTAIQIGYGVSKDKHLSKPEKGHLL

KSGEILLKHLKEYRVEENSSYEIGKEITVTNFEVGQKVDISGKSMGRGFSGYQKRHGFSRGPMSHGSKNH

RAPGSTGAGTTPGRIYPGKRMAGRYGGKKITTKGLLVVKIDDQKNLLVVKGSVPGKPGSIVNIKPNNVVG

NKGGAKS

>gi|33634331|emb|CAE20016.1| 50S ribosomal protein L4 [Prochlorococcus marinus subsp. pastoris str. CCMP1986]

MTTLETLKWDGKKAGKVSIDLKVAKETSSADLIHRAVLRQLANKRQGTASTLTRSEVRGGGRKPYKQKGT

GRARQGSIRTPLRPGGGVIFGPKPRSYNLDMNRKERRLALRTALMSRISDFKTVEDFGSTLDQPKTSEII

NGLSRLGIEKTEKVLVILDNPSDVIKKSINNLEKVKLIAADQLNVFDILNANKLVIGQSAINKIQEVYAS

>gi|33634330|emb|CAE20015.1| 50S ribosomal protein L23 [Prochlorococcus marinus subsp. pastoris str. CCMP1986]

MSRLFDSRLADIIRKPVITEKATNALDLNQYTFEVDHRAAKPQIKAAIEALFDVKVIGINTMNPPRRTRR

VGKFSGKRSQIKKAIVRLAEGDKIQLFPES

>gi|33634329|emb|CAE20014.1| 50S ribosomal protein L2 [Prochlorococcus marinus subsp. pastoris str. CCMP1986]

MAIRKFKPYTPGTRQRVVTDFSEITGSKPERSLIVSKHRNKGRNNRGVITCRHRGGGHKRQYRLVDFRRD

KKNINAKVAAIHYDPHRNARLALLFYEDGEKRYIIAPAGIKVGQNVISGEGVPIEEGNAMPLSSMPLGSN

VHCVELYAGRGAQMVRSAGASAQLMAKEGEYVALKLPSTEVRLVRKECYATLGEVGNSEIRNTSLGKAGR

RRWLGRRPQVRGSVMNPCDHPHGGGEGKAPIGRAGPVTPWGKAALGLKTRKKNKPSNKLVVRRRRRISKR

SRGGRDS

>gi|33634328|emb|CAE20013.1| 30S Ribosomal protein S19 [Prochlorococcus marinus subsp. pastoris str. CCMP1986]

MGRSLKKGPFIADSLLKKVEKQNTDNDKSVIKTWSRSSTILPVMIGHTIAVHNGKAHIPVFITEQMIGHK

LGEFAPTRTYRGHLRDKKGAR

>gi|33634323|emb|CAE20008.1| 30S Ribosomal protein S17 [Prochlorococcus marinus subsp. pastoris str. CCMP1986]

MALKERIGTVVSDKMDKTVVVAVINRYPHPTYKKIVSKTTRYKAHDPENSCVLGDRVKIKETRPLSAHKR

WAIEEILNKTIMSKEDKK

>gi|33634322|emb|CAE20007.1| 50S Ribosomal protein L14 [Prochlorococcus marinus subsp. pastoris str. CCMP1986]

MIQQETYLTVADNSGAKRLQCIRVLGSNRRYAHVGDVVVASVKDALPNMGVKKSDVVKAVIVRTRHTLRR

NTGNSIRFDDNAAVLINEDKNPKGTRVFGPVARELRDKNFTKIVSLAPEVI

>gi|33634321|emb|CAE20006.1| 50S ribosomal protein L24 [Prochlorococcus marinus subsp. pastoris str. CCMP1986]

MLDSLKQKKNFKRIKMRIKTGDLVKVINGKEKGKTGEVLKTIPLENRVVVKGVNLRTKHVKPSQEGESGR

ILTEEASLHASNVMFFSKDKNLISKIEYFIDKEGVKKRRLKKTGELID

>gi|33634319|emb|CAE20004.1| 30S ribosomal protein S8 [Prochlorococcus marinus subsp. pastoris str. CCMP1986]

MSNHDPISDMLTRIRNASQKKHTSTAIPASKMSLSIAKVLQKEGFITDINEEGEGYKSQIVLGLKYSGKN

KFPTIRSMQRVSKPGLRVYKNTKGLPKVLGGLGVAIVSTSKGVMSDRDARKQGIGGEVLCYVY

>gi|33634317|emb|CAE20002.1| 50S ribosomal protein L18 [Prochlorococcus marinus subsp. pastoris str. CCMP1986]

MAKISRKLQTQKRHKRLRRYLIGSTARPRLAVFRSNNHIYAQVIDDDAQQTICSASTVDKELKEDKEKLS

SNCSSSSIVGKLLAKRAMKKGVKEVIFDRGGNLYHGRVKALADAARDAGLNF

>gi|33634315|emb|CAE20000.1| 50S ribosomal protein L15 [Prochlorococcus marinus subsp. pastoris str. CCMP1986]

MTSTLNTLKSNTGSRKKKLRKGRGIAAGQGASCGFGMRGQKSRSGRPTRPGFEGGQMPLYRRVPKLKHFE

IINQKNFSIVNLSKLSEFKESEVVNIDSLVKKKLLFKPKFPLKILGNGEVKVKLKVQAHAFTKVAKEKIE

AAGGSCEIINNK

>gi|33634312|emb|CAE19997.1| 50S Ribosomal protein L36 [Prochlorococcus marinus subsp. pastoris str. CCMP1986]

MKVRASVKKMCDKCRVIRRHGRVMVICTASPRHKQRQG

>gi|33634311|emb|CAE19996.1| 30S ribosomal protein S13 [Prochlorococcus marinus subsp. pastoris str. CCMP1986]

MARIAGIDIPREKRVEIALTYIYGVGLTRSKLILSNTGVNPDIRVKDLSDSDVQKLRGATEDFTVEGDLR

RKEGMAMKRLQDIGCVRGRRHRMSLPVRGQRTRTNARTRRGSRKTVAGRKK

>gi|33634310|emb|CAE19995.1| 30S ribosomal protein S11 [Prochlorococcus marinus subsp. pastoris str. CCMP1986]

MAAPVKKTGSKKSKKNVPNGVVHIQSTFNNTIVSITDTSGHVISWSSAGASGFKGARKGTPFAAQTAAEA

AAKRALDQGMRQIKVLVRGPGSGRETAIRALQVAGLEITLIRDVTPLPHNGCRRPKRRRV

>gi|33634308|emb|CAE19993.1| 50S ribosomal protein L17 [Prochlorococcus marinus subsp. pastoris str. CCMP1986]

MRHQLRVPLLSKPADQRKALLRALTTQLIREGRITTTKARAKALRNEAERMISLAKEGTLSARRRALGYI

YDKKLVHSLFEKAQERYGERNGGYTRIVRTVARKGDNAQMAIIELV

>gi|33634306|emb|CAE19991.1| 50S ribosomal protein L13 [Prochlorococcus marinus subsp. pastoris str. CCMP1986]

MNKTITPSIETIERNWFLVDAKDKTLGRLSTEIAAVLRGKNKPTFTPHLDTGDFVIVVNAEKVEVTGKKA

SQKLYRRHSGRPGGMKVEKFESLQERIPERIIEQAVKGMLPHNSLGRQQFKKLKVYKGSDHPHAAQNPVL

LNS

>gi|33634305|emb|CAE19990.1| 30S ribosomal protein S9 [Prochlorococcus marinus subsp. pastoris str. CCMP1986]

MNSQVKNKAVYWGTGRRKTSVARVRLIPGNGQIKINGRSGDDYLNFNPSHLNSVKAPLLTLGLENSYDIF

VNVFGGGLTGQADAIKQGAARALCGLSPDNRKPLKTEGHLSRDPRSKERKKYGLKKARKAGQFSKR

>gi|33634304|emb|CAE19989.1| 50S ribosomal protein L31 [Prochlorococcus marinus subsp. pastoris str. CCMP1986]

MPKSEIHPKWYPDAKVICNGEVVMTTGSTKPELHVDVWSGNHPFFTGTQKILDTEGRVDRFMKKYGMGSA

DSATSKETKESKKSDK

>gi|33634285|emb|CAE19970.1| 30S ribosomal protein S12 [Prochlorococcus marinus subsp. pastoris str. CCMP1986]

MPTISQLIGSERKRLTRKTKSPALKSCPERRGVCTRVYTSTPKKPNSALRKVARVRLTSGFEVTAYIPGI

GHNLQEHSVVLLRGGRVKDLPGVRYHIIRGTLDTAGVKDRRQSRSKYGAKAPKND

>gi|33634284|emb|CAE19969.1| 30S ribosomal protein S7 [Prochlorococcus marinus subsp. pastoris str. CCMP1986]

MSRRNAAVKRPVLPDPQFNSRLASMMISRLMKHGKKSTAQKILSDAFSLISERTGGNAVELFETAVKNAT

PLVEVRARRVGGATYQVPMEVRQERGTAMALRWLVTFSRGRNGKSMSQKLAGELMDAANETGSAVKKRED

THKMAEANKAFAHYRY

>gi|33634281|emb|CAE19966.1| 30S ribosomal protein S10 [Prochlorococcus marinus subsp. pastoris str. CCMP1986]

MTASLTQQKIRIRLKAFDRRMLDLSCDKIIQTADTTAASAIGPIPLPTKRKIYCVLRSPHVDKDSREHFE

TRTHRRIIDIYSPSAKTIDALMKLDLPSGVDIEVKL

>gi|33634261|emb|CAE19946.1| 30s Ribosomal protein S20 [Prochlorococcus marinus subsp. pastoris str. CCMP1986]

MANNKSAKKRIQVAERNRLVNKSYKSTVRTLTKKTLANCEKYKQEPNSDNKDLVLVSVNQAFSLIDKAVK

KNVLHKNNGANKKSKINKVVKDFLTSK

>gi|33634120|emb|CAE19446.1| 30S Ribosomal protein S21 [Prochlorococcus marinus subsp. pastoris str. CCMP1986]

MTQVTVGENEGIESALRRFKRQVSKSGIFADLKRLRHHETPIEKYKRKLQQRRKARRR

>gi|33634077|emb|CAE19402.1| 30S Ribosomal protein S15 [Prochlorococcus marinus subsp. pastoris str. CCMP1986]

MTLDTAEKQKLIESHQVHATDTGSVEVQVAMLSERISKLSDHLQGNIHDYASRQGLLKMIGKRKRLLSYI

KGKNPQNYQDLIKKIGIRG

>gi|33634036|emb|CAE19361.1| 50S ribosomal protein L28 [Prochlorococcus marinus subsp. pastoris str. CCMP1986]

MSRVCELTGAKANNGMAVSHSHIRTKKLQQVNLQKRRLWWQEGKKWVNIKISTKALKSIQKVGLDKVAKT

NGVDLNKF

>gi|33634004|emb|CAE19329.1| 50S Ribosomal protein L33 [Prochlorococcus marinus subsp. pastoris str. CCMP1986]

MAKKGTRVVVTLECTEARTSSEPRRSNGVSRYTTEKNKRNTTERLELKKFNPHLNKMTIHKEIK

>gi|33634003|emb|CAE19328.1| 30S Ribosomal protein S18 [Prochlorococcus marinus subsp. pastoris str. CCMP1986]

MPNSIFKKQLSPIKPGDPIDYKDVELLKKFITERGKILPRRMTGLTSKQQRDLTLAVKRARIVALLPFVN

PEG

>gi|33633987|emb|CAE19312.1| 50S ribosomal protein L32 [Prochlorococcus marinus subsp. pastoris str. CCMP1986]

MAVPKKKKSKSKRNHRHAVWKGKAALAAQKAMSLGKSVLTGKAQGFVYPIDEEEESEE

>gi|33633887|emb|CAE19212.1| 30S ribosomal protein S2 [Prochlorococcus marinus subsp. pastoris str. CCMP1986]

MAVVSLSEMMEAGAHFGHQTRRWNPKMSKYIYCARNGVHIIDLVKTALCMNNAYKWTRNAAKSGKRFLFV

GTKKQASDVVAQEAVRCGAAYVNQRWLGGMLTNWTTMKARIERLKDLERMESSGAIAMRPKKEAAVLRRE

LERLQKYLGGLKGMRRLPDVVVLVDQRRESNAVLEARKLDISLVSMLDTNCDPDLCEVPIPCNDDAVRSV

QLILGRLADAINEGRKGSNDQRKN

>gi|33639659|emb|CAE18828.1| possible Ribosomal protein S14p/S29e [Prochlorococcus marinus subsp. pastoris str. CCMP1986]

MLMQKRASKLETDFRIREAADLVIEGLAFSSITSYMSKKYTISRRQARRIAVDAYKVIRTDIEESDLDRK

EMTSKLVCLLENTMHLAMKEKQYSAVATNARVLMRLIRLE

>gi|33634325|emb|CAE20010.1| 50S ribosomal protein L16 [Prochlorococcus marinus subsp. pastoris str. CCMP1986]

MLSPKRTKFRKQHRGRMKGIASKGNTIAFGQFALQAQDCGWVTARQIEASRRAMTRYVKRGGKIWIRIFP

DKPVTMRPAETRMGSGKGNPEFWVAVVKPGRILFEMGGEEITEEIAKEAMRLAQYKLPVKTKFISSDINL

SSDSSGEGKTGKDSKEEVKK

>gi|33634324|emb|CAE20009.1| 50S ribosomal protein L29 [Prochlorococcus marinus subsp. pastoris str. CCMP1986]

MKNSESIKEFKKLNSSQITEKIDQLRKDLFDLRFKQATRQLNETHKFKIIKKQVAQLLTLSKSQSTSQKP

AD

>gi|33634478|emb|CAE20163.1| Molecular chaperone DnaK2, heat shock protein hsp70-2 [Prochlorococcus marinus subsp. pastoris str. CCMP1986]

MGKVVGIDLGTTNSCVAVMEGGKPTVIANAEGFRTTPSVVAYTKNQDQLVGQIAKRQAVMNPENTFYSAK

RFVGRRVDEVNEESKEVSYGIEKAGSNVKLKCPVLDKQFSPEEVSAQVLRKLSEDAGKYLGENITQAVIT

VPAYFNDSQRQATKDAGKIAGLEVLRIINEPTAAALAYGLDKKSNERILVFDLGGGTFDVSVLEVGDGVF

EVLSTSGDTHLGGDDFDRCIVDHLASIFKSNEGIDLRQDKQALQRLTEAAEKAKIELSNATQSEINLPFI

TATPEGPKHLDLNLTRANFEELASKLIDRCRVPVEQALKDAKLSTGEIDEIVMVGGSTRMPAVQELVKRV

TGKDPNQTVNPDEVVAVGAAIQGGVLAGEVKDILLLDVTPLSLGVETLGGVMTKMITRNTTVPTKKSETY

STAVDGQTNVEIHVLQGEREMASDNKSLGTFRLDGIPSAPRGVPQIEVTFDIDANGILSVTAKDKGSGKE

QSISITGASTLSDNEVDKMVKDAESNASVDKEKREKIDLKNQAETLVYQTEKQLGELGDKVDASAKAKVE

EKSKALKEATSKEDYEAMKKLLEELQQELYAIGSSVYQQPGNQPPAPGTPDSNESNDKGGDDDVIDADFT

ETKD

>gi|33634266|emb|CAE19951.1| N utilization substance protein A [Prochlorococcus marinus subsp. pastoris str. CCMP1986]

MALVILPGLNNLIEDISEEKKLPPHVVELALREALLKGYEKYRKTFYIGVKEDPFDEEYFSNFDVGFDLD

EEGYRILSSKIIVENVESEDHQISLQEVKQVADDAQIGDTVVLDVTPEKEDFGRMAASTTKQVLAQKLRD

QQRKMIQEEFADLEDPVLTARVIRFERQSVIMGVSSGIGRPEVEAELPKRDQLPNDNYRANATFKVFLKE

VSEVARKGPQLFVSRANAGLVVYLFENEVPEIQEGTVKIVAVSREANPPSRAVGPRTKVAVDSIEREVDP

VGACIGARGARIQQVVNELRGEKIDVIKWSSDPIQYILNSLSPAKVDLVRLVDPEGQHAHVLVPPDQLSL

AIGREGQNVRLAARLTGWKIDVKNSYEYDQETEDSAVAELIVQREEEENLQREAEQRLEAEQAERAAEDK

RLRELYPLPEDDEEYGDELYEEETRSENDQTENLKQEELLSKEENTR

>gi|33634222|emb|CAE19548.1| putative ribosomal-protein-alanine acetyltransferase [Prochlorococcus marinus subsp. pastoris str. CCMP1986]

MISIKEIDQKDFELCYKLDSATICLWTKKQWQSEFNKSGTKVVAILLKKKIIGIYVVQTIIDEAQISYFS

IKQKFRRKGYGSQLMTYLIKDCEKLNIKKLLLEVSETNSIAEIFYCKFNFLTVGRRKNYYKSGADAVLKE

KKFT

>gi|33633521|emb|CAE18478.1| conserved hypothetical protein [Prochlorococcus marinus subsp. pastoris str. CCMP1986]

MKVNKKYKGLVTKKFNEFYLVELDKYETSVANKKFLCKIKKSVNFRNQFVFVGDEVIVYQIDLQSKRATI

ESLVKRNNLLERPSVANISNIYVICSVEEPKLNLSQVNKFLISSEQLGVEVSLVLTKCDLITEEKRLLLI

EKFHQWGYQAITLNLNNPENLRTLLIELKKKKCSIFMGPSGVGKTTLLNMIIPNLDNKTAPVSSKIKRGK

NTTRNVELFSLSSKSYIVDTPGFNIQTLEIDIRELSNLYPEIYKQVVNEGIHCKFRNCLHVNDEGCKLNK

NFERYTFYKEMVESSKSHYCLIQED

>gi|33633604|emb|CAE18561.1| possible Fe-S oxidoreductase [Prochlorococcus marinus subsp. pastoris str. CCMP1986]

MKQNNQNVKDKKISKVAFSHVGCEKNLVDTEHMQGLLDKEGYEVGNDIEDANVVVVNTCSFIETAREESI

RKIIEFTDQGKEVIVAGCMAQHFKEELLKEMPEIKGLVGTGDYQKIAKVIKRVEKGEIVNEVSKIPEFIA

DEKIPRFVDKNKFVAYLRIAEGCDYKCAFCIIPKLRGPQRSRDIESIVSEAKNLAAQGIQEIILISQITT

NYGQDIYGKPSLARLLKELSKVSVPWIRIHYAYPTGLTDEVIKSFKDSNNIVPYFDLPLQHSHPDVLKSM

NRPWQASLNESILSQIREQIPSAVLRTSLIVGFPGENQKHFQHLLDFLHRHEFDHVGVFIFSPEEGTSAF

DLPNRVPSEVADARKDNIISIQQNISKKKNQLYVGTTIKVLVEKISENKELIGRSYNFAPEIDGNVILSI

KNYEDEKNYIGKFVEANICFADEYDLYGEVIKTL

>gi|300567859|emb|CBV15643.1| unnamed protein product [Prochlorococcus marinus subsp. pastoris str. CCMP1986]

MPKSEIHPKWYPDAKVICNGEVVMTTGSTKPELHVDVWSGNHPFFTGTQKILDTEGRVDRFMKKYGMGSA

DSATSKETKESKKSDK

>gi|259657491|emb|CBG02123.1| unnamed protein product [Prochlorococcus marinus subsp. pastoris str. CCMP1986]

MPKSEIHPKWYPDAKVICNGEVVMTTGSTKPELHVDVWSGNHPFFTGTQKILDTEGRVDRFMKKYGMGSA

DSATSKETKESKKSDK

>gi|81712576|sp|Q7UZV8.1|RL6_PROMP RecName: Full=50S ribosomal protein L6

MSRIGKSPVQIPEKVSVDIKGLSITVKGPKGELKRLMPEGVNFDQKENQIVVTPATTKRYSRERHGLCRT

LISNMVQGVTEGYEKKLEIVGVGSRAQVKGKNLVVSAGYSHPVEMTPPDGITYKVESNTNVTVSGIDKEI

VGNEAAKIRSIRPPEPYKGKGIKYQDERIIRKAGKSGKK

>gi|81575518|sp|Q7UZJ1.1|RL9_PROMP RecName: Full=50S ribosomal protein L9

MAKRVKVVLTESVATLGRDGDVVEVAPGYARNFLLPFGKAANVTPSILKQIERKRAKEKIAAEKVKQEAI

DFKTALATIGRFTIKKQVGEDGVLFGTVTNGDVAEAIEAATKKDIDRRDITVPDIHNLGSFVAKIKLHQE

VSAEVNIEVTS

>gi|73919090|sp|Q7UZW0.1|RS5_PROMP RecName: Full=30S ribosomal protein S5

MTDTPTKQENQSKTENPPSSNANEQRRGNRNNDRKRNRRGDSKNERDSEWQERVVQIRRVSKTVKGGKKM

SFRAIVVVGNEKGQVGVGVGKAGDVIGAVRKGVSDGKKHLVRVPLTPNNSIPTLSKGRDGAANVLIRPAA

PGTGVIAGGSIRTVLELAGIKNVLAKRLGSKTPLNNARAAMVALSQLRTHKSASRERGISLEQLYS

>gi|61215245|sp|Q7V383.1|RL10_PROMP RecName: Full=50S ribosomal protein L10

MGRTIENKQKIVTEIKSLLDDSEMAVVLDYKGLTIKEMSDLRSRLQTNNGICKVTKNSLMRKAIDGNSNW

TDLESLLTGTNAFVLIKEDVGGAVKAIQSFQKETKKSETKGALFEGRLLSESEIKEIASLPSREVLMAKI

AGALNGVATKIAISINEVPSGIARSLKQHSEKSES

>gi|38605171|sp|Q7V381.1|RL11_PROMP RecName: Full=50S ribosomal protein L11

MAKKIVAVIKLALQAGKANPAPPVGPALGQHGVNIMAFCKEYNARTQDKAGFVIPVEISVFEDRSFTFIT

KTPPASVLITKAAGIEKGAGESSKGSVGNISKSQLEEIAKTKLPDLNCTSIESAMKVIEGTARNMGVSIT

E

>gi|81576465|sp|Q7V384.1|RL7_PROMP RecName: Full=50S ribosomal protein L7/L12

MTAKTEEILDSLKSLSLLEASELVKQIEEAFGVSAAASAGVVMAAPGAGGGDGDGGAAEEKTEFDVILES

FDAAAKIKVLKVVRNATGLGLGDAKALVESAPKTVKEGIAKADAETLKKEIEEAGGKVTLK

>gi|61215698|sp|Q7V382.1|RL1_PROMP RecName: Full=50S ribosomal protein L1

MKKLSKRMKALSTKIEDRTYAPLEALGIVKENANAKFDETIEAHIRLGIDPKYTDQQLRTTVALPNGTGQ

SIKIAVITSGENVAKAKSAGADLFGEEDLVESINKGNMDFDLLIATPDMMPKVAKLGRVLGPRGLMPNPK

AGTVTGDIASAIKEFKAGKLEFRADKAGIVHVRFGKASFTENALFENLKTLQESIDKNKPSGAKGKYWRS

FYLTSTMGPSVQVDINALQDYQPES

>gi|61215695|sp|Q7UZV2.1|RS3_PROMP RecName: Full=30S ribosomal protein S3

MGNKINPTGFRLGITQEHRSKWFATSKTYPTLLQEDDKIRTFIQKKYSSAGISDVLIARKADQLELELKT

ARPGVIVGRQGSGIEELRSGIQKTIGDRTRQVRINVVEVERVDADAYLLAEYIAQQLEKRVAFRRTIRMA

LQRAQRAGVLGLKVQVGGRLNGAEIARTEWTREGRVPLHTLRAEVDYALREANTTYGVLGIKVWVFKGEV

LPKEEQTIPVGAIPRRKGSRKPQQFEDRSNENS

>gi|51316770|sp|Q7UZV1.1|RL22_PROMP RecName: Full=50S ribosomal protein L22

MTKTPEMTKTAIAHGKYIRGSASKVRRVLDQIRGKSYRDALIMLEFMPYRSTDPITKVLRSAVANAEHNL

GMDPSSLVISSASADNGPVMKRFRPRAQGRAFSIKKQTCHISISVESAPNQTNTEAQN

>gi|50403611|sp|Q7TU65.1|RS16_PROMP RecName: Full=30S ribosomal protein S16

MIKLRLKRFGKKKEASFRIVACNSTSRRDGRPLQELGFYNPRTKETRLDTEALRIRLTQGAQPTDVVRTL

LEKGGLLEKKVRPSIAIGKAKLEKEKIAKAKSKEAESDSKEAES

>gi|50401253|sp|Q7UZV6.1|RL5_PROMP RecName: Full=50S ribosomal protein L5

MTLKTRYKEAIRPKLLKDLGLKNIHQVPKVIKVNVNRGLGEAASNSKALEASLNEMATITGQKALVTRSK

KAIAGFKIREGMAIGCTVTLRGDRMYSFLERFINLALPRIRDFRGVNPKSFDGRGNYTLGVKEQLIFPEI

SFDKIDSIRGMDITIVTSASTDQEGKALLKELGMPFSN

>gi|41017826|sp|Q7V2R1.1|RS4_PROMP RecName: Full=30S ribosomal protein S4

MSRYRGPRLRVTRRLGELPGLTRKASKKSNPPGQHGQARRKRSEYAIRLEEKQKLRFNYGVSERQLVRYV

KKARAQEGSTGTNLLRLLENRLDNVCFRLGFGGTIPGSRQLVNHGHVTINGKVLDIAGYQCKPGDVISIK

ENKASKKLVEGNIEFPGLANVPPHIELDKPKLTGKINGKCDREWVALEINELLVVEYYSRKV

>gi|71649182|sp|Q7V0S1.1|RL34_PROMP RecName: Full=50S ribosomal protein L34

MTKRTFGGTSRKRKRVSGFRVRMRSHTGRRVIKSRRKRGRERIAV

>gi|59798833|sp|Q7TU30.1|RL36_PROMP RecName: Full=50S ribosomal protein L36

MKVRASVKKMCDKCRVIRRHGRVMVICTASPRHKQRQG

>gi|54036289|sp|Q7UZK2.1|RL35_PROMP RecName: Full=50S ribosomal protein L35

MSKLKTRKSAAKRFKATATGKFTRRRAFHNHLLDHKSSKLKRHLKTKAVVDERDADNVKLMIPYA

>gi|51316773|sp|Q7V1L2.1|RL32_PROMP RecName: Full=50S ribosomal protein L32

MAVPKKKKSKSKRNHRHAVWKGKAALAAQKAMSLGKSVLTGKAQGFVYPIDEEEESEE

>gi|81711983|sp|Q7TU88.1|RS21_PROMP RecName: Full=30S ribosomal protein S21

MTQVTVGENEGIESALRRFKRQVSKSGIFADLKRLRHHETPIEKYKRKLQQRRKARRR

>gi|67461501|sp|Q7V1H0.1|RL28_PROMP RecName: Full=50S ribosomal protein L28

MSRVCELTGAKANNGMAVSHSHIRTKKLQQVNLQKRRLWWQEGKKWVNIKISTKALKSIQKVGLDKVAKT

NGVDLNKF

>gi|81835544|sp|Q7UZU9.1|RL23_PROMP RecName: Full=50S ribosomal protein L23

MSRLFDSRLADIIRKPVITEKATNALDLNQYTFEVDHRAAKPQIKAAIEALFDVKVIGINTMNPPRRTRR

VGKFSGKRSQIKKAIVRLAEGDKIQLFPES

>gi|81834818|sp|Q7TU28.1|RS17_PROMP RecName: Full=30S ribosomal protein S17

MALKERIGTVVSDKMDKTVVVAVINRYPHPTYKKIVSKTTRYKAHDPENSCVLGDRVKIKETRPLSAHKR

WAIEEILNKTIMSKEDKK

>gi|81712587|sp|Q7V0C2.1|RL21_PROMP RecName: Full=50S ribosomal protein L21

MTSSKKPSNSSAKNENLYAIAETSGQQFWFEVDKYYDIDRLNAKEKDKITIDKILLIKDKDNISLGQPYV

KNAKIELEVVSHKRDKKIIVYKMRPKKKTRRKMGHRQELTRVMVKSISITNSTPKTSSKTEVKKKSTSPK

ASNPEN

>gi|81711985|sp|Q7TU99.1|RL33_PROMP RecName: Full=50S ribosomal protein L33

MAKKGTRVVVTLECTEARTSSEPRRSNGVSRYTTEKNKRNTTERLELKKFNPHLNKMTIHKEIK

>gi|73621817|sp|Q7UZX0.1|RL31_PROMP RecName: Full=50S ribosomal protein L31

MPKSEIHPKWYPDAKVICNGEVVMTTGSTKPELHVDVWSGNHPFFTGTQKILDTEGRVDRFMKKYGMGSA

DSATSKETKESKKSDK

>gi|67461500|sp|Q7V0C1.1|RL27_PROMP RecName: Full=50S ribosomal protein L27

MAHKKGTGSTRNGRDSNSKRLGVKAYGGEKVSAGSIIIRQRGTSFLPGINVGKGKDDTLFALKEGTVSFD

SIKRNLRNRKRVNVVL

>gi|67461180|sp|Q7UZV7.1|RS8_PROMP RecName: Full=30S ribosomal protein S8

MSNHDPISDMLTRIRNASQKKHTSTAIPASKMSLSIAKVLQKEGFITDINEEGEGYKSQIVLGLKYSGKN

KFPTIRSMQRVSKPGLRVYKNTKGLPKVLGGLGVAIVSTSKGVMSDRDARKQGIGGEVLCYVY

>gi|62287311|sp|Q7UZY5.1|RS7_PROMP RecName: Full=30S ribosomal protein S7

MSRRNAAVKRPVLPDPQFNSRLASMMISRLMKHGKKSTAQKILSDAFSLISERTGGNAVELFETAVKNAT

PLVEVRARRVGGATYQVPMEVRQERGTAMALRWLVTFSRGRNGKSMSQKLAGELMDAANETGSAVKKRED

THKMAEANKAFAHYRY

>gi|61216282|sp|Q7UZG1.1|RS6_PROMP RecName: Full=30S ribosomal protein S6

MTDQIYYETMYILRPDIAEDEVTNHIDKYNKLLEESGGKILDSQMRGKRRLAYQIAKHREGIYVQLSHQG

DGQHIFKIEKAMRLSEDVIRYLTVKQEGPLPTPRSSNKGYNQSEKKDIESIDSTNKSEFKEEANDKKTAT

SESTSSQGKESQKS

>gi|61215697|sp|Q7V1V0.1|RS2_PROMP RecName: Full=30S ribosomal protein S2

MAVVSLSEMMEAGAHFGHQTRRWNPKMSKYIYCARNGVHIIDLVKTALCMNNAYKWTRNAAKSGKRFLFV

GTKKQASDVVAQEAVRCGAAYVNQRWLGGMLTNWTTMKARIERLKDLERMESSGAIAMRPKKEAAVLRRE

LERLQKYLGGLKGMRRLPDVVVLVDQRRESNAVLEARKLDISLVSMLDTNCDPDLCEVPIPCNDDAVRSV

QLILGRLADAINEGRKGSNDQRKN

>gi|59798835|sp|Q7TUA0.1|RS18_PROMP RecName: Full=30S ribosomal protein S18

MPNSIFKKQLSPIKPGDPIDYKDVELLKKFITERGKILPRRMTGLTSKQQRDLTLAVKRARIVALLPFVN

PEG

>gi|59798834|sp|Q7TU38.1|RS20_PROMP RecName: Full=30S ribosomal protein S20

MANNKSAKKRIQVAERNRLVNKSYKSTVRTLTKKTLANCEKYKQEPNSDNKDLVLVSVNQAFSLIDKAVK

KNVLHKNNGANKKSKINKVVKDFLTSK

>gi|54036400|sp|Q7TU27.1|RS19_PROMP RecName: Full=30S ribosomal protein S19

MGRSLKKGPFIADSLLKKVEKQNTDNDKSVIKTWSRSSTILPVMIGHTIAVHNGKAHIPVFITEQMIGHK

LGEFAPTRTYRGHLRDKKGAR

>gi|46577194|sp|Q7UZU8.1|RL4_PROMP RecName: Full=50S ribosomal protein L4

MTTLETLKWDGKKAGKVSIDLKVAKETSSADLIHRAVLRQLANKRQGTASTLTRSEVRGGGRKPYKQKGT

GRARQGSIRTPLRPGGGVIFGPKPRSYNLDMNRKERRLALRTALMSRISDFKTVEDFGSTLDQPKTSEII

NGLSRLGIEKTEKVLVILDNPSDVIKKSINNLEKVKLIAADQLNVFDILNANKLVIGQSAINKIQEVYAS

>gi|46396793|sp|Q7UZV5.1|RL24_PROMP RecName: Full=50S ribosomal protein L24

MLDSLKQKKNFKRIKMRIKTGDLVKVINGKEKGKTGEVLKTIPLENRVVVKGVNLRTKHVKPSQEGESGR

ILTEEASLHASNVMFFSKDKNLISKIEYFIDKEGVKKRRLKKTGELID

>gi|42559598|sp|Q7UZU7.1|RL3_PROMP RecName: Full=50S ribosomal protein L3

MSIGILGKKLGMSQLFDKDGNAVPVTLIEAGPCRVTQLKTQPLDGYTAIQIGYGVSKDKHLSKPEKGHLL

KSGEILLKHLKEYRVEENSSYEIGKEITVTNFEVGQKVDISGKSMGRGFSGYQKRHGFSRGPMSHGSKNH

RAPGSTGAGTTPGRIYPGKRMAGRYGGKKITTKGLLVVKIDDQKNLLVVKGSVPGKPGSIVNIKPNNVVG

NKGGAKS

>gi|42559203|sp|Q7UZV0.1|RL2_PROMP RecName: Full=50S ribosomal protein L2

MAIRKFKPYTPGTRQRVVTDFSEITGSKPERSLIVSKHRNKGRNNRGVITCRHRGGGHKRQYRLVDFRRD

KKNINAKVAAIHYDPHRNARLALLFYEDGEKRYIIAPAGIKVGQNVISGEGVPIEEGNAMPLSSMPLGSN

VHCVELYAGRGAQMVRSAGASAQLMAKEGEYVALKLPSTEVRLVRKECYATLGEVGNSEIRNTSLGKAGR

RRWLGRRPQVRGSVMNPCDHPHGGGEGKAPIGRAGPVTPWGKAALGLKTRKKNKPSNKLVVRRRRRISKR

SRGGRDS

>gi|39932078|sp|Q7UZW9.1|RS9_PROMP RecName: Full=30S ribosomal protein S9

MNSQVKNKAVYWGTGRRKTSVARVRLIPGNGQIKINGRSGDDYLNFNPSHLNSVKAPLLTLGLENSYDIF

VNVFGGGLTGQADAIKQGAARALCGLSPDNRKPLKTEGHLSRDPRSKERKKYGLKKARKAGQFSKR

>gi|39931778|sp|Q7V2K1.1|RL19_PROMP RecName: Full=50S ribosomal protein L19

MIIEHKMAKEKQETELEITNETDTTTELTVEKQGKELIAQTNLSSSNLIKEFEREQLKKQLPEIYVGDTV

KVGVKITEGNKERVQPYEGVVIAKRHGGLHQTITVRRIFQGIGVERVFMLHSPQVASLKVERRGKVRRAK

LFYLRDRVGKATRVKQRFDR

>gi|39931773|sp|Q7UZK1.1|RL20_PROMP RecName: Full=50S ribosomal protein L20

MARVKRGNIARKRRNKILNLAKGFRGGNKNLFRTANQRVMKALCNAYRDRRRRKRDFRRLWISRINASAR

INGTNYSRLINGMKNSEIIINRKMLAQLALSDPQCFEKIVSTVNN

>gi|81712580|sp|Q7UZW8.1|RL13_PROMP RecName: Full=50S ribosomal protein L13

MNKTITPSIETIERNWFLVDAKDKTLGRLSTEIAAVLRGKNKPTFTPHLDTGDFVIVVNAEKVEVTGKKA

SQKLYRRHSGRPGGMKVEKFESLQERIPERIIEQAVKGMLPHNSLGRQQFKKLKVYKGSDHPHAAQNPVL

LNS

>gi|81712579|sp|Q7UZW7.1|RL17_PROMP RecName: Full=50S ribosomal protein L17

MRHQLRVPLLSKPADQRKALLRALTTQLIREGRITTTKARAKALRNEAERMISLAKEGTLSARRRALGYI

YDKKLVHSLFEKAQERYGERNGGYTRIVRTVARKGDNAQMAIIELV

>gi|81712577|sp|Q7UZW1.1|RL15_PROMP RecName: Full=50S ribosomal protein L15

MTSTLNTLKSNTGSRKKKLRKGRGIAAGQGASCGFGMRGQKSRSGRPTRPGFEGGQMPLYRRVPKLKHFE

IINQKNFSIVNLSKLSEFKESEVVNIDSLVKKKLLFKPKFPLKILGNGEVKVKLKVQAHAFTKVAKEKIE

AAGGSCEIINNK

>gi|81711984|sp|Q7TU92.1|RS15_PROMP RecName: Full=30S ribosomal protein S15

MTLDTAEKQKLIESHQVHATDTGSVEVQVAMLSERISKLSDHLQGNIHDYASRQGLLKMIGKRKRLLSYI

KGKNPQNYQDLIKKIGIRG

>gi|81711980|sp|Q7TU29.1|RL14_PROMP RecName: Full=50S ribosomal protein L14

MIQQETYLTVADNSGAKRLQCIRVLGSNRRYAHVGDVVVASVKDALPNMGVKKSDVVKAVIVRTRHTLRR

NTGNSIRFDDNAAVLINEDKNPKGTRVFGPVARELRDKNFTKIVSLAPEVI

>gi|81575584|sp|Q7UZW4.1|RS13_PROMP RecName: Full=30S ribosomal protein S13

MARIAGIDIPREKRVEIALTYIYGVGLTRSKLILSNTGVNPDIRVKDLSDSDVQKLRGATEDFTVEGDLR

RKEGMAMKRLQDIGCVRGRRHRMSLPVRGQRTRTNARTRRGSRKTVAGRKK

>gi|81572983|sp|Q7TU67.1|RS14_PROMP RecName: Full=30S ribosomal protein S14

MAKKSMIAREVKRKKLVKKYATKRKSLLDEFNAAKDPMERLEIHRKIQGLPRNSAPTRVRNRCWATGKPR

GVYRDFGLCRNQLRLRAHNGELPGVVKSSW

>gi|73621672|sp|Q7UZV9.1|RL18_PROMP RecName: Full=50S ribosomal protein L18

MAKISRKLQTQKRHKRLRRYLIGSTARPRLAVFRSNNHIYAQVIDDDAQQTICSASTVDKELKEDKEKLS

SNCSSSSIVGKLLAKRAMKKGVKEVIFDRGGNLYHGRVKALADAARDAGLNF

>gi|59798848|sp|Q7UZW5.1|RS11_PROMP RecName: Full=30S ribosomal protein S11

MAAPVKKTGSKKSKKNVPNGVVHIQSTFNNTIVSITDTSGHVISWSSAGASGFKGARKGTPFAAQTAAEA

AAKRALDQGMRQIKVLVRGPGSGRETAIRALQVAGLEITLIRDVTPLPHNGCRRPKRRRV

>gi|52783391|sp|Q7UZY4.1|RS12_PROMP RecName: Full=30S ribosomal protein S12

MPTISQLIGSERKRLTRKTKSPALKSCPERRGVCTRVYTSTPKKPNSALRKVARVRLTSGFEVTAYIPGI

GHNLQEHSVVLLRGGRVKDLPGVRYHIIRGTLDTAGVKDRRQSRSKYGAKAPKND

>gi|44888408|sp|Q7UZY8.1|RS10_PROMP RecName: Full=30S ribosomal protein S10

MTASLTQQKIRIRLKAFDRRMLDLSCDKIIQTADTTAASAIGPIPLPTKRKIYCVLRSPHVDKDSREHFE

TRTHRRIIDIYSPSAKTIDALMKLDLPSGVDIEVKL

>gi|46577386|sp|Q7V048.2|RRP3_PROMP RecName: Full=Probable 30S ribosomal protein PSRP-3; AltName: Full=Ycf65-like protein

MMGANAVLAAAKIDEDGVPTGYTPKPDEGRFIIKILWLPDNVALAVDQIVGGGSSPLTAYYFWPRDDAWE

KLKSELENKSWITDNERVEILNKATEVINYWQEEGKTKKLEEAKLKFPEVAFCGTA

>gi|73917121|sp|Q7UZV4.1|RL29_PROMP RecName: Full=50S ribosomal protein L29

MKNSESIKEFKKLNSSQITEKIDQLRKDLFDLRFKQATRQLNETHKFKIIKKQVAQLLTLSKSQSTSQKP

AD

>gi|81575583|sp|Q7UZV3.1|RL16_PROMP RecName: Full=50S ribosomal protein L16

MLSPKRTKFRKQHRGRMKGIASKGNTIAFGQFALQAQDCGWVTARQIEASRRAMTRYVKRGGKIWIRIFP

DKPVTMRPAETRMGSGKGNPEFWVAVVKPGRILFEMGGEEITEEIAKEAMRLAQYKLPVKTKFISSDINL

SSDSSGEGKTGKDSKEEVKK

>gi|81576526|sp|Q7V3H3.1|RIMO_PROMP RecName: Full=Ribosomal protein S12 methylthiotransferase RimO; Short=S12 MTTase; Short=S12 methylthiotransferase; AltName: Full=Ribosomal protein S12 (aspartate-C(3))-methylthiotransferase; AltName: Full=Ribosome maturation factor RimO

MKQNNQNVKDKKISKVAFSHVGCEKNLVDTEHMQGLLDKEGYEVGNDIEDANVVVVNTCSFIETAREESI

RKIIEFTDQGKEVIVAGCMAQHFKEELLKEMPEIKGLVGTGDYQKIAKVIKRVEKGEIVNEVSKIPEFIA

DEKIPRFVDKNKFVAYLRIAEGCDYKCAFCIIPKLRGPQRSRDIESIVSEAKNLAAQGIQEIILISQITT

NYGQDIYGKPSLARLLKELSKVSVPWIRIHYAYPTGLTDEVIKSFKDSNNIVPYFDLPLQHSHPDVLKSM

NRPWQASLNESILSQIREQIPSAVLRTSLIVGFPGENQKHFQHLLDFLHRHEFDHVGVFIFSPEEGTSAF

DLPNRVPSEVADARKDNIISIQQNISKKKNQLYVGTTIKVLVEKISENKELIGRSYNFAPEIDGNVILSI

KNYEDEKNYIGKFVEANICFADEYDLYGEVIKTL

>gi|38605163|sp|Q7TU56.1|PRMA_PROMP RecName: Full=Ribosomal protein L11 methyltransferase; Short=L11 Mtase

MEIKNWYELTFEIETNLEEIIIWKLNELGISSYAFEILLNNKNNKKVIIWLPHLNWPESLRIKLVRNIKE

VLDKNNYQTNCFEWNLIEQEDWISSWKKYWGPEIVGDNLLILPCWLELPEEYKNKKVIKIDPGAAFGTGS

HPTTSLCLEELEKISLSSKKILDIGSGSGILSIAARSFGASKIYSIDNDYLAINSTESNFRLNFGNLDNL

KTYLGSFDGLVSKYTLKNFDFILCNILAEVIKGIIPDIRNCLKIDGEVILSGILNSQKDEIIKLLKASNL

RINDVSSKKDWVCITAQKIP

>gi|48474356|sp|Q7UZQ3.1|RIMM_PROMP RecName: Full=Ribosome maturation factor RimM

MINHNEWLIVGLITSPQGINGKIKIKSLSDFEERFTKPGKRWIQKGNETPIEFELTHGFKKPGKESFIIT

FKGINNRTQAENLKGQKILVKVDAIPKLSHGEYHLTELINLNVKISENNQLHIIGKVINLSNEKNNLLVI

QLLKNNKEVLIPFVKEIVPIVDIKKNFILLTPPSGLLEL

>gi|39930991|sp|Q7UZG3.1|DNAK2_PROMP RecName: Full=Chaperone protein dnaK2; AltName: Full=HSP70-2; AltName: Full=Heat shock 70 kDa protein 2; AltName: Full=Heat shock protein 70-2

MGKVVGIDLGTTNSCVAVMEGGKPTVIANAEGFRTTPSVVAYTKNQDQLVGQIAKRQAVMNPENTFYSAK

RFVGRRVDEVNEESKEVSYGIEKAGSNVKLKCPVLDKQFSPEEVSAQVLRKLSEDAGKYLGENITQAVIT

VPAYFNDSQRQATKDAGKIAGLEVLRIINEPTAAALAYGLDKKSNERILVFDLGGGTFDVSVLEVGDGVF

EVLSTSGDTHLGGDDFDRCIVDHLASIFKSNEGIDLRQDKQALQRLTEAAEKAKIELSNATQSEINLPFI

TATPEGPKHLDLNLTRANFEELASKLIDRCRVPVEQALKDAKLSTGEIDEIVMVGGSTRMPAVQELVKRV

TGKDPNQTVNPDEVVAVGAAIQGGVLAGEVKDILLLDVTPLSLGVETLGGVMTKMITRNTTVPTKKSETY

STAVDGQTNVEIHVLQGEREMASDNKSLGTFRLDGIPSAPRGVPQIEVTFDIDANGILSVTAKDKGSGKE

QSISITGASTLSDNEVDKMVKDAESNASVDKEKREKIDLKNQAETLVYQTEKQLGELGDKVDASAKAKVE

EKSKALKEATSKEDYEAMKKLLEELQQELYAIGSSVYQQPGNQPPAPGTPDSNESNDKGGDDDVIDADFT

ETKD

>gi|159888250|gb|ABX08464.1| 30S ribosomal protein S1-like protein B, putative Nbp1 [Prochlorococcus marinus str. MIT 9211]

MAGSESTQPNRPNPFGKASDLPRKPLQVMHISRKKEQERLRKEEKNALKEKKSADQKNDAKESIENAYPP

SRPPKSDNKTYTNDLNLDVQEGVSMEDLLKEETIKSPGFNQADSMERILDDFDFDEEAFLEALNENEPIG

STGEIAKGCVIGIESDGVYVDIGGKAPGFMPKNECGLGVITNLKERFPKGLEVEVLVTREQNADGMVTIS

CRALVLRKSWETVMQLEKDGKTVEVTINGFNRGGVTCDLEGLRGFIPRSQLEQTENHESLVGKKLCVAFI

EVNPDSRKLILSEKKAATAARFAELEIGQIVAGEILAIKPYGFFVDLGGVSGLLHQSMITNGSIRSLREV

FSQGESIKALITDLDPKRGRIGLNTALLEGPPGEILVEKAKVLLEAEDRAKKVRGILKKKEESTEE

>gi|159888064|gb|ABX08278.1| 30S ribosomal protein S1-like protein A [Prochlorococcus marinus str. MIT 9211]

MVENSPEASQKLETEEQAAKSIDESQTSSPEINNKTPEETGNQVDTDIPEDIPTADDPSSRVKKHDFDGV

GFTLEEFDSLLSKYDYNFKPGDIVNGTVFALETKGAMIDIGAKTAAFMPMQEVSINRVEGLSDVLQPSEV

RQFFIMSEENEDGQLSLSIRRIEYQRAWERVRQLQKEDATIYSEVFATNRGGALVRVEGLRGFIPGSHIS

TRKAKEELVAEFLPLKFLEVDEERNRLVLSHRRALVERKMNRLEVGEVVVGAVRGIKPYGAFIDIGGVSG

LLHISEISHEHIETPHSVLNVNDQMKVMIIDLDAERGRISLSTKALEPEPGDMLSDPQKVFDKAEEMAAK

YKEMLLEQAEEGENPIATMEI

>gi|159889517|gb|ABX09731.1| 50S ribosomal protein L9 [Prochlorococcus marinus str. MIT 9211]

MAKRVQLVLKEDILSLGKDGDVVEVAPGYARNFLLPHGKALPVTPAVMKQVEHRRAKQKEHEAALKDEAL

AFETALKTIGRFTVKKQVGDDGVLFGTVTNGDIAEAIEKATEKEIDRRTISVPEVHAIGQYKVQIKLHHE

VTAEINLEVVSY

>gi|159889381|gb|ABX09595.1| 50S ribosomal protein L6 [Prochlorococcus marinus str. MIT 9211]

MSRIGKQPIPVPEKVAVELDGLSLTVKGPKGELSRTLPEGVSISQVDNSIVVSAINSKRKSRERHGLSRS

LVANMVEGVSKGYSKKLEIVGVGSRAQVKGKNLIVSAGYSHPVEVIPPDGITFVVENNTNVTVSGIDKEL

VGNEAAKIRAIRPPEPYKGKGIKYAGERIIRKAGKSGKK

>gi|159889379|gb|ABX09593.1| 30S ribosomal protein S5 [Prochlorococcus marinus str. MIT 9211]

MSDTPNKNQTKETSSAVPAAAEGQQQQQRKGGGNRGEKRNRRSGRNQERDSEWQERVVQIRRVSKTVKGG

KKMSFRAIVVVGNEKGQVGVGVGKAGDVIGAVRKGVADGKKNLVKVPLTRNSSIPTLSNGRDGAASVLIR

PAAPGTGVIAGGSIRTVLELAGIKNVLAKRLGSKTPLNNARAAMVALAELRTHKGTAKERGISLEQIYS

>gi|159887939|gb|ABX08153.1| 50S ribosomal protein L11 [Prochlorococcus marinus str. MIT 9211]

MAKKIVAVIKLALQAGKANPAPPVGPALGQHGVNIMAFCKEYNAKTQDKAGFVIPVEISVFEDRSFTFIT

KTPPASVLITKAAGIEKGSGDSAKGQVGTINRAQLEEIAKTKLPDLNCSNIESAMRVIEGTARNMGVSVK

D

>gi|159887937|gb|ABX08151.1| 50S ribosomal protein L10 [Prochlorococcus marinus str. MIT 9211]

MGRTLESKKQIVEELKALLDQAEMALVIDYQGLTIKEMSDLRTRLGPSSGICKVTKNTLMRKAIDGDTSW

SSLESLLNGTNAFVLVKGDVGGALKAVQAFQKETKKSKTKGGLFEGKLLSQDEIKAIANLPTKEVLMAQI

AGALNSIATKMAVGINEVPSGLARSLKQHADSGEN

>gi|159889390|gb|ABX09604.1| 50S ribosomal protein L22 [Prochlorococcus marinus str. MIT 9211]

MVESSSSKTKFAQAHGRYIRGSASKVRRVLDQIRGRTYRDALIMLEFMPYRSTGPITKVLRSAVANAENN

MGLDPASLVITRATADMAPSMKRYRPRAQGRAFAIKKQTCHISISVAPSSESTNSEASD

>gi|159889389|gb|ABX09603.1| 30S ribosomal protein S3 [Prochlorococcus marinus str. MIT 9211]

MGHKIHPNGLRLGITQEHRSRWYASSKTYPTLLQEDDRIRGFIQKKYASAGISDVLIARKADQLEVELKT

ARPGVIVGRQGSGIEELRSGIQKTIGDRSRQVRINVVEIERVDADAHLLAEYIAQQLEKRVAFRRTIRMA

VQRAQRAGVLGLKIQVGGRLNGAEIARSEWTREGRVPLHTLRAEIDYANKTANTTYGVLGIKVWVFKGEV

LSKEDQPLPVGASPRRKGSRRPQQFEDRSNDGK

>gi|159889383|gb|ABX09597.1| 50S ribosomal protein L5 [Prochlorococcus marinus str. MIT 9211]

MSLKHRYRETIRPKLLKDLGLSNIHQVPKVVKVNVNRGLGEAAQNSKTLEASLSEMATITGQKALVTRAK

KAIAGFKIREGMPIGCTVTLRGERMYAFLERLINLALPRIRDFRGVSPKSFDGRGNYTLGVKEQLIFPEI

SFDKIDTIRGMDITIVTSARSDEEGRALLKALGFPFRST

>gi|159888125|gb|ABX08339.1| 30S ribosomal protein S4 [Prochlorococcus marinus str. MIT 9211]

MSRYRGPRLRITRRLGDLPGLTRKAAKRSNPPGQHGQARRKRSEYAIRLEEKQKLRFNYGISERQLVRYV

KKARSLEGSTGTNLLKLLENRLDNVCFRLGFGPTIPGSRQLVNHGHVTVNGKVLDIASYQCKPGDVISIR

EKKGSKKLAEGNLEFPGLANVPPHLEFEKSKMSAKITGKCEREWVAIEINELLVVEYYSRKV

>gi|159887938|gb|ABX08152.1| 50S ribosomal protein L1 [Prochlorococcus marinus str. MIT 9211]

MTKISKRMASLSSKIEDRAYPPLEAINLVKESSTAKFDETIEAHVRLGIDPKYTDQQIRTTVTLPNGTGQ

TIRIAVIARGEKVAEAKSAGADLAGEEELVDSISKGEMGFDLLIATPDMMPKVAKLGRVLGPRGLMPNPK

TGTVTADLVGAIKEFKAGKLEFRADKAGIIHVRFGKASFNADALLENLKTLQETIDRNKPSGAKGRFWKT

LYITSTMGPSIEVDIAALQDISQE

>gi|159887936|gb|ABX08150.1| 50S ribosomal protein L7/L12 [Prochlorococcus marinus str. MIT 9211]

MSKKTDEILDSLKSLSLLEASELVKQIEEAFGVSAAASAGVVMAAPGAGGGASAAGEAAEEKTEFDVILE

SFDAAAKIKVLKEVRNATGLGLGEAKAMVEAAPKTIKEGASKEDAEALKKAIEAVGGKVTLK

>gi|159889560|gb|ABX09774.1| 30S ribosomal protein S6 [Prochlorococcus marinus str. MIT 9211]

MTNQPYYETMYILRPTIPEDEVDSHLKKYTEILESAGGEVLDSQMRGKRRLAYPIGKHKEGIYVQLSHQG

DGQHIAVLEKAMRLTEDVIRYLTVKQDGPLPAKRVVKTSEKNVKEDKEVENKETTTEDKDQKGDLKETKK

SENKDSVTEAEGQKDIKEAKEIENKEIEKKED

>gi|159889505|gb|ABX09719.1| 50S ribosomal protein L20 [Prochlorococcus marinus str. MIT 9211]

MSRVKRGNVARKRRNKILRIAKGYRGGNGKLFRTANQRVMKALCNAYRDRKRRKRDFRRLWIARINAAAR

LNGMSYSKLMGNLKKADVRINRKMLAQLAVIDPKSFKNVVTHSQK

>gi|159889504|gb|ABX09718.1| 50S ribosomal protein L35 [Prochlorococcus marinus str. MIT 9211]

MPKLKTRKAAAKRFKVTGTGKFMRRRAFRNHLLDHKSTKLKRHLATKAVVDERDADNVSLMLPYS

>gi|159889395|gb|ABX09609.1| 50S ribosomal protein L3 [Prochlorococcus marinus str. MIT 9211]

MSIGILGKKLGMSQFFDDKGRSVPVTLIEAGPCRITQLKSLATDGYSAVQIGYGFTREKLVNKPFKGHLS

KSGEGFLRHLHEYRVDDLGVYELGAKITVGNFEAGQKVDVSGNSMGRGFAGYQKRHSFSRGPMSHGSKNH

RQPGSTGAGTTPGRIYPGKRMAGRYGGKKITTRGLTIMKIDSDRNLLVVKGSVPGKPGSLLNIRPAQRVG

SQNLTGGKK

>gi|159889394|gb|ABX09608.1| 50S ribosomal protein L4 [Prochlorococcus marinus str. MIT 9211]

MANCVVLDWQGQEAGKATLDLKVAKETTAVDLIHRAVVRQQAHSRQGTASTLTRSEVRGGGRKPYKQKGT

GRARQGSIRTPLRPGGGIIFGPKPRSFSLGMNRKERRLALRTALMARINDLMIVQDFGTALKVPKTREIV

EALSRFGIADDAKVLIILAQPSEIIIRSVRNIERVKVIGADQLNVFDLLNANSLVIGEKALSTIKEVYGD

D

>gi|159889393|gb|ABX09607.1| 50S ribosomal protein L23 [Prochlorococcus marinus str. MIT 9211]

MFKERLADVIRRPLITEKATRGLDLNQYTFEVDPRAAKPDIKAAIEKMFDVKVIGISTMNPPRRTRRVGR

FAGKRSQVKKAIVRLAEGNTIQLFPEA

>gi|159889392|gb|ABX09606.1| 50S ribosomal protein L2 [Prochlorococcus marinus str. MIT 9211]

MAIRTFRPYTPGTRTRVVTDFNELTGRKPERSLVVSKHRLKGRNNRGVITCRHRGGGHKRLYRIVDFRRN

KHDVPAKVAAIHYDPHRNARLALLFYSDGEKRYILAPAGIAIGQEVISGPKVPIETGNAMPLSAIPLGSS

VHCVELYAGRGGQMVRSAGASAQVMAKEGDYVALRLPSTEVRLIRRECYATLGEVGNSEIRNTSLGKAGR

RRWLGRRPQVRGSVMNPCDHPHGGGEGRAPVGRAGPVTPWGKPALGLKTRKRNKPSNRFVLRKRRRVSKR

SRGGRDS

>gi|159889391|gb|ABX09605.1| 30S Ribosomal protein S19 [Prochlorococcus marinus str. MIT 9211]

MGRSLKKGPFIADSLLKKVEKQNSNDDRSVIKTWSRASTILPVMIGHTIAVHNGKSHIPVFITEQMVGHK

LGEFAPTRTYKGHIKDKKGAR

>gi|159889386|gb|ABX09600.1| 30S Ribosomal protein S17 [Prochlorococcus marinus str. MIT 9211]

MALKERLGTVVSDKMDKTVVVAVENRFPHPIYQKIVSRTARYKVHDAENTCKVGDKVRITETRPLSASKR

WTVAEVLRSTHEIKEVSK

>gi|159889385|gb|ABX09599.1| 50S Ribosomal protein L14 [Prochlorococcus marinus str. MIT 9211]

MIQQETFLTVADNSGAKKLQCIRVLGSNRRYAHVGDVIVAAVKDALPNMGVKKSDVVKAVVVRTKATLRR

ETGNSIRFDDNAAVLINEDKNPRGTRVFGPVARELRERNFTKIVSLAPEVI

>gi|159889384|gb|ABX09598.1| 50S ribosomal protein L24 [Prochlorococcus marinus str. MIT 9211]

MRIRKGDTVQVINGKEKGKTGEVLKTLPFENRVVVQGINLRTRHVKPTQEGETGRIVTEEASVHASNVMI

YSTEKKVASKVEIFIEKDGSKKRRLKKTGELID

>gi|159889382|gb|ABX09596.1| 30S ribosomal protein S8 [Prochlorococcus marinus str. MIT 9211]

MANHDPISDMLTRIRNASEKRHETTRIPASRMSRSIAKVLQKEGFIAQISEEGEGFKTQLVLELKYSGKH

RHPTIRSMQRVSKPGLRIYKNTRGLPKILGGLGVAIISTSKGVMSDRDARKQGVGGEVLCYVY

>gi|159889380|gb|ABX09594.1| 50S ribosomal protein L18 [Prochlorococcus marinus str. MIT 9211]

MATLSKKQQTQKRHKRLRRHLNGTNHRPRLAVFRSNNHIYAQVIDDEAQSTICSASTLDKDLREKLKASG

GSCDASMAVGALLAQRALAKGIEQVVFDRGGNLYHGRVKALAKSAREAGLKF

>gi|159889378|gb|ABX09592.1| 50S ribosomal protein L15 [Prochlorococcus marinus str. MIT 9211]

MTIKLDSLKPNKGSRRRKLRKGRGIAAGQGASCGFGMRGQKSRSGRPTRPGFEGGQMPLYRRVPKLKHFP

LVNPKSYSIVNVASLNQLKDGSKVNLDVLVKEGILTSPKYPLKVLGNGNLKVKLNVQAAAFTMSAKSKIE

GAGGTCETFDYKKFD

>gi|159889374|gb|ABX09588.1| 30S ribosomal protein S13 [Prochlorococcus marinus str. MIT 9211]

MARIAGIDIPREKRVEVALTYIYGVGLTRAKSILAKAGVNPDIRVKDLDDGDVQKLRTAVESFTLEGDLR

RQEGMALKRLQDIGCLRGRRHRMSLPVRGQRTRTNARTRRGSRKTVAGRKK

>gi|159889373|gb|ABX09587.1| 30S ribosomal protein S11 [Prochlorococcus marinus str. MIT 9211]

MATPAKKTGSKKSKRNVPNGVVHIQSTFNNTIVSITDTNGEVVSWSSAGASGFKGARKGTPFAAQTAAEA

AARRALEQGMRQIEVLVRGPGSGRETAIRALQVAGLEITLIRDVTPLPHNGCRRPKRRRV

>gi|159889371|gb|ABX09585.1| 50S ribosomal protein L17 [Prochlorococcus marinus str. MIT 9211]

MRHQLRVPKLGRPADQRKAILRGLTTQLIREGRVTTTKAKAKALRNEAERMITLAKEGTLAARRRAIGYI

YDKKLVHQLFEKAQDRYGDREGGYTRIVRTVPRRGDNAEMAIIELV

>gi|159889369|gb|ABX09583.1| 50S ribosomal protein L13 [Prochlorococcus marinus str. MIT 9211]

MFKKVLFFIKQMYLPIRYAGEMNKTITPKIDAIDRQWYLVDAENQTLGRLASKIASVLRGKNKPNFTPHL

DTGDFVVVVNAEKIQISGKKSQQKLYRRHSGRPGGMKVETFNSLQERIPERIVEKAVKGMLPHNSLGRQL

FRKLKVYKGADHPHAAQEPKLLNLDTNGLI

>gi|159889368|gb|ABX09582.1| 30S ribosomal protein S9 [Prochlorococcus marinus str. MIT 9211]

MNSSSQRNTVVYWGTGRRKTSVARVRLIPGTGKITINGRPGDHYLNFNPAYLAAVKAPLQTLGLSDSYDV

LVNVYGGGLTGQADAIKQGAARALCELSADNRKPLKIEGHLSRDPRAKERRKYGLKKARKAPQFSKR

>gi|159889367|gb|ABX09581.1| 50S ribosomal protein L31 [Prochlorococcus marinus str. MIT 9211]

MPKEDIHPTWYPDAKVICNGEVVMTTGSTQPEIHVDVWSGNHPFFTGTQKILDTEGRVDRFMRKYGMADS

ENDSTDKKKTTNEKKVSDSPSKES

>gi|159889351|gb|ABX09565.1| 30S ribosomal protein S12 [Prochlorococcus marinus str. MIT 9211]

MPTIQQLIRTERKRLTRKTKSPALRSCPERRGVCTRVYTSTPKKPNSALRKVARVRLTSGFEVTAYIPGI

GHNLQEHSVVLLRGGRVKDLPGVRYHIIRGTLDTAGVKDRRQSRSKYGAKVPK

>gi|159889350|gb|ABX09564.1| 30S ribosomal protein S7 [Prochlorococcus marinus str. MIT 9211]

MSRRNAAEKRPVLPDPQFNNRLATMMVARLMKHGKKSTAQRILSDAFGLINERTGSDPIELFETAVKNAT

PLVEVRARRVGGATYQVPMEVRQERGTAMALRWLVNFSRSRNGRSMAHKLAGELMDAANEAGNAVRKREE

THKMAEANKAFAHYRY

>gi|159889347|gb|ABX09561.1| 30S ribosomal protein S10 [Prochlorococcus marinus str. MIT 9211]

MSTAIAQQKIRIRLKAFDRRMLDLSCDKIIETADNTAATAIGPIPLPTKRKIYCVLRSPHVDKDSREHFE

TRTHRRIIDIYNPSAKTIDALMKLDLPSGVDIEVKL

>gi|159889326|gb|ABX09540.1| 30s Ribosomal protein S20 [Prochlorococcus marinus str. MIT 9211]

MANNNSAKKRIQIAERNRLQNRSYKSAMRTLMKRCLTAAGSYLEKPGEEAKANLQQNINEAFSKIDKAVK

KGVLHRNNGANKKSRLNAAVKKLIEPATKR

>gi|159889125|gb|ABX09339.1| putative methyltransferase for Ribosomal protein L11 [Prochlorococcus marinus str. MIT 9211]

MIDFRNIFWWRLKLPVPHELEESMIWKLQHLSIKSYAIEIDPKNNSRSFFYIWLISSEWPKYQREQLINC

FKPLARTFDKTLEQVTWEKVDDEDWSSSWKKYWGPAPVGKRLLILPAWMDLPASYSERIVVKLDPGAAFG

TGDHPTTKLCLEAIERQRPKDLRIVDIGCGSGVLGLAALRLGAKEVIGVDIDPLAIGSAQRNAFLNDFDE

NSFRTFHGSIDTVHNELQGAKADLLLCNILAPVIKTLGEDFDRVISPQGNALISGLLVEQMQDITKFLVD

LGWNFIASYQQDNWALIQLSKSSRH

>gi|159889116|gb|ABX09330.1| 50S ribosomal protein L27 [Prochlorococcus marinus str. MIT 9211]

MAHKKGTGSTRNGRDSNSKRLGVKAYGGEPVTAGSILIRQRGTSVLPGINVGKGKDDTLFALTDGIVTFE

TIRRGLKNRKRISVALTS

>gi|159889115|gb|ABX09329.1| 50S ribosomal protein L21 [Prochlorococcus marinus str. MIT 9211]

MTSSKKTTDKSTTSSSSSNYAIVETSGSQFWLEANRYYELDRINADIDEIITLDKVLLLNDEKGITLGKP

YIDGAKVEVKVIAHRRGPKIIVYKMRPKKKTRRKNGHRQELTRVMVQSIKTAANKVSATKGKVAAPKVQT

KKEAKGSKTVNKAKEPSKVKTPKTKGTATT

>gi|159889049|gb|ABX09263.1| 30S Ribosomal protein S16 [Prochlorococcus marinus str. MIT 9211]

MIKLRLKRYGKKREASFRLVACNSTSRRDGRPLEELGFYNPRTKETRLDTEALRTRLSQGAQPTDAVRSL

LEKGGLIEKTIRPAEIEGKKKQALARQSASKKAVKEKTEESKGSEVDSETSTSAD

>gi|159888994|gb|ABX09208.1| 30S Ribosomal protein S14 [Prochlorococcus marinus str. MIT 9211]

MAKKSMIARDVKRKKLVERYATKRKKLLDEFNSAKDPMERLEIHRKIQALPRNSAPSRMRNRCWATGKPR

GVYRDFGLCRNQLRERAHKGELPGVVKSSW

>gi|159888727|gb|ABX08941.1| 30S ribosomal protein S2 [Prochlorococcus marinus str. MIT 9211]

MAVVTLSEMMEAGAHFGHQTRRWNPKMSRYIYCARNGVHIIDLVKTAVCMNSAYKWTRNAAKSGKRFLFV

GTKKQASEVVALEANRCGASYVNQRWLGGMLTNWTTMKARIDRLKDLERMESSGAIAMRPKKEASVLRHE

LERLQKYLGGLKGMKRLPDVVVLVDQRRETNAVLEARKLDIPLVSMLDTNCDPDLCEVPIPCNDDAVRSV

QLILGRLADAINEGRHGSNEHRGGQRLK

>gi|159888612|gb|ABX08826.1| 30S Ribosomal protein S18 [Prochlorococcus marinus str. MIT 9211]

MPSSVFKKKLSPIKPGDPIDYKDVETLKKFITERGKILPRRLTGLTAQQQRDLTVAVKRARIIALLPFVN

PEG

>gi|159888611|gb|ABX08825.1| 50S Ribosomal protein L33 [Prochlorococcus marinus str. MIT 9211]

MAKKGTRVVVTLECTECRTVPPSEKRSPGVSRYTTEKNRRNTTERLELKKFCPQLNKMTIHKEIK

>gi|159888575|gb|ABX08789.1| 50S ribosomal protein L28 [Prochlorococcus marinus str. MIT 9211]

MSRVCDLSGTRANNGMAVSHSHIRTKKLQQANLQQRKLWWEEGKKWIKVRVTTRTLKTIQKKGLNSYAKA

MGIDLSKV

>gi|159888418|gb|ABX08632.1| 30S Ribosomal protein S15 [Prochlorococcus marinus str. MIT 9211]

MTLNTQEKQKLINTHQNHGTDTGSAEVQVAMLSERISKLSNHLQKNIHDFSSRQGLLKMIGQRKRLLNYL

RDKSNKRYTDIITKLKLRG

>gi|159888193|gb|ABX08407.1| Ribosomal protein L19 [Prochlorococcus marinus str. MIT 9211]

MTADLQNTSSTEESSSGGLTSPPESDSPKEKKPSSSSGLKTHNQKGETKDLIKEFELSQQKDKVPEVYVG

DTVKVGVRISEGNKERVQPYEGVVIAKRHGGINQTITVRRIFQGVGVERVFMVHSPQVASIKVERRGKVR

RAKLFYLRERVGKATRVKQRFDR

>gi|159889388|gb|ABX09602.1| 50S ribosomal protein L16 [Prochlorococcus marinus str. MIT 9211]

MLSPKRTKFRKQQRGRMRGVATRGNKIAFGQFALQAQECGWITSRQIEASRRAMTRYVKRGGQIWIRIFP

DKPVTMRPAETRMGSGKGNPEFWVAVIKPGRILFEMGGEEITESIAREAMRLAQYKLPIKTKFIALAEGE

TPTQVGKASSASLANLDEDANSQTDDETSSSGSVATVES

>gi|159889387|gb|ABX09601.1| 50S ribosomal protein L29 [Prochlorococcus marinus str. MIT 9211]

MARPEISEVTKLTDDDLKNKIDEIRKELFDLRFKRATRQLSETHRFKEARIQLAQLLTVQGDRNRSKTSS

>gi|159889375|gb|ABX09589.1| 50S Ribosomal protein L36 [Prochlorococcus marinus str. MIT 9211]

MKVRASVKKMCEKCRVIRRHGRVMVICTATQKHKQRQG

>gi|159888987|gb|ABX09201.1| 50S ribosomal protein L34 [Prochlorococcus marinus str. MIT 9211]

MTKRTLGGTSRKRKRVSGFRVRMRTHTGRRVIRARRKKGRSQLAV

>gi|159888624|gb|ABX08838.1| 50S ribosomal protein L32 [Prochlorococcus marinus str. MIT 9211]

MAVPKKKTSKGKRNQRHSIWKAKAGIAAQKALSLGKSVLTGRSQGFVYPIDEQEEAEE

>gi|159889438|gb|ABX09652.1| Predicted hydrolase or acyltransferases (alpha/beta hydrolase superfamily) [Prochlorococcus marinus str. MIT 9211]

MTDLAAKPNEPWSYLGHKVFSVSSSPSGQVSDVNQNNPVVLLVHGFGASTEHWRHNIPVLSRSHEVHAID

LLGFGRSAKPSELEYGGELWKEQVVAYVKERIGKPTVIVGNSLGGYAALAAGAALESKSAGVVLLNAAGY

FSDETLVKQPTDFFSRLRQFIGLGLSRDLLIKWFLYPLMQRLIFENLRRPNVIRNTLKQVYIDPTNVDDY

LIESIRRPSLDPGAFQVFRKVFQARGLKGKPIDELFNELEAPLLLLWGDSDPWLRNAKAKQEKFLLFARE

ASLEVKEVLLRAGHCPHDEIPDRVNEEMLAWLKG

>gi|159888995|gb|ABX09209.1| polyribonucleotide nucleotidyltransferase [Prochlorococcus marinus str. MIT 9211]

MQGQTTTVSFDGREIRLTTGRYAPQAGGSVLIECGDTAVLVTATQSPGREGADFLPLICDYEERLYAAGR

IPGSFMRREGRPPERATLISRLIDRPLRPLFPSWMRDDIQVVATCLSLDERVPSDVLAVTASSMATLLAE

IPFYGPMAAVRVGLLGDDFVLNPSFREIERGDLDLVVAGTPDGVVMIEAGANQLSEQDVIEAVDFGYEAV

TELIKAQQSILKESGIDHKKPDEQEIDETLPNYLDKNGRKPIGELLKKFELTKKERDLKLEEIKTNLGEK

IDSLKEDNAVKKAISSNPKLLTTSFKSLTKKLMREQIIKDGKRVDGRALDEVRKIEAAAGILPKRVHGSG

LFQRGLTQVLSTATLGTPSDAQEMDDLNPSSDKTYIHHYNFPPYSVGETRPMRTPGRREVGHGALAERAL

IPVLPPKESFPYVLRVVSEVLSSNGSTSMGSVCGSTIALLDAGVPLKAPVSGAAMGLIKEGKEIRILTDI

QGIEDFLGDMDFKVAGTEKGITALQMDMKVTGLEVKTIADAINQAKPARTHILEKMNETIDKPRETLSPH

APRLLSFRIDPELIGTVIGPGGRTIKGITERTNTKIDIEDGGIVTIASHDGVAAEEAQKIIEGLTRKVHE

GEIFTGSITRIIPIGAFVEILPGKEGMIHISQLSEARVEKVEDVVKVGDEVTVRVREIDNRGRINLTLRG

VSQNNNDMNYPQPTPTPVAPLN

>gi|159888814|gb|ABX09028.1| putative ribosomal-protein-alanine acetyltransferase [Prochlorococcus marinus str. MIT 9211]

MRLNDIALNGLWSKSQWEYELKSDYRHCLGVSEQESLIAISCGWIVANQLDITAVAVDPRYRRLGLGSKI

LCALINHAKSKGATNATLEVNEENIDGINFYRSLGFLQVGYRKNYYKDLSSAILFSLYIDQ

>gi|253784624|emb|CAZ76988.1| unnamed protein product [Prochlorococcus marinus str. MIT 9211]

MATPAKKTGSKKSKRNVPNGVVHIQSTFNNTIVSITDTNGEVVSWSSAGASGFKGARKGTPFAAQTAAEA

AARRALEQGMRQIEVLVRGPGSGRETAIRALQVAGLEITLIRDVTPLPHNGCRRPKRRRV

>gi|159888897|gb|ABX09111.1| Hypothetical protein P9211_11801 [Prochlorococcus marinus str. MIT 9211]

MTQVTVGENEGIESALRRFKRQVSKAGIFGELKRLRHHETPVEKYKRKLQQRRRNRRR

>gi|159888227|gb|ABX08441.1| Hypothetical protein P9211_05101 [Prochlorococcus marinus str. MIT 9211]

MFNGDVASNDSQPLDITADLKDYKTGMTNSDSLIGIDEVQKALNRSRASVYRYTNTDTRNLNPPFNPRKL

NPEYRSDQKDPLLFHPNEVARFAKDILRIKEVTVEVLNSPSTETQNVLASILEELRLIRIQLEGTSPASE

EFLTNRERKDWPAA

>gi|159887833|gb|ABX08047.1| possible Fe-S oxidoreductase [Prochlorococcus marinus str. MIT 9211]

MHTSVFKEPKNNDAVLSNHCNASVAFLHLGCEKNLVDTEHMMGLLASEGYGVSSNTDDAEVVVVNTCSFI

EQAREESVRALVGLADQGKEIIIAGCLAQHFKSELLESIPEAKAIVGTGDYQNIIEVLQRVRQGERVNQV

SENPKFVGDENLPRYRTTGRFVSYLKVAEGCNYRCAFCIIPTLRGNQRSRSVQSIVNEANQLAKEGIQEL

ILISQITTNYGMDLYGRPYLADLLRALSHVDIPWIRIHYAYPTGLTPEVVLAYKEVPNVLPYFDLPLQHS

HPDVLRAMNRPWQSDVSSALLNRIKEQLPEAVMRTTLIVGFPGETKAQFDHLCAFVENQKFDHVGVFAFS

REEGTEAAKLPNQVPFEIAQARKDKLVAIQQPISAAKNQALIGQTVDVLIEREDLATGELIGRSARFAPE

VDGEVRLRPSQVLFNDLHGKIVPALITGSELYDLTGEINHLN

>gi|159887792|gb|ABX08006.1| Hypothetical protein P9211_00751 [Prochlorococcus marinus str. MIT 9211]

MSQITVGENEGIESAIRRFKRQVSKAGIFLEIKRLRHHETPFEKYKRKQIQRKRSR

>gi|226734605|sp|A9BDB8.1|RL9_PROM4 RecName: Full=50S ribosomal protein L9

MAKRVQLVLKEDILSLGKDGDVVEVAPGYARNFLLPHGKALPVTPAVMKQVEHRRAKQKEHEAALKDEAL

AFETALKTIGRFTVKKQVGDDGVLFGTVTNGDIAEAIEKATEKEIDRRTISVPEVHAIGQYKVQIKLHHE

VTAEINLEVVSY

>gi|226733578|sp|A9BCN3.1|RL6_PROM4 RecName: Full=50S ribosomal protein L6

MSRIGKQPIPVPEKVAVELDGLSLTVKGPKGELSRTLPEGVSISQVDNSIVVSAINSKRKSRERHGLSRS

LVANMVEGVSKGYSKKLEIVGVGSRAQVKGKNLIVSAGYSHPVEVIPPDGITFVVENNTNVTVSGIDKEL

VGNEAAKIRAIRPPEPYKGKGIKYAGERIIRKAGKSGKK

>gi|226702759|sp|A9BDG7.1|RL11_PROM4 RecName: Full=50S ribosomal protein L11

MAKKIVAVIKLALQAGKANPAPPVGPALGQHGVNIMAFCKEYNAKTQDKAGFVIPVEISVFEDRSFTFIT

KTPPASVLITKAAGIEKGSGDSAKGQVGTINRAQLEEIAKTKLPDLNCSNIESAMRVIEGTARNMGVSVK

D

>gi|226699986|sp|A9BDG5.1|RL10_PROM4 RecName: Full=50S ribosomal protein L10

MGRTLESKKQIVEELKALLDQAEMALVIDYQGLTIKEMSDLRTRLGPSSGICKVTKNTLMRKAIDGDTSW

SSLESLLNGTNAFVLVKGDVGGALKAVQAFQKETKKSKTKGGLFEGKLLSQDEIKAIANLPTKEVLMAQI

AGALNSIATKMAVGINEVPSGLARSLKQHADSGEN

>gi|226733721|sp|A9BDG4.1|RL7_PROM4 RecName: Full=50S ribosomal protein L7/L12

MSKKTDEILDSLKSLSLLEASELVKQIEEAFGVSAAASAGVVMAAPGAGGGASAAGEAAEEKTEFDVILE

SFDAAAKIKVLKEVRNATGLGLGEAKAMVEAAPKTIKEGASKEDAEALKKAIEAVGGKVTLK

>gi|226731314|sp|A9BCN5.1|RL5_PROM4 RecName: Full=50S ribosomal protein L5

MSLKHRYRETIRPKLLKDLGLSNIHQVPKVVKVNVNRGLGEAAQNSKTLEASLSEMATITGQKALVTRAK

KAIAGFKIREGMPIGCTVTLRGERMYAFLERLINLALPRIRDFRGVSPKSFDGRGNYTLGVKEQLIFPEI

SFDKIDTIRGMDITIVTSARSDEEGRALLKALGFPFRST

>gi|226724953|sp|A9BDG6.1|RL1_PROM4 RecName: Full=50S ribosomal protein L1

MTKISKRMASLSSKIEDRAYPPLEAINLVKESSTAKFDETIEAHVRLGIDPKYTDQQIRTTVTLPNGTGQ

TIRIAVIARGEKVAEAKSAGADLAGEEELVDSISKGEMGFDLLIATPDMMPKVAKLGRVLGPRGLMPNPK

TGTVTADLVGAIKEFKAGKLEFRADKAGIIHVRFGKASFNADALLENLKTLQETIDRNKPSGAKGRFWKT

LYITSTMGPSIEVDIAALQDISQE

>gi|226699105|sp|A9BE29.1|RS4_PROM4 RecName: Full=30S ribosomal protein S4

MSRYRGPRLRITRRLGDLPGLTRKAAKRSNPPGQHGQARRKRSEYAIRLEEKQKLRFNYGISERQLVRYV

KKARSLEGSTGTNLLKLLENRLDNVCFRLGFGPTIPGSRQLVNHGHVTVNGKVLDIASYQCKPGDVISIR

EKKGSKKLAEGNLEFPGLANVPPHLEFEKSKMSAKITGKCEREWVAIEINELLVVEYYSRKV

>gi|226697746|sp|A9BCP1.1|RS3_PROM4 RecName: Full=30S ribosomal protein S3

MGHKIHPNGLRLGITQEHRSRWYASSKTYPTLLQEDDRIRGFIQKKYASAGISDVLIARKADQLEVELKT

ARPGVIVGRQGSGIEELRSGIQKTIGDRSRQVRINVVEIERVDADAHLLAEYIAQQLEKRVAFRRTIRMA

VQRAQRAGVLGLKIQVGGRLNGAEIARSEWTREGRVPLHTLRAEIDYANKTANTTYGVLGIKVWVFKGEV

LSKEDQPLPVGASPRRKGSRRPQQFEDRSNDGK

>gi|215274854|sp|A9BCP2.1|RL22_PROM4 RecName: Full=50S ribosomal protein L22

MVESSSSKTKFAQAHGRYIRGSASKVRRVLDQIRGRTYRDALIMLEFMPYRSTGPITKVLRSAVANAENN

MGLDPASLVITRATADMAPSMKRYRPRAQGRAFAIKKQTCHISISVAPSSESTNSEASD

>gi|226712552|sp|A9BBI9.1|RL34_PROM4 RecName: Full=50S ribosomal protein L34

MTKRTLGGTSRKRKRVSGFRVRMRTHTGRRVIRARRKKGRSQLAV

>gi|226708152|sp|A9BCL9.1|RL31_PROM4 RecName: Full=50S ribosomal protein L31

MPKEDIHPTWYPDAKVICNGEVVMTTGSTQPEIHVDVWSGNHPFFTGTQKILDTEGRVDRFMRKYGMADS

ENDSTDKKKTTNEKKVSDSPSKES

>gi|238687107|sp|A9BAS9.1|RS2_PROM4 RecName: Full=30S ribosomal protein S2

MAVVTLSEMMEAGAHFGHQTRRWNPKMSRYIYCARNGVHIIDLVKTAVCMNSAYKWTRNAAKSGKRFLFV

GTKKQASEVVALEANRCGASYVNQRWLGGMLTNWTTMKARIDRLKDLERMESSGAIAMRPKKEASVLRHE

LERLQKYLGGLKGMKRLPDVVVLVDQRRETNAVLEARKLDIPLVSMLDTNCDPDLCEVPIPCNDDAVRSV

QLILGRLADAINEGRHGSNEHRGGQRLK

>gi|229485531|sp|A9BAG3.1|RL33_PROM4 RecName: Full=50S ribosomal protein L33

MAKKGTRVVVTLECTECRTVPPSEKRSPGVSRYTTEKNRRNTTERLELKKFCPQLNKMTIHKEIK

>gi|226737970|sp|A9BBW8.1|RL27_PROM4 RecName: Full=50S ribosomal protein L27

MAHKKGTGSTRNGRDSNSKRLGVKAYGGEPVTAGSILIRQRGTSVLPGINVGKGKDDTLFALTDGIVTFE

TIRRGLKNRKRISVALTS

>gi|226735232|sp|A9BCP3.1|RS19_PROM4 RecName: Full=30S ribosomal protein S19

MGRSLKKGPFIADSLLKKVEKQNSNDDRSVIKTWSRASTILPVMIGHTIAVHNGKSHIPVFITEQMVGHK

LGEFAPTRTYKGHIKDKKGAR

>gi|226733827|sp|A9B9X0.1|RS15_PROM4 RecName: Full=30S ribosomal protein S15

MTLNTQEKQKLINTHQNHGTDTGSAEVQVAMLSERISKLSNHLQKNIHDFSSRQGLLKMIGQRKRLLNYL

RDKSNKRYTDIITKLKLRG

>gi|226731534|sp|A9BBJ6.1|RS14_PROM4 RecName: Full=30S ribosomal protein S14

MAKKSMIARDVKRKKLVERYATKRKKLLDEFNSAKDPMERLEIHRKIQALPRNSAPSRMRNRCWATGKPR

GVYRDFGLCRNQLRERAHKGELPGVVKSSW

>gi|226730707|sp|A9BCP6.1|RL4_PROM4 RecName: Full=50S ribosomal protein L4

MANCVVLDWQGQEAGKATLDLKVAKETTAVDLIHRAVVRQQAHSRQGTASTLTRSEVRGGGRKPYKQKGT

GRARQGSIRTPLRPGGGIIFGPKPRSFSLGMNRKERRLALRTALMARINDLMIVQDFGTALKVPKTREIV

EALSRFGIADDAKVLIILAQPSEIIIRSVRNIERVKVIGADQLNVFDLLNANSLVIGEKALSTIKEVYGD

D

>gi|226730549|sp|A9BCP7.1|RL3_PROM4 RecName: Full=50S ribosomal protein L3

MSIGILGKKLGMSQFFDDKGRSVPVTLIEAGPCRITQLKSLATDGYSAVQIGYGFTREKLVNKPFKGHLS

KSGEGFLRHLHEYRVDDLGVYELGAKITVGNFEAGQKVDVSGNSMGRGFAGYQKRHSFSRGPMSHGSKNH

RQPGSTGAGTTPGRIYPGKRMAGRYGGKKITTRGLTIMKIDSDRNLLVVKGSVPGKPGSLLNIRPAQRVG

SQNLTGGKK

>gi|226730436|sp|A9BDA6.1|RL20_PROM4 RecName: Full=50S ribosomal protein L20

MSRVKRGNVARKRRNKILRIAKGYRGGNGKLFRTANQRVMKALCNAYRDRKRRKRDFRRLWIARINAAAR

LNGMSYSKLMGNLKKADVRINRKMLAQLAVIDPKSFKNVVTHSQK

>gi|226725049|sp|A9BDA5.1|RL35_PROM4 RecName: Full=50S ribosomal protein L35

MPKLKTRKAAAKRFKVTGTGKFMRRRAFRNHLLDHKSTKLKRHLATKAVVDERDADNVSLMLPYS

>gi|226723362|sp|A9BCN2.1|RL18_PROM4 RecName: Full=50S ribosomal protein L18

MATLSKKQQTQKRHKRLRRHLNGTNHRPRLAVFRSNNHIYAQVIDDEAQSTICSASTLDKDLREKLKASG

GSCDASMAVGALLAQRALAKGIEQVVFDRGGNLYHGRVKALAKSAREAGLKF

>gi|226712822|sp|A9BCM0.1|RS9_PROM4 RecName: Full=30S ribosomal protein S9

MNSSSQRNTVVYWGTGRRKTSVARVRLIPGTGKITINGRPGDHYLNFNPAYLAAVKAPLQTLGLSDSYDV

LVNVYGGGLTGQADAIKQGAARALCELSADNRKPLKIEGHLSRDPRAKERRKYGLKKARKAPQFSKR

>gi|226712777|sp|A9BCM6.1|RS13_PROM4 RecName: Full=30S ribosomal protein S13

MARIAGIDIPREKRVEVALTYIYGVGLTRAKSILAKAGVNPDIRVKDLDDGDVQKLRTAVESFTLEGDLR

RQEGMALKRLQDIGCLRGRRHRMSLPVRGQRTRTNARTRRGSRKTVAGRKK

>gi|226712634|sp|A9BCK3.1|RS12_PROM4 RecName: Full=30S ribosomal protein S12

MPTIQQLIRTERKRLTRKTKSPALRSCPERRGVCTRVYTSTPKKPNSALRKVARVRLTSGFEVTAYIPGI

GHNLQEHSVVLLRGGRVKDLPGVRYHIIRGTLDTAGVKDRRQSRSKYGAKVPK

>gi|226712230|sp|A9BAH6.1|RL32_PROM4 RecName: Full=50S ribosomal protein L32

MAVPKKKTSKGKRNQRHSIWKAKAGIAAQKALSLGKSVLTGRSQGFVYPIDEQEEAEE

>gi|226712195|sp|A9BCM3.1|RL17_PROM4 RecName: Full=50S ribosomal protein L17

MRHQLRVPKLGRPADQRKAILRGLTTQLIREGRVTTTKAKAKALRNEAERMITLAKEGTLAARRRAIGYI

YDKKLVHQLFEKAQDRYGDREGGYTRIVRTVPRRGDNAEMAIIELV

>gi|226708566|sp|A9BCN4.1|RS8_PROM4 RecName: Full=30S ribosomal protein S8

MANHDPISDMLTRIRNASEKRHETTRIPASRMSRSIAKVLQKEGFIAQISEEGEGFKTQLVLELKYSGKH

RHPTIRSMQRVSKPGLRIYKNTRGLPKILGGLGVAIISTSKGVMSDRDARKQGVGGEVLCYVY

>gi|226708401|sp|A9BCM5.1|RS11_PROM4 RecName: Full=30S ribosomal protein S11

MATPAKKTGSKKSKRNVPNGVVHIQSTFNNTIVSITDTNGEVVSWSSAGASGFKGARKGTPFAAQTAAEA

AARRALEQGMRQIEVLVRGPGSGRETAIRALQVAGLEITLIRDVTPLPHNGCRRPKRRRV

>gi|226705608|sp|A9BCJ9.1|RS10_PROM4 RecName: Full=30S ribosomal protein S10

MSTAIAQQKIRIRLKAFDRRMLDLSCDKIIETADNTAATAIGPIPLPTKRKIYCVLRSPHVDKDSREHFE

TRTHRRIIDIYNPSAKTIDALMKLDLPSGVDIEVKL

>gi|226705539|sp|A9BCN7.1|RL14_PROM4 RecName: Full=50S ribosomal protein L14

MIQQETFLTVADNSGAKKLQCIRVLGSNRRYAHVGDVIVAAVKDALPNMGVKKSDVVKAVVVRTKATLRR

ETGNSIRFDDNAAVLINEDKNPRGTRVFGPVARELRERNFTKIVSLAPEVI

>gi|226702980|sp|A9BCP4.1|RL2_PROM4 RecName: Full=50S ribosomal protein L2

MAIRTFRPYTPGTRTRVVTDFNELTGRKPERSLVVSKHRLKGRNNRGVITCRHRGGGHKRLYRIVDFRRN

KHDVPAKVAAIHYDPHRNARLALLFYSDGEKRYILAPAGIAIGQEVISGPKVPIETGNAMPLSAIPLGSS

VHCVELYAGRGGQMVRSAGASAQVMAKEGDYVALRLPSTEVRLIRRECYATLGEVGNSEIRNTSLGKAGR

RRWLGRRPQVRGSVMNPCDHPHGGGEGRAPVGRAGPVTPWGKPALGLKTRKRNKPSNRFVLRKRRRVSKR

SRGGRDS

>gi|226699147|sp|A9BAC7.1|RL28_PROM4 RecName: Full=50S ribosomal protein L28

MSRVCDLSGTRANNGMAVSHSHIRTKKLQQANLQQRKLWWEEGKKWIKVRVTTRTLKTIQKKGLNSYAKA

MGIDLSKV

>gi|226697874|sp|A9BBQ1.1|RS16_PROM4 RecName: Full=30S ribosomal protein S16

MIKLRLKRYGKKREASFRLVACNSTSRRDGRPLEELGFYNPRTKETRLDTEALRTRLSQGAQPTDAVRSL

LEKGGLIEKTIRPAEIEGKKKQALARQSASKKAVKEKTEESKGSEVDSETSTSAD

>gi|226695402|sp|A9BCH8.1|RS20_PROM4 RecName: Full=30S ribosomal protein S20

MANNNSAKKRIQIAERNRLQNRSYKSAMRTLMKRCLTAAGSYLEKPGEEAKANLQQNINEAFSKIDKAVK

KGVLHRNNGANKKSRLNAAVKKLIEPATKR

>gi|226695114|sp|A9BDR2.1|RS6_PROM4 RecName: Full=30S ribosomal protein S6

MTNQPYYETMYILRPTIPEDEVDSHLKKYTEILESAGGEVLDSQMRGKRRLAYPIGKHKEGIYVQLSHQG

DGQHIAVLEKAMRLTEDVIRYLTVKQDGPLPAKRVVKTSEKNVKEDKEVENKETTTEDKDQKGDLKETKK

SENKDSVTEAEGQKDIKEAKEIENKEIEKKED

>gi|205831058|sp|A9BCM7.1|RL36_PROM4 RecName: Full=50S ribosomal protein L36

MKVRASVKKMCEKCRVIRRHGRVMVICTATQKHKQRQG

>gi|205806698|sp|A9BCK2.1|RS7_PROM4 RecName: Full=30S ribosomal protein S7

MSRRNAAEKRPVLPDPQFNNRLATMMVARLMKHGKKSTAQRILSDAFGLINERTGSDPIELFETAVKNAT

PLVEVRARRVGGATYQVPMEVRQERGTAMALRWLVNFSRSRNGRSMAHKLAGELMDAANEAGNAVRKREE

THKMAEANKAFAHYRY

>gi|226712337|sp|A9BCP0.1|RL16_PROM4 RecName: Full=50S ribosomal protein L16

MLSPKRTKFRKQQRGRMRGVATRGNKIAFGQFALQAQECGWITSRQIEASRRAMTRYVKRGGQIWIRIFP

DKPVTMRPAETRMGSGKGNPEFWVAVIKPGRILFEMGGEEITESIAREAMRLAQYKLPIKTKFIALAEGE

TPTQVGKASSASLANLDEDANSQTDDETSSSGSVATVES

>gi|226699273|sp|A9BCN9.1|RL29_PROM4 RecName: Full=50S ribosomal protein L29

MARPEISEVTKLTDDDLKNKIDEIRKELFDLRFKRATRQLSETHRFKEARIQLAQLLTVQGDRNRSKTSS

>gi|238066463|sp|A9BCV9.1|RIMO_PROM4 RecName: Full=Ribosomal protein S12 methylthiotransferase RimO; Short=S12 MTTase; Short=S12 methylthiotransferase; AltName: Full=Ribosomal protein S12 (aspartate-C(3))-methylthiotransferase; AltName: Full=Ribosome maturation factor RimO

MHTSVFKEPKNNDAVLSNHCNASVAFLHLGCEKNLVDTEHMMGLLASEGYGVSSNTDDAEVVVVNTCSFI

EQAREESVRALVGLADQGKEIIIAGCLAQHFKSELLESIPEAKAIVGTGDYQNIIEVLQRVRQGERVNQV

SENPKFVGDENLPRYRTTGRFVSYLKVAEGCNYRCAFCIIPTLRGNQRSRSVQSIVNEANQLAKEGIQEL

ILISQITTNYGMDLYGRPYLADLLRALSHVDIPWIRIHYAYPTGLTPEVVLAYKEVPNVLPYFDLPLQHS

HPDVLRAMNRPWQSDVSSALLNRIKEQLPEAVMRTTLIVGFPGETKAQFDHLCAFVENQKFDHVGVFAFS

REEGTEAAKLPNQVPFEIAQARKDKLVAIQQPISAAKNQALIGQTVDVLIEREDLATGELIGRSARFAPE

VDGEVRLRPSQVLFNDLHGKIVPALITGSELYDLTGEINHLN

>gi|238687125|sp|A9BD47.1|RIMM_PROM4 RecName: Full=Ribosome maturation factor RimM

MSRKDSWLTIGKLVGAQGLRGEVKVNPSSDFPERFINPGERWLQKNTEEPSRIELKSGRQLPGKSIYIVS

FIGITDRNKAESIVGNKLLVPSDQKPKLKEGEFHLVDLLGLKAKFTQDGSDVGEVIDLTSAGNDLLVIKL

VEGKTVLIPFVKEIVPVINLKQGWLLIKPPPGLLEL

>gi|226702676|sp|A9BBJ7.1|PNP_PROM4 RecName: Full=Polyribonucleotide nucleotidyltransferase; AltName: Full=Polynucleotide phosphorylase; Short=PNPase

MQGQTTTVSFDGREIRLTTGRYAPQAGGSVLIECGDTAVLVTATQSPGREGADFLPLICDYEERLYAAGR

IPGSFMRREGRPPERATLISRLIDRPLRPLFPSWMRDDIQVVATCLSLDERVPSDVLAVTASSMATLLAE

IPFYGPMAAVRVGLLGDDFVLNPSFREIERGDLDLVVAGTPDGVVMIEAGANQLSEQDVIEAVDFGYEAV

TELIKAQQSILKESGIDHKKPDEQEIDETLPNYLDKNGRKPIGELLKKFELTKKERDLKLEEIKTNLGEK

IDSLKEDNAVKKAISSNPKLLTTSFKSLTKKLMREQIIKDGKRVDGRALDEVRKIEAAAGILPKRVHGSG

LFQRGLTQVLSTATLGTPSDAQEMDDLNPSSDKTYIHHYNFPPYSVGETRPMRTPGRREVGHGALAERAL

IPVLPPKESFPYVLRVVSEVLSSNGSTSMGSVCGSTIALLDAGVPLKAPVSGAAMGLIKEGKEIRILTDI

QGIEDFLGDMDFKVAGTEKGITALQMDMKVTGLEVKTIADAINQAKPARTHILEKMNETIDKPRETLSPH

APRLLSFRIDPELIGTVIGPGGRTIKGITERTNTKIDIEDGGIVTIASHDGVAAEEAQKIIEGLTRKVHE

GEIFTGSITRIIPIGAFVEILPGKEGMIHISQLSEARVEKVEDVVKVGDEVTVRVREIDNRGRINLTLRG

VSQNNNDMNYPQPTPTPVAPLN

>gi|157387249|gb|ABV49954.1| Ribosomal protein S1 [Prochlorococcus marinus str. MIT 9215]

MNENSSQTIKELSEDQEIKNSSDLDNDSASKNEEDLSFENSDIPSADSSSSRTNTDFDNAGFTQEEFASL

LGKYDYNFKPGDLVKGTVFALEPKGAMIDIGAKTAAFMPVQEVSINRVEGLNDVLQPSESREFFIMSEEN

EDGQLALSIRRIEYQRAWERVRQLQKEDATIYSEVFATNRGGALVRVEGLRGFIPGSHISARKIKDDLEG

EYLPLKFLEVDEERNRLVLSHRRALVEKKMNRLEVGEVVVGSVKGIKPYGAFIDIGGVSGLLHISEISHE

HIETPHNVLNVNDQMKVMIIDLDSERGRISLSTKALEPEPGDMLTDPQKVFSKAEEMAAKYKQMLFEQTD

ENEDMPTATAETV

>gi|157387521|gb|ABV50226.1| Ribosomal protein S1 [Prochlorococcus marinus str. MIT 9215]

MGVSNKNAQDNIQPKGNKKDFKKPLQVLHISKKDTQKIKNEQINSQEEIKKENISIKPQLIKDGPVKEIE

DSIENTKAFDIAQQDLNRPLNFSEQNTDFQLERTVDEFDFDESAFLEALNANEPIGATGETISGKVIAIE

SDGLYVDIGGKAPGYMPKKECGLGIITNFKEKFSIGLEMEVLVIKEQNADGMVTVSARALILRQSWEKVS

SSAKNGELINVLINGFNRGGLTCDVDGLRGFIPRSQLEDGQDYQSFVGKTLKVAFLEVNPESRKLVLSEK

KASLVSKLTNLELGQLIEGEVLAVKPYGFFIDLGGASGLLHQSSLTNGSIRSLREVFREGEMIKALISEI

DLEKGRIGLNTALLENSAGELIIDKQKVMQEATERALKTKALFDKKEQDK

>gi|157388854|gb|ABV51559.1| 50S ribosomal protein L9 [Prochlorococcus marinus str. MIT 9215]

MAKRVQLALTESIASLGKEGDLVEVAPGYARNFLLPYGKAMNVTPAVLKQIERKKEKEKIAADKLKQEAL

DFQTALSTIGRFTIKKQVGEDGVLFGTVTNGDVAEAIEEATKKEIDRRNITVPDIHNLGSFTAKIKLHPE

VNAEVNIEVTS

>gi|157388723|gb|ABV51428.1| 50S ribosomal protein L6 [Prochlorococcus marinus str. MIT 9215]

MSRIGKTPVLIPDKVTVDFDGLIVTVKGPKGELKRQMPEGVCFDKKDNTVVVSPTTSKIFSRQRHGLCRA

LIANMVEGVSQGFSKKLEIVGVGSRAQVKGKNLVVSAGYSHPIEMIPPDGITYKVESNTNVTVSGIDKEI

VGNEAAKIRSIRPPEPYKGKGIKYHDEIILRKAGKSGKK

>gi|157388721|gb|ABV51426.1| 30S ribosomal protein S5 [Prochlorococcus marinus str. MIT 9215]

MTDTPTKQETQSNKDNVPGAIPVEQKKNNRNDRKRNRRGDSKNLERDSDWQERVVQIRRVSKTVKGGKKM

SFRAIVVVGNEKGQVGVGVGKAGDVIGAVRKGVSDGKKNLVRVPLTPNNSIPTLSKGRDGAANVLIRPAA

PGTGVIAGGSIRTVLELAGIKNVLAKRLGSKTPLNNARAAMVALSQLRTHKSASRERGISLEQLYS

>gi|157387137|gb|ABV49842.1| 50S ribosomal protein L11 [Prochlorococcus marinus str. MIT 9215]

MAKKIVAVIKLALQAGKANPAPPVGPALGQHGVNIMAFCKEYNARTQDKAGFVIPVEISVFEDRSFTFIT

KTPPASVLITKAAGIEKGSGESAKGSVGNISKAQLEEIAKTKLPDLNCSSVESAMKVIEGTARNMGVSIT

D

>gi|157387135|gb|ABV49840.1| 50S ribosomal protein L10 [Prochlorococcus marinus str. MIT 9215]

MGRTLENKQQIVTEIKSLLNDSEMAVVLDYKGLTIKEMSDLRSRLRTTNGICRVTKNSLMRKAIDGDSNW

NDLESLLTGTNAFVLIKEDVGGAVKAIQSFQKDTKKSETKGALFEGRLLSDSEIKEIASLPSKEVLMAKI

AGALNGVATKIAISINEVPSGLARSLKQHSEKSES

>gi|157388733|gb|ABV51438.1| 50S ribosomal protein L22 [Prochlorococcus marinus str. MIT 9215]

MTKTSETTKIAIAHGNYIRGSASKVRRVLDQIRGRSYRDALIMLEFMPYRSTDPITKVLRSAVANAEHNL

GMDPSTLIISSAWANSGPVMKRYRPRAQGRAFSIKKQTCHISISVESAPNKTNAEVQN

>gi|157388732|gb|ABV51437.1| 30S ribosomal protein S3 [Prochlorococcus marinus str. MIT 9215]

MGHKIHPSGLRLGITQEHRSKWFATSKTYPILLQEDFKIRTFIEKKYGAAGISDVLIARKADQLELELKT

ARPGVIVGRQGSGIEELRSGIQKTIGDRTRQVRINVVEVERVDADAFLLAEYIAQQLEKRVAFRRTIRMA

LQRAQRAGVLGLKIQVGGRLNGAEIARTEWTREGRVPLHTLRAEIDYATREANTTYGVLGIKVWVFKGEV

LPKEEQTIPVGANPKRKASRRPQQFEDRSNENS

>gi|157388725|gb|ABV51430.1| 50S ribosomal protein L5 [Prochlorococcus marinus str. MIT 9215]

MTLKNRYKESIRPKLLKELGLKNIHQVPKVVKVNVNRGLGEAASNSKALEASLNEMATITGQKALVTRAK

KAIAGFKIREGMPIGCTVTLRGDRMYSFLERFINLALPRIRDFRGVNPKSFDGRGNYTVGVKEQLIFPEI

SFDKIDSIRGMDITIVTSARSDQEGKALLQELGMPFSKN

>gi|157387397|gb|ABV50102.1| 30S ribosomal protein S4 [Prochlorococcus marinus str. MIT 9215]

MSRYRGPRLRVTRRLGELPGLTRKASKKSNPPGQHGQARRKRSEYAIRLEEKQKLRFNYGVSEKQLVRYV

KKARAQEGSTGTNLLRLLENRLDNVCFRLGFGGTIPGSRQLVNHGHVTVNGKVLDIAGYQCKSGDVIGIK

ENKASKKLVEGNIEFPGLANVPPHLDLDKPKLTGKINGKCDREWVALEINELLVVEYYSRKV

>gi|157387136|gb|ABV49841.1| 50S ribosomal protein L1 [Prochlorococcus marinus str. MIT 9215]

MKKLSKRMASLSTKIEDRIYAPLEALSIIKENANAKFDETIEAHIRLGIDPKYTDQQLRTTVALPHGTGQ

SIRIAVITSGENVSKAKSAGADLFGEEDLVESINKGNMEFDLLIATPDMMPKVAKLGRVLGPRGLMPNPK

AGTVTNDIGNAIKEFKAGKLEFRADKAGIVHVRFGKASFTKEALFENLKTLQESIDKNKPSGAKGKYWKS

FYVTSTMGPSVQVDINAVQDYQAEG

>gi|157387134|gb|ABV49839.1| 50S ribosomal protein L7/L12 [Prochlorococcus marinus str. MIT 9215]

MSAKTEEILESLKSLSLLEASELVKQIEEAFGVSAAASAGVVMAAPGAAGGDGDDGAAEEKTEFDVVLES

FDAAAKIKVLKVVRNATGLGLGDAKTLVESAPKTVKEGIAKADAESLKKEIEEAGGKVTLK

>gi|157388887|gb|ABV51592.1| 30S ribosomal protein S6 [Prochlorococcus marinus str. MIT 9215]

MSETMNDQQSYYETMYILRPDIAEDEVTNHIDKYNKLLEEFGGSILDSQMRGKRRLAYQIAKHREGIYVQ

LSHQGDGQHIFKIEKAMRLSEDVIRYMTVKQEGPLPTPRPSTKSSTQADDKKNQETKVESKGEQPVVSTD

ASTSGKDDTETKENAEP

>gi|157388843|gb|ABV51548.1| 50S ribosomal protein L20 [Prochlorococcus marinus str. MIT 9215]

MARVKRGNIARKRRNKILNLAKGFRGGNKNLFRTANQRVMKALCNAYRDRRRRKRDFRRLWISRINASAR

INGTNYSKLINGMKNSEIIINRKMLAQLALSDPKCFEKIVSSVSN

>gi|157388842|gb|ABV51547.1| 50S ribosomal protein L35 [Prochlorococcus marinus str. MIT 9215]

MSKLKTRKSAAKRFKATATGKFMRRRAFHNHLLDHKSSKLKRHLSTKAVVDERDADNVRLMIPYA

>gi|157388738|gb|ABV51443.1| 50S ribosomal protein L3 [Prochlorococcus marinus str. MIT 9215]

MSIGILGKKLGMSQLFDDKGNAVPVTLIEAGPCRVTQLKTNALDGYTAIQIGYGVSKEKHISKPEKGHLL

KSGEELLKHLKEYRVEETSSYEIGNQITVKNFEVGQKVDISGKSMGRGFSGYQKRHGFSRGPMSHGSKNH

RAPGSTGAGTTPGRIYPGKRMAGRYGGKQITTKGLLVLKIDDQKNLLVVKGSVPGKPGSIVNIKPNNVVG

KKGGEKS

>gi|157388737|gb|ABV51442.1| 50S ribosomal protein L4 [Prochlorococcus marinus str. MIT 9215]

MTTLETLKWDGKKSGKVTLDLTVAKETSSADLIHRAVLRQLANKRQGTASTLTRSEVRGGGRKPYKQKGT

GRARQGSIRTPLRPGGGIIFGPKPRSYNLDMNRKERRLALRTALMSRVSDMKAVEDFGSTLKQPKTSDII

NGLARLGIQKNEKVLVILDSPSDVIKKSINNIEKVKLIAADQLNVFDILNANKLLIGQSAIDKIQEVYAS

>gi|157388736|gb|ABV51441.1| 50S ribosomal protein L23 [Prochlorococcus marinus str. MIT 9215]

MTKLFDSRLADVIRKPVITEKATNALDLNQYTFEVDHRAAKPEIKAAIEALFSVKVIGVNTMNPPRRTRR

VGKFSGKRSQVKKAIVRLAEGDKIQLFPES

>gi|157388735|gb|ABV51440.1| 50S ribosomal protein L2 [Prochlorococcus marinus str. MIT 9215]

MAIRKFKPYTPGTRQRVVTDFSEITSAKPERSLIVPKHRVKGRNNRGVITCRHRGGGHKRQYRLVDFRRD

KRNINAKVAAIHYDPHRNARLALLFYEDGEKRYIIAPAGVKVGQNVISGESVPIEDGNAMPLSVMPLGSS

VHCVELYAGRGAQMVRSAGASAQVMAKEGDYVALKLPSTEVRLVRKECYATLGEVGNSEIRNTSLGKAGR

RRWLGRRPQVRGSVMNPCDHPHGGGEGKAPIGRAGPVTPWGKPALGLKTRKKNKPSNKLVVRRRRRISKR

SRGGRDS

>gi|157388734|gb|ABV51439.1| 30S Ribosomal protein S19 [Prochlorococcus marinus str. MIT 9215]

MGRSLKKGPFIADSLLKKVEKQNTDNDKSVIKTWSRSSTILPVMIGHTIAVHNGKTHIPVFITEQMIGHK

LGEFAPTRTYRGHIRDKKGAKS

>gi|157388728|gb|ABV51433.1| 30S Ribosomal protein S17 [Prochlorococcus marinus str. MIT 9215]

MALKERIGTVVSDKMDKTVVVAVINRYPHPTYKKIVSRTTRYKAHDPENTCVTGDRVKIRETRPLSAQKR

WAIEEILNKTNQAKEVKK

>gi|157388727|gb|ABV51432.1| 50S Ribosomal protein L14 [Prochlorococcus marinus str. MIT 9215]

MIQQETYLTVADNSGAKRLQCIRVLGSNRRYAHVGDVIVATVKDALPNMGVKKSEVVKAVIVRTKATLRR

NTGNSIRFDDNAAVLINEDKNPKGTRVFGPVARELRDKNYTKIVSLAPEVI

>gi|157388726|gb|ABV51431.1| 50S ribosomal protein L24 [Prochlorococcus marinus str. MIT 9215]

MLDSLKQKKNFQRIKMRIKTGDLVKVINGKEKGKTGEVLKTIPLENKVVVKGINLRTKHVKPTQEGETGR

ILTEEASLHASNVMFFSKEKNLTSKIEYFIDKEGVKKRRLKKTGEVID

>gi|157388724|gb|ABV51429.1| 30S ribosomal protein S8 [Prochlorococcus marinus str. MIT 9215]

MSNHDPISDMLTRIRNASQKKHTTTTIPGSKMSLSIAKVLQKEGFISEINEEGEGYKSQIILGLKYSGKN

KFPTIRSMQRVSKPGLRIYKNTRALPKVLGGLGVAIISTSKGVMSDRDARKQGIGGEVLCYVY

>gi|157388722|gb|ABV51427.1| 50S ribosomal protein L18 [Prochlorococcus marinus str. MIT 9215]

MTKLSRKLQTQKRHRRLRRFLIGDATRPRLSVFRSNNHIYAQVIDDSAQTTICSASTVDKELREKSEKLP

SDCNSSSIVGKLLAKRAIKKGVKQVIFDRGGNIYHGRVKALADAAREAGLEF

>gi|157388720|gb|ABV51425.1| 50S ribosomal protein L15 [Prochlorococcus marinus str. MIT 9215]

MTSTLNTLKSNSGSRKKKLRKGRGIAAGQGASCGFGMRGQKSRSGRPTRPGFEGGQMPLYRRVPKLKHFE

IINQKNFSIINLEKLNDFKDNDTVNIDSLVKKGLIFKPKFPLKILGNGKINVKLKVQAHAFTKVAKQKIE

AAGGSCELINNK

>gi|157388717|gb|ABV51422.1| 30S ribosomal protein S13 [Prochlorococcus marinus str. MIT 9215]

MARIAGIDIPREKRVEIALTYVYGIGLTRSKLILANTGVNPDTRVKDLSDSDVQKLRGATEEFTLEGDLR

RKEGMALKRLQDIGCVRGRRHRMSLPVRGQRTRTNARTRRGSRKTVAGRKK

>gi|157388716|gb|ABV51421.1| 30S ribosomal protein S11 [Prochlorococcus marinus str. MIT 9215]

MAATVKKTGSKKSKRNVPNGVVHIQSTFNNTIVSISDTSGHVISWSSAGASGFKGARKGTPFAAQTAAEA

AARRALDQGMRQIEVLVRGPGAGRETAIRALQVAGLEITLIRDVTPLPHNGCRRPKRRRV

>gi|157388714|gb|ABV51419.1| 50S ribosomal protein L17 [Prochlorococcus marinus str. MIT 9215]

MRHQLRIPLLSKPADQRKALLRGLTTQLIREGRVTTTKARAKALRNEAERMISLAKDGSLASRRRAIGYI

YDKKLVHSLFEKAKERYGDREGGYTRIVRTVSRKGDNAQMAIIELV

>gi|157388712|gb|ABV51417.1| 50S ribosomal protein L13 [Prochlorococcus marinus str. MIT 9215]

MNKTITPSLETIERNWFLVDAKNKTLGRLATEIATLLRGKNKPTFTPHLDTGDFVIVVNAEKVEVTGKKA

SQKLYRRHSGRPGGMKIEKFESLKERIPERIIEQAVKGMLPHNSLGRQQFKKLKVYKGADHPHAAQNPVL

LNS

>gi|157388711|gb|ABV51416.1| 30S ribosomal protein S9 [Prochlorococcus marinus str. MIT 9215]

MNSQIKTKAVYWGTGRRKTSVARVRLIPGNGLITINGRSGDDYLNFNPLHLNSVKAPLQTLGLENSYDIL

VNVFGGGLTGQADAIKQGAARALCELSPDNRKPLKTEGHLSRDPRAKERRKYGLKKARKAPQFSKR

>gi|157388710|gb|ABV51415.1| 50S ribosomal protein L31 [Prochlorococcus marinus str. MIT 9215]

MPKSEIHPKWYPDAKVICNGEVVMTTGSTQPELHVDVWSGNHPFFTGTQKILDTEGRVDRFMKKYGMGSA

NSATSKEQKADKDSQK

>gi|157388687|gb|ABV51392.1| 30S ribosomal protein S12 [Prochlorococcus marinus str. MIT 9215]

MPTISQLVGSERKRLTKKTKSPALKACPERRGVCTRVYTSTPKKPNSALRKVARVRLTSGFEVTAYIPGI

GHNLQEHSVVLLRGGRVKDLPGVRYHIIRGTLDTAGVKDRRQSRSKYGAKAPKD

>gi|157388686|gb|ABV51391.1| 30S ribosomal protein S7 [Prochlorococcus marinus str. MIT 9215]

MSRRNAAVKRPVLPDPQFNSRLASMMISRLMKHGKKSTAQRILSDAFSLISERTGGNAVELFETAVKNAT

PLVEVRARRVGGATYQVPMEVRQERGTAMALRWLVTFSRARNGKSMSQKLAGELMDAANETGSAVKKRED

THKMAEANKAFAHYRY

>gi|157388683|gb|ABV51388.1| 30S ribosomal protein S10 [Prochlorococcus marinus str. MIT 9215]

MTLHSKLFETMTASIAQQKIRIRLKAFDRRMLDLSCDKIIQTADTTSASAIGPIPLPTKRKIYCVLRSPH

VDKDSREHFETRTHRRIIDIYSPSAKTIDALMKLDLPSGVDIEVKL

>gi|157388664|gb|ABV51369.1| 30s Ribosomal protein S20 [Prochlorococcus marinus str. MIT 9215]

MANNKSAKKRIQIAERNRLNNKSYKSTVRTLTKKTLENCEKYKKEPNEDNMNLVKTSLNKAFSLIDKAVK

KNVLHKNNGANKKSKINKFVKTALNIK

>gi|157388490|gb|ABV51195.1| putative methyltransferase for Ribosomal protein L11 [Prochlorococcus marinus str. MIT 9215]

MEIKDWYKLTFQIESDSEDIIIWKLNELGIFSFSFEYLIKNQNKKEVNIWLPFDSWDNNSRSDFEKIISK

ILKINDSKNKFFNWSIIKEEDWLTSWKKFWAPELVGNHFLILPCWINLNEKFKDKQIIKIDPGAAFGTGS

HPSTYLCLEKMENILFSDKKVLDIGSGSGILSIAARLGGAKEVCAVDNDYLAINSTNSNFQLNFGNLNNL

NTYLGSFNEVILKNQLKQFDFVLCNILAEVIKGMIPNIYKCLRNNGEVIFSGILNSQKDEIIKILIQNNL

KLLDVSSRKNWACISAQKPASNPKHKIYL

>gi|157388482|gb|ABV51187.1| 50S ribosomal protein L27 [Prochlorococcus marinus str. MIT 9215]

MAHKKGTGSTRNGRDSNSKRLGVKAYGGEKVTAGSILIRQRGTSFLPGINVGKGKDDTLFALKEGTVSFE

SIKRNLRNRKRVNIVI

>gi|157388481|gb|ABV51186.1| 50S ribosomal protein L21 [Prochlorococcus marinus str. MIT 9215]

MTNSKKSSNNSLKNHELYAIAETSGQQFWFEVNRYYDIDRLKAKEKDKITLDKVLLLKDKDSITVGKPYV

KDAKIELEVVSHKRDKKILVYKMRPKKKTRRKMGHRQELTRVMVKSIKVGKPTPKSSSKKEETVKKETKP

KSEKSTN

>gi|157388421|gb|ABV51126.1| 30S Ribosomal protein S16 [Prochlorococcus marinus str. MIT 9215]

MIKLRLKRFGKKKEASFRIVACNSTSRRDGRPLQELGFYNPRTKETRLDTEALRTRLTQGAQPTDVVRTL

LEKGGLLEKIERPSIAIGKAKLEKEKKAKAKTKEEENEGSKTESGSNEAES

>gi|157388314|gb|ABV51019.1| 30S Ribosomal protein S14 [Prochlorococcus marinus str. MIT 9215]

MAKKSMIAREVKRKKLVKKYAAKRKSLLDEFNAAKDPMERLEIHRKIQGLPRNSAPNRVRNRCWATGKPR

GVYRDFGLCRNQLRQRAHNGELPGVVKSSW

>gi|157387934|gb|ABV50639.1| 30S Ribosomal protein S18 [Prochlorococcus marinus str. MIT 9215]

MPNSIFKKQLSPIKPGDPIDYKDVELLKKFITERGKILPRRMTGLTSKQQRDLTLAVKRARIVALLPFVN

PEG

>gi|157387900|gb|ABV50605.1| 50S ribosomal protein L28 [Prochlorococcus marinus str. MIT 9215]

MSRVCELTGAKANNGMAVSHSHIRTKKLQQVNLQKRRLWWEEGKKWVNIKISTKALKSIQKVGLDKFAKS

NGVDLQKF

>gi|157387857|gb|ABV50562.1| 30S Ribosomal protein S15 [Prochlorococcus marinus str. MIT 9215]

MELNKINFMSLDTAEKQKLIETHQVHPTDTGSAEVQVAMLSKRISKLSDHLQGNIHDFSSRQGLLKMIGK

RKRLLSYIKDKNIQRYQELVKKIGIRG

>gi|157387757|gb|ABV50462.1| 30S ribosomal protein S2 [Prochlorococcus marinus str. MIT 9215]

MAVVSLSEMMEAGAHFGHQTRRWNPKMSKYIYCARNGVHIIDLVKTALCMNNAYKWTRNAAKSGKRFLFV

GTKKQASDVVAQEATRCGAAYVNQRWLGGMLTNWTTMKARIERLKDLERMESSGSIAMRPKKEAAVLRRE

LERLQKYLGGLKGMRRLPDVVVLVDQRRESNAVLEARKLDISLVSMLDTNCDPDLCEVPIPCNDDAVRSV

QLILGRLADAINEGRKGSNAERKN

>gi|157387466|gb|ABV50171.1| Ribosomal protein L19 [Prochlorococcus marinus str. MIT 9215]

MTKMAKEKQENELETIIKADASVDVADEQKEENMVSETTQTLSASNLIKEFENEQLKKELPEIYVGDTVK

VGVKITEGNKERVQPYEGVVIAKRHGGINQTITVRRIFQGIGVERVFMLHSPQVASLKVERRGKVRRAKL

FYLRDRVGKATRVKQRFDR

>gi|157388730|gb|ABV51435.1| 50S ribosomal protein L16 [Prochlorococcus marinus str. MIT 9215]

MLSPKRTKFRKQHRGRMRGVASKGNTIAFGQFALQAQDCGWVTARQIEASRRAMTRYIKRGGQIWIRIFP

DKPVTMRPAETRMGSGKGNPEFWVAVVKPGRILFEMGGEDITEEVAKEAMRLAQYKLPVKTKFISIDKNL

EAPSQEKTKNSKKSQEEVKQ

>gi|157388729|gb|ABV51434.1| 50S ribosomal protein L29 [Prochlorococcus marinus str. MIT 9215]

MKNSESLKEFKKLNSDQITEKIDQLRKDLFDLRFKQATRQLNETHKFKIIKKQVAQLLTLSKSQSASQTT

SD

>gi|157388307|gb|ABV51012.1| 50S ribosomal protein L34 [Prochlorococcus marinus str. MIT 9215]

MTKRTFGGTSRKRKRVSGFRVRMRSHTGRRVIKSRRQKGRERIAV

>gi|157388008|gb|ABV50713.1| 30S Ribosomal protein S21 [Prochlorococcus marinus str. MIT 9215]

MTQVTVGENEGIESALRRFKRQVSKSGIFADLKRLRHHETPIEKYKRKLQQRRKARRR

>gi|157387933|gb|ABV50638.1| 50S Ribosomal protein L33 [Prochlorococcus marinus str. MIT 9215]

MTLECTEARTSTDPKRSNGVSRYTTEKNRRNTTERLELKKFNPHLNRMTIHKEIK

>gi|157387957|gb|ABV50662.1| 50S ribosomal protein L32 [Prochlorococcus marinus str. MIT 9215]

MAVPKKKKSKSKRNQRHAVWKGKAAIAAQKAISLGKSILTGKAQGFVYPIEEEEEE

>gi|157388669|gb|ABV51374.1| N utilization substance protein A [Prochlorococcus marinus str. MIT 9215]

MALVILPGLNNLIEDISEEKKLPPNIVEAALREALLKGYEKYRRTFYIGVNEDPFDEEYFSNFDVGLDLD

EEGYRILSSKIIVEEVESEDHQISLLEVKQVADDAQIGDTVVLDVTPEKEDFGRMAASTTKQVLAQKLRD

QQRKMIQEEFADLEDPVLTARVIRFERQSVIMGVSSGIGRPEVEAELPKRDQLPNDNYRANATFKVFLKE

VSEIARKGPQLFVSRANAGLVVYLFENEVPEIQEGTVKIVAVSREANPPSRAVGPRTKVAVDSVEQEVDP

VGACIGARGARIQQVVNELRGEKIDVIKWSSDPIQYILNSLSPAKVDLVRLVDPEGQHAHVLVPPDQLSL

AIGREGQNVRLAARLTGWKIDVKNSHEYDQEAEDAAVSELIIQREEEENLQREAELRLEAEQAERAAEDA

RLRELYPLPEDDEEYGEETYVEETFTDSDQLETIQDGEISAKEERKR

>gi|157388135|gb|ABV50840.1| putative ribosomal-protein-alanine acetyltransferase [Prochlorococcus marinus str. MIT 9215]

MTLDLNYINIHNKMISIKQINKKDIDLCYELDSKTISLWSKKQWANEFKKEGTKILGLLFKNFVIGICVF

QVVLDEAQINYFVVNQKFRKKGFGSYLMLYLIENCEKLSLKKLLLEVSQSNVSAERFYTRFDFYTVGVRK

NYYKDGSHALLKEKKLTTK

>gi|157387553|gb|ABV50258.1| possible acetyltransferase [Prochlorococcus marinus str. MIT 9215]

MIFRNQRSFIKKSNSISRDELINLYGLNSYEFTQTNKEEIFVCSKSKDLDLIELDQLLQTVGWSRRPIRR

VKRALDFSILVVGLWRHDDKFPRLVGFARCTGDGILEATVWDVAINPVYQGIGLGKELMKYVLKELKNIG

ISKVTLFADAEVVSFYKRQGWILEPRGSKCAFWYAN

>gi|157386932|gb|ABV49637.1| Predicted GTPase [Prochlorococcus marinus str. MIT 9215]

MKSNSKHLGLVTKKFNDFFSVDIKTQEKYGNSDKFLCKVRKSINFKDQLIYVGDQVFIENIDLKRKRALI

TSLKKRKNLLVRPSVANISNIYITFSVEEPELNLSQVNRFLISAESMGVEVSLVLTKCDLISDTRRFFLI

NKFEKWGYQAITLNLEKSDHFNNLIVELKKKECSIFMGPSGVGKTTLLNMIIPGLQNSTAPVSNKIKRGK

NTTRNVELFSISNQSYIVDTPGFNMQPLNVDIRLLPSLYSEINKQLIDEKINCKFRNCLHLNDEGCNLNK

SFERYSFYKDMIESSKSHYYQNQED

>gi|157387032|gb|ABV49737.1| possible Fe-S oxidoreductase [Prochlorococcus marinus str. MIT 9215]

MKQNSLNVKEKKLSKVAFSHVGCEKNLVDTEHMQGLLDKEGYEVDSNINEANVVVVNTCSFIQTAREESI

RKILEYTNQGKEVIVAGCMAQHFKDELIKEIPEIKGLIGTGDYQKIAKVLDRVEKGEIVNEVSKIPEFIA

DEEIPRFVDKNKFVAYLRIAEGCNYNCAFCIIPKLRGPQRSRTIESIVSEAKSLAKQGIQEIILISQITT

NYGQDIYGKPSLAKLLNELSKVPIPWIRIHYAYPTGLTDQVIRAFKDSKNIVPYFDLPLQHSHPDVLKSM

NRPWQASLNESILEKIREEIPSAVLRTSLIVGFPGEKKEHFEHLLEFLDRHKFDHVGVFIFSPEVGTAAF

DLPNKVSPEVAEARKDNVISVQQNISKDKNQSYVGSKMKILVEKISDNNELIGRSYNFAPEIDGTVILSV

KDKIDLKNYSGKFVEANISFADEYDLYGETLKIL

>gi|157387004|gb|ABV49709.1| putative ATPase, AAA family [Prochlorococcus marinus str. MIT 9215]

MHSENLFTDYSQVENNAPLADKLRPKNLEDFFGQQPILNENSLLRSAILNDKISNFIFSGPPGVGKTTLI

EIISCNTRSKLIKLNAVLSSVKELRNEIANAKERLINSKRKTILFIDEVHRFTAVQQDALLPSIESGIIT

FIGATTENPFFAVNKALVSRSRIFTLLPLVKNDLQKIIQKVITHYSKKKDSKKVHLTKDAISHLIKFSGG

DARTLINALEMAIETTADNDAKEININLSIAEDAIQKKNIVYDKNGQNHYDVISAFIKSIRGSDPDATLF

WLANMLEAGEDPNFIFRRLLISASEDIGIADPNAIVVVKSCCDAFDRVGFPEGLYFLTQASLYLAMSPKS

NSTKSIFKAIETIKSTNAFAVPLHLKNNSNSYVNPHNYPGNWVSQEYLPKSLRGLKIWEPNNNGWEKTQY

EELLRRKEN

>gi|253784512|emb|CAZ76932.1| unnamed protein product [Prochlorococcus marinus str. MIT 9215]

MAATVKKTGSKKSKRNVPNGVVHIQSTFNNTIVSISDTSGHVISWSSAGASGFKGARKGTPFAAQTAAEA

AARRALDQGMRQIEVLVRGPGAGRETAIRALQVAGLEITLIRDVTPLPHNGCRRPKRRRV

>gi|189045429|sp|A8G745.1|RS5_PROM2 RecName: Full=30S ribosomal protein S5

MTDTPTKQETQSNKDNVPGAIPVEQKKNNRNDRKRNRRGDSKNLERDSDWQERVVQIRRVSKTVKGGKKM

SFRAIVVVGNEKGQVGVGVGKAGDVIGAVRKGVSDGKKNLVRVPLTPNNSIPTLSKGRDGAANVLIRPAA

PGTGVIAGGSIRTVLELAGIKNVLAKRLGSKTPLNNARAAMVALSQLRTHKSASRERGISLEQLYS

>gi|166991001|sp|A8G7H8.1|RL9_PROM2 RecName: Full=50S ribosomal protein L9

MAKRVQLALTESIASLGKEGDLVEVAPGYARNFLLPYGKAMNVTPAVLKQIERKKEKEKIAADKLKQEAL

DFQTALSTIGRFTIKKQVGEDGVLFGTVTNGDVAEAIEEATKKEIDRRNITVPDIHNLGSFTAKIKLHPE

VNAEVNIEVTS

>gi|166982272|sp|A8G2L1.1|RL11_PROM2 RecName: Full=50S ribosomal protein L11

MAKKIVAVIKLALQAGKANPAPPVGPALGQHGVNIMAFCKEYNARTQDKAGFVIPVEISVFEDRSFTFIT

KTPPASVLITKAAGIEKGSGESAKGSVGNISKAQLEEIAKTKLPDLNCSSVESAMKVIEGTARNMGVSIT

D

>gi|166981704|sp|A8G2K9.1|RL10_PROM2 RecName: Full=50S ribosomal protein L10

MGRTLENKQQIVTEIKSLLNDSEMAVVLDYKGLTIKEMSDLRSRLRTTNGICRVTKNSLMRKAIDGDSNW

NDLESLLTGTNAFVLIKEDVGGAVKAIQSFQKDTKKSETKGALFEGRLLSDSEIKEIASLPSKEVLMAKI

AGALNGVATKIAISINEVPSGLARSLKQHSEKSES

>gi|215274852|sp|A8G757.1|RL22_PROM2 RecName: Full=50S ribosomal protein L22

MTKTSETTKIAIAHGNYIRGSASKVRRVLDQIRGRSYRDALIMLEFMPYRSTDPITKVLRSAVANAEHNL

GMDPSTLIISSAWANSGPVMKRYRPRAQGRAFSIKKQTCHISISVESAPNKTNAEVQN

>gi|189045028|sp|A8G756.1|RS3_PROM2 RecName: Full=30S ribosomal protein S3

MGHKIHPSGLRLGITQEHRSKWFATSKTYPILLQEDFKIRTFIEKKYGAAGISDVLIARKADQLELELKT

ARPGVIVGRQGSGIEELRSGIQKTIGDRTRQVRINVVEVERVDADAFLLAEYIAQQLEKRVAFRRTIRMA

LQRAQRAGVLGLKIQVGGRLNGAEIARTEWTREGRVPLHTLRAEIDYATREANTTYGVLGIKVWVFKGEV

LPKEEQTIPVGANPKRKASRRPQQFEDRSNENS

>gi|172047276|sp|A8G3C1.1|RS4_PROM2 RecName: Full=30S ribosomal protein S4

MSRYRGPRLRVTRRLGELPGLTRKASKKSNPPGQHGQARRKRSEYAIRLEEKQKLRFNYGVSEKQLVRYV

KKARAQEGSTGTNLLRLLENRLDNVCFRLGFGGTIPGSRQLVNHGHVTVNGKVLDIAGYQCKSGDVIGIK

ENKASKKLVEGNIEFPGLANVPPHLDLDKPKLTGKINGKCDREWVALEINELLVVEYYSRKV

>gi|166990987|sp|A8G2K8.1|RL7_PROM2 RecName: Full=50S ribosomal protein L7/L12

MSAKTEEILESLKSLSLLEASELVKQIEEAFGVSAAASAGVVMAAPGAAGGDGDDGAAEEKTEFDVVLES

FDAAAKIKVLKVVRNATGLGLGDAKTLVESAPKTVKEGIAKADAESLKKEIEEAGGKVTLK

>gi|166990963|sp|A8G749.1|RL5_PROM2 RecName: Full=50S ribosomal protein L5

MTLKNRYKESIRPKLLKELGLKNIHQVPKVVKVNVNRGLGEAASNSKALEASLNEMATITGQKALVTRAK

KAIAGFKIREGMPIGCTVTLRGDRMYSFLERFINLALPRIRDFRGVNPKSFDGRGNYTVGVKEQLIFPEI

SFDKIDSIRGMDITIVTSARSDQEGKALLQELGMPFSKN

>gi|166984548|sp|A8G2L0.1|RL1_PROM2 RecName: Full=50S ribosomal protein L1

MKKLSKRMASLSTKIEDRIYAPLEALSIIKENANAKFDETIEAHIRLGIDPKYTDQQLRTTVALPHGTGQ

SIRIAVITSGENVSKAKSAGADLFGEEDLVESINKGNMEFDLLIATPDMMPKVAKLGRVLGPRGLMPNPK

AGTVTNDIGNAIKEFKAGKLEFRADKAGIVHVRFGKASFTKEALFENLKTLQESIDKNKPSGAKGKYWKS

FYVTSTMGPSVQVDINAVQDYQAEG

>gi|166988024|sp|A8G5Y1.1|RL34_PROM2 RecName: Full=50S ribosomal protein L34

MTKRTFGGTSRKRKRVSGFRVRMRSHTGRRVIKSRRQKGRERIAV

>gi|226708150|sp|A8G734.1|RL31_PROM2 RecName: Full=50S ribosomal protein L31

MPKSEIHPKWYPDAKVICNGEVVMTTGSTQPELHVDVWSGNHPFFTGTQKILDTEGRVDRFMKKYGMGSA

NSATSKEQKADKDSQK

>gi|166982782|sp|A8G736.1|RL13_PROM2 RecName: Full=50S ribosomal protein L13

MNKTITPSLETIERNWFLVDAKNKTLGRLATEIATLLRGKNKPTFTPHLDTGDFVIVVNAEKVEVTGKKA

SQKLYRRHSGRPGGMKIEKFESLKERIPERIIEQAVKGMLPHNSLGRQQFKKLKVYKGADHPHAAQNPVL

LNS

>gi|226731532|sp|A8G5Y8.1|RS14_PROM2 RecName: Full=30S ribosomal protein S14

MAKKSMIAREVKRKKLVKKYAAKRKSLLDEFNAAKDPMERLEIHRKIQGLPRNSAPNRVRNRCWATGKPR

GVYRDFGLCRNQLRQRAHNGELPGVVKSSW

>gi|218547085|sp|A8G750.1|RL24_PROM2 RecName: Full=50S ribosomal protein L24

MLDSLKQKKNFQRIKMRIKTGDLVKVINGKEKGKTGEVLKTIPLENKVVVKGINLRTKHVKPTQEGETGR

ILTEEASLHASNVMFFSKEKNLTSKIEYFIDKEGVKKRRLKKTGEVID

>gi|167012628|sp|A8G735.1|RS9_PROM2 RecName: Full=30S ribosomal protein S9

MNSQIKTKAVYWGTGRRKTSVARVRLIPGNGLITINGRSGDDYLNFNPLHLNSVKAPLQTLGLENSYDIL

VNVFGGGLTGQADAIKQGAARALCELSPDNRKPLKTEGHLSRDPRAKERRKYGLKKARKAPQFSKR

>gi|167012613|sp|A8G748.1|RS8_PROM2 RecName: Full=30S ribosomal protein S8

MSNHDPISDMLTRIRNASQKKHTTTTIPGSKMSLSIAKVLQKEGFISEINEEGEGYKSQIILGLKYSGKN

KFPTIRSMQRVSKPGLRIYKNTRALPKVLGGLGVAIISTSKGVMSDRDARKQGIGGEVLCYVY

>gi|167012600|sp|A8G710.1|RS7_PROM2 RecName: Full=30S ribosomal protein S7

MSRRNAAVKRPVLPDPQFNSRLASMMISRLMKHGKKSTAQRILSDAFSLISERTGGNAVELFETAVKNAT

PLVEVRARRVGGATYQVPMEVRQERGTAMALRWLVTFSRARNGKSMSQKLAGELMDAANETGSAVKKRED

THKMAEANKAFAHYRY

>gi|167011237|sp|A8G4D1.1|RS2_PROM2 RecName: Full=30S ribosomal protein S2

MAVVSLSEMMEAGAHFGHQTRRWNPKMSKYIYCARNGVHIIDLVKTALCMNNAYKWTRNAAKSGKRFLFV

GTKKQASDVVAQEATRCGAAYVNQRWLGGMLTNWTTMKARIERLKDLERMESSGSIAMRPKKEAAVLRRE

LERLQKYLGGLKGMRRLPDVVVLVDQRRESNAVLEARKLDISLVSMLDTNCDPDLCEVPIPCNDDAVRSV

QLILGRLADAINEGRKGSNAERKN

>gi|167011227|sp|A8G532.1|RS21_PROM2 RecName: Full=30S ribosomal protein S21

MTQVTVGENEGIESALRRFKRQVSKSGIFADLKRLRHHETPIEKYKRKLQQRRKARRR

>gi|167011214|sp|A8G6Y8.1|RS20_PROM2 RecName: Full=30S ribosomal protein S20

MANNKSAKKRIQIAERNRLNNKSYKSTVRTLTKKTLENCEKYKKEPNEDNMNLVKTSLNKAFSLIDKAVK

KNVLHKNNGANKKSKINKFVKTALNIK

>gi|167011204|sp|A8G758.1|RS19_PROM2 RecName: Full=30S ribosomal protein S19

MGRSLKKGPFIADSLLKKVEKQNTDNDKSVIKTWSRSSTILPVMIGHTIAVHNGKTHIPVFITEQMIGHK

LGEFAPTRTYRGHIRDKKGAKS

>gi|167011182|sp|A8G752.1|RS17_PROM2 RecName: Full=30S ribosomal protein S17

MALKERIGTVVSDKMDKTVVVAVINRYPHPTYKKIVSRTTRYKAHDPENTCVTGDRVKIRETRPLSAQKR

WAIEEILNKTNQAKEVKK

>gi|167011170|sp|A8G695.1|RS16_PROM2 RecName: Full=30S ribosomal protein S16

MIKLRLKRFGKKKEASFRIVACNSTSRRDGRPLQELGFYNPRTKETRLDTEALRTRLTQGAQPTDVVRTL

LEKGGLLEKIERPSIAIGKAKLEKEKKAKAKTKEEENEGSKTESGSNEAES

>gi|166991601|sp|A8G741.1|RS13_PROM2 RecName: Full=30S ribosomal protein S13

MARIAGIDIPREKRVEIALTYVYGIGLTRSKLILANTGVNPDTRVKDLSDSDVQKLRGATEEFTLEGDLR

RKEGMALKRLQDIGCVRGRRHRMSLPVRGQRTRTNARTRRGSRKTVAGRKK

>gi|166991592|sp|A8G711.1|RS12_PROM2 RecName: Full=30S ribosomal protein S12

MPTISQLVGSERKRLTKKTKSPALKACPERRGVCTRVYTSTPKKPNSALRKVARVRLTSGFEVTAYIPGI

GHNLQEHSVVLLRGGRVKDLPGVRYHIIRGTLDTAGVKDRRQSRSKYGAKAPKD

>gi|166991581|sp|A8G740.1|RS11_PROM2 RecName: Full=30S ribosomal protein S11

MAATVKKTGSKKSKRNVPNGVVHIQSTFNNTIVSISDTSGHVISWSSAGASGFKGARKGTPFAAQTAAEA

AARRALDQGMRQIEVLVRGPGAGRETAIRALQVAGLEITLIRDVTPLPHNGCRRPKRRRV

>gi|166990951|sp|A8G761.1|RL4_PROM2 RecName: Full=50S ribosomal protein L4

MTTLETLKWDGKKSGKVTLDLTVAKETSSADLIHRAVLRQLANKRQGTASTLTRSEVRGGGRKPYKQKGT

GRARQGSIRTPLRPGGGIIFGPKPRSYNLDMNRKERRLALRTALMSRVSDMKAVEDFGSTLKQPKTSDII

NGLARLGIQKNEKVLVILDSPSDVIKKSINNIEKVKLIAADQLNVFDILNANKLLIGQSAIDKIQEVYAS

>gi|166990938|sp|A8G762.1|RL3_PROM2 RecName: Full=50S ribosomal protein L3

MSIGILGKKLGMSQLFDDKGNAVPVTLIEAGPCRVTQLKTNALDGYTAIQIGYGVSKEKHISKPEKGHLL

KSGEELLKHLKEYRVEETSSYEIGNQITVKNFEVGQKVDISGKSMGRGFSGYQKRHGFSRGPMSHGSKNH

RAPGSTGAGTTPGRIYPGKRMAGRYGGKQITTKGLLVLKIDDQKNLLVVKGSVPGKPGSIVNIKPNNVVG

KKGGEKS

>gi|166988036|sp|A8G7G6.1|RL35_PROM2 RecName: Full=50S ribosomal protein L35

MSKLKTRKSAAKRFKATATGKFMRRRAFHNHLLDHKSSKLKRHLSTKAVVDERDADNVRLMIPYA

>gi|166987940|sp|A8G759.1|RL2_PROM2 RecName: Full=50S ribosomal protein L2

MAIRKFKPYTPGTRQRVVTDFSEITSAKPERSLIVPKHRVKGRNNRGVITCRHRGGGHKRQYRLVDFRRD

KRNINAKVAAIHYDPHRNARLALLFYEDGEKRYIIAPAGVKVGQNVISGESVPIEDGNAMPLSVMPLGSS

VHCVELYAGRGAQMVRSAGASAQVMAKEGDYVALKLPSTEVRLVRKECYATLGEVGNSEIRNTSLGKAGR

RRWLGRRPQVRGSVMNPCDHPHGGGEGKAPIGRAGPVTPWGKPALGLKTRKKNKPSNKLVVRRRRRISKR

SRGGRDS

>gi|166987914|sp|A8G4S4.1|RL28_PROM2 RecName: Full=50S ribosomal protein L28

MSRVCELTGAKANNGMAVSHSHIRTKKLQQVNLQKRRLWWEEGKKWVNIKISTKALKSIQKVGLDKFAKS

NGVDLQKF

>gi|166987902|sp|A8G6F6.1|RL27_PROM2 RecName: Full=50S ribosomal protein L27

MAHKKGTGSTRNGRDSNSKRLGVKAYGGEKVTAGSILIRQRGTSFLPGINVGKGKDDTLFALKEGTVSFE

SIKRNLRNRKRVNIVI

>gi|166987297|sp|A8G760.1|RL23_PROM2 RecName: Full=50S ribosomal protein L23

MTKLFDSRLADVIRKPVITEKATNALDLNQYTFEVDHRAAKPEIKAAIEALFSVKVIGVNTMNPPRRTRR

VGKFSGKRSQVKKAIVRLAEGDKIQLFPES

>gi|166986914|sp|A8G6F5.1|RL21_PROM2 RecName: Full=50S ribosomal protein L21

MTNSKKSSNNSLKNHELYAIAETSGQQFWFEVNRYYDIDRLKAKEKDKITLDKVLLLKDKDSITVGKPYV

KDAKIELEVVSHKRDKKILVYKMRPKKKTRRKMGHRQELTRVMVKSIKVGKPTPKSSSKKEETVKKETKP

KSEKSTN

>gi|166984767|sp|A8G7G7.1|RL20_PROM2 RecName: Full=50S ribosomal protein L20

MARVKRGNIARKRRNKILNLAKGFRGGNKNLFRTANQRVMKALCNAYRDRRRRKRDFRRLWISRINASAR

INGTNYSKLINGMKNSEIIINRKMLAQLALSDPKCFEKIVSSVSN

>gi|166984256|sp|A8G746.1|RL18_PROM2 RecName: Full=50S ribosomal protein L18

MTKLSRKLQTQKRHRRLRRFLIGDATRPRLSVFRSNNHIYAQVIDDSAQTTICSASTVDKELREKSEKLP

SDCNSSSIVGKLLAKRAIKKGVKQVIFDRGGNIYHGRVKALADAAREAGLEF

>gi|166984045|sp|A8G738.1|RL17_PROM2 RecName: Full=50S ribosomal protein L17

MRHQLRIPLLSKPADQRKALLRGLTTQLIREGRVTTTKARAKALRNEAERMISLAKDGSLASRRRAIGYI

YDKKLVHSLFEKAKERYGDREGGYTRIVRTVSRKGDNAQMAIIELV

>gi|166983593|sp|A8G744.1|RL15_PROM2 RecName: Full=50S ribosomal protein L15

MTSTLNTLKSNSGSRKKKLRKGRGIAAGQGASCGFGMRGQKSRSGRPTRPGFEGGQMPLYRRVPKLKHFE

IINQKNFSIINLEKLNDFKDNDTVNIDSLVKKGLIFKPKFPLKILGNGKINVKLKVQAHAFTKVAKQKIE

AAGGSCELINNK

>gi|166983260|sp|A8G751.1|RL14_PROM2 RecName: Full=50S ribosomal protein L14

MIQQETYLTVADNSGAKRLQCIRVLGSNRRYAHVGDVIVATVKDALPNMGVKKSEVVKAVIVRTKATLRR

NTGNSIRFDDNAAVLINEDKNPKGTRVFGPVARELRDKNYTKIVSLAPEVI

>gi|218551747|sp|A8G4V7.2|RL33_PROM2 RecName: Full=50S ribosomal protein L33

MAKKGTRVVVTLECTEARTSTDPKRSNGVSRYTTEKNRRNTTERLELKKFNPHLNRMTIHKEIK

>gi|167011192|sp|A8G4V8.1|RS18_PROM2 RecName: Full=30S ribosomal protein S18

MPNSIFKKQLSPIKPGDPIDYKDVELLKKFITERGKILPRRMTGLTSKQQRDLTLAVKRARIVALLPFVN

PEG

>gi|215274978|sp|A8G4N1.2|RS15_PROM2 RecName: Full=30S ribosomal protein S15

MSLDTAEKQKLIETHQVHPTDTGSAEVQVAMLSKRISKLSDHLQGNIHDFSSRQGLLKMIGKRKRLLSYI

KDKNIQRYQELVKKIGIRG

>gi|166987965|sp|A8G4Y1.1|RL32_PROM2 RecName: Full=50S ribosomal protein L32

MAVPKKKKSKSKRNQRHAVWKGKAAIAAQKAISLGKSILTGKAQGFVYPIEEEEEE

>gi|166987927|sp|A8G753.1|RL29_PROM2 RecName: Full=50S ribosomal protein L29

MKNSESLKEFKKLNSDQITEKIDQLRKDLFDLRFKQATRQLNETHKFKIIKKQVAQLLTLSKSQSASQTT

SD

>gi|166983716|sp|A8G754.1|RL16_PROM2 RecName: Full=50S ribosomal protein L16

MLSPKRTKFRKQHRGRMRGVASKGNTIAFGQFALQAQDCGWVTARQIEASRRAMTRYIKRGGQIWIRIFP

DKPVTMRPAETRMGSGKGNPEFWVAVVKPGRILFEMGGEDITEEVAKEAMRLAQYKLPVKTKFISIDKNL

EAPSQEKTKNSKKSQEEVKQ

>gi|238066594|sp|A8G2A6.1|RIMO_PROM2 RecName: Full=Ribosomal protein S12 methylthiotransferase RimO; Short=S12 MTTase; Short=S12 methylthiotransferase; AltName: Full=Ribosomal protein S12 (aspartate-C(3))-methylthiotransferase; AltName: Full=Ribosome maturation factor RimO

MKQNSLNVKEKKLSKVAFSHVGCEKNLVDTEHMQGLLDKEGYEVDSNINEANVVVVNTCSFIQTAREESI

RKILEYTNQGKEVIVAGCMAQHFKDELIKEIPEIKGLIGTGDYQKIAKVLDRVEKGEIVNEVSKIPEFIA

DEEIPRFVDKNKFVAYLRIAEGCNYNCAFCIIPKLRGPQRSRTIESIVSEAKSLAKQGIQEIILISQITT

NYGQDIYGKPSLAKLLNELSKVPIPWIRIHYAYPTGLTDQVIRAFKDSKNIVPYFDLPLQHSHPDVLKSM

NRPWQASLNESILEKIREEIPSAVLRTSLIVGFPGEKKEHFEHLLEFLDRHKFDHVGVFIFSPEVGTAAF

DLPNKVSPEVAEARKDNVISVQQNISKDKNQSYVGSKMKILVEKISDNNELIGRSYNFAPEIDGTVILSV

KDKIDLKNYSGKFVEANISFADEYDLYGETLKIL

>gi|166989899|sp|A8G6G4.1|PRMA_PROM2 RecName: Full=Ribosomal protein L11 methyltransferase; Short=L11 Mtase

MEIKDWYKLTFQIESDSEDIIIWKLNELGIFSFSFEYLIKNQNKKEVNIWLPFDSWDNNSRSDFEKIISK

ILKINDSKNKFFNWSIIKEEDWLTSWKKFWAPELVGNHFLILPCWINLNEKFKDKQIIKIDPGAAFGTGS

HPSTYLCLEKMENILFSDKKVLDIGSGSGILSIAARLGGAKEVCAVDNDYLAINSTNSNFQLNFGNLNNL

NTYLGSFNEVILKNQLKQFDFVLCNILAEVIKGMIPNIYKCLRNNGEVIFSGILNSQKDEIIKILIQNNL

KLLDVSSRKNWACISAQKPASNPKHKIYL

>gi|166985968|sp|A8G7B1.1|RIMM_PROM2 RecName: Full=Ribosome maturation factor RimM

MINKNKWLTVGLITSCHGINGQVKVKSLSDFEERFLKPGMRWLQKEDEPPSKIELISGFKQPGKQTFVIK

LKDINSRNHAEQLKKFKILVKTNEIPKLKKEEFHLLELINLKVNTLENDKLNTIGKVINLENEKNNLLVI

KLFKNQKEVFIPFVKEIVPIVDIKNNFIIINPPNGLLEL

>gi|126542718|gb|ABO16960.1| 30S ribosomal protein S1-like protein A [Prochlorococcus marinus str. MIT 9301]

MNENSSQTIKELSENQEIKNSSELDNDAASQNEEDLSFEKSDIPSADSSSSRTNTDFDNAGFTQEEFASL

LGKYDYNFKPGDLVKGTVFALEPKGAMIDIGAKTAAFMPVQEVSINRVEGLNDVLQPSESREFFIMSEEN

EDGQLALSIRRIEYQRAWERVRQLQKEDATIYSEVFATNRGGALVRVEGLRGFIPGSHISARKIKDDLEG

EYLPLKFLEVDEERNRLVLSHRRALVEKKMNRLEVGEVVVGNVKGIKPYGAFIDIGGVSGLLHISEISHE

HIETPHNVLNVNDQMKVMIIDLDSERGRISLSTKALEPEPGDMLTDPQKVFSKAEEMAAKYKQMLFEQTD

DIEEIPTASNEAE

>gi|126542937|gb|ABO17179.1| 30S ribosomal protein S1-like protein B, putative Nbp1 [Prochlorococcus marinus str. MIT 9301]

MGVSNKNAQDNIEPKGNKKPLQVLHISKKDTQKIDIEHSNSQEEIKKEDIAIKPQIIKNDSVKKIEENYE

NTKDFDISLQNSTQQDLNRPLNFSEQNPDFHSERTVDEFDFDESAFLEALNANEPIGATGETISGKVIAI

ESDGLYIDIGGKAPGYMPKKECGLGVITNFKEKFSIGLEMEVLVIKEQNADGMVTVSARALILRQSWEKV

SSSAKNGELINVLINGFNRGGLTCDVDGLRGFIPRSQLEDGQDYQSFVGKTLKVAFLEVNPESRKLVLSE

KKASLVSKLTSLELGQLIEGEVLAVKPYGFFIDLGGASGLLHQSSLTNGSIRSLREVFREGEMIKALISE

IDLEKGRIGLNTALLENSAGELIIDKQKVMQEATERALKTKALFDKKEQDK

>gi|126544244|gb|ABO18486.1| 50S ribosomal protein L9 [Prochlorococcus marinus str. MIT 9301]

MAKRVQVALTESIASLGKEGDLVEVAPGYARNFLLPYGKAMNVTPAVLKQIERKKEKEKIAADKLKQEAL

DFQTALSTIGRFTIKKQVGEDGVLFGTVTNGDVAEAIEAATKKEIDRRNITVPDIHNLGSFTAKIKLHPE

VNAEVNIEVTS

>gi|126544116|gb|ABO18358.1| 50S ribosomal protein L6 [Prochlorococcus marinus str. MIT 9301]

MSRIGKTPVLIPDKVSVDFDGLTVTVKGPKGELKRQMPEGVSFDKKDNTVIVSPTTTKIFSRQRHGLCRA

LIANMVKGVSQGFSKKLEIVGVGSRAQVKGKNLVVSAGYSHPIEMIPPDGITYKVESNTNVTVSGIDKEI

VGNEAAKIRSIRPPEPYKGKGIKYHDERILRKAGKSGKK

>gi|126544114|gb|ABO18356.1| 30S ribosomal protein S5 [Prochlorococcus marinus str. MIT 9301]

MTDTPTKQEIQSKNDNVPGATPVEQKKNNRNDRKRNRRGDSKNLERDSDWQERVVQIRRVSKTVKGGKKM

SFRAIVVVGNEKGQVGVGVGKAGDVIGAVRKGVSDGKKNLVRVPLTPNNSIPTLSLGRDGAANVLIRPAA

PGTGVIAGGSIRTVLELAGIKNVLAKRLGSKTPLNNARAAMVALSQLRTHKSASKERGISLEQLYS

>gi|126542605|gb|ABO16847.1| 50S ribosomal protein L11 [Prochlorococcus marinus str. MIT 9301]

MAKKIVAVIKLALQAGKANPAPPVGPALGQHGVNIMAFCKEYNARTQDKAGFVIPVEISVFEDRSFTFIT

KTPPASVLITKAAGIEKGSGESAKGSVGNISKAQLEEIAKTKLPDLNCSSVESAMKVIEGTARNMGVSIT

D

>gi|126542603|gb|ABO16845.1| 50S ribosomal protein L10 [Prochlorococcus marinus str. MIT 9301]

MGRTLENKQQIVTEIKSLLNDSEMAVVLDYKGLTIKEMSDLRSRLQTTNGICKVTKNSLMRKAIDGDSNW

NDLESLLTGTNAFVLIKEDVGGAVKAIQSFQKDTKKSETKGALFEGRLLSDSEIKEIASLPSKEVLMAKI

AGALNGVATKIAISINEVPSGLARSLKQHSEKSES

>gi|126544125|gb|ABO18367.1| 50S ribosomal protein L22 [Prochlorococcus marinus str. MIT 9301]

MTTTPETQKTAVAHGNYVRGSASKVRRVLDQIRGRSYRDALIMLEFMPYRSTDPITKVLRSAVANAEHNL

GMDPSTLVISSAWANSGPVMKRYRPRAQGRAFSIKKQTCHISISVESAPTQTNAEVQN

>gi|126544124|gb|ABO18366.1| 30S ribosomal protein S3 [Prochlorococcus marinus str. MIT 9301]

MGHKIHPSGLRLGITQEHRSKWFATSKTYPILLQEDFKIRTFIQKKYGAAGISDVLIARKADQLELELKT

ARPGVIVGRQGSGIEELRSGIQKTIGDRTRQVRINVVEVERVDADAFLLAEYIAQQLEKRVAFRRTIRMA

LQRAQRAGVLGLKIQVGGRLNGAEIARTEWTREGRVPLHTLRAEIDYATREANTTYGVLGIKVWVFKGEV

LPKEEQTIPVGASPKRKASRRPQQFEDRSNENS

>gi|126544118|gb|ABO18360.1| 50S ribosomal protein L5 [Prochlorococcus marinus str. MIT 9301]

MTLKNRYKESIRPKLLKDLGLKNIHQVPKVVKVNVNRGLGEAASNSKALEASLNEMATITGQKALVTRAK

KAIAGFKIREGMPIGCTVTLRGDRMYSFLERFINLALPRIRDFRGVNPKSFDGRGNYTVGVKEQLIFPEI

SFDKIDSIRGMDITIVTSARSDQEGKALLQELGMPFSKN

>gi|126542810|gb|ABO17052.1| 30S ribosomal protein S4 [Prochlorococcus marinus str. MIT 9301]

MSRYRGPRLRVTRRLGELPGLTRKASKKSNPPGQHGQARRKRSEYAIRLEEKQKLRFNYGVSEKQLVRYV

KKARAQEGSTGTNLLRLLENRLDNVCFRLGFGGTIPGSRQLVNHGHVTVNGKVLDIAGYQCKSGDVIGIK

ENKASKKLVEGNIEFPGLANVPPHLDLDKPKLTGKINGKCDREWVALEINELLVVEYYSRKV

>gi|126542604|gb|ABO16846.1| 50S ribosomal protein L1 [Prochlorococcus marinus str. MIT 9301]

MKKLSKRMAALSTKIEDRIYAPLEALSIIKENANAKFDETIEAHIRLGIDPKYTDQQLRTTVALPHGTGQ

SIKIAVITSGENVSKAKAAGADLFGEEDLVESINKGNMEFDLLIATPDMMPKVAKLGRVLGPRGLMPNPK

AGTVTNDIANAIKEFKAGKLEFRADKAGIVHVRFGKASFTKEALFDNLKTLQESIDKNKPSGAKGKYWKT

FYVTSTMGPSVQVDINAVQDYQPEG

>gi|126542602|gb|ABO16844.1| 50S ribosomal protein L7/L12 [Prochlorococcus marinus str. MIT 9301]

MSAKTEEILESLKSLSLLEASELVKQIEEAFGVSAAASAGVVMAAPGAAGGDADGGAAEEKTEFDVVLES

FDAAAKIKVLKVVRNATGLGLGDAKALVESAPKTVKEGIAKADAESLKKEIEEAGGKVTLK

>gi|126544278|gb|ABO18520.1| 30S ribosomal protein S6 [Prochlorococcus marinus str. MIT 9301]

MNDQQSYYETMYILRPDIAEDEVTTYIDKYNKLLEEFGGTILDSQMRGKRRLAYQIAKHREGIYVQLSHQ

GDGQHIFKIEKAMRLSEDVIRYMTVKQEGPLPTPRPSSKSSTKADDKENPETKVEPKEEQPVISANTSTS

EKDDTETRENAES

>gi|126544233|gb|ABO18475.1| 50S ribosomal protein L20 [Prochlorococcus marinus str. MIT 9301]

MARVKRGNIARKRRNKILNLAKGFIGGNKNLFRTANQRVMKALCNAYRDRRRRKRDFRRLWISRINASAR

INGTNYSKLINGMKNAEIIINRKMLAQLALNDPKCFEKIVSSVSN

>gi|126544232|gb|ABO18474.1| 50S ribosomal protein L35 [Prochlorococcus marinus str. MIT 9301]

MSKLKTRKSAAKRFKATATGKFMRRRAFHNHLLDHKSSKLKRHLSTKAVVDERDADNVKLMIPYA

>gi|126544130|gb|ABO18372.1| 50S ribosomal protein L3 [Prochlorococcus marinus str. MIT 9301]

MSIGILGKKLGMSQLFDEKGNSVPVTLIEAGPCRITQLKTTALDGYTAVQIGYGLSKDKHISKPEKGHLL

KSGEELLKHLKEYRVEETSSYEIGNQITVKNFEVGQKVDISGKSMGRGFAGYQKRHGFSRGPMSHGSKNH

RAPGSTGAGTTPGRIYPGKRMAGRYGGKQITTKGLLVLKIDDQKNLLVVKGSVPGKPGSIINIKPNNIVG

KKGGEKS

>gi|126544129|gb|ABO18371.1| 50S ribosomal protein L4 [Prochlorococcus marinus str. MIT 9301]

MTTLETLKWDGKKSGKVSLDLAVAKKTSSADLIHRAVLRQLANKRQGTASTLTRSEVRGGGRKPYKQKGT

GRARQGSIRTPLRPGGGIIFGPKPRSYNLDMNRKERRLALRTALMSRVSDIKAVEDFGSTLKQPKTSDII

NGLARLGIQKTEKVLVILDSPSDIIKKSINNIEKVKLIAADQLNVFDILNANKLVIGQSAIDKIQEVYAS

>gi|126544128|gb|ABO18370.1| 50S ribosomal protein L23 [Prochlorococcus marinus str. MIT 9301]

MSKLFNSRLADVIRKPVITEKATNALDFNQYTFEVDHRAAKPQIKAAIEALFSVKVIGVNTMNPPRRTRR

VGKFSGKRSQVKKAIVRLAEGDKIQLFPES

>gi|126544127|gb|ABO18369.1| 50S ribosomal protein L2 [Prochlorococcus marinus str. MIT 9301]

MAIRKFKPYTPGTRQRVVTDFSEITSSKPERSLIVSKHRVKGRNNRGVITCRHRGGGHKRQYRLVDFRRD

KRNINAKVAAIHYDPHRNARLALLFYEDGEKRYIIAPAGVKVGQNVISGESVPIEDGNAMPLSVMPLGSS

VHCVELYAGRGAQMVRSAGASAQVMAKEGDYVALKLPSTEVRLVRKECYATLGEVGNSEIRNTSLGKAGR

RRWLGRRPQVRGSVMNPCDHPHGGGEGKAPIGRAGPVTPWGKPALGLKTRKKNKPSNKLVVRRRRRISKR

SRGGRDS

>gi|126544126|gb|ABO18368.1| 30S Ribosomal protein S19 [Prochlorococcus marinus str. MIT 9301]

MGRSLKKGPFIADSLLKKVEKQNTDNDKSVIKTWSRSSTILPLMIGHTIAVHNGKTHIPVFITEQMIGHK

LGEFAPTRTYRGHIRDKKGAKS

>gi|126544121|gb|ABO18363.1| 30S Ribosomal protein S17 [Prochlorococcus marinus str. MIT 9301]

MALKERIGTVVSDKMDKTVVVAVINRYPHPTYKKIVSRTTRYKAHDPENTCVLGDRVKIRETRPLSAHKR

WAIEEILNKTSQAKEVKK

>gi|126544120|gb|ABO18362.1| 50S Ribosomal protein L14 [Prochlorococcus marinus str. MIT 9301]

MIQQETYLTVADNSGAKRLQCIRVLGSNRRYAHVGDVIVATVKDALPNMGVKKSEVVKAVIVRTKATLRR

NTGNSIRFDDNAAVLINEDKNPKGTRVFGPVARELRDKNYTKIVSLAPEVI

>gi|126544119|gb|ABO18361.1| 50S ribosomal protein L24 [Prochlorococcus marinus str. MIT 9301]

MLDSLKQKKNFQRIKMRIKTGDLVKVINGKEKGKTGEVLKTIPLENRVVVKGINLRTKHVKPTQEGETGR

ILTEEASLHASNVMFFSKEKNLTSKIEYFIDKEGVKKRRLKKTGEVID

>gi|126544117|gb|ABO18359.1| 30S ribosomal protein S8 [Prochlorococcus marinus str. MIT 9301]

MSNHDPISDMLTRIRNASQKKHTTTSIPSSKMSLSIAKVLQKEGFISDINEEGEGYKSQIILGLKYSGKN

KFPTIRSMQRVSKPGLRIYKNTRALPKVLGGLGVAIISTSKGVMSDRDARKQGIGGEVLCYVY

>gi|126544115|gb|ABO18357.1| 50S ribosomal protein L18 [Prochlorococcus marinus str. MIT 9301]

MTKLSRKLQTQKRHRRLRRFLIGDATRPRLSVFRSNNHIYAQVIDDSAQTTICSASTVDKELREKSEKLP

SDCNSSSIVGKLLAKRAIKKGIKQVIFDRGGNLYHGRVKALADAAREAGLEF

>gi|126544113|gb|ABO18355.1| 50S ribosomal protein L15 [Prochlorococcus marinus str. MIT 9301]

MTSTLNTLKSNSGSRKKKLRKGRGIAAGQGASCGFGMRGQKSRSGRPTRPGFEGGQMPLYRRVPKLKHFE

IINQKNFSIINLEKLNDFKDNDTVNLDSLVKKGLIFKPKFPLKILGNGKLNVKLKVQAHAFTKVAKQKIE

DAGGSCELINNK

>gi|126544110|gb|ABO18352.1| 30S ribosomal protein S13 [Prochlorococcus marinus str. MIT 9301]

MARIAGIDIPREKRVEIALTYVYGIGLTRSKLILANTGVNPDTRVKDLSDSDVQKLRGATEEFTLEGDLR

RKEGMALKRLQDIGCVRGRRHRMSLPVRGQRTRTNARTRRGSRKTVAGRKK

>gi|126544109|gb|ABO18351.1| 30S ribosomal protein S11 [Prochlorococcus marinus str. MIT 9301]

MPATVKKTGSKKSKRNVPNGVVHIQSTFNNTIVSITDTSGHVISWSSAGASGFKGARKGTPFAAQTAAEA

AARRALDQGMRQIEVLVRGPGAGRETAIRALQVAGLEITLIRDVTPLPHNGCRRPKRRRV

>gi|126544107|gb|ABO18349.1| 50S ribosomal protein L17 [Prochlorococcus marinus str. MIT 9301]

MRHQLRIPLLSKPADQRKALLRGLTTQLIREGRVTTTKARAKALRNEAERMISLAKEGSLASRRRAIGYI

YDKKLVHSLFEKAKERYGDRNGGYTRIVRTVSRKGDNAQMAIIELV

>gi|126544105|gb|ABO18347.1| 50S ribosomal protein L13 [Prochlorococcus marinus str. MIT 9301]

MNKTITPSLETIERNWFLVDAKDKTLGRLATEIATVLRGKNKPTFTPHLDTGDFVIVVNAEKVEVTGKKA

SQKLYRRHSGRPGGMKIEKFESLQERIPERIIEQAVKGMLPHNSLGRQQFKKLKVYKGADHPHAAQNPVL

LNS

>gi|126544104|gb|ABO18346.1| 30S ribosomal protein S9 [Prochlorococcus marinus str. MIT 9301]

MNSQIKNKAVYWGTGRRKTSVARVRLIPGNGLIKINGRAGDDYLNFNPLHLNSIKAPLQTLGLENSYDMY

VNVFGGGLTGQADAIKQGAARALCELSPDNRKPLKTEGHLSRDPRAKERRKYGLKKARKAPQFSKR

>gi|126544103|gb|ABO18345.1| 50S ribosomal protein L31 [Prochlorococcus marinus str. MIT 9301]

MPKSEIHPKWYPDAKVICNGEVVMTTGSTQPELHVDVWSGNHPFFTGTQKILDTEGRVDRFMKKYGMGSA

NSSESKDQKEEKDSKK

>gi|126544083|gb|ABO18325.1| 30S ribosomal protein S12 [Prochlorococcus marinus str. MIT 9301]

MPTISQLVGSERKRLTKKTKSPALKSCPERRGVCTRVYTSTPKKPNSALRKVARVRLTSGFEVTAYIPGI

GHNLQEHSVVLLRGGRVKDLPGVRYHIIRGTLDTAGVKDRRQSRSKYGAKAPKD

>gi|126544082|gb|ABO18324.1| 30S ribosomal protein S7 [Prochlorococcus marinus str. MIT 9301]

MSRRNAAVKRPVLPDPQFNSRLASMMISRLMKHGKKSTAQRILSDAFSLISERTGGNAVELFETAVKNAT

PLVEVRARRVGGATYQVPMEVRQERGTAMALRWLVTFSRARNGKSMSQKLAGELMDAANETGSAVKKRED

THKMAEANKAFAHYRY

>gi|126544079|gb|ABO18321.1| 30S ribosomal protein S10 [Prochlorococcus marinus str. MIT 9301]

MTASIAQQKIRIRLKAFDRRMLDLSCDKIIQTADTTSASAIGPIPLPTKRKIYCVLRSPHVDKDSREHFE

TRTHRRIIDIYSPSAKTIDALMKLDLPSGVDIEVKL

>gi|126544059|gb|ABO18301.1| 30s Ribosomal protein S20 [Prochlorococcus marinus str. MIT 9301]

MANNKSAKKRIQIAERNRLINKSYKSTVRTLTKKTLENCEKYKKEPNDENKNLVTTSLNKAFSLIDKAVK

KNVLHKNNGANRKSKINNFVKTTLTTK

>gi|126543920|gb|ABO18162.1| putative methyltransferase for Ribosomal protein L11 [Prochlorococcus marinus str. MIT 9301]

METKDWYKLTFLIESDSEEIIIWKLNELGIFSFSFEYLIKNENKKEVNIWLPIDDWDESSRSSFEKIIIK

LLNINPPKNKFFEWSIIKQEDWLTSWKKYWAPELVGNHFLILPCWINLNKKFKDKQIIKIDPGAAFGTGS

HPSTYLCLEKMENILFSDKKVLDIGSGSGILSVAARLLGAKEVCAVDNDYLAINSTKSNFQLNFGNLNKL

NTYLGSFNEVILKNQLEQFDFVLCNILAEVIKGMIPNIYKCLRNNGEVIFSGILNSQKDEIIKILIQHDL

KLLDVSTRKDWACISAQKASNST

>gi|126543912|gb|ABO18154.1| 50S ribosomal protein L27 [Prochlorococcus marinus str. MIT 9301]

MAHKKGTGSTRNGRDSNSKRLGVKAYGGEKVTAGSILIRQRGTSFLPGINVGKGKDDTLFALKEGTVSFE

SIKRNLRNRKRVNIVI

>gi|126543911|gb|ABO18153.1| 50S ribosomal protein L21 [Prochlorococcus marinus str. MIT 9301]

MTNSKKSSNNSSKSSELYAIAETSGQQFWFEVDRYYDIDRLNAKEKDKITLEKVLLLKDKDSISVGKPYV

KDAKIELEVVSHKRDKKILVYKMRPKKKTRRKMGHRQELTRVMVKSITIGKSAPKSSSKKETVKKETKPK

SEKSTN

>gi|126543851|gb|ABO18093.1| 30S Ribosomal protein S16 [Prochlorococcus marinus str. MIT 9301]

MIKLRLKRFGKKKEASFRIVACNSTSRRDGRPLQELGFYNPRTKETRLDTEALRTRLTQGAQPTNVVRTL

LEKGGLLEKTERPSIAIGKARLEKEKLAKAKTKDGDNDSSKAESESNEAET

>gi|126543772|gb|ABO18014.1| 30S Ribosomal protein S14 [Prochlorococcus marinus str. MIT 9301]

MAKKSMIAREVKRKKLVKKYAAKRKSLLDEFNAAKDPMERLEIHRKIQGLPRNSAPNRVRNRCWATGKPR

GVYRDFGLCRNQLRQRAHNGELPGLVKSSW

>gi|126543372|gb|ABO17614.1| 30S Ribosomal protein S18 [Prochlorococcus marinus str. MIT 9301]

MPNSIFKKQLSPIKPGDPIDYKDVELLKKFITERGKILPRRMTGLTSKQQRDLTLAVKRARIVALLPFVN

PEG

>gi|126543338|gb|ABO17580.1| 50S ribosomal protein L28 [Prochlorococcus marinus str. MIT 9301]

MSRVCELTGAKANNGMAVSHSHIRTKKLQQVNLQKRRLWWEEGKKWVNIKISTKALKSIQKVGLDKFAKS

NGVDLNKF

>gi|126543296|gb|ABO17538.1| 30S Ribosomal protein S15 [Prochlorococcus marinus str. MIT 9301]

MSLDTAEKQKLIENHQVHPTDTGSAEVQVAMLSKRISKLSDHLQGNIHDFASRQGLLKMIGKRKRLLSYL

KDKNVQKYQELVKKIGIRG

>gi|126543194|gb|ABO17436.1| 30S ribosomal protein S2 [Prochlorococcus marinus str. MIT 9301]

MAVVSLSEMMEAGAHFGHQTRRWNPKMSKYIYCARNGVHIIDLVKTALCMNNAYKWTRNAAKSGKRFLFV

GTKKQASDVVAQEATRCGAAYVNQRWLGGMLTNWTTMKARIERLKDLERMESSGSIAMRPKKEAAVLRRE

LERLQKYLGGLKGMRRLPDVVVLVDQRRESNAVLEARKLDISLVSMLDTNCDPDLCEVPIPCNDDAVRSV

QLILGRLADAINEGRKGSNAERKN

>gi|126542881|gb|ABO17123.1| Ribosomal protein L19 [Prochlorococcus marinus str. MIT 9301]

MTKMAKEKQEKDLETGIKAGASVDVAVEQKEKNTVSETTQTLSASNLIKEFENEQLKKELPEIYVGDTVK

VGVKITEGNKERVQPYEGVVIAKRHGGINQTITVRRIFQGIGVERVFMLHSPQVASLKVERRGKVRRAKL

FYLRDRVGKATRVKQRFDR

>gi|126544123|gb|ABO18365.1| 50S ribosomal protein L16 [Prochlorococcus marinus str. MIT 9301]

MLSPKRTKFRKQHRGRMRGVASKGNTIAFGQFALQAQDCGWVTARQIEASRRAMTRYIKRGGQIWIRIFP

DKPVTMRPAETRMGSGKGNPEFWVAVVKPGRILFEMGGEDITEETAKEAMRLAQYKLPVKTKFISIDKNL

EVSSQENTKNSKKSQEEVKQ

>gi|126543765|gb|ABO18007.1| 50S ribosomal protein L34 [Prochlorococcus marinus str. MIT 9301]

MTKRTFGGTSRKRKRVSGFRVRMRSHTGRRVIKSRRQKGRERIAV

>gi|126543449|gb|ABO17691.1| 30S Ribosomal protein S21 [Prochlorococcus marinus str. MIT 9301]

MTQVTVGENEGIESALRRFKRQVSKSGIFADLKRLRHHETPVEKYKRKLQQRRKARRR

>gi|126543371|gb|ABO17613.1| 50S Ribosomal protein L33 [Prochlorococcus marinus str. MIT 9301]

MTLECTEARTSSDPKRSNGVSRYTTEKNRRNTTERLELKKFNPHLNRMTIHKEIK

>gi|126544122|gb|ABO18364.1| 50S ribosomal protein L29 [Prochlorococcus marinus str. MIT 9301]

MKNSESLKEFKKLNSDQITEKIGQLRKDLFELRFKQATRQLNETHKFKIIKKQVAQLLTLSKTQSASKTT

SD

>gi|126543396|gb|ABO17638.1| 50S ribosomal protein L32 [Prochlorococcus marinus str. MIT 9301]

MAVPKKKKSKSKRNQRHAVWKGKAAIAAQKAISLGKSVLTGKAQGFVYPIEEEEEE

>gi|126544064|gb|ABO18306.1| N utilization substance protein A [Prochlorococcus marinus str. MIT 9301]

MALVILPGLNNLIEDISEEKKLPPNIVEAALREALLKGYEKYRRTFYIGVNEDPFDEEYFSNFDVGLDLD

EEGYRILSSKIIVEEVESEDHQISLIEVKQVADDAQIGDTVVLDVTPEKEDFGRMAASTTKQVLAQKLRD

QQRKMIQEEFADLEDPVLTARVIRFERQSVIMGVSSGIGRPEVEAELPKRDQLPNDNYRANATFKVFLKE

VSEIARKGPQLFVSRANAGLVVYLFENEVPEIQEGTVKIVAVSREANPPSRAVGPRTKVAVDSVENEVDP

VGACIGARGARIQQVVNELRGEKIDVIKWSSDPIQYILNSLSPAKVDLVRLVDPEGQHAHVLVPPDQLSL

AIGREGQNVRLAARLTGWKIDVKNSHEYDQEAEDAAVSELIIQREDEEKLQREAELRLEAEQAERAAEDA

RLRELYPLPEDEEEYGEEQYEGEELTDNDPLETLQDTDISAKEEKKR

>gi|126543577|gb|ABO17819.1| putative ribosomal-protein-alanine acetyltransferase [Prochlorococcus marinus str. MIT 9301]

MISIKQMHKKDIDLCYELDSNTISLWSKEQWANEFKKDGTKIFGLLIKNLVIGICVFQVVLDEAQINYFV

VNKKFRKKGFGSYLMGYLIKKCEKLNLKNLLLEVSQSNVTAERFYSRFDFSTVGIRKNYYKDGSNALLKE

KKINNKIM

>gi|126542399|gb|ABO16641.1| Predicted GTPase [Prochlorococcus marinus str. MIT 9301]

MKTNSKNLGLVTKKFNDFFLVDLKNQENSENSDKFLCKVRKSINFKDQLIYVGDEVIIENIDLKSKRAII

TSLMKRKNLLIRPSVANISNIYITFSVEEPELNLSQVNRFLISAESMGVEVSLVLTKCDLISDKRRSSLI

DKFEKWGYQAITLNFERSDYFNNLLADLKQKKCSIFMGPSGVGKTTLLNMIIPGLQNSTAPVSNKIKRGK

NTTRNIELFSISNQSYIVDTPGFNMQPPEIDINLLPNLYAEIYKQVIEEGIKCKFRNCLHLKDEGCNLNK

SFERYSFYKEMIESAKSHYYQNQED

>gi|126542498|gb|ABO16740.1| possible Fe-S oxidoreductase [Prochlorococcus marinus str. MIT 9301]

MKQNSLNVKQKKLSKIAFSHVGCEKNLVDTEHMQGLLHKEGYEVDSNINDANVVVVNTCSFIETAREESI

RKILEYTNQGKEVIVAGCMAQHFKDELIKEIPEIKGLVGTGDYQKIAKVLDRVEKGEIVNEVSKIPEFIA

DEEMPRFVDKNKFVAYLRIAEGCNYNCAFCIIPKLRGPQRSRTIESILSEAKSLAKKGIQEIILISQITT

NYGQDIYGKPSLAKLLNELSKVPIPWIRIHYAYPTGLTDEVIRAFKDSKNIVPYFDLPLQHSHPDVLKSM

NRPWQASLNESILEKIREEIPSAVLRTSLIVGFPGEKKEHFEHLLQFLDRHKFDHVGVFIFSPEEGTAAF

HLPNKVSPEVAEARKDNVISVQQNISREKNQIYVGSKMKIMVEQISDNNELIGRSYNFAPEIDGTVILSV

KEKIDLKNYIGKFVEANISFADEYDLYGETIKIL

>gi|189045428|sp|A3PF31.1|RS5_PROM0 RecName: Full=30S ribosomal protein S5

MTDTPTKQEIQSKNDNVPGATPVEQKKNNRNDRKRNRRGDSKNLERDSDWQERVVQIRRVSKTVKGGKKM

SFRAIVVVGNEKGQVGVGVGKAGDVIGAVRKGVSDGKKNLVRVPLTPNNSIPTLSLGRDGAANVLIRPAA

PGTGVIAGGSIRTVLELAGIKNVLAKRLGSKTPLNNARAAMVALSQLRTHKSASKERGISLEQLYS

>gi|166230910|sp|A3PAS2.1|RL11_PROM0 RecName: Full=50S ribosomal protein L11

MAKKIVAVIKLALQAGKANPAPPVGPALGQHGVNIMAFCKEYNARTQDKAGFVIPVEISVFEDRSFTFIT

KTPPASVLITKAAGIEKGSGESAKGSVGNISKAQLEEIAKTKLPDLNCSSVESAMKVIEGTARNMGVSIT

D

>gi|166223840|sp|A3PFG1.1|RL9_PROM0 RecName: Full=50S ribosomal protein L9

MAKRVQVALTESIASLGKEGDLVEVAPGYARNFLLPYGKAMNVTPAVLKQIERKKEKEKIAADKLKQEAL

DFQTALSTIGRFTIKKQVGEDGVLFGTVTNGDVAEAIEAATKKEIDRRNITVPDIHNLGSFTAKIKLHPE

VNAEVNIEVTS

>gi|166230046|sp|A3PAS0.1|RL10_PROM0 RecName: Full=50S ribosomal protein L10

MGRTLENKQQIVTEIKSLLNDSEMAVVLDYKGLTIKEMSDLRSRLQTTNGICKVTKNSLMRKAIDGDSNW

NDLESLLTGTNAFVLIKEDVGGAVKAIQSFQKDTKKSETKGALFEGRLLSDSEIKEIASLPSKEVLMAKI

AGALNGVATKIAISINEVPSGLARSLKQHSEKSES

>gi|215274850|sp|A3PF42.1|RL22_PROM0 RecName: Full=50S ribosomal protein L22

MTTTPETQKTAVAHGNYVRGSASKVRRVLDQIRGRSYRDALIMLEFMPYRSTDPITKVLRSAVANAEHNL

GMDPSTLVISSAWANSGPVMKRYRPRAQGRAFSIKKQTCHISISVESAPTQTNAEVQN

>gi|166222192|sp|A3PAR9.1|RL7_PROM0 RecName: Full=50S ribosomal protein L7/L12

MSAKTEEILESLKSLSLLEASELVKQIEEAFGVSAAASAGVVMAAPGAAGGDADGGAAEEKTEFDVVLES

FDAAAKIKVLKVVRNATGLGLGDAKALVESAPKTVKEGIAKADAESLKKEIEEAGGKVTLK

>gi|166216363|sp|A3PF35.1|RL5_PROM0 RecName: Full=50S ribosomal protein L5

MTLKNRYKESIRPKLLKDLGLKNIHQVPKVVKVNVNRGLGEAASNSKALEASLNEMATITGQKALVTRAK

KAIAGFKIREGMPIGCTVTLRGDRMYSFLERFINLALPRIRDFRGVNPKSFDGRGNYTVGVKEQLIFPEI

SFDKIDSIRGMDITIVTSARSDQEGKALLQELGMPFSKN

>gi|160166293|sp|A3PAS1.1|RL1_PROM0 RecName: Full=50S ribosomal protein L1

MKKLSKRMAALSTKIEDRIYAPLEALSIIKENANAKFDETIEAHIRLGIDPKYTDQQLRTTVALPHGTGQ

SIKIAVITSGENVSKAKAAGADLFGEEDLVESINKGNMEFDLLIATPDMMPKVAKLGRVLGPRGLMPNPK

AGTVTNDIANAIKEFKAGKLEFRADKAGIVHVRFGKASFTKEALFDNLKTLQESIDKNKPSGAKGKYWKT

FYVTSTMGPSVQVDINAVQDYQPEG

>gi|152112244|sp|A3PBC7.1|RS4_PROM0 RecName: Full=30S ribosomal protein S4

MSRYRGPRLRVTRRLGELPGLTRKASKKSNPPGQHGQARRKRSEYAIRLEEKQKLRFNYGVSEKQLVRYV

KKARAQEGSTGTNLLRLLENRLDNVCFRLGFGGTIPGSRQLVNHGHVTVNGKVLDIAGYQCKSGDVIGIK

ENKASKKLVEGNIEFPGLANVPPHLDLDKPKLTGKINGKCDREWVALEINELLVVEYYSRKV

>gi|152060891|sp|A3PF41.1|RS3_PROM0 RecName: Full=30S ribosomal protein S3

MGHKIHPSGLRLGITQEHRSKWFATSKTYPILLQEDFKIRTFIQKKYGAAGISDVLIARKADQLELELKT

ARPGVIVGRQGSGIEELRSGIQKTIGDRTRQVRINVVEVERVDADAFLLAEYIAQQLEKRVAFRRTIRMA

LQRAQRAGVLGLKIQVGGRLNGAEIARTEWTREGRVPLHTLRAEIDYATREANTTYGVLGIKVWVFKGEV

LPKEEQTIPVGASPKRKASRRPQQFEDRSNENS

>gi|166199805|sp|A3PE32.1|RL34_PROM0 RecName: Full=50S ribosomal protein L34

MTKRTFGGTSRKRKRVSGFRVRMRSHTGRRVIKSRRQKGRERIAV

>gi|226708144|sp|A3PF20.1|RL31_PROM0 RecName: Full=50S ribosomal protein L31

MPKSEIHPKWYPDAKVICNGEVVMTTGSTQPELHVDVWSGNHPFFTGTQKILDTEGRVDRFMKKYGMGSA

NSSESKDQKEEKDSKK

>gi|166231009|sp|A3PF22.1|RL13_PROM0 RecName: Full=50S ribosomal protein L13

MNKTITPSLETIERNWFLVDAKDKTLGRLATEIATVLRGKNKPTFTPHLDTGDFVIVVNAEKVEVTGKKA

SQKLYRRHSGRPGGMKIEKFESLQERIPERIIEQAVKGMLPHNSLGRQQFKKLKVYKGADHPHAAQNPVL

LNS

>gi|156637360|sp|A3PF00.1|RS12_PROM0 RecName: Full=30S ribosomal protein S12

MPTISQLVGSERKRLTKKTKSPALKSCPERRGVCTRVYTSTPKKPNSALRKVARVRLTSGFEVTAYIPGI

GHNLQEHSVVLLRGGRVKDLPGVRYHIIRGTLDTAGVKDRRQSRSKYGAKAPKD

>gi|226731530|sp|A3PE39.1|RS14_PROM0 RecName: Full=30S ribosomal protein S14

MAKKSMIAREVKRKKLVKKYAAKRKSLLDEFNAAKDPMERLEIHRKIQGLPRNSAPNRVRNRCWATGKPR

GVYRDFGLCRNQLRQRAHNGELPGLVKSSW

>gi|218547104|sp|A3PF36.1|RL24_PROM0 RecName: Full=50S ribosomal protein L24

MLDSLKQKKNFQRIKMRIKTGDLVKVINGKEKGKTGEVLKTIPLENRVVVKGINLRTKHVKPTQEGETGR

ILTEEASLHASNVMFFSKEKNLTSKIEYFIDKEGVKKRRLKKTGEVID

>gi|166987293|sp|A3PF45.1|RL23_PROM0 RecName: Full=50S ribosomal protein L23

MSKLFNSRLADVIRKPVITEKATNALDFNQYTFEVDHRAAKPQIKAAIEALFSVKVIGVNTMNPPRRTRR

VGKFSGKRSQVKKAIVRLAEGDKIQLFPES

>gi|166986911|sp|A3PEH8.1|RL21_PROM0 RecName: Full=50S ribosomal protein L21

MTNSKKSSNNSSKSSELYAIAETSGQQFWFEVDRYYDIDRLNAKEKDKITLEKVLLLKDKDSISVGKPYV

KDAKIELEVVSHKRDKKILVYKMRPKKKTRRKMGHRQELTRVMVKSITIGKSAPKSSSKKETVKKETKPK

SEKSTN

>gi|166234497|sp|A3PF30.1|RL15_PROM0 RecName: Full=50S ribosomal protein L15

MTSTLNTLKSNSGSRKKKLRKGRGIAAGQGASCGFGMRGQKSRSGRPTRPGFEGGQMPLYRRVPKLKHFE

IINQKNFSIINLEKLNDFKDNDTVNLDSLVKKGLIFKPKFPLKILGNGKLNVKLKVQAHAFTKVAKQKIE

DAGGSCELINNK

>gi|166234341|sp|A3PCR3.1|RS15_PROM0 RecName: Full=30S ribosomal protein S15

MSLDTAEKQKLIENHQVHPTDTGSAEVQVAMLSKRISKLSDHLQGNIHDFASRQGLLKMIGKRKRLLSYL

KDKNVQKYQELVKKIGIRG

>gi|166233175|sp|A3PF47.1|RL3_PROM0 RecName: Full=50S ribosomal protein L3

MSIGILGKKLGMSQLFDEKGNSVPVTLIEAGPCRITQLKTTALDGYTAVQIGYGLSKDKHISKPEKGHLL

KSGEELLKHLKEYRVEETSSYEIGNQITVKNFEVGQKVDISGKSMGRGFAGYQKRHGFSRGPMSHGSKNH

RAPGSTGAGTTPGRIYPGKRMAGRYGGKQITTKGLLVLKIDDQKNLLVVKGSVPGKPGSIINIKPNNIVG

KKGGEKS

>gi|166232681|sp|A3PF37.1|RL14_PROM0 RecName: Full=50S ribosomal protein L14

MIQQETYLTVADNSGAKRLQCIRVLGSNRRYAHVGDVIVATVKDALPNMGVKKSEVVKAVIVRTKATLRR

NTGNSIRFDDNAAVLINEDKNPKGTRVFGPVARELRDKNYTKIVSLAPEVI

>gi|166231308|sp|A3PEZ6.1|RS10_PROM0 RecName: Full=30S ribosomal protein S10

MTASIAQQKIRIRLKAFDRRMLDLSCDKIIQTADTTSASAIGPIPLPTKRKIYCVLRSPHVDKDSREHFE

TRTHRRIIDIYSPSAKTIDALMKLDLPSGVDIEVKL

>gi|166229585|sp|A3PF21.1|RS9_PROM0 RecName: Full=30S ribosomal protein S9

MNSQIKNKAVYWGTGRRKTSVARVRLIPGNGLIKINGRAGDDYLNFNPLHLNSIKAPLQTLGLENSYDMY

VNVFGGGLTGQADAIKQGAARALCELSPDNRKPLKTEGHLSRDPRAKERRKYGLKKARKAPQFSKR

>gi|166228458|sp|A3PEZ9.1|RS7_PROM0 RecName: Full=30S ribosomal protein S7

MSRRNAAVKRPVLPDPQFNSRLASMMISRLMKHGKKSTAQRILSDAFSLISERTGGNAVELFETAVKNAT

PLVEVRARRVGGATYQVPMEVRQERGTAMALRWLVTFSRARNGKSMSQKLAGELMDAANETGSAVKKRED

THKMAEANKAFAHYRY

>gi|166226598|sp|A3PCV5.1|RL28_PROM0 RecName: Full=50S ribosomal protein L28

MSRVCELTGAKANNGMAVSHSHIRTKKLQQVNLQKRRLWWEEGKKWVNIKISTKALKSIQKVGLDKFAKS

NGVDLNKF

>gi|166225294|sp|A3PCG1.1|RS2_PROM0 RecName: Full=30S ribosomal protein S2

MAVVSLSEMMEAGAHFGHQTRRWNPKMSKYIYCARNGVHIIDLVKTALCMNNAYKWTRNAAKSGKRFLFV

GTKKQASDVVAQEATRCGAAYVNQRWLGGMLTNWTTMKARIERLKDLERMESSGSIAMRPKKEAAVLRRE

LERLQKYLGGLKGMRRLPDVVVLVDQRRESNAVLEARKLDISLVSMLDTNCDPDLCEVPIPCNDDAVRSV

QLILGRLADAINEGRKGSNAERKN

>gi|166225029|sp|A3PEH9.1|RL27_PROM0 RecName: Full=50S ribosomal protein L27

MAHKKGTGSTRNGRDSNSKRLGVKAYGGEKVTAGSILIRQRGTSFLPGINVGKGKDDTLFALKEGTVSFE

SIKRNLRNRKRVNIVI

>gi|166224050|sp|A3PD66.1|RS21_PROM0 RecName: Full=30S ribosomal protein S21

MTQVTVGENEGIESALRRFKRQVSKSGIFADLKRLRHHETPVEKYKRKLQQRRKARRR

>gi|166223965|sp|A3PEX6.1|RS20_PROM0 RecName: Full=30S ribosomal protein S20

MANNKSAKKRIQIAERNRLINKSYKSTVRTLTKKTLENCEKYKKEPNDENKNLVTTSLNKAFSLIDKAVK

KNVLHKNNGANRKSKINNFVKTTLTTK

>gi|166219718|sp|A3PF38.1|RS17_PROM0 RecName: Full=30S ribosomal protein S17

MALKERIGTVVSDKMDKTVVVAVINRYPHPTYKKIVSRTTRYKAHDPENTCVLGDRVKIRETRPLSAHKR

WAIEEILNKTSQAKEVKK

>gi|166219676|sp|A3PFF0.1|RL20_PROM0 RecName: Full=50S ribosomal protein L20

MARVKRGNIARKRRNKILNLAKGFIGGNKNLFRTANQRVMKALCNAYRDRRRRKRDFRRLWISRINASAR

INGTNYSKLINGMKNAEIIINRKMLAQLALNDPKCFEKIVSSVSN

>gi|166218716|sp|A3PF32.1|RL18_PROM0 RecName: Full=50S ribosomal protein L18

MTKLSRKLQTQKRHRRLRRFLIGDATRPRLSVFRSNNHIYAQVIDDSAQTTICSASTVDKELREKSEKLP

SDCNSSSIVGKLLAKRAIKKGIKQVIFDRGGNLYHGRVKALADAAREAGLEF

>gi|166216200|sp|A3PF24.1|RL17_PROM0 RecName: Full=50S ribosomal protein L17

MRHQLRIPLLSKPADQRKALLRGLTTQLIREGRVTTTKARAKALRNEAERMISLAKEGSLASRRRAIGYI

YDKKLVHSLFEKAKERYGDRNGGYTRIVRTVSRKGDNAQMAIIELV

>gi|166201331|sp|A3PEB8.1|RS16_PROM0 RecName: Full=30S ribosomal protein S16

MIKLRLKRFGKKKEASFRIVACNSTSRRDGRPLQELGFYNPRTKETRLDTEALRTRLTQGAQPTNVVRTL

LEKGGLLEKTERPSIAIGKARLEKEKLAKAKTKDGDNDSSKAESESNEAET

>gi|166199900|sp|A3PF43.1|RS19_PROM0 RecName: Full=30S ribosomal protein S19

MGRSLKKGPFIADSLLKKVEKQNTDNDKSVIKTWSRSSTILPLMIGHTIAVHNGKTHIPVFITEQMIGHK

LGEFAPTRTYRGHIRDKKGAKS

>gi|166199841|sp|A3PF46.1|RL4_PROM0 RecName: Full=50S ribosomal protein L4

MTTLETLKWDGKKSGKVSLDLAVAKKTSSADLIHRAVLRQLANKRQGTASTLTRSEVRGGGRKPYKQKGT

GRARQGSIRTPLRPGGGIIFGPKPRSYNLDMNRKERRLALRTALMSRVSDIKAVEDFGSTLKQPKTSDII

NGLARLGIQKTEKVLVILDSPSDIIKKSINNIEKVKLIAADQLNVFDILNANKLVIGQSAIDKIQEVYAS

>gi|166199816|sp|A3PFE9.1|RL35_PROM0 RecName: Full=50S ribosomal protein L35

MSKLKTRKSAAKRFKATATGKFMRRRAFHNHLLDHKSSKLKRHLSTKAVVDERDADNVKLMIPYA

>gi|160358604|sp|A3PF44.1|RL2_PROM0 RecName: Full=50S ribosomal protein L2

MAIRKFKPYTPGTRQRVVTDFSEITSSKPERSLIVSKHRVKGRNNRGVITCRHRGGGHKRQYRLVDFRRD

KRNINAKVAAIHYDPHRNARLALLFYEDGEKRYIIAPAGVKVGQNVISGESVPIEDGNAMPLSVMPLGSS

VHCVELYAGRGAQMVRSAGASAQVMAKEGDYVALKLPSTEVRLVRKECYATLGEVGNSEIRNTSLGKAGR

RRWLGRRPQVRGSVMNPCDHPHGGGEGKAPIGRAGPVTPWGKPALGLKTRKKNKPSNKLVVRRRRRISKR

SRGGRDS

>gi|158706259|sp|A3PF27.1|RS13_PROM0 RecName: Full=30S ribosomal protein S13

MARIAGIDIPREKRVEIALTYVYGIGLTRSKLILANTGVNPDTRVKDLSDSDVQKLRGATEEFTLEGDLR

RKEGMALKRLQDIGCVRGRRHRMSLPVRGQRTRTNARTRRGSRKTVAGRKK

>gi|152060805|sp|A3PF26.1|RS11_PROM0 RecName: Full=30S ribosomal protein S11

MPATVKKTGSKKSKRNVPNGVVHIQSTFNNTIVSITDTSGHVISWSSAGASGFKGARKGTPFAAQTAAEA

AARRALDQGMRQIEVLVRGPGAGRETAIRALQVAGLEITLIRDVTPLPHNGCRRPKRRRV

>gi|148841159|sp|A3PF34.1|RS8_PROM0 RecName: Full=30S ribosomal protein S8

MSNHDPISDMLTRIRNASQKKHTTTSIPSSKMSLSIAKVLQKEGFISDINEEGEGYKSQIILGLKYSGKN

KFPTIRSMQRVSKPGLRIYKNTRALPKVLGGLGVAIISTSKGVMSDRDARKQGIGGEVLCYVY

>gi|218551746|sp|A3PCY8.2|RL33_PROM0 RecName: Full=50S ribosomal protein L33

MAKKGTRVVVTLECTEARTSSDPKRSNGVSRYTTEKNRRNTTERLELKKFNPHLNRMTIHKEIK

>gi|166220970|sp|A3PCY9.1|RS18_PROM0 RecName: Full=30S ribosomal protein S18

MPNSIFKKQLSPIKPGDPIDYKDVELLKKFITERGKILPRRMTGLTSKQQRDLTLAVKRARIVALLPFVN

PEG

>gi|166228239|sp|A3PF39.1|RL29_PROM0 RecName: Full=50S ribosomal protein L29

MKNSESLKEFKKLNSDQITEKIGQLRKDLFELRFKQATRQLNETHKFKIIKKQVAQLLTLSKTQSASKTT

SD

>gi|166199701|sp|A3PF40.1|RL16_PROM0 RecName: Full=50S ribosomal protein L16

MLSPKRTKFRKQHRGRMRGVASKGNTIAFGQFALQAQDCGWVTARQIEASRRAMTRYIKRGGQIWIRIFP

DKPVTMRPAETRMGSGKGNPEFWVAVVKPGRILFEMGGEDITEETAKEAMRLAQYKLPVKTKFISIDKNL

EVSSQENTKNSKKSQEEVKQ

>gi|156630850|sp|A3PD13.1|RL32_PROM0 RecName: Full=50S ribosomal protein L32

MAVPKKKKSKSKRNQRHAVWKGKAAIAAQKAISLGKSVLTGKAQGFVYPIEEEEEE

>gi|238066596|sp|A3PAG5.1|RIMO_PROM0 RecName: Full=Ribosomal protein S12 methylthiotransferase RimO; Short=S12 MTTase; Short=S12 methylthiotransferase; AltName: Full=Ribosomal protein S12 (aspartate-C(3))-methylthiotransferase; AltName: Full=Ribosome maturation factor RimO

MKQNSLNVKQKKLSKIAFSHVGCEKNLVDTEHMQGLLHKEGYEVDSNINDANVVVVNTCSFIETAREESI

RKILEYTNQGKEVIVAGCMAQHFKDELIKEIPEIKGLVGTGDYQKIAKVLDRVEKGEIVNEVSKIPEFIA

DEEMPRFVDKNKFVAYLRIAEGCNYNCAFCIIPKLRGPQRSRTIESILSEAKSLAKKGIQEIILISQITT

NYGQDIYGKPSLAKLLNELSKVPIPWIRIHYAYPTGLTDEVIRAFKDSKNIVPYFDLPLQHSHPDVLKSM

NRPWQASLNESILEKIREEIPSAVLRTSLIVGFPGEKKEHFEHLLQFLDRHKFDHVGVFIFSPEEGTAAF

HLPNKVSPEVAEARKDNVISVQQNISREKNQIYVGSKMKIMVEQISDNNELIGRSYNFAPEIDGTVILSV

KEKIDLKNYIGKFVEANISFADEYDLYGETIKIL

>gi|166223427|sp|A3PEI7.1|PRMA_PROM0 RecName: Full=Ribosomal protein L11 methyltransferase; Short=L11 Mtase

METKDWYKLTFLIESDSEEIIIWKLNELGIFSFSFEYLIKNENKKEVNIWLPIDDWDESSRSSFEKIIIK

LLNINPPKNKFFEWSIIKQEDWLTSWKKYWAPELVGNHFLILPCWINLNKKFKDKQIIKIDPGAAFGTGS

HPSTYLCLEKMENILFSDKKVLDIGSGSGILSVAARLLGAKEVCAVDNDYLAINSTKSNFQLNFGNLNKL

NTYLGSFNEVILKNQLEQFDFVLCNILAEVIKGMIPNIYKCLRNNGEVIFSGILNSQKDEIIKILIQHDL

KLLDVSTRKDWACISAQKASNST

>gi|166226462|sp|A3PF96.1|RIMM_PROM0 RecName: Full=Ribosome maturation factor RimM

MINKNEWLTVGLITSCHGINGQVKVKSLSDFEERFLKPGMRWLQKENEPPSKIELISGFKQPGKETFIVK

LQGINTRNDAEQLKTFKILVRTDKLPKLKKEEFHLLELINLEVKTLENDELKTIGKVINLENEKNNLLII

ELFKNQKKVLIPFVKEIVPLVDIKNQFVIINPPNGLLEL

>gi|123964195|gb|ABM78951.1| 30S ribosomal protein S1, protein A [Prochlorococcus marinus str. MIT 9303]

MSATPTEQVQDSAAESTSSEQVINTASETAEAANQALAEEDLSIPEDVPTADDPSSRAAKNDLSGAGFTL

DEFASLLSKYDYNFKPGDIVNGTVFALESKGAMIDIGAKTAAFMPLQEVSINRVEGLSDVLLPGEIREFF

IMSEENEDGQLSLSIRRIEYQRAWERVRQLQKEDATIYSEVFATNRGGALVRVEGLRGFIPGSHISTRKP

KEELVADFLPLKFLEVDEERNRLVLSHRRALVERKMNRLEVGEVVIGAVRGIKPYGAFIDIGGVSGLLHI

SEISHEHIETPHSVLNVNDQMKVMIIDLDAERGRISLSTKALEPEPGDMLTDPQKVFDKAEEMAARYKQM

LLEQAEEGEDPEVVPLD

>gi|123962770|gb|ABM77526.1| 30S ribosomal protein S1 protein B, putative Nbp1 [Prochlorococcus marinus str. MIT 9303]

MLFTGPEWPNQGGSLQPNRLCSLWPAEMGKAPARNRHQHQSANRTTPCLSECLTDDPKAPMAGSGSPQPN

RPKPPKPAAEAPRKPLQVMHISRRGEQEKLVREAAETTSPGSEATTGSGQLSNAPNRSVSADAASDESRF

DLGELQNMTMADLLGPADQSRRSGAAPKGNDYRNEEGQSNPARSVDDFDFDEDAFLAALDENEPIGTTGE

VATGKVIALESDGVYVDIGGKAPGFMPKNECGLGVITNLKERFPKGLEVEVLVTREQNADGMVTISCRAL

ELRKSWSKVQQMEKEGKVAQVKVNGFNRGGVTCDLEGLRGFIPRSQLQNGENHEALVGKTLGVAFLEVNP

ETRKLVLSEKRAATAARFSELEVGQLVEGQVVAVKPYGFFIDLGGVSGLLHQSMITGGSLRSLREVFNQG

DRVKALITEMDPGRGRIALNTALLEGQPGELLIEKDKVMAEATDRANKARNVLRQQEQSAG

>gi|123964870|gb|ABM79626.1| 50S ribosomal protein L9 [Prochlorococcus marinus str. MIT 9303]

MAKRVQVVLNEDVLSLGKDGDLVEVAPGYARNFLLPFGKAVPVTPAVMKQVGHRRAKQAEHQAAIKQEAL

DFQTALVTIGRFTVKKQTGEDDVLFGTVTNGDVAEAIETATKKEIDRRNIIVPEIHRTGSYKVQVKLHNE

VNAEINLEVVSY

>gi|123964753|gb|ABM79509.1| 50S ribosomal protein L10 [Prochlorococcus marinus str. MIT 9303]

MGRTLESKQQIVEELKGLLGEAEMALVLDYQGLSIKEMSDLRTRLQASNGVCKVTKNTLMRHAINGNGAW

SNLESLLTGTNAFVLIKGDVGGAVKAVQAFQKDTKKSETKGGLFEGKLLSQGEIKAIGDLPTKEVLMAQI

AGSLNALATKVAVGINEVPSGLARALHQHAESGES

>gi|123964751|gb|ABM79507.1| 50S ribosomal protein L11 [Prochlorococcus marinus str. MIT 9303]

MGHHLAALVPRARSSAIVQSRSSMAKKVVSVIKLALQAGKANPAPPVGPALGQHGVNIMAFCKEYNARTQ

DKAGLVIPVEISVFEDRSFTFITKTPPASVLITKAAGIEKGSGESAHGKVGSLSRSQLEEIAKTKLPDLN

CTSIESAMRIIEGTARNMGVSISD

>gi|123964298|gb|ABM79054.1| 30S ribosomal protein S5 [Prochlorococcus marinus str. MIT 9303]

MTQPNTQTTPNDVPAAAEGQHQEQQQQQRRGGGRERRGGGRRGDRRGQERDSEWQERVVQIRRVSKTVKG

GKKMSFRAIVVVGNERGQVGVGVGKAGDVIGAVRKGVADGKKHLVKVPLTRHNSIPTLSNGREGAANVLI

RPAAPGTGVIAGGSIRTVLELAGIKNVLAKRLGSKTPLNNARAAMVALASLRTHKETAKERGISLEQIYS

>gi|123964296|gb|ABM79052.1| 50S ribosomal protein L6 [Prochlorococcus marinus str. MIT 9303]

MSRIGKNPIPIPDKVAVTLDGLAVSVKGPKGELNRTLPEGVSVSQVENTIVVTPTSQKRKSRERHGLCRS

LVANMVEGVSKGYTRKLELIGVGSRAQVKGKKLVVSAGFSHPVEMDPPEGITFAVENNTNVTVSGADKEL

VGNEAAKIRAIRPPEPYKGKGIRYEGERILRKAGKSGKK

>gi|123964752|gb|ABM79508.1| 50S ribosomal protein L1 [Prochlorococcus marinus str. MIT 9303]

MCRLHPTSTMPKLSKRITGLLAKVEDRVYQPIEAIQLVKENATAKFDETIEAHVRLGIDPKYTDQQLRTT

VALPQGTGQSVRIAVITRGEKLAEAKTAGAELAGDDDLVESIGKGQMDFDLLIATPDMMPKVAKLGRVLG

PRGLMPNPKAGTVTTDLAAAIKEFKAGKLEFRADRAGIVHVRFGKASFSADALLENLKTLQETIDRNKPS

GAKGRYWKSLYITSTMGPSVEVDVTALQDIEEDA

>gi|123964294|gb|ABM79050.1| 50S ribosomal protein L5 [Prochlorococcus marinus str. MIT 9303]

MSLKQRYRETIQPKLLKDLSLSNIHEVPKVLKITVNRGLGEAAQNAKSLEASITELATITGQKVVVTRAK

KAIAGFKIRQGMPIGCAVTLRGERMYAFLERLINLALPRIRDFRGVSPKSFDGRGNYTLGVREQLIFPEV

SFDKIDAIRGMDITIVTSARTDEEGQSLLREMGMPFRSN

>gi|123964287|gb|ABM79043.1| 30S ribosomal protein S3 [Prochlorococcus marinus str. MIT 9303]

MGNKIHPTGLRLGITQEHRSRWYATSKMYPILLQEDDRIRRFIHKKYGAAGISDVLIARKADQLEVELKT

ARPGVLVGRQGSGIEELRTGIQKTIGDHSRQVRINVVEVERVDADAFLLAEYIAQQLEKRVAFRRTIRMA

VQRAQRAGVLGLKIQVGGRLNGAEIARNEWTREGRVPLHTLRAEIDYATKVASTTYGVLGIKVWIFKGEV

LGDEAQSMPVGASPRRRGNRRPQQFEDRSNEG

>gi|123964286|gb|ABM79042.1| 50S ribosomal protein L22 [Prochlorococcus marinus str. MIT 9303]

MTTSSPTTTTIAKAHGRFIRGSVSKVRRVLDQIRGRTYRDALIMLEFMPYRSTGPITKVLRSAVANAEHN

LGLDPASLVIAQASADMGPSMKRYRPRAQGRAFAIKKQTCHISIAVAAQTDS

>gi|123964095|gb|ABM78851.1| 30S ribosomal protein S4 [Prochlorococcus marinus str. MIT 9303]

MSRYRGPRLRITRRLGDLPGLTRKAAKRSHPPGQHGQARRKRSEYAIRLEEKQKLRFNYGISERQLVRYV

KKARAQDGSTGTNLLKLLENRLDNVCFRLGFGPTVPGARQLVNHGHVTVNGRVLDIASYQCKAGDVVAIR

ERKGSKKLAEANLEFPGLANVPPHIELDKAKMSAKIISKCEREWVALEINELLVVEYYSRKV

>gi|123964981|gb|ABM79737.1| 30S ribosomal protein S6 [Prochlorococcus marinus str. MIT 9303]

MSQQPYYETMYILRPDIPEEEVETHVTKYREMVTEAGAEVLDNQMRGKRRLAYPISNHKEGIYVQLSHNG

NGQQVAVLEKAMRLSEDVIRYLTVKQEGPLPAPRIVPGSEPEPVEQQEAAAVEA

>gi|123964343|gb|ABM79099.1| 30S ribosomal protein S10 [Prochlorococcus marinus str. MIT 9303]

MSTAIAQQKIRIRLKAFDRRMLDLSCDKIIETADNTAATAIGPIPLPTKRKIYCVLCSPHVDKDSREHFE

TRTHRRIIDIYNPSAKTIDALMKLDLPSGVDIEVKL

>gi|123964339|gb|ABM79095.1| 30S ribosomal protein S7 [Prochlorococcus marinus str. MIT 9303]

MSRRNAAEKRPVLPDPQFNNRLATMMISRLMKHGKKSTAQRILAQAFGLINERTGGDPIELFETAVKNAT

PLVEVRARRVGGATYQVPMEVRQERGTAMALRWLVNFSRARNGRSMSQKLAAELMDAANEAGSAVRKREE

THKMAEANKAFAHYRY

>gi|123964338|gb|ABM79094.1| 30S ribosomal protein S12 [Prochlorococcus marinus str. MIT 9303]

MPTIQQLIRTERQHLTRKTKSPALRACPERRGVCTRVYTSTPKKPNSALRKVARVRLTSGFEVTAYIPGI

GHNLQEHSVVLIRGGRVKDLPGVRYHIIRGTLDTAGVKDRSQSRSKYGAKASKQD

>gi|123964310|gb|ABM79066.1| 50S ribosomal protein L31 [Prochlorococcus marinus str. MIT 9303]

MPKPDIHPNWYPDAKVICNGEVVMTTGSTQPELHVDVWSGNHPFFTGTQKILDTEGRVDRFMRKYGMGSA

DAAADEKKTDAKNNNKDNTSKED

>gi|123964309|gb|ABM79065.1| 30S ribosomal protein S9 [Prochlorococcus marinus str. MIT 9303]

MSSSNNSVVYWGTGRRKTSVARVRLVPGTGTITINGRPGDHYLNFNPAYLAAVKAPLQTLGLNEQYDVLV

NVHGGGLTGQADAIKQGAARALCELSADNRKPLKTEGHLSRDPRAKERRKYGLKKARKAPQFSKR

>gi|123964308|gb|ABM79064.1| 50S ribosomal protein L13 [Prochlorococcus marinus str. MIT 9303]

MNKTSVPSIDSIERQWFLVDAENQTLGRLATEVASVLRGKNKPSFTPHLDTGDFVVVVNADKIRVSGNKA

NQKLYRRHSGRPGGMKVETFQALQDRLPERIVEKAIKGMLPHNALGRQLFRKLKVYRGPEHPHSAQRPQT

LQLNPAASSQ

>gi|123964306|gb|ABM79062.1| 50S ribosomal protein L17 [Prochlorococcus marinus str. MIT 9303]

MRHQCRVPKLGRPTDQRKAMLRGLTTQLIREGRVTTTKARAKALRDEAERMITLAKNGSLASRRRAIGYI

YDKQLVHALFDKAQDRYGDRQGGYTRIIRTVPRRGDNAEMAIIELV

>gi|123964304|gb|ABM79060.1| 30S ribosomal protein S11 [Prochlorococcus marinus str. MIT 9303]

MAKPTKKTGSKKTKRNVPNGVAHIQSTFNNTIVSITDTAGEVIAWSSAGASGFKGARKGTPFAAQTAAEA

AARRALEQGMRQIEVLVRGPGSGRETAIRALQVAGLEITLIRDVTPLPHNGCRRPKRRRV

>gi|123964303|gb|ABM79059.1| 30S ribosomal protein S13 [Prochlorococcus marinus str. MIT 9303]

MARIAGVDIPRDKRVEVALTYIYGIGLTRAKTILTKSGVNPDIRVKDLEDGDVQKLRTALEAFTIEGDLR

RQEGMALKRLQDIGCLRGRRHRMSLPVRGQRTRTNARTRRGARKTVAGKKK

>gi|123964299|gb|ABM79055.1| 50S ribosomal protein L15 [Prochlorococcus marinus str. MIT 9303]

MPELPWWLWQVSAPIRRPQRNGGSPSSRSTPDSPMTTLRLDSLKANAGARRRKMRKGRGIAAGQGASCGF

GMRGQKSRSGRPTRPGFEGGQMPLYRRVPKLKHFTTVNSKEFTVVNVAALNELKTGSTINLDTLVKNGVV

TSPKYPLKVLGNGELKVKLTIQAAAFTATARNKIEAAGGTCEILD

>gi|123964297|gb|ABM79053.1| 50S ribosomal protein L18 [Prochlorococcus marinus str. MIT 9303]

MSNLSRKQQTQKRHRRLRRHLNGTAQRPRLAVFRSNNHIYAQVIDDEAQNTLCAASTLDKDLRTSLKADG

SSCDASNAVGDLVAKRALAKGIQQVVFDRGGNLYHGRVKSLADAAREAGLQF

>gi|123964295|gb|ABM79051.1| 30S ribosomal protein S8 [Prochlorococcus marinus str. MIT 9303]

MANHDPISDMLTRIRNASEKRHQTTRVPASRMSRSIAKVLQQEGFISEISEEGEGVLTHLVLELKYSGKH

RHPTIRSMQRVSKPGLRIYKNTRALPKVLGGLGMAIISTSKGVMSDRDARKQGVGGEVLCYVY

>gi|123964292|gb|ABM79048.1| 50S ribosomal protein L24 [Prochlorococcus marinus str. MIT 9303]

MPITTPKQKTTQRIKMRIHKGDTVQVITGKDKGKTGEVLRTLPIENRVIVQGVNIRTRHVKPTQEGESGR

IVTEEASVHASNVMLYSNNKKIASRVALVVEKDGSKKRRLKKTGELID

>gi|123964291|gb|ABM79047.1| 50S Ribosomal protein L14 [Prochlorococcus marinus str. MIT 9303]

MIQQESFLTVADNSGAKRIQCIRVLGSNRRYAHVGDIIVAAVKDAMPNMSVKKSEVVKAVVVRTKATLRR

ETGNSIRFDDNAAVLINEDKNPRGTRVFGPVARELRERNFTKIVSLAPEVI

>gi|123964290|gb|ABM79046.1| 30S Ribosomal protein S17 [Prochlorococcus marinus str. MIT 9303]

MALKERLGTVVSDKMDKTVVVAVENRFPHPIYQKTVSRTTRYKAHDAGNTCRIGDRVRITETRPISRSKR

WTVAEVLSHSPKAQEVST

>gi|123964285|gb|ABM79041.1| 30S Ribosomal protein S19 [Prochlorococcus marinus str. MIT 9303]

MGRSLKKGPFIADSLLRKLEKQNADDDKSVIKTWSRASTILPMMIGHTIAVHNGRSHVPVFITEQMVGHK

LGEFAPTRTFKGHIKDKKGGR

>gi|123964284|gb|ABM79040.1| 50S ribosomal protein L2 [Prochlorococcus marinus str. MIT 9303]

MAIRTFRPYTPGTRTRVVTDFNEVTGRKPERSLVVAKHRRKGRNNRGVITCRHRGGGHKRLYRIVDFRRN

KHGIPAKVAAIHYDPHRNAHLALLFYTDGEKRYILAPNGIAIGQQLISGPESPIETGNALPLSAIPLGSS

VHNVELYAGRGGQMARTAGSSAQVMAKEGDYVALKLPSTEVRLVRHECYATLGEVGNSEVRNTSLGKAGR

RRWLGRRPQVRGSVMNPCDHPHGGGEGRAPIGRSGPVTPWGKPALGLKTRKRNKPSNRFVLRKRRRTSKR

SRGGRDS

>gi|123964283|gb|ABM79039.1| 50S ribosomal protein L23 [Prochlorococcus marinus str. MIT 9303]

MTERFNGRLADVIRRPLITEKATSALEQNQYTFEVDHRAAKPDIKAAVEQLFDVRVVGISTMNPPRRSRR

VGRFTGKRAQVKKAIVRLAEGNTIQLFPES

>gi|123964282|gb|ABM79038.1| 50S ribosomal protein L4 [Prochlorococcus marinus str. MIT 9303]

MAECVVHDWQGKEAGKASLELKVSKETTAVDLMHRAVLRQQAHSRQGTASTLTRAEVRGGGRKPYKQKGT

GRARQGTIRTPLRPGGGIIFGPKPRTYNLAMNRKERRLALRTALMARLEDVIVVKDFGDSLKAPKTREIS

DALVRLGVAADAKVLIILSTPSEIIRRSVRNLEKVKLIAADQLNVFDLLHANSLVLSEEALAKIQEVYGD

D

>gi|123964281|gb|ABM79037.1| 50S ribosomal protein L3 [Prochlorococcus marinus str. MIT 9303]

MSIGILGKKLGMSQFFDDQGRAIPVTLIEAGPCRITQLKTSDIDGYAAVQIGFGDTREKLINKPSKGHLT

KSGEVLLKHLREYRVEGLEGLELGAAITVGSFEAGQKVDVSGDTMGRGFSGYQKRHGFSRGPMSHGSKNH

REPGSTGAGTTPGRIYPGKRMAGRYGGKKRTTRGLTILKVDSNRNLLVVKGSVPGKPGALLNIRPAKRVG

SKPAQGGK

>gi|123963941|gb|ABM78697.1| 30S Ribosomal protein S16 [Prochlorococcus marinus str. MIT 9303]

MIKLRLKRFGKKREASFRLVACNSTSRRDGRPLQELGFYNPRTKETRLDTEALRLRLSQGAQPTDAVRSL

LEKGGLIEKTVRPAEVVGKAKQAEARKAGAKNVAKQAAEAKAEETPADNTEA

>gi|123963652|gb|ABM78408.1| 30S ribosomal protein S2 [Prochlorococcus marinus str. MIT 9303]

MAVVTLSEMMEAGAHFGHQTRRWNPKMSRYIYCARNGVHIIDLVQTAICMNNAYKWTRSSARSGKRFLFV

GTKKQASEVVALEATRCGASYVNQRWLGGMLTNWTTMKARIDRLKDLERMESSGAIAMRPKKEASVLRRE

LERLQKYLGGLKGMRRLPDVVVLVDQRRETNAVLEARKLDIPLVSMLDTNCDPDLCEVPIPCNDDAVRSV

QLVLGRLADAINEGRHGTNEQRGADDNDD

>gi|123963573|gb|ABM78329.1| 30S Ribosomal protein S15 [Prochlorococcus marinus str. MIT 9303]

MSLDTTEKQQLINANQTHGTDTGSVEVQVAMLSERITKLSSHLQENKHDFSSRQGLLKMIGRRKRLLSYV

RGKSEQRYNGLITKLGIRG

>gi|123963514|gb|ABM78270.1| 50S ribosomal protein L28 [Prochlorococcus marinus str. MIT 9303]

MSRVCQLTGTRANNGMAVSHSHIRTKKLQQANLQQRRLWWAEGNRWLKLRVSTRALKTIQKKGLGVYAKS

LGIDLNKI

>gi|123963468|gb|ABM78224.1| 30S Ribosomal protein S18 [Prochlorococcus marinus str. MIT 9303]

MSSSFFKKRLSPIKPGDPIDYKDVDLLKKFITDRGKILPRRLTGLTSKQQRDLTNAVKRARIIALLPFVN

PEG

>gi|123962677|gb|ABM77433.1| Ribosomal protein L19 [Prochlorococcus marinus str. MIT 9303]

MTADSKDTSMSEDNTETATTIENSSAMVTDVTSKSATKVRLSADALIQEFEATQQKSDLNDIYVGDTVRV

GVRISEGNKERIQPYEGVVIAKRHGGIHATITVRRIFQGIGVERVFMLHSPQVASIKVERRGKVRRAKLF

YLRERVGKATRVKQRFDR

>gi|123962599|gb|ABM77355.1| 30S Ribosomal protein S14 [Prochlorococcus marinus str. MIT 9303]

MAKKSMIARDVKRKKIVERYAAKRAALMAAFDAAKDPMQRLEIHRKIQALPRNSAPTRIRNRCWATGKPR

GVYRDFGLCRNQLRERAHKGELPGVVKSSW

>gi|123962537|gb|ABM77293.1| 50S ribosomal protein L21 [Prochlorococcus marinus str. MIT 9303]

MAEKPAAKPKAAAAKAEAKDQSDSYAIVEASGQQFWLQPNRYYDLDRLQAAVDDTVTLENVLLIKDGKND

TTVGQPYVKGASVELKVMDHRRGPKIIVYKMRPKKKTRRKNGHRQELTRVMVQSISIDGKALS

>gi|123962536|gb|ABM77292.1| 50S ribosomal protein L27 [Prochlorococcus marinus str. MIT 9303]

MAHKKGTGSTRNGRDSNSKRLGVKAYGGETVTAGSILIRQRGTSVLPGVNVGQGKDDTLFALTDGVVTFE

SIRRSLRNRKRISVVASS

>gi|123962521|gb|ABM77277.1| putative methyltransferase for Ribosomal protein L11 [Prochlorococcus marinus str. MIT 9303]

MNSASACCWWRLALPIADELEESLIWKLTELGISRIAVQHVPEKAERTLLAWLPSSEWSESDRDQLMVNL

RPLAEPFGLQLANPTWCEVADEDWSLNWKQHWQSDPVGQRLLILPAWLDLPQEYADRLVVRMDPGSAFGT

GSHPSTRLCLEALEKNPPLGLRVADLGCGSGVLGFAALGFGARQVLAADTDSQAVCASRANAELNQLDLD

RFRVVHGSVDALSAQLQGEVVDLLLCNILAPVIEALASSFDQLLSANGRCLLSGLLVDQAPRLQVVLEAL

GWRVNSLTVQGCWGLLDVSKR

>gi|123962430|gb|ABM77186.1| 30s Ribosomal protein S20 [Prochlorococcus marinus str. MIT 9303]

MANNKSSKKRVQIAERNRLENKSYKSAMRTLMKRCFSACSNYSQQPGETAKANVKASIDSAFSKIDKAVK

RGVLHRNTAAHQKSRLSAAVKQAIEPAPST

>gi|123962061|gb|ABM76817.1| 50S ribosomal protein L35 [Prochlorococcus marinus str. MIT 9303]

MPKLKTRKAAAKRFKATVTGKFMRRRAFRNHLLDHKSPKLKRHLATKAVVDERDAENVRLMLPYA

>gi|123962060|gb|ABM76816.1| 50S ribosomal protein L20 [Prochlorococcus marinus str. MIT 9303]

MARVKRGNVARKRRNKILRLARGFQGSNGSLFRTANQRVMKALCNAYRDRRRRKRDFRRLWIARINAAAR

INGVSYSRLIGDLKKADVRINRKMLAQLAVMDPKSFTSVVTSAKS

>gi|123964302|gb|ABM79058.1| 50S Ribosomal protein L36 [Prochlorococcus marinus str. MIT 9303]

MKVRASVKKMCEKCRVIRRHGRVMVICSNPKHKQRQG

>gi|123964289|gb|ABM79045.1| 50S ribosomal protein L29 [Prochlorococcus marinus str. MIT 9303]

MAHPKAAEVRKLTDADITEQIDGIRRELFDLRFQQATRQLSNTHRFKESRTKLAQLLTVQKERSRSAAAS

>gi|123964288|gb|ABM79044.1| 50S ribosomal protein L16 [Prochlorococcus marinus str. MIT 9303]

MLSPKRVKFRKQQRGRMRGVATRGNTIAFGEFALQAQECGWITSRQIEASRRAMTRYVKRGGKIWIRIFP

DKPVTMRPAETRMGSGKGNPEFWVAVIKPGRILFEMGGEEITEEIAREAMRLAQYKLPIKTKFIGLDDQE

KVAGSDKPASVPAITAES

>gi|123963469|gb|ABM78225.1| 50S Ribosomal protein L33 [Prochlorococcus marinus str. MIT 9303]

MAKNKGVRIVITLECNECRSNPAIDKRSHGVSRYTTEKNRRNTTERLEIKKFCRYCNKSTTHKEIK

>gi|123963054|gb|ABM77810.1| 30S Ribosomal protein S21 [Prochlorococcus marinus str. MIT 9303]

MAQVTVGENEGVESALRRFKRAVSKAGIFSDLKRIRHHETPVEKYKRKAQQRRRSRRR

>gi|123962608|gb|ABM77364.1| 50S ribosomal protein L34 [Prochlorococcus marinus str. MIT 9303]

MTKRTFGGTSRKRKRVSGFRVRMRTHTGRRVIRSRRKRGRTRLAV

>gi|123964182|gb|ABM78938.1| possible Ribosomal protein S14p/S29e [Prochlorococcus marinus str. MIT 9303]

MKLTKLSQSELDRGCELNNVLKECWKAFYSLTAQMNNVETDSTKYLQLDKLRQTCERNIGEFKHELDELN

WLLRIGIRD

>gi|123963693|gb|ABM78449.1| possible Ribosomal protein L11 [Prochlorococcus marinus str. MIT 9303]

MNTKSKLTMEFSIKQRGVLGGMLTGTAISIGLIIFGILLNPFNFQSNLTLLEKSSVLFKSLILLALCLAF

SIGRLAKHRFFNPDEIDGRGLRTDSDRAIFLQSLLQNTLEQSVLAAFVYGTWTFVMPSAWLSVVPLAALS

FALGRVLFFAGYRRGAVGRAVGFTMAFYPSVLMLICTVCVLPISAVAGGS

>gi|123963447|gb|ABM78203.1| 50S ribosomal protein L32 [Prochlorococcus marinus str. MIT 9303]

MAVPKKKTSKGKRNQRHAIWKAKAATAAQRALSIGKSVLSGRAQGFVYPMQESDDDES

>gi|123963154|gb|ABM77910.1| possible Ribosomal protein L36 [Prochlorococcus marinus str. MIT 9303]

MLKQLVSHSRAEPSNYDKIYKSSSSLYMRRAPISDFILKCAIAGILLCTSTISAEAKDKVIESTTYSYNC

REGLWKSKIIGRGVNDKDWITIERLEELSGTRQTSLRDEIQESCQSSSQIAKAKRAFGDNCELVSDSFDP

GNWQIDYASRFNAHGNKYLIFAIRYDDGSRILCLSKNNFRDNSPIETPGRSLFIHNIERQANSSIIEYEY

HPGNGWGYEVKKYRLDFSDPERPSFSVVDSWIHQR

>gi|123962507|gb|ABM77263.1| possible Ribosomal protein L36 [Prochlorococcus marinus str. MIT 9303]

MTMDRLQRLVLSFYREDPCIEAELEPLLDCRMTRSWGSIRIECVDEEHLEEVSALLTRLRLPLAALGLGR

QIVLRVPGSLQRTYPMHVPFHSDLLA

>gi|123964754|gb|ABM79510.1| Hypothetical protein P9303_27801 [Prochlorococcus marinus str. MIT 9303]

MPWLPRLLWVSTRFLPALPGRFINTPRVARAERLRFDCFSLICCLIQTMSKKTDDILDSLKTLSLLEASE

LVKQIEDAFGVSAAPSAGVVMAAGGGAAGGAAAEAAEEQTEFDVVLESFDASAKIKVLKAVREATGLGLG

DAKAMVEAAPKTIKEGIAKNDAEALKKAIEEVGGKVSLK

>gi|123962985|gb|ABM77741.1| putative ribosomal-protein-alanine acetyltransferase [Prochlorococcus marinus str. MIT 9303]

MLKHDIQAMEVIHLGPEQINACMELNQLALNGLWSKQQWNKELIDSRSLCMGVLKSSALLALACGWLVVD

ELHLTAIGVHPQHRRQGLARLLLSKLLEQGQLTGAVHATLEVARNNSAARGLYESCGFKTAGCRHHYYSN

GQDALIQWRSLEKKAEPRQKI

>gi|123962598|gb|ABM77354.1| polyribonucleotide nucleotidyltransferase [Prochlorococcus marinus str. MIT 9303]

MQGQTQSISFDGREIRLTTGRYAPQAGGSVMMECGDTSVLVTATRSTGREGIDFLPLICDYEERLYAAGR

IPGSFMRREGRPPERATLIARLIDRPMRPLFPSWMRDDLQIVATCLSLDERVPADVLAVTGASMATLLAG

IPFQGPMAAVRVGLLGDDFVLNPSYREIERGDLDLVVAGTPDGVVMVEAGANQLPEGDVIEAIDFGYEAV

CELIKAQQSILKDAGIKQVQPEPPTQDTKLSTYLEKNCSKSIGEVLKQFEQTKAERDSKLDAIKAKTAEA

IDSLKEDDAVRKSVNANSKVLSNNFKALTKKLMREQIIKQGKRVDGRKLDEVRTITSAAGVLPKRVHGSG

LFQRGLTQVLSTATLGTPSDAQEMDDLNPGPEKTYLHHYNFPPYSVGETRPMRSPGRREVGHGSLAERAI

IPVLPPKDTFPYVLRVVSEVLSSNGSTSMGSVCGSTLALMDAGVPLKAPVSGAAMGLIKEDAEIRILTDI

QGIEDFLGDMDFKVAGTKDGITALQMDMKITGLPVKTIAEAVNQARPARIHILEKMLEAIDAPRTSLSPH

APRLLSFRIDPELIGTVIGPGGRTIKGITERTNTKIDIEDGGIVTIASHDGAAAEAAQRIIEGLTRKVNE

GEVFSGTITRIIPIGAFVEILPGKEGMIHISQLSEARVEKVDDVVKVGDEVTVRIREIDNRGRINLTLRG

VPQNGEETQSEPAPTPVAPLN

>gi|123962411|gb|ABM77167.1| N utilization substance protein A [Prochlorococcus marinus str. MIT 9303]

MALVLLPGLNNLIEDISEEKKLPTQVVEAALREALLKGYERYRRTLYLGISEDPFEEEYFSNFDVGLELD

DEGYRVLASKIIVEEVESEDHQIALQEVMQVAEDAQIGDTVVLDVTPEKEDFGRMAAATTKQVLAQKLRD

QQRRMIQEEFADLEDPVLTARVIRFERHSVIMAVSSGLGRPEVEAELPRRDQLPNDNYRANATFKVFLKE

VSEVPRRGPQLFVSRSNAGLVVYLFENEVPEIQEGSVRIVAVAREANPPSRSVGPRTKVAVDSIEREVDP

VGACIGARGSRIQQVVNELRGEKIDVIRWSPDPGQYIANSLSPARVEMVRLVDPEGQHAHVLVPPDQLSL

AIGREGQNVRLAARLTGWKIDIKNSQEYDQASEDTTVAELISQREEEEALQRDAESRLAAEQATRAEEDA

RLRELYPLPEDEEEYDQEEPAKTMAEDENASDADGQPDDLSSQPDTSSEQLSNEESVEEEDRAR

>gi|123963077|gb|ABM77833.1| Hypothetical protein P9303_10841 [Prochlorococcus marinus str. MIT 9303]

MTSKHPITEIENNLFSFLKLFNHWRRTNIHDTKEMLMLKTNIPCPIFNSISRADLSTDVIEETLDKLINE

YQSASLPILWWISPSTRPADLGQRLLARGFQVEKSIGMACNLRNEYPHDSPRLQLSIRRVKNNNELKTWC

YVMCTAFGMSNDYIEYFYELFSSIGLDSYNTCRHYLAFDKDKAVATSTMLINDSVAGFYNIATIEEFRNM

GIGSAMMNALITESISKKCKLSILHASQSGFNLYKSFGFNNYCNITQYSLI

>gi|123962820|gb|ABM77576.1| possible acetyltransferase [Prochlorococcus marinus str. MIT 9303]

MISSCSLTPEVLEQAYGHGARECPSSNEQINLVFSQDRSFDLVELEQLLEAVGWSRRPMRRVRLALDHTL

LKVGLWRHDPLLPRLVGFARCTGDGVLEATVWDVAIHPIYQGVGLGKHLMDYTLESLKEMGVKRVTLFAD

PGVVDFYERQGWTLEPDGHKCAFWYA

>gi|123962215|gb|ABM76971.1| possible Fe-S oxidoreductase [Prochlorococcus marinus str. MIT 9303]

MTKPALRSDIPMKPTAHKQEKPSVAFAHLGCEKNRVDTEHMLGLLTEAGYSVSSDENDAAVVVVNTCSFI

QDAREESVRTLIGLAEQGKELIIAGCLAQHFQEELLESIPEAKAIVGTGDYQHIVDVLKRVEAGERVNRV

SAFPTFVGDETLPRQRTTDQAVAYLKVAEGCDYRCAFCIIPKLRGDQRSRPVESIVAEAHQLAEQGVQEL

ILISQITTNYGLDLYGKPKFAELLQALGEVDIPWVRVHYAYPTGLTPEVLAAYREVPNVLRYLDLPLQHS

HPDVLRAMNRPWQTDVNERLLDRIREQLPDAVLRTTLIVGFPGETEDHFNHLAAFIERQRFDHVGVFTFS

PEDGTAAADLPNRVDPSIAAARKDRLMALQQPISAERNQRWVGRTIDVLIEQHNPETGAMIGRCDRFAPE

VDGEVLVLPSEKGLQASPGTMVPVFITGSDVYDLTGQLVDTNAMAVTAQTSQ

>gi|166230048|sp|A2CDE9.1|RL10_PROM3 RecName: Full=50S ribosomal protein L10

MGRTLESKQQIVEELKGLLGEAEMALVLDYQGLSIKEMSDLRTRLQASNGVCKVTKNTLMRHAINGNGAW

SNLESLLTGTNAFVLIKGDVGGAVKAVQAFQKDTKKSETKGGLFEGKLLSQGEIKAIGDLPTKEVLMAQI

AGSLNALATKVAVGINEVPSGLARALHQHAESGES

>gi|189045430|sp|A2CC44.1|RS5_PROM3 RecName: Full=30S ribosomal protein S5

MTQPNTQTTPNDVPAAAEGQHQEQQQQQRRGGGRERRGGGRRGDRRGQERDSEWQERVVQIRRVSKTVKG

GKKMSFRAIVVVGNERGQVGVGVGKAGDVIGAVRKGVADGKKHLVKVPLTRHNSIPTLSNGREGAANVLI

RPAAPGTGVIAGGSIRTVLELAGIKNVLAKRLGSKTPLNNARAAMVALASLRTHKETAKERGISLEQIYS

>gi|166223842|sp|A2CDR6.1|RL9_PROM3 RecName: Full=50S ribosomal protein L9

MAKRVQVVLNEDVLSLGKDGDLVEVAPGYARNFLLPFGKAVPVTPAVMKQVGHRRAKQAEHQAAIKQEAL

DFQTALVTIGRFTVKKQTGEDDVLFGTVTNGDVAEAIETATKKEIDRRNIIVPEIHRTGSYKVQVKLHNE

VNAEINLEVVSY

>gi|166216365|sp|A2CC40.1|RL5_PROM3 RecName: Full=50S ribosomal protein L5

MSLKQRYRETIQPKLLKDLSLSNIHEVPKVLKITVNRGLGEAAQNAKSLEASITELATITGQKVVVTRAK

KAIAGFKIRQGMPIGCAVTLRGERMYAFLERLINLALPRIRDFRGVSPKSFDGRGNYTLGVREQLIFPEV

SFDKIDAIRGMDITIVTSARTDEEGQSLLREMGMPFRSN

>gi|160166817|sp|A2CDE8.2|RL1_PROM3 RecName: Full=50S ribosomal protein L1

MPKLSKRITGLLAKVEDRVYQPIEAIQLVKENATAKFDETIEAHVRLGIDPKYTDQQLRTTVALPQGTGQ

SVRIAVITRGEKLAEAKTAGAELAGDDDLVESIGKGQMDFDLLIATPDMMPKVAKLGRVLGPRGLMPNPK

AGTVTTDLAAAIKEFKAGKLEFRADRAGIVHVRFGKASFSADALLENLKTLQETIDRNKPSGAKGRYWKS

LYITSTMGPSVEVDVTALQDIEEDA

>gi|152060893|sp|A2CC33.1|RS3_PROM3 RecName: Full=30S ribosomal protein S3

MGNKIHPTGLRLGITQEHRSRWYATSKMYPILLQEDDRIRRFIHKKYGAAGISDVLIARKADQLEVELKT

ARPGVLVGRQGSGIEELRTGIQKTIGDHSRQVRINVVEVERVDADAFLLAEYIAQQLEKRVAFRRTIRMA

VQRAQRAGVLGLKIQVGGRLNGAEIARNEWTREGRVPLHTLRAEIDYATKVASTTYGVLGIKVWIFKGEV

LGDEAQSMPVGASPRRRGNRRPQQFEDRSNEG

>gi|215274853|sp|A2CC32.1|RL22_PROM3 RecName: Full=50S ribosomal protein L22

MTTSSPTTTTIAKAHGRFIRGSVSKVRRVLDQIRGRTYRDALIMLEFMPYRSTGPITKVLRSAVANAEHN

LGLDPASLVIAQASADMGPSMKRYRPRAQGRAFAIKKQTCHISIAVAAQTDS

>gi|152112246|sp|A2CBJ1.1|RS4_PROM3 RecName: Full=30S ribosomal protein S4

MSRYRGPRLRITRRLGDLPGLTRKAAKRSHPPGQHGQARRKRSEYAIRLEEKQKLRFNYGISERQLVRYV

KKARAQDGSTGTNLLKLLENRLDNVCFRLGFGPTVPGARQLVNHGHVTVNGRVLDIASYQCKAGDVVAIR

ERKGSKKLAEANLEFPGLANVPPHIELDKAKMSAKIISKCEREWVALEINELLVVEYYSRKV

>gi|166199807|sp|A2C7A4.1|RL34_PROM3 RecName: Full=50S ribosomal protein L34

MTKRTFGGTSRKRKRVSGFRVRMRTHTGRRVIRSRRKRGRTRLAV

>gi|226708151|sp|A2CC56.1|RL31_PROM3 RecName: Full=50S ribosomal protein L31

MPKPDIHPNWYPDAKVICNGEVVMTTGSTQPELHVDVWSGNHPFFTGTQKILDTEGRVDRFMRKYGMGSA

DAAADEKKTDAKNNNKDNTSKED

>gi|226731533|sp|A2C795.1|RS14_PROM3 RecName: Full=30S ribosomal protein S14

MAKKSMIARDVKRKKIVERYAAKRAALMAAFDAAKDPMQRLEIHRKIQALPRNSAPTRIRNRCWATGKPR

GVYRDFGLCRNQLRERAHKGELPGVVKSSW

>gi|218547106|sp|A2CC38.1|RL24_PROM3 RecName: Full=50S ribosomal protein L24

MPITTPKQKTTQRIKMRIHKGDTVQVITGKDKGKTGEVLRTLPIENRVIVQGVNIRTRHVKPTQEGESGR

IVTEEASVHASNVMLYSNNKKIASRVALVVEKDGSKKRRLKKTGELID

>gi|166987298|sp|A2CC29.1|RL23_PROM3 RecName: Full=50S ribosomal protein L23

MTERFNGRLADVIRRPLITEKATSALEQNQYTFEVDHRAAKPDIKAAVEQLFDVRVVGISTMNPPRRSRR

VGRFTGKRAQVKKAIVRLAEGNTIQLFPES

>gi|166233177|sp|A2CC27.1|RL3_PROM3 RecName: Full=50S ribosomal protein L3

MSIGILGKKLGMSQFFDDQGRAIPVTLIEAGPCRITQLKTSDIDGYAAVQIGFGDTREKLINKPSKGHLT

KSGEVLLKHLREYRVEGLEGLELGAAITVGSFEAGQKVDVSGDTMGRGFSGYQKRHGFSRGPMSHGSKNH

REPGSTGAGTTPGRIYPGKRMAGRYGGKKRTTRGLTILKVDSNRNLLVVKGSVPGKPGALLNIRPAKRVG

SKPAQGGK

>gi|166232683|sp|A2CC37.1|RL14_PROM3 RecName: Full=50S ribosomal protein L14

MIQQESFLTVADNSGAKRIQCIRVLGSNRRYAHVGDIIVAAVKDAMPNMSVKKSEVVKAVVVRTKATLRR

ETGNSIRFDDNAAVLINEDKNPRGTRVFGPVARELRERNFTKIVSLAPEVI

>gi|166230736|sp|A2C9R5.1|RL33_PROM3 RecName: Full=50S ribosomal protein L33

MAKNKGVRIVITLECNECRSNPAIDKRSHGVSRYTTEKNRRNTTERLEIKKFCRYCNKSTTHKEIK

>gi|166229586|sp|A2CC55.1|RS9_PROM3 RecName: Full=30S ribosomal protein S9

MSSSNNSVVYWGTGRRKTSVARVRLVPGTGTITINGRPGDHYLNFNPAYLAAVKAPLQTLGLNEQYDVLV

NVHGGGLTGQADAIKQGAARALCELSADNRKPLKTEGHLSRDPRAKERRKYGLKKARKAPQFSKR

>gi|166227364|sp|A2CE27.1|RS6_PROM3 RecName: Full=30S ribosomal protein S6

MSQQPYYETMYILRPDIPEEEVETHVTKYREMVTEAGAEVLDNQMRGKRRLAYPISNHKEGIYVQLSHNG

NGQQVAVLEKAMRLSEDVIRYLTVKQEGPLPAPRIVPGSEPEPVEQQEAAAVEA

>gi|166226600|sp|A2C9W0.1|RL28_PROM3 RecName: Full=50S ribosomal protein L28

MSRVCQLTGTRANNGMAVSHSHIRTKKLQQANLQQRRLWWAEGNRWLKLRVSTRALKTIQKKGLGVYAKS

LGIDLNKI

>gi|166225031|sp|A2C732.1|RL27_PROM3 RecName: Full=50S ribosomal protein L27

MAHKKGTGSTRNGRDSNSKRLGVKAYGGETVTAGSILIRQRGTSVLPGVNVGQGKDDTLFALTDGVVTFE

SIRRSLRNRKRISVVASS

>gi|166224052|sp|A2C8K0.1|RS21_PROM3 RecName: Full=30S ribosomal protein S21

MAQVTVGENEGVESALRRFKRAVSKAGIFSDLKRIRHHETPVEKYKRKAQQRRRSRRR

>gi|166223967|sp|A2C6S6.1|RS20_PROM3 RecName: Full=30S ribosomal protein S20

MANNKSSKKRVQIAERNRLENKSYKSAMRTLMKRCFSACSNYSQQPGETAKANVKASIDSAFSKIDKAVK

RGVLHRNTAAHQKSRLSAAVKQAIEPAPST

>gi|166219720|sp|A2CC36.1|RS17_PROM3 RecName: Full=30S ribosomal protein S17

MALKERLGTVVSDKMDKTVVVAVENRFPHPIYQKTVSRTTRYKAHDAGNTCRIGDRVRITETRPISRSKR

WTVAEVLSHSPKAQEVST

>gi|166219678|sp|A2C5Q6.1|RL20_PROM3 RecName: Full=50S ribosomal protein L20

MARVKRGNVARKRRNKILRLARGFQGSNGSLFRTANQRVMKALCNAYRDRRRRKRDFRRLWIARINAAAR

INGVSYSRLIGDLKKADVRINRKMLAQLAVMDPKSFTSVVTSAKS

>gi|166218718|sp|A2CC43.1|RL18_PROM3 RecName: Full=50S ribosomal protein L18

MSNLSRKQQTQKRHRRLRRHLNGTAQRPRLAVFRSNNHIYAQVIDDEAQNTLCAASTLDKDLRTSLKADG

SSCDASNAVGDLVAKRALAKGIQQVVFDRGGNLYHGRVKSLADAAREAGLQF

>gi|166201333|sp|A2CB37.1|RS16_PROM3 RecName: Full=30S ribosomal protein S16

MIKLRLKRFGKKREASFRLVACNSTSRRDGRPLQELGFYNPRTKETRLDTEALRLRLSQGAQPTDAVRSL

LEKGGLIEKTVRPAEVVGKAKQAEARKAGAKNVAKQAAEAKAEETPADNTEA

>gi|166199842|sp|A2CC28.1|RL4_PROM3 RecName: Full=50S ribosomal protein L4

MAECVVHDWQGKEAGKASLELKVSKETTAVDLMHRAVLRQQAHSRQGTASTLTRAEVRGGGRKPYKQKGT

GRARQGTIRTPLRPGGGIIFGPKPRTYNLAMNRKERRLALRTALMARLEDVIVVKDFGDSLKAPKTREIS

DALVRLGVAADAKVLIILSTPSEIIRRSVRNLEKVKLIAADQLNVFDLLHANSLVLSEEALAKIQEVYGD

D

>gi|166199818|sp|A2C5Q7.1|RL35_PROM3 RecName: Full=50S ribosomal protein L35

MPKLKTRKAAAKRFKATVTGKFMRRRAFRNHLLDHKSPKLKRHLATKAVVDERDAENVRLMLPYA

>gi|160358606|sp|A2CC30.1|RL2_PROM3 RecName: Full=50S ribosomal protein L2

MAIRTFRPYTPGTRTRVVTDFNEVTGRKPERSLVVAKHRRKGRNNRGVITCRHRGGGHKRLYRIVDFRRN

KHGIPAKVAAIHYDPHRNAHLALLFYTDGEKRYILAPNGIAIGQQLISGPESPIETGNALPLSAIPLGSS

VHNVELYAGRGGQMARTAGSSAQVMAKEGDYVALKLPSTEVRLVRHECYATLGEVGNSEVRNTSLGKAGR

RRWLGRRPQVRGSVMNPCDHPHGGGEGRAPIGRSGPVTPWGKPALGLKTRKRNKPSNRFVLRKRRRTSKR

SRGGRDS

>gi|158706261|sp|A2CC49.1|RS13_PROM3 RecName: Full=30S ribosomal protein S13

MARIAGVDIPRDKRVEVALTYIYGIGLTRAKTILTKSGVNPDIRVKDLEDGDVQKLRTALEAFTIEGDLR

RQEGMALKRLQDIGCLRGRRHRMSLPVRGQRTRTNARTRRGARKTVAGKKK

>gi|158512756|sp|A2CC48.1|RL36_PROM3 RecName: Full=50S ribosomal protein L36

MKVRASVKKMCEKCRVIRRHGRVMVICSNPKHKQRQG

>gi|152060807|sp|A2CC50.1|RS11_PROM3 RecName: Full=30S ribosomal protein S11

MAKPTKKTGSKKTKRNVPNGVAHIQSTFNNTIVSITDTAGEVIAWSSAGASGFKGARKGTPFAAQTAAEA

AARRALEQGMRQIEVLVRGPGSGRETAIRALQVAGLEITLIRDVTPLPHNGCRRPKRRRV

>gi|148841161|sp|A2CC41.1|RS8_PROM3 RecName: Full=30S ribosomal protein S8

MANHDPISDMLTRIRNASEKRHQTTRVPASRMSRSIAKVLQQEGFISEISEEGEGVLTHLVLELKYSGKH

RHPTIRSMQRVSKPGLRIYKNTRALPKVLGGLGMAIISTSKGVMSDRDARKQGVGGEVLCYVY

>gi|166986915|sp|A2C733.1|RL21_PROM3 RecName: Full=50S ribosomal protein L21

MAEKPAAKPKAAAAKAEAKDQSDSYAIVEASGQQFWLQPNRYYDLDRLQAAVDDTVTLENVLLIKDGKND

TTVGQPYVKGASVELKVMDHRRGPKIIVYKMRPKKKTRRKNGHRQELTRVMVQSISIDGKALS

>gi|166228460|sp|A2CC85.1|RS7_PROM3 RecName: Full=30S ribosomal protein S7

MSRRNAAEKRPVLPDPQFNNRLATMMISRLMKHGKKSTAQRILAQAFGLINERTGGDPIELFETAVKNAT

PLVEVRARRVGGATYQVPMEVRQERGTAMALRWLVNFSRARNGRSMSQKLAAELMDAANEAGSAVRKREE

THKMAEANKAFAHYRY

>gi|166225295|sp|A2CA98.1|RS2_PROM3 RecName: Full=30S ribosomal protein S2

MAVVTLSEMMEAGAHFGHQTRRWNPKMSRYIYCARNGVHIIDLVQTAICMNNAYKWTRSSARSGKRFLFV

GTKKQASEVVALEATRCGASYVNQRWLGGMLTNWTTMKARIDRLKDLERMESSGAIAMRPKKEASVLRRE

LERLQKYLGGLKGMRRLPDVVVLVDQRRETNAVLEARKLDIPLVSMLDTNCDPDLCEVPIPCNDDAVRSV

QLVLGRLADAINEGRHGTNEQRGADDNDD

>gi|166220972|sp|A2C9R4.1|RS18_PROM3 RecName: Full=30S ribosomal protein S18

MSSSFFKKRLSPIKPGDPIDYKDVDLLKKFITDRGKILPRRLTGLTSKQQRDLTNAVKRARIIALLPFVN

PEG

>gi|166199902|sp|A2CC31.1|RS19_PROM3 RecName: Full=30S ribosomal protein S19

MGRSLKKGPFIADSLLRKLEKQNADDDKSVIKTWSRASTILPMMIGHTIAVHNGRSHVPVFITEQMVGHK

LGEFAPTRTFKGHIKDKKGGR

>gi|166234343|sp|A2CA19.1|RS15_PROM3 RecName: Full=30S ribosomal protein S15

MSLDTTEKQQLINANQTHGTDTGSVEVQVAMLSERITKLSSHLQENKHDFSSRQGLLKMIGRRKRLLSYV

RGKSEQRYNGLITKLGIRG

>gi|166231310|sp|A2CC89.1|RS10_PROM3 RecName: Full=30S ribosomal protein S10

MSTAIAQQKIRIRLKAFDRRMLDLSCDKIIETADNTAATAIGPIPLPTKRKIYCVLCSPHVDKDSREHFE

TRTHRRIIDIYNPSAKTIDALMKLDLPSGVDIEVKL

>gi|166231011|sp|A2CC54.1|RL13_PROM3 RecName: Full=50S ribosomal protein L13

MNKTSVPSIDSIERQWFLVDAENQTLGRLATEVASVLRGKNKPSFTPHLDTGDFVVVVNADKIRVSGNKA

NQKLYRRHSGRPGGMKVETFQALQDRLPERIVEKAIKGMLPHNALGRQLFRKLKVYRGPEHPHSAQRPQT

LQLNPAASSQ

>gi|166216202|sp|A2CC52.1|RL17_PROM3 RecName: Full=50S ribosomal protein L17

MRHQCRVPKLGRPTDQRKAMLRGLTTQLIREGRVTTTKARAKALRDEAERMITLAKNGSLASRRRAIGYI

YDKQLVHALFDKAQDRYGDRQGGYTRIIRTVPRRGDNAEMAIIELV

>gi|156637362|sp|A2CC84.1|RS12_PROM3 RecName: Full=30S ribosomal protein S12

MPTIQQLIRTERQHLTRKTKSPALRACPERRGVCTRVYTSTPKKPNSALRKVARVRLTSGFEVTAYIPGI

GHNLQEHSVVLIRGGRVKDLPGVRYHIIRGTLDTAGVKDRSQSRSKYGAKASKQD

>gi|166228241|sp|A2CC35.1|RL29_PROM3 RecName: Full=50S ribosomal protein L29

MAHPKAAEVRKLTDADITEQIDGIRRELFDLRFQQATRQLSNTHRFKESRTKLAQLLTVQKERSRSAAAS

>gi|166199703|sp|A2CC34.1|RL16_PROM3 RecName: Full=50S ribosomal protein L16

MLSPKRVKFRKQQRGRMRGVATRGNTIAFGEFALQAQECGWITSRQIEASRRAMTRYVKRGGKIWIRIFP

DKPVTMRPAETRMGSGKGNPEFWVAVIKPGRILFEMGGEEITEEIAREAMRLAQYKLPIKTKFIGLDDQE

KVAGSDKPASVPAITAES

>gi|156630852|sp|A2C9P3.1|RL32_PROM3 RecName: Full=50S ribosomal protein L32

MAVPKKKTSKGKRNQRHAIWKAKAATAAQRALSIGKSVLSGRAQGFVYPMQESDDDES

>gi|238066589|sp|A2C661.1|RIMO_PROM3 RecName: Full=Ribosomal protein S12 methylthiotransferase RimO; Short=S12 MTTase; Short=S12 methylthiotransferase; AltName: Full=Ribosomal protein S12 (aspartate-C(3))-methylthiotransferase; AltName: Full=Ribosome maturation factor RimO

MTKPALRSDIPMKPTAHKQEKPSVAFAHLGCEKNRVDTEHMLGLLTEAGYSVSSDENDAAVVVVNTCSFI

QDAREESVRTLIGLAEQGKELIIAGCLAQHFQEELLESIPEAKAIVGTGDYQHIVDVLKRVEAGERVNRV

SAFPTFVGDETLPRQRTTDQAVAYLKVAEGCDYRCAFCIIPKLRGDQRSRPVESIVAEAHQLAEQGVQEL

ILISQITTNYGLDLYGKPKFAELLQALGEVDIPWVRVHYAYPTGLTPEVLAAYREVPNVLRYLDLPLQHS

HPDVLRAMNRPWQTDVNERLLDRIREQLPDAVLRTTLIVGFPGETEDHFNHLAAFIERQRFDHVGVFTFS

PEDGTAAADLPNRVDPSIAAARKDRLMALQQPISAERNQRWVGRTIDVLIEQHNPETGAMIGRCDRFAPE

VDGEVLVLPSEKGLQASPGTMVPVFITGSDVYDLTGQLVDTNAMAVTAQTSQ

>gi|166223428|sp|A2C717.1|PRMA_PROM3 RecName: Full=Ribosomal protein L11 methyltransferase; Short=L11 Mtase

MNSASACCWWRLALPIADELEESLIWKLTELGISRIAVQHVPEKAERTLLAWLPSSEWSESDRDQLMVNL

RPLAEPFGLQLANPTWCEVADEDWSLNWKQHWQSDPVGQRLLILPAWLDLPQEYADRLVVRMDPGSAFGT

GSHPSTRLCLEALEKNPPLGLRVADLGCGSGVLGFAALGFGARQVLAADTDSQAVCASRANAELNQLDLD

RFRVVHGSVDALSAQLQGEVVDLLLCNILAPVIEALASSFDQLLSANGRCLLSGLLVDQAPRLQVVLEAL

GWRVNSLTVQGCWGLLDVSKR

>gi|171769889|sp|A2CCX2.2|RIMM_PROM3 RecName: Full=Ribosome maturation factor RimM

MNGDEDWLTVGKLVAAQGMQGELRINPSSDFPERFTLPGQRWLKERNGEPRPIELLTGRQLPGRSLYVVK

FAGVNNRNAAEALVGQNLLVPSSDRPSLAEGEFHLLDLVGLEARLQAEGPAIGHVIDLTTAGNDLLEIEL

LAGRRVLVPFVEAIVPEVQLNQGWLRLTPPPGLLEL

>gi|187610312|sp|A2C794.1|PNP_PROM3 RecName: Full=Polyribonucleotide nucleotidyltransferase; AltName: Full=Polynucleotide phosphorylase; Short=PNPase

MQGQTQSISFDGREIRLTTGRYAPQAGGSVMMECGDTSVLVTATRSTGREGIDFLPLICDYEERLYAAGR

IPGSFMRREGRPPERATLIARLIDRPMRPLFPSWMRDDLQIVATCLSLDERVPADVLAVTGASMATLLAG

IPFQGPMAAVRVGLLGDDFVLNPSYREIERGDLDLVVAGTPDGVVMVEAGANQLPEGDVIEAIDFGYEAV

CELIKAQQSILKDAGIKQVQPEPPTQDTKLSTYLEKNCSKSIGEVLKQFEQTKAERDSKLDAIKAKTAEA

IDSLKEDDAVRKSVNANSKVLSNNFKALTKKLMREQIIKQGKRVDGRKLDEVRTITSAAGVLPKRVHGSG

LFQRGLTQVLSTATLGTPSDAQEMDDLNPGPEKTYLHHYNFPPYSVGETRPMRSPGRREVGHGSLAERAI

IPVLPPKDTFPYVLRVVSEVLSSNGSTSMGSVCGSTLALMDAGVPLKAPVSGAAMGLIKEDAEIRILTDI

QGIEDFLGDMDFKVAGTKDGITALQMDMKITGLPVKTIAEAVNQARPARIHILEKMLEAIDAPRTSLSPH

APRLLSFRIDPELIGTVIGPGGRTIKGITERTNTKIDIEDGGIVTIASHDGAAAEAAQRIIEGLTRKVNE

GEVFSGTITRIIPIGAFVEILPGKEGMIHISQLSEARVEKVDDVVKVGDEVTVRIREIDNRGRINLTLRG

VPQNGEETQSEPAPTPVAPLN

>gi|78712414|gb|ABB49591.1| SSU ribosomal protein S1P [Prochlorococcus marinus str. MIT 9312]

MGVSNKNAQDNIQPKGNKKNFKKPLQVLHISKKDTQKKSQEIQNEQNNSQEEIKKENIAIKPQIIKDESV

NEIEDSNENTKAFDSSQQNLNRPLNFSEQNTDFQLERTVDEFDFDESAFLEALNENEPIGATGETISGKV

IAIESDGLYVDIGGKAPGYMPKKECGLGVITNFKEKFSIGLEMEVLVIKEQNADGMVTVSARALILRQSW

EKVSNSAKNGELIDVLINGFNRGGLTCDVDGLRGFIPRSQLEDGQDYQSFVGKTLKVAFLEVNPESRKLV

LSEKKASLVSKLTSLDLGQLIEGEVLAVKPYGFFIDLGGASGLLHQSSLTKGSIRSLREIFREGEIIKAL

ISEIDLEKGRIGLNTALLENSAGELIIDKEKVMREATDRALKTKALFDKKNQDK

>gi|78712202|gb|ABB49379.1| SSU ribosomal protein S1P [Prochlorococcus marinus str. MIT 9312]

MQQMNENSSQTTKELSENKEFKNSPELDNNSMPKNEEDLSFEKSDIPSADASSSRTNTDLDNAGFTQEEF

ASLLGKYDYNFKLGDLVKGTVFALEPKGAMIDIGAKTAAFMPVQEVSINRVEGLDDVLQPSESREFFIMS

EENEDGQLALSIRRIEYQRAWERVRQLQKEDATIYSEVFATNRGGALVRVEGLRGFIPGSHISARRVKDD

LEGEYLPLKFLEVDEERNRLVLSHRRALVEKKMNRLEVGEVVVGSVKGIKPYGAFIDIGGVSGLLHISEI

SHEHIETPHNVLNVSDQMKVMIIDLDSERGRISLSTKALEPEPGDMLTDPQKVFSKAEEMAAKYKQMLFE

QTDEAEETASASSETV

>gi|78713649|gb|ABB50826.1| LSU ribosomal protein L9P [Prochlorococcus marinus str. MIT 9312]

MAKRVKVALTESIASLGKEGDLVEVAPGYARNFLLPYGKAMNVTPAVLKQIERKKEKEKIAADKLKQEAL

DFQTALSTIGRFTIKKQVGEDGVLFGTVTNGDVAEAIEAATKKEIDRRNITVPDIHNLGSFTAKIKLHPD

VNAEVNIEVTS

>gi|78713520|gb|ABB50697.1| LSU ribosomal protein L6P [Prochlorococcus marinus str. MIT 9312]

MSRIGKTPVLIPEKVTVDLDGLIVTVKGPKGELKRLMPEGVSFDKKDNTVVVSPTTNKIHSRQRHGLCRA

LIANMVEGVTQGFSKKLEIVGVGSRAQVKGKNLVVSAGYSHPVEMIPPDGITYKVESNTNVTVSGIDKEI

VGNEAAKIRSIRPPEPYKGKGIKYHDERILRKAGKSGKK

>gi|78713518|gb|ABB50695.1| SSU ribosomal protein S5P [Prochlorococcus marinus str. MIT 9312]

MTDTPTKQEITSKNDKVPGAIPGEQKKNNRNNDRKRNRRGDSKNLERDSDWQERVVQIRRVSKTVKGGKK

MSFRAIVVVGNEKGQVGVGVGKAGDVIGAVRKGVSDGKKNLVRVPLTPNNSIPTLSKGRDGAANVLIRPA

APGTGVIAGGSIRTVLELAGIKNVLAKRLGSKTPLNNARAAMVALSQLRTHKSASRERGISLEQLYS

>gi|78712091|gb|ABB49268.1| LSU ribosomal protein L11P [Prochlorococcus marinus str. MIT 9312]

MAKKIVAVIKLALQAGKANPAPPVGPALGQHGVNIMAFCKEYNAKTQDKAGFVIPVEISVFEDRSFTFIT

KTPPAAVLITKAAGIDKGSGESSKGSVGNISKAQLEEIAKTKLPDLNCSSVESAMKVIEGTARNMGVSIT

D

>gi|78712089|gb|ABB49266.1| LSU ribosomal protein L10P [Prochlorococcus marinus str. MIT 9312]

MGRTLENKQQIVTEIKSLLNDSEMAVVLDYKGLTIKEMSDLRSRLQTTNGICKVTKNSLMRKAIDGDSNW

NDLESLLTGTNAFVLIKEDVGGAVKAIQSFQKDTKKSETKGALFEGRLLSNSEIKEIASLPSKEVLMAKI

AGALNGVATKIAISINEVPSGLARSLKQHSEKPES

>gi|78713529|gb|ABB50706.1| LSU ribosomal protein L22P [Prochlorococcus marinus str. MIT 9312]

MTNTSETTKTAIAHGNYVRGSASKVRRVLDQIRGRSYRDALIMLEFMPYRSTDPITKVLRSAVANAEHNY

GMDPSTLIISSAWANNGPVMKRYRPRAQGRAFSIKKQTCHISISVESAPTQINAEVQN

>gi|78713528|gb|ABB50705.1| SSU ribosomal protein S3P [Prochlorococcus marinus str. MIT 9312]

MGHKIHPSGLRLGITQEHRSKWFATSKTYPILLQEDFKIRTFIQKKYGAAGISDVLIARKADQLELELKT

ARPGVIVGRQGSGIEELRAGIQKTIGDNTRQVRINVVEVERVDADAFLLAEYISQQLEKRVAFRRTIRMA

LQRAQRAGVLGLKIQVGGRLNGAEIARTEWTREGRVPLHTLRAEIDYATREANTTYGVLGIKVWVFKGEV

LPKEEKTIPVGASPKRKAGRRPQQFEDRSNENS

>gi|78713522|gb|ABB50699.1| LSU ribosomal protein L5P [Prochlorococcus marinus str. MIT 9312]

MTLKNRYKESIRPKLLKDLGLKNIHQVPKVVKVNVNRGLGEAASNSKSLEASLNEMATITGQKALVTRAK

KAIAGFKIREGMPIGCTVTLRGDRMYSFLERFINLALPRIRDFRGVNPKSFDGRGNYTVGVKEQLIFPEI

SFDKIDSIRGMDITIVTSARSDQEGKALLQELGMPFSKN

>gi|78713264|gb|ABB50441.1| SSU ribosomal protein S16P [Prochlorococcus marinus str. MIT 9312]

MIKLRLKRFGKKKEASFRIVACNSTSRRDGRPLQELGFYNPRTKETRLNTEALRIRLTQGAQPTDVVRNL

LEKGGLLEKKERPSIAIGKAKLEKEKVAKAKIKNEENDSSKAESDSNEAES

>gi|78712982|gb|ABB50159.1| [SSU ribosomal protein S18P]-alanine acetyltransferase [Prochlorococcus marinus str. MIT 9312]

MISIKDINEKDIDLCYELDSNTISLWSKKQWANEFKKEGIKVFGLLLSNLVIGICVFHVVLDEAQINFFV

VNQKYRERGFGSYLMSYLIKQCEKLNINKLFLEVSQTNVVAEKFYSRFDFCTVGIRRNYYKDGSDALLKE

KKLTTK

>gi|78712289|gb|ABB49466.1| SSU ribosomal protein S4P [Prochlorococcus marinus str. MIT 9312]

MSRYRGPRLRVTRRLGELPGLTRKASKKSNPPGQHGQARRKRSEYAIRLEEKQKLRFNYGVSEKQLVRYV

KKARAQEGSTGTNLLRLLENRLDNVCFRLGFGGTIPGSRQLVNHGHVTVNGKVLDIAGYQCKSGDVIGIK

ENKASKKLVEGNIEFPGLANVPPHLDLDKPKLTGKITGKCDREWVALEINELLVVEYYSRKV

>gi|78712090|gb|ABB49267.1| LSU ribosomal protein L1P [Prochlorococcus marinus str. MIT 9312]

MKKLSKRMAALSTKIEDRIYPPLEALSIIKENANAKFDETIEAHIRLGIDPKYTDQQLRTTVALPHGTGQ

SIKIAVITSGENVSKAKSAGADLFGEEDLVESINKGNMDFDLLIATPDMMPKVAKLGRVLGPRGLMPNPK

AGTVTNDIANAIKEFKAGKLEFRADKAGIVHVRFGKASFTKEALFDNLKTLQESIDKNKPSGSKGKYWKS

FYVTSTMGPSVQVDISAVQDYQPEG

>gi|78712088|gb|ABB49265.1| LSU ribosomal protein L12P [Prochlorococcus marinus str. MIT 9312]

MSAKTEEILESLKSLSLLEASELVKQIEEAFGVSAAASAGVVMAAPGATGGDGDGGAAEEKTEFDVVLES

FDAAAKIKVLKVVRNATGLGLGDAKALVESAPKTVKEGIAKADAESLKKEIEEAGGKVTLK

>gi|78713683|gb|ABB50860.1| SSU ribosomal protein S6P [Prochlorococcus marinus str. MIT 9312]

MTNQSYYETMYILRPDIAEDEVTNHIDKYNKLLEEFGGTILDSQMRGKRRLAYQIAKHREGIYVQLSHQG

DGQHIFKIEKAMRISEDVIRYMTVKQEGPLPTPKPSNKSSTQSENKDNPETKVESKEEQSVTNSDTSTTK

KDDNEIKENTES

>gi|78713638|gb|ABB50815.1| LSU ribosomal protein L20P [Prochlorococcus marinus str. MIT 9312]

MARVKRGNIARKRRNKILNLAKGFRGGNKNLFRTANQRVMKALCNAYRDRRRRKRDFRRLWISRINASAR

INGTNYSKLINGMKNSEIIINRKMLAQLALNDPKCFEKIVSSVSN

>gi|78713637|gb|ABB50814.1| LSU ribosomal protein L35P [Prochlorococcus marinus str. MIT 9312]

MSKLKTRKSAAKRFKATATGKFMRRRAYHNHLLDHKSSKLKRHLSTKAVVDERDADNVKLMIPYA

>gi|78713534|gb|ABB50711.1| LSU ribosomal protein L3P [Prochlorococcus marinus str. MIT 9312]

MSIGILGKKLGMSQLFDDKGNAVPVTLIEAGPCRVTQLKTTTLDGYSAVQIGYGLSKDKHINKPEKGHLL

KSGEELLKHLKEYRVEETTSYEIGNQITVKNFEVGQKVDISGKSMGRGFAGYQKRHGFSRGPMSHGSKNH

RAPGSTGAGTTPGRIYPGKRMAGRYGGKQITTKGLLVLKIDDQKNLLVIKGSVPGKPGSIVNIKPNNVVG

KKGGQKS

>gi|78713533|gb|ABB50710.1| LSU ribosomal protein L4P [Prochlorococcus marinus str. MIT 9312]

MTTIETLKWDGKKSGEVKLDLTVAKETSSADLIHRAVLRQLANKRQGTASTLTRSEVRGGGRKPYKQKGT

GRARQGSIRTPLRPGGGIIFGPKPRSYNLDMNRKERRLALRTALMSRISDVKAVEDFGSTLKQPKTSDII

NGLTRLGIQKTEKVLVILDSPSEVIKKSINNIEKVKLIAADQLNVFDILNANKLVIGQSAINKIKEVYAS

>gi|78713532|gb|ABB50709.1| LSU ribosomal protein L23P [Prochlorococcus marinus str. MIT 9312]

MSKLFDSRLADVIRKPVITEKATNALDLNQYTFEVDHRAAKPQIKAAIEALFSVKVIGVNTMNPPRRTRR

VGKFSGKRSQVKKAIVRLAEGDKIQLFPES

>gi|78713531|gb|ABB50708.1| LSU ribosomal protein L2P [Prochlorococcus marinus str. MIT 9312]

MAIRKFKPYTPGTRQRVVTDFSEITSAKPERSLIVSKHRVKGRNNRGVITCRHRGGGHKRQYRLVDFRRD

KRNINAKVAAIHYDPHRNARLALLFYEDGEKRYIIAPAGVKVGQNVISGESVPIEDGNAMPLSVMPLGSS

VHCVELYAGRGAQMVRSAGASAQVMAKEGDYVALKLPSTEVRLVRKECYATLGEVGNSEIRNTSLGKAGR

RRWLGRRPQVRGSVMNPCDHPHGGGEGKAPIGRAGPVTPWGKPALGLKTRKKNKPSNKLVVRRRRRISKR

SRGGRDS

>gi|78713530|gb|ABB50707.1| SSU ribosomal protein S19P [Prochlorococcus marinus str. MIT 9312]

MGRSLKKGPFIADSLLKKVEKQNTDNDKSVIKTWSRASTILPLMIGHTIAVHNGKTHIPVFITEQMIGHK

LGEFAPTRTYRGHIRDKKGAKS

>gi|78713525|gb|ABB50702.1| SSU ribosomal protein S17P [Prochlorococcus marinus str. MIT 9312]

MALKERIGTVVSDKMDKTVVVAVINRYPHPTYKKIVSRTTRYKAHDPENTCALGDRVKIRETRPLSAHKR

WAIQEILNKTSQTKEVKK

>gi|78713524|gb|ABB50701.1| LSU ribosomal protein L14P [Prochlorococcus marinus str. MIT 9312]

MIQQETYLTVADNSGAKRLQCIRVLGSNRRYAHVGDVIVATVKDALPNMGVKKSEVVKAVIVRTKATLRR

NTGNSIRFDDNAAVLINEDKNPKGTRVFGPVARELRDKNYTKIVSLAPEVI

>gi|78713523|gb|ABB50700.1| LSU ribosomal protein L24P [Prochlorococcus marinus str. MIT 9312]

MLDSLKQKKNFQRIKMRIKTGDLVKVINGKDKGKTGEVLKTIPLENRVVVKGINLRTKHVKPTQEGETGR

ILTEEASLHASNVMFFSKDKNLTSKIEYFIDKQGVKKRRLKKTGEVID

>gi|78713521|gb|ABB50698.1| SSU ribosomal protein S8P [Prochlorococcus marinus str. MIT 9312]

MSNHDPISDMLTRIRNASQKKHTTTTIPGSKMSLSIAKVLQKEGFISDINEEGEGYKSQIILSLKYSGKN

KFPTIRSMQRVSKPGLRIYKNTRGLPKVLGGLGVAIISTSKGVMSDRDARKQGIGGEVLCYVY

>gi|78713519|gb|ABB50696.1| LSU ribosomal protein L18P [Prochlorococcus marinus str. MIT 9312]

MTKLSRKLQTQKRHRRLRRSVIGDATRPRLSVFRSNNHIYAQVIDDSAQKTICSASTVDKELREKSDKLP

SDCNSSSIVGKLLANRAIKKGIKQVIFDRGGNLYHGRVKALADAAREAGLEF

>gi|78713517|gb|ABB50694.1| LSU ribosomal protein L15P [Prochlorococcus marinus str. MIT 9312]

MTSTLNTLKSNSGSRKKKLRKGRGIAAGQGASCGFGMRGQKSRSGRPTRPGFEGGQMPLYRRVPKLKHFE

IINQKNFSIINLDKLKDFNDNDTVNLDSLVKKGLIFKPKFPLKILGNGKVNVKLTVQAHAFTKVAKQKIE

DAGGSCELLNNK

>gi|78713514|gb|ABB50691.1| 50S Ribosomal protein L36 [Prochlorococcus marinus str. MIT 9312]

MKVRSSVKKISPDDQIVRRRGKIYVINKKRPRNKQRQG

>gi|78713513|gb|ABB50690.1| SSU ribosomal protein S13P [Prochlorococcus marinus str. MIT 9312]

MARIAGIDIPREKRVEIALTYVYGIGLTRSKLILANAGVNPDIRVKDLSDSDVQKLRGATEEFTLEGDLR

RKEGMALKRLQDIGCVRGRRHRMSLPVRGQRTRTNARTRRGSRKTVAGRKK

>gi|78713512|gb|ABB50689.1| SSU ribosomal protein S11P [Prochlorococcus marinus str. MIT 9312]

MAATVKKTGSKKSKRNVPNGVVHIQSTFNNTIVSITDTSGHVISWSSAGASGFKGARKGTPFAAQTAAEA

AARRALDQGMRQIEVLVRGPGSGRETAIRALQVAGLEITLIRDVTPLPHNGCRRPKRRRV

>gi|78713510|gb|ABB50687.1| LSU ribosomal protein L17P [Prochlorococcus marinus str. MIT 9312]

MRHQLRIPLLSKPADQRKALLRGLTTQLIREGRVTTTKARAKALRNEAERMISLAKDGSLASRRRAIGYI

YDKKLVHSLFEKAQERYGDRKGGYTRIVRTVSRKGDNAQMAIIELV

>gi|78713508|gb|ABB50685.1| LSU ribosomal protein L13P [Prochlorococcus marinus str. MIT 9312]

MNKTITPSLETIERNWFLVDAKDKTLGRLATEIATVLRGKNKPTFTPHLDTGDFVIVVNAEKIEVSGKKA

SQKLYRRHSGRPGGMKTEKFESLQERIPERIIEQAVKGMLPHNSLGRQQFKKLKVYKGADHPHAAQNPVL

LNS

>gi|78713507|gb|ABB50684.1| SSU ribosomal protein S9P [Prochlorococcus marinus str. MIT 9312]

MNSQIKNKAVYWGTGRRKTSVARVRLIPGNGQIKINGRSGDDYLNFNPLHLNSIKAPLQTLGLENSYDIL

VNVFGGGLTGQADAIKQGAARALCELSPDNRKPLKTEGHLSRDPRAKERRKYGLKKARKAPQFSKR

>gi|78713506|gb|ABB50683.1| LSU ribosomal protein L31P [Prochlorococcus marinus str. MIT 9312]

MVLKKQEKLHNSPNVKEILIMPKSEIHPKWYPDAKVICNGEVVMTTGSTQPELHVDVWSGNHPFFTGTQK

ILDTEGRVDRFMKKYGMGSANSATSKEQKEEKDSNK

>gi|78713486|gb|ABB50663.1| SSU ribosomal protein S12P [Prochlorococcus marinus str. MIT 9312]

MPTISQLIGSERKRLTRKTKSPALKSCPERRGVCTRVYTSTPKKPNSALRKVARVRLTSGFEVTAYIPGI

GHNLQEHSVVLLRGGRVKDLPGVRYHIIRGTLDTAGVKDRRQSRSKYGAKAPKD

>gi|78713485|gb|ABB50662.1| SSU ribosomal protein S7P [Prochlorococcus marinus str. MIT 9312]

MSRRNAAVKRPVLPDPQFNSRLASMMISRLMKHGKKSTAQRILSDAFSLISERTGGDAVELFETAVKNAT

PLVEVRARRVGGATYQVPMEVRQERGTAMALRWLVTFSRARNGKSMSQKLAGELMDAANETGSSVKKRED

THKMAEANKAFAHYRY

>gi|78713482|gb|ABB50659.1| SSU ribosomal protein S10P [Prochlorococcus marinus str. MIT 9312]

MTASIAQQKIRIRLKAFDRRMLDLSCEKIIQTADTTSASAIGPIPLPTKRKIYCVLRSPHVDKDSREHFE

TRTHRRLIDIYSPSAKTIDALMKLDLPSGVDIEVKL

>gi|78713463|gb|ABB50640.1| SSU ribosomal protein S20P [Prochlorococcus marinus str. MIT 9312]

MANNKSAKKRIKIAERNRLINKSYKSTVRTLTKKTLENCEKYKKDPNDENKDLVKTSLNKAFSLIDKAVK

KNVLHKNNGANRKSKINNLVKTTLATQ

>gi|78713334|gb|ABB50511.1| [LSU ribosomal protein L11P]-lysine N-methyltransferase [Prochlorococcus marinus str. MIT 9312]

MASKDWYKLNFQIESDLEEIIIWKLNELGIFSFSFEYLIKTENKKEVNIWLPINEWDESSRSDFEKIICK

LLNINDSINKFFDWSVIKEEDWLTSWKKYWAPELVGNHFLILPCWINLNEEFNDKQIIKIDPGAAFGTGS

HPSTYLCLEKMEKILLSDKKVLDIGSGSGILSIAARLLGAKEVCAIDNDYLAINSTNSNFQLNFGNLNNL

NTYLGSFNEVILKHQLKKIDFVVCNILAEVIKEMIPNIYKCLRNNGEVIFSGILNSQKDEIIKILIQNNL

KLLDVSSRKDWACIYAQKAKNLT

>gi|78713326|gb|ABB50503.1| LSU ribosomal protein L27P [Prochlorococcus marinus str. MIT 9312]

MAHKKGTGSTRNGRDSNSKRLGVKAYGGEKVTAGSILIRQRGTSFLPGINVGKGKDDTLFALKEGTVSFE

SIKRNLRNRKRVNVVI

>gi|78713325|gb|ABB50502.1| LSU ribosomal protein L21P [Prochlorococcus marinus str. MIT 9312]

MTNSKNSSNNSTNGNELYAIAETSGQQFWFEVNRYYDIDRLNAKEKDKITLEKILLLKDKNSITIGKPYV

KDAKIELEVVSHKRDKKILVYKMRPKKKTRRKMGHRQELTRVMVKSISLGKSAPKSSAKKETVKKETKPK

SEKSTN

>gi|78713173|gb|ABB50350.1| SSU ribosomal protein S14P [Prochlorococcus marinus str. MIT 9312]

MAKKSMIARDVKRKKLVKKYAAKRKSLLDEFNAAKDPMERLEIHRKIQALPRNSAPNRVRNRCWATGKPR

GVYRDFGLCRNQLRQRAHNGELPGVVKSSW

>gi|78713166|gb|ABB50343.1| LSU ribosomal protein L34P [Prochlorococcus marinus str. MIT 9312]

MTKRTFGGTSRKRKRVSGFRVRMRSHTGRRVIKSRRQKGRERIAV

>gi|78712876|gb|ABB50053.1| SSU ribosomal protein S21P [Prochlorococcus marinus str. MIT 9312]

MLEYNFIKEILTQVTVGENEGIESALRRFKRQVSKSGIFADLKRLRHHETPIEKYKRKLQQRRKARRR

>gi|78712815|gb|ABB49992.1| SSU ribosomal protein S18P [Prochlorococcus marinus str. MIT 9312]

MPNSIFKKQLSPIKPGDPIDYKDVELLKKFITERGKILPRRMTGLTSKQQRDLTLAVKRARIVALLPFVN

PEG

>gi|78712814|gb|ABB49991.1| LSU ribosomal protein L33P [Prochlorococcus marinus str. MIT 9312]

MYNRKLYNKVLIKMAKKGTRVVVTLECTEARTSTDPKRSNGVSRYTTEKNRRNTTERLELKKFNPHLNRM

TIHKEIK

>gi|78712781|gb|ABB49958.1| LSU ribosomal protein L28P [Prochlorococcus marinus str. MIT 9312]

MSRVCELTGAKANNGMAVSHSHIRTKKLQQVNLQKRRLWWEEGKKWVNIKISTKALKSVQKVGLDKFAKS

NGVDLKKF

>gi|78712739|gb|ABB49916.1| SSU ribosomal protein S15P [Prochlorococcus marinus str. MIT 9312]

MSLDTAEKQKLIETHQVHATDTGSVEVQVAMLSKRISKLSEHLQGNIHDFASRQGLLKMIGKRKRLLSYI

KDKNVQKYQDLVKKIGIRG

>gi|78712644|gb|ABB49821.1| SSU ribosomal protein S2P [Prochlorococcus marinus str. MIT 9312]

MAVVSLSEMMEAGAHFGHQTRRWNPKMSKYIYCARNGVHIIDLVKTALCMNNAYKWTRNAAKGGKRFLFV

GTKKQASDVVAQEATRCGAAYVNQRWLGGMLTNWTTMKARIERLKDLERMESSGSIAMRPKKEAAVLRRE

LERLQKYLGGLKGMRRLPDVVVLVDQRRETNAVLEARKLDISLVSMLDTNCDPDLCEVPIPCNDDAVRSV

QLILGRLADAINEGRKASNNEKN

>gi|78712359|gb|ABB49536.1| LSU ribosomal protein L19P [Prochlorococcus marinus str. MIT 9312]

MAKEKQENELETIIKTDGSVDVAVEQKEKNLVSETTKTISVSNLIEEFENEQLKKELPEIYVGDTVKVGV

RITEGNKERVQPYEGVVIAKRHGGIHQTITVRRIFQGIGVERVFMLHSPQVASLKVERRGKVRRAKLFYL

RDRVGKATRVKQRFDR

>gi|78711990|gb|ABB49167.1| SSU ribosomal protein S12P methylthiotransferase [Prochlorococcus marinus str. MIT 9312]

MRVINGMPFEFFNFLKFVKQNNLNVKEKKFSKVAFSHVGCEKNLVDTQHMQGLLDKDGYEVESNINDANI

VVVNTCSFIETAREESIRKILEYTNQGKEVIVAGCMAQHFKEELLKEIPEIKGLVGTGDYQKIAKVLDRV

EKGEIVNEVSKIPEFIADEEIPRFVDKNKFVAYLRIAEGCNYNCAFCIIPKLRGPQRSRTIESIVSEAKS

LAKQGIKEIILISQITTNYGKDIYGKPSLAKLLNELSKVPIPWIRIHYAYPTGLTDEVIRAFKDSKNIVP

YFDLPLQHSHPDVLKSMNRPWQASLNESILEKIREEIPSAVLRTSLIVGFPGEKKEHFEHLLEFLDRHKF

DHVGVFIFSPEEGTAAFDLPNKVSPEVAAARKDNVISVQQNISKDKNQSYVGSKMKILVEKISDNNELIG

RSYNFAPEIDGNVILSISANNYLRNYIGKFVEANISFADEYDLYGETIKIL

>gi|78713527|gb|ABB50704.1| LSU ribosomal protein L16P [Prochlorococcus marinus str. MIT 9312]

MLSPKRTKFRKQHRGRMRGVASKGNTIAFGQFALQAQDCGWVTARQIEASRRAMTRYIKRGGQIWIRIFP

DKPVTMRPAETRMGSGKGNPEFWVAVVKPGRILFEMGGDDITEEIAKEAMRLAQYKLPVKTKFISSDKNL

EVSSQENTKNNKESQEEVKQ

>gi|78713526|gb|ABB50703.1| LSU ribosomal protein L29P [Prochlorococcus marinus str. MIT 9312]

MKNSESLKEFKKLNSAQITEKIDQLRKDLFDLRFKQATRQLNETHKFKTIKKQVAQLLTLSKSQSASQTT

SE

>gi|78712830|gb|ABB50007.1| LSU ribosomal protein L32P [Prochlorococcus marinus str. MIT 9312]

MAVPKKKKSKSKRNHRHAVWKGKAAIAAQKAISLGKSVLTGKAQGFVYPIEEEEEE

>gi|78712280|gb|ABB49457.1| SSU ribosomal protein S30P / sigma 54 modulation protein [Prochlorococcus marinus str. MIT 9312]

MKILIHGKNLELTGALKEYTQAKIEKATHHYKDIVKEADIHLSIEKNPSVSLQTAEVTIFANGTVIRAEE

KTENLYSSIDLVSNKLCRKLRKYKERNYKTNYINQFKNNESFPIESADSNVLDKTLFKQGREASLPEPSI

KNKYFEMNPISSEEARKQLDLIDHDFYVFRNKKNNELQVIYKRNHGGYGLIQSK

>gi|78713468|gb|ABB50645.1| NusA antitermination factor [Prochlorococcus marinus str. MIT 9312]

MALVILPGLNNLIEDISEEKKLPPNIVELALREALLKGYEKYRKTFYIGVVEDPFDEEYFNNFDVGLDLD

EEGYRVLSSKIIVEEVESEDHQISLAEVKQVAEDAQIGDTVVLDVTPEKEDFGRMAASTTKQVLAQKLRD

QQRKMIQEEFADLEDPVLTARVIRFERQSVIMGVSSGIGRPEVEAELPKRDQLPNDNYRANATFKVFLKE

VSEIARKGPQLFVSRANAGLVVYLFENEVPEIQEGTVKIVAVSREANPPSRGVGPRTKVAVDSVEQEVDP

VGACIGARGARIQQVVNELRGEKIDVIKWSSDPIQYILNSLSPAKVDQVRLVDPAGQHAHVLVPPDQLSL

AIGREGQNVRLAARLTGWKIDVKNSHEYDQKAEDAAVSELIIQREDEENLQREAELRLEAEQAERAAEDA

RLRELYPLPEDDQEYEEEEQYGEGDLSDNDQLETLQDSEIAAKEERKR

>gi|78711903|gb|ABB49080.1| GTPase EngC [Prochlorococcus marinus str. MIT 9312]

MKTNSKHLGLVTKKFNDFFLVDLKNKENIGKSKKFLCKVKKSINFKDQFIYVGDEVIIDNIDLRSKRALI

TSLKKRKNLLIRPSVANISNIYIIFSVKEPELNLSQVNRFLISAESMGVEVSLVLTKCDLISEKKRSFLL

DKFGKWGYQTITLNLQKPNYLKNLLVELKKKKCSIFMGPSGVGKTTLLNMIIPGLQNITAPVSNKIKRGK

NTTRNVELFPISSQSYIVDTPGFNMQPLKVDIRLLPNLYSEIYTQVIDDGIRCKFRNCLHLNDEGCNLNK

SFERYSFYKEMIESSKSQYYQNQED

>gi|78712320|gb|ABB49497.1| conserved hypothetical protein [Prochlorococcus marinus str. MIT 9312]

MILKKKEFFLLIFLILMQSCSGGRIGNFLESSFNDLEKISQDEDFQKNLEKPKSVLENKKINEKNNEKRK

KIEKSKSVLENKKINEKNNEKRKKIEKPKSVLENKKINEKNNEKRKKIEKSKSVLENKKEINSEKIQKQK

NNKIKNSSKKRKIELQSYKIIFILKDVDPKDPTEELSTILSDSEVNFEIEKIERILDSKNKIMNKN

>gi|118573633|sp|Q318J8.1|RL6_PROM9 RecName: Full=50S ribosomal protein L6

MSRIGKTPVLIPEKVTVDLDGLIVTVKGPKGELKRLMPEGVSFDKKDNTVVVSPTTNKIHSRQRHGLCRA

LIANMVEGVTQGFSKKLEIVGVGSRAQVKGKNLVVSAGYSHPVEMIPPDGITYKVESNTNVTVSGIDKEI

VGNEAAKIRSIRPPEPYKGKGIKYHDERILRKAGKSGKK

>gi|108864720|sp|Q317W9.1|RL9_PROM9 RecName: Full=50S ribosomal protein L9

MAKRVKVALTESIASLGKEGDLVEVAPGYARNFLLPYGKAMNVTPAVLKQIERKKEKEKIAADKLKQEAL

DFQTALSTIGRFTIKKQVGEDGVLFGTVTNGDVAEAIEAATKKEIDRRNITVPDIHNLGSFTAKIKLHPD

VNAEVNIEVTS

>gi|118597273|sp|Q31CX7.1|RL11_PROM9 RecName: Full=50S ribosomal protein L11

MAKKIVAVIKLALQAGKANPAPPVGPALGQHGVNIMAFCKEYNAKTQDKAGFVIPVEISVFEDRSFTFIT

KTPPAAVLITKAAGIDKGSGESSKGSVGNISKAQLEEIAKTKLPDLNCSSVESAMKVIEGTARNMGVSIT

D

>gi|91207876|sp|Q318K0.1|RS5_PROM9 RecName: Full=30S ribosomal protein S5

MTDTPTKQEITSKNDKVPGAIPGEQKKNNRNNDRKRNRRGDSKNLERDSDWQERVVQIRRVSKTVKGGKK

MSFRAIVVVGNEKGQVGVGVGKAGDVIGAVRKGVSDGKKNLVRVPLTPNNSIPTLSKGRDGAANVLIRPA

APGTGVIAGGSIRTVLELAGIKNVLAKRLGSKTPLNNARAAMVALSQLRTHKSASRERGISLEQLYS

>gi|97181829|sp|Q31CX9.1|RL10_PROM9 RecName: Full=50S ribosomal protein L10

MGRTLENKQQIVTEIKSLLNDSEMAVVLDYKGLTIKEMSDLRSRLQTTNGICKVTKNSLMRKAIDGDSNW

NDLESLLTGTNAFVLIKEDVGGAVKAIQSFQKDTKKSETKGALFEGRLLSNSEIKEIASLPSKEVLMAKI

AGALNGVATKIAISINEVPSGLARSLKQHSEKPES

>gi|109894782|sp|Q319K4.1|RS16_PROM9 RecName: Full=30S ribosomal protein S16

MIKLRLKRFGKKKEASFRIVACNSTSRRDGRPLQELGFYNPRTKETRLNTEALRIRLTQGAQPTDVVRNL

LEKGGLLEKKERPSIAIGKAKLEKEKVAKAKIKNEENDSSKAESDSNEAES

>gi|109893781|sp|Q31CY0.1|RL7_PROM9 RecName: Full=50S ribosomal protein L7/L12

MSAKTEEILESLKSLSLLEASELVKQIEEAFGVSAAASAGVVMAAPGATGGDGDGGAAEEKTEFDVVLES

FDAAAKIKVLKVVRNATGLGLGDAKALVESAPKTVKEGIAKADAESLKKEIEEAGGKVTLK

>gi|109893707|sp|Q318J6.1|RL5_PROM9 RecName: Full=50S ribosomal protein L5

MTLKNRYKESIRPKLLKDLGLKNIHQVPKVVKVNVNRGLGEAASNSKSLEASLNEMATITGQKALVTRAK

KAIAGFKIREGMPIGCTVTLRGDRMYSFLERFINLALPRIRDFRGVNPKSFDGRGNYTVGVKEQLIFPEI

SFDKIDSIRGMDITIVTSARSDQEGKALLQELGMPFSKN

>gi|109893218|sp|Q318I9.1|RL22_PROM9 RecName: Full=50S ribosomal protein L22

MTNTSETTKTAIAHGNYVRGSASKVRRVLDQIRGRSYRDALIMLEFMPYRSTDPITKVLRSAVANAEHNY

GMDPSTLIISSAWANNGPVMKRYRPRAQGRAFSIKKQTCHISISVESAPTQINAEVQN

>gi|91207367|sp|Q31CX8.1|RL1_PROM9 RecName: Full=50S ribosomal protein L1

MKKLSKRMAALSTKIEDRIYPPLEALSIIKENANAKFDETIEAHIRLGIDPKYTDQQLRTTVALPHGTGQ

SIKIAVITSGENVSKAKSAGADLFGEEDLVESINKGNMDFDLLIATPDMMPKVAKLGRVLGPRGLMPNPK

AGTVTNDIANAIKEFKAGKLEFRADKAGIVHVRFGKASFTKEALFDNLKTLQESIDKNKPSGSKGKYWKS

FYVTSTMGPSVQVDISAVQDYQPEG

>gi|91207828|sp|Q318J0.1|RS3_PROM9 RecName: Full=30S ribosomal protein S3

MGHKIHPSGLRLGITQEHRSKWFATSKTYPILLQEDFKIRTFIQKKYGAAGISDVLIARKADQLELELKT

ARPGVIVGRQGSGIEELRAGIQKTIGDNTRQVRINVVEVERVDADAFLLAEYISQQLEKRVAFRRTIRMA

LQRAQRAGVLGLKIQVGGRLNGAEIARTEWTREGRVPLHTLRAEIDYATREANTTYGVLGIKVWVFKGEV

LPKEEKTIPVGASPKRKAGRRPQQFEDRSNENS

>gi|90101704|sp|Q31CC9.1|RS4_PROM9 RecName: Full=30S ribosomal protein S4

MSRYRGPRLRVTRRLGELPGLTRKASKKSNPPGQHGQARRKRSEYAIRLEEKQKLRFNYGVSEKQLVRYV

KKARAQEGSTGTNLLRLLENRLDNVCFRLGFGGTIPGSRQLVNHGHVTVNGKVLDIAGYQCKSGDVIGIK

ENKASKKLVEGNIEFPGLANVPPHLDLDKPKLTGKITGKCDREWVALEINELLVVEYYSRKV

>gi|123553962|sp|Q319V2.1|RL34_PROM9 RecName: Full=50S ribosomal protein L34

MTKRTFGGTSRKRKRVSGFRVRMRSHTGRRVIKSRRQKGRERIAV

>gi|124078997|sp|Q317Y1.1|RL35_PROM9 RecName: Full=50S ribosomal protein L35

MSKLKTRKSAAKRFKATATGKFMRRRAYHNHLLDHKSSKLKRHLSTKAVVDERDADNVKLMIPYA

>gi|123553958|sp|Q319U5.1|RS14_PROM9 RecName: Full=30S ribosomal protein S14

MAKKSMIARDVKRKKLVKKYAAKRKSLLDEFNAAKDPMERLEIHRKIQALPRNSAPNRVRNRCWATGKPR

GVYRDFGLCRNQLRQRAHNGELPGVVKSSW

>gi|123553850|sp|Q319E2.1|RL27_PROM9 RecName: Full=50S ribosomal protein L27

MAHKKGTGSTRNGRDSNSKRLGVKAYGGEKVTAGSILIRQRGTSFLPGINVGKGKDDTLFALKEGTVSFE

SIKRNLRNRKRVNVVI

>gi|123553722|sp|Q318L0.1|RL13_PROM9 RecName: Full=50S ribosomal protein L13

MNKTITPSLETIERNWFLVDAKDKTLGRLATEIATVLRGKNKPTFTPHLDTGDFVIVVNAEKIEVSGKKA

SQKLYRRHSGRPGGMKTEKFESLQERIPERIIEQAVKGMLPHNSLGRQQFKKLKVYKGADHPHAAQNPVL

LNS

>gi|123553721|sp|Q318K8.1|RL17_PROM9 RecName: Full=50S ribosomal protein L17

MRHQLRIPLLSKPADQRKALLRGLTTQLIREGRVTTTKARAKALRNEAERMISLAKDGSLASRRRAIGYI

YDKKLVHSLFEKAQERYGDRKGGYTRIVRTVSRKGDNAQMAIIELV

>gi|123553720|sp|Q318K4.1|RL36_PROM9 RecName: Full=50S ribosomal protein L36

MKVRSSVKKISPDDQIVRRRGKIYVINKKRPRNKQRQG

>gi|123553648|sp|Q317Y0.1|RL20_PROM9 RecName: Full=50S ribosomal protein L20

MARVKRGNIARKRRNKILNLAKGFRGGNKNLFRTANQRVMKALCNAYRDRRRRKRDFRRLWISRINASAR

INGTNYSKLINGMKNSEIIINRKMLAQLALNDPKCFEKIVSSVSN

>gi|119365969|sp|Q319E3.1|RL21_PROM9 RecName: Full=50S ribosomal protein L21

MTNSKNSSNNSTNGNELYAIAETSGQQFWFEVNRYYDIDRLNAKEKDKITLEKILLLKDKNSITIGKPYV

KDAKIELEVVSHKRDKKILVYKMRPKKKTRRKMGHRQELTRVMVKSISLGKSAPKSSAKKETVKKETKPK

SEKSTN

>gi|119361703|sp|Q318J4.1|RL14_PROM9 RecName: Full=50S ribosomal protein L14

MIQQETYLTVADNSGAKRLQCIRVLGSNRRYAHVGDVIVATVKDALPNMGVKKSEVVKAVIVRTKATLRR

NTGNSIRFDDNAAVLINEDKNPKGTRVFGPVARELRDKNYTKIVSLAPEVI

>gi|118597449|sp|Q31B29.1|RS15_PROM9 RecName: Full=30S ribosomal protein S15

MSLDTAEKQKLIETHQVHATDTGSVEVQVAMLSKRISKLSEHLQGNIHDFASRQGLLKMIGKRKRLLSYI

KDKNVQKYQDLVKKIGIRG

>gi|118572993|sp|Q318L2.2|RL31_PROM9 RecName: Full=50S ribosomal protein L31

MPKSEIHPKWYPDAKVICNGEVVMTTGSTQPELHVDVWSGNHPFFTGTQKILDTEGRVDRFMKKYGMGSA

NSATSKEQKEEKDSNK

>gi|116256022|sp|Q31C59.2|RL19_PROM9 RecName: Full=50S ribosomal protein L19

MVSETTKTISVSNLIEEFENEQLKKELPEIYVGDTVKVGVRITEGNKERVQPYEGVVIAKRHGGIHQTIT

VRRIFQGIGVERVFMLHSPQVASLKVERRGKVRRAKLFYLRDRVGKATRVKQRFDR

>gi|115502684|sp|Q318K1.1|RL15_PROM9 RecName: Full=50S ribosomal protein L15

MTSTLNTLKSNSGSRKKKLRKGRGIAAGQGASCGFGMRGQKSRSGRPTRPGFEGGQMPLYRRVPKLKHFE

IINQKNFSIINLDKLKDFNDNDTVNLDSLVKKGLIFKPKFPLKILGNGKVNVKLTVQAHAFTKVAKQKIE

DAGGSCELLNNK

>gi|115305698|sp|Q318Q5.1|RS20_PROM9 RecName: Full=30S ribosomal protein S20

MANNKSAKKRIKIAERNRLINKSYKSTVRTLTKKTLENCEKYKKDPNDENKDLVKTSLNKAFSLIDKAVK

KNVLHKNNGANRKSKINNLVKTTLATQ

>gi|115305666|sp|Q318N2.1|RS12_PROM9 RecName: Full=30S ribosomal protein S12

MPTISQLIGSERKRLTRKTKSPALKSCPERRGVCTRVYTSTPKKPNSALRKVARVRLTSGFEVTAYIPGI

GHNLQEHSVVLLRGGRVKDLPGVRYHIIRGTLDTAGVKDRRQSRSKYGAKAPKD

>gi|115305643|sp|Q318N6.1|RS10_PROM9 RecName: Full=30S ribosomal protein S10

MTASIAQQKIRIRLKAFDRRMLDLSCEKIIQTADTTSASAIGPIPLPTKRKIYCVLRSPHVDKDSREHFE

TRTHRRLIDIYSPSAKTIDALMKLDLPSGVDIEVKL

>gi|109894841|sp|Q318N3.1|RS7_PROM9 RecName: Full=30S ribosomal protein S7

MSRRNAAVKRPVLPDPQFNSRLASMMISRLMKHGKKSTAQRILSDAFSLISERTGGDAVELFETAVKNAT

PLVEVRARRVGGATYQVPMEVRQERGTAMALRWLVTFSRARNGKSMSQKLAGELMDAANETGSSVKKRED

THKMAEANKAFAHYRY

>gi|109893618|sp|Q318I5.1|RL4_PROM9 RecName: Full=50S ribosomal protein L4

MTTIETLKWDGKKSGEVKLDLTVAKETSSADLIHRAVLRQLANKRQGTASTLTRSEVRGGGRKPYKQKGT

GRARQGSIRTPLRPGGGIIFGPKPRSYNLDMNRKERRLALRTALMSRISDVKAVEDFGSTLKQPKTSDII

NGLTRLGIQKTEKVLVILDSPSEVIKKSINNIEKVKLIAADQLNVFDILNANKLVIGQSAINKIKEVYAS

>gi|109893305|sp|Q318J5.1|RL24_PROM9 RecName: Full=50S ribosomal protein L24

MLDSLKQKKNFQRIKMRIKTGDLVKVINGKDKGKTGEVLKTIPLENRVVVKGINLRTKHVKPTQEGETGR

ILTEEASLHASNVMFFSKDKNLTSKIEYFIDKQGVKKRRLKKTGEVID

>gi|94730532|sp|Q318J3.1|RS17_PROM9 RecName: Full=30S ribosomal protein S17

MALKERIGTVVSDKMDKTVVVAVINRYPHPTYKKIVSRTTRYKAHDPENTCALGDRVKIRETRPLSAHKR

WAIQEILNKTSQTKEVKK

>gi|91207714|sp|Q318K5.1|RS13_PROM9 RecName: Full=30S ribosomal protein S13

MARIAGIDIPREKRVEIALTYVYGIGLTRSKLILANAGVNPDIRVKDLSDSDVQKLRGATEEFTLEGDLR

RKEGMALKRLQDIGCVRGRRHRMSLPVRGQRTRTNARTRRGSRKTVAGRKK

>gi|91207667|sp|Q318K6.1|RS11_PROM9 RecName: Full=30S ribosomal protein S11

MAATVKKTGSKKSKRNVPNGVVHIQSTFNNTIVSITDTSGHVISWSSAGASGFKGARKGTPFAAQTAAEA

AARRALDQGMRQIEVLVRGPGSGRETAIRALQVAGLEITLIRDVTPLPHNGCRRPKRRRV

>gi|123741472|sp|Q31BC4.1|RS2_PROM9 RecName: Full=30S ribosomal protein S2

MAVVSLSEMMEAGAHFGHQTRRWNPKMSKYIYCARNGVHIIDLVKTALCMNNAYKWTRNAAKGGKRFLFV

GTKKQASDVVAQEATRCGAAYVNQRWLGGMLTNWTTMKARIERLKDLERMESSGSIAMRPKKEAAVLRRE

LERLQKYLGGLKGMRRLPDVVVLVDQRRETNAVLEARKLDISLVSMLDTNCDPDLCEVPIPCNDDAVRSV

QLILGRLADAINEGRKASNNEKN

>gi|123741424|sp|Q318I6.1|RL23_PROM9 RecName: Full=50S ribosomal protein L23

MSKLFDSRLADVIRKPVITEKATNALDLNQYTFEVDHRAAKPQIKAAIEALFSVKVIGVNTMNPPRRTRR

VGKFSGKRSQVKKAIVRLAEGDKIQLFPES

>gi|123554241|sp|Q31AY7.1|RL28_PROM9 RecName: Full=50S ribosomal protein L28

MSRVCELTGAKANNGMAVSHSHIRTKKLQQVNLQKRRLWWEEGKKWVNIKISTKALKSVQKVGLDKFAKS

NGVDLKKF

>gi|123553723|sp|Q318L1.1|RS9_PROM9 RecName: Full=30S ribosomal protein S9

MNSQIKNKAVYWGTGRRKTSVARVRLIPGNGQIKINGRSGDDYLNFNPLHLNSIKAPLQTLGLENSYDIL

VNVFGGGLTGQADAIKQGAARALCELSPDNRKPLKTEGHLSRDPRAKERRKYGLKKARKAPQFSKR

>gi|119367288|sp|Q31AP2.2|RS21_PROM9 RecName: Full=30S ribosomal protein S21

MTQVTVGENEGIESALRRFKRQVSKSGIFADLKRLRHHETPIEKYKRKLQQRRKARRR

>gi|119367139|sp|Q318I8.1|RS19_PROM9 RecName: Full=30S ribosomal protein S19

MGRSLKKGPFIADSLLKKVEKQNTDNDKSVIKTWSRASTILPLMIGHTIAVHNGKTHIPVFITEQMIGHK

LGEFAPTRTYRGHIRDKKGAKS

>gi|115504928|sp|Q318J9.1|RL18_PROM9 RecName: Full=50S ribosomal protein L18

MTKLSRKLQTQKRHRRLRRSVIGDATRPRLSVFRSNNHIYAQVIDDSAQKTICSASTVDKELREKSDKLP

SDCNSSSIVGKLLANRAIKKGIKQVIFDRGGNLYHGRVKALADAAREAGLEF

>gi|109893536|sp|Q318I4.1|RL3_PROM9 RecName: Full=50S ribosomal protein L3

MSIGILGKKLGMSQLFDDKGNAVPVTLIEAGPCRVTQLKTTTLDGYSAVQIGYGLSKDKHINKPEKGHLL

KSGEELLKHLKEYRVEETTSYEIGNQITVKNFEVGQKVDISGKSMGRGFAGYQKRHGFSRGPMSHGSKNH

RAPGSTGAGTTPGRIYPGKRMAGRYGGKQITTKGLLVLKIDDQKNLLVIKGSVPGKPGSIVNIKPNNVVG

KKGGQKS

>gi|108862042|sp|Q318I7.1|RL2_PROM9 RecName: Full=50S ribosomal protein L2

MAIRKFKPYTPGTRQRVVTDFSEITSAKPERSLIVSKHRVKGRNNRGVITCRHRGGGHKRQYRLVDFRRD

KRNINAKVAAIHYDPHRNARLALLFYEDGEKRYIIAPAGVKVGQNVISGESVPIEDGNAMPLSVMPLGSS

VHCVELYAGRGAQMVRSAGASAQVMAKEGDYVALKLPSTEVRLVRKECYATLGEVGNSEIRNTSLGKAGR

RRWLGRRPQVRGSVMNPCDHPHGGGEGKAPIGRAGPVTPWGKPALGLKTRKKNKPSNKLVVRRRRRISKR

SRGGRDS

>gi|91207929|sp|Q317T5.1|RS6_PROM9 RecName: Full=30S ribosomal protein S6

MTNQSYYETMYILRPDIAEDEVTNHIDKYNKLLEEFGGTILDSQMRGKRRLAYQIAKHREGIYVQLSHQG

DGQHIFKIEKAMRISEDVIRYMTVKQEGPLPTPKPSNKSSTQSENKDNPETKVESKEEQSVTNSDTSTTK

KDDNEIKENTES

>gi|90101742|sp|Q318J7.1|RS8_PROM9 RecName: Full=30S ribosomal protein S8

MSNHDPISDMLTRIRNASQKKHTTTTIPGSKMSLSIAKVLQKEGFISDINEEGEGYKSQIILSLKYSGKN

KFPTIRSMQRVSKPGLRIYKNTRGLPKVLGGLGVAIISTSKGVMSDRDARKQGIGGEVLCYVY

>gi|218551743|sp|Q31AV4.2|RL33_PROM9 RecName: Full=50S ribosomal protein L33

MAKKGTRVVVTLECTEARTSTDPKRSNGVSRYTTEKNRRNTTERLELKKFNPHLNRMTIHKEIK

>gi|123741464|sp|Q31AV3.1|RS18_PROM9 RecName: Full=30S ribosomal protein S18

MPNSIFKKQLSPIKPGDPIDYKDVELLKKFITERGKILPRRMTGLTSKQQRDLTLAVKRARIVALLPFVN

PEG

>gi|123554208|sp|Q31AT8.1|RL32_PROM9 RecName: Full=50S ribosomal protein L32

MAVPKKKKSKSKRNHRHAVWKGKAAIAAQKAISLGKSVLTGKAQGFVYPIEEEEEE

>gi|123553719|sp|Q318J2.1|RL29_PROM9 RecName: Full=50S ribosomal protein L29

MKNSESLKEFKKLNSAQITEKIDQLRKDLFDLRFKQATRQLNETHKFKTIKKQVAQLLTLSKSQSASQTT

SE

>gi|115502775|sp|Q318J1.1|RL16_PROM9 RecName: Full=50S ribosomal protein L16

MLSPKRTKFRKQHRGRMRGVASKGNTIAFGQFALQAQDCGWVTARQIEASRRAMTRYIKRGGQIWIRIFP

DKPVTMRPAETRMGSGKGNPEFWVAVVKPGRILFEMGGDDITEEIAKEAMRLAQYKLPVKTKFISSDKNL

EVSSQENTKNNKESQEEVKQ

>gi|238066629|sp|Q31D78.2|RIMO_PROM9 RecName: Full=Ribosomal protein S12 methylthiotransferase RimO; Short=S12 MTTase; Short=S12 methylthiotransferase; AltName: Full=Ribosomal protein S12 (aspartate-C(3))-methylthiotransferase; AltName: Full=Ribosome maturation factor RimO

MKQNNLNVKEKKFSKVAFSHVGCEKNLVDTQHMQGLLDKDGYEVESNINDANIVVVNTCSFIETAREESI

RKILEYTNQGKEVIVAGCMAQHFKEELLKEIPEIKGLVGTGDYQKIAKVLDRVEKGEIVNEVSKIPEFIA

DEEIPRFVDKNKFVAYLRIAEGCNYNCAFCIIPKLRGPQRSRTIESIVSEAKSLAKQGIKEIILISQITT

NYGKDIYGKPSLAKLLNELSKVPIPWIRIHYAYPTGLTDEVIRAFKDSKNIVPYFDLPLQHSHPDVLKSM

NRPWQASLNESILEKIREEIPSAVLRTSLIVGFPGEKKEHFEHLLEFLDRHKFDHVGVFIFSPEEGTAAF

DLPNKVSPEVAAARKDNVISVQQNISKDKNQSYVGSKMKILVEKISDNNELIGRSYNFAPEIDGNVILSI

SANNYLRNYIGKFVEANISFADEYDLYGETIKIL

>gi|123553845|sp|Q319D4.1|PRMA_PROM9 RecName: Full=Ribosomal protein L11 methyltransferase; Short=L11 Mtase

MASKDWYKLNFQIESDLEEIIIWKLNELGIFSFSFEYLIKTENKKEVNIWLPINEWDESSRSDFEKIICK

LLNINDSINKFFDWSVIKEEDWLTSWKKYWAPELVGNHFLILPCWINLNEEFNDKQIIKIDPGAAFGTGS

HPSTYLCLEKMEKILLSDKKVLDIGSGSGILSIAARLLGAKEVCAIDNDYLAINSTNSNFQLNFGNLNNL

NTYLGSFNEVILKHQLKKIDFVVCNILAEVIKEMIPNIYKCLRNNGEVIFSGILNSQKDEIIKILIQNNL

KLLDVSSRKDWACIYAQKAKNLT

>gi|123553678|sp|Q318D6.1|RIMM_PROM9 RecName: Full=Ribosome maturation factor RimM

MINNNKWLVVGLITSCHGINGQLKVKSLSDFEERFLKPGIRWLQKENEPPSKIELTSGFKQPGKETFIIK

LQGINNRNQAERLKKCKILVKTNKLPKLKKEEFHLLELINLEVKTLENEELKIIGKVINLENEKNNLLVV

ELFKNKKKVLIPFVKEIVPLVDIKNNFLIINPPNGLLDL

>gi|33635519|emb|CAE21843.1| 30S ribosomal protein S1, homolog A [Prochlorococcus marinus str. MIT 9313]

MSATPTEQVQDSAADSTSSEQVINTTSETAEAAKQALAEEDLSIPEDVPTADDPSSRAAKNDLSGAGFTL

DEFASLLSKYDYNFKPGDIVNGTVFALESKGAMIDIGAKTAAFMPLQEVSINRVEGLSDVLLPGEIREFF

IMSEENEDGQLSLSIRRIEYQRAWERVRQLQKEDATIYSEVFATNRGGALVRVEGLRGFIPGSHISTRKP

KEELVADFLPLKFLEVDEERNRLVLSHRRALVERKMNRLEVGEVVIGAVRGIKPYGAFIDIGGVSGLLHI

SEISHEHIETPHSVLNVNDQMKVMIIDLDAERGRISLSTKALEPEPGDMLTDPQKVFDKAEEMAARYKQM

LLEQAEEGEDPEVVPLD

>gi|33640950|emb|CAE21408.1| 30S ribosomal protein S1 homolog B, putative Nbp1 [Prochlorococcus marinus str. MIT 9313]

MAGSGSPQPNRPKPPKPAAEAPRKPLQVMHISKRGEQDKLVREAAEITSPGSKATAASGQLSNAPNRSVS

ADAASDESRFDLGELQNMTMADLLGPADQSRRSASAPKGIDHRNEEGQSNPARSVDDFDFDEDAFLAALD

ENEPIGTTGEVATGKVIALESDGVYVDIGGKAPGFMPKNECGLGVITNLKERFPKGLEVEVLVTREQNAD

GMVTISCRALELRKSWSKVQQMEKEGKVAQVKVNGFNRGGVTCDLEGLRGFIPRSQLQNGENHEALIGKT

LGVAFLEVNPETRKLVLSEKRAATAARFSELEVGQLVEGQVVAVKPYGFFIDLGGVSGLLHQSMITGGSL

RSLREVFNQGDRVKALITEMDPGRGRIALNTALLEGQPGELLIEKDKVMAEATDRANKARNVLRQQEQSA

G

>gi|33641219|emb|CAE22349.1| 50S ribosomal protein L9 [Prochlorococcus marinus str. MIT 9313]

MAKRVQVVLNEDVLSLGKDGDLVEVAPGYARNFLLPFGKAVPVTPAVMKQVGHRRAKQAEHQAAIKQEAL

DFQTALVTIGRFTVKKQTGEDDVLFGTVTNGDVAEAIETATKKEIDRRNIIVPEIHRTGSYKVQVKLHNE

VNAEINLEVVSY

>gi|33641134|emb|CAE22264.1| 50S ribosomal protein L10 [Prochlorococcus marinus str. MIT 9313]

MGRTLESKQQIVEELKGLLGEAEMALVLDYQGLSIKEMSDLRTRLQASNGVCKVTKNTLMRHAINGNGAW

SNLESLLTGTNAFVLIKGDVGGAVKAVQAFQKDTKKSETKGGLFEGKLLSQGEIKAIGDLPTKEVLMAQI

AGSLNALATKVAVGINEVPSGLARALHQHAESGES

>gi|33641132|emb|CAE22262.1| 50S ribosomal protein L11 [Prochlorococcus marinus str. MIT 9313]

MAKKVVSVIKLALQAGKANPAPPVGPALGQHGVNIMAFCKEYNARTQDKAGLVIPVEISVFEDRSFTFIT

KTPPASVLITKAAGIEKGSGESAHGKVGSLSRSQLEEIAKTKLPDLNCTSIESAMRIIEGTARNMGVSIS

D

>gi|33635599|emb|CAE21923.1| 30S ribosomal protein S5 [Prochlorococcus marinus str. MIT 9313]

MTQPNTQTTPNDVPAAAEGQQEQQQQQRRGGGRERRGGGRRGDRRGQERDSEWQERVVQIRRVSKTVKGG

KKMSFRAIVVVGNERGQVGVGVGKAGDVIGAVRKGVADGKKHLVKVPLTRHNSIPTLSNGRDGAANVLIR

PAAPGTGVIAGGSIRTVLELAGIKNVLAKRLGSKTPLNNARAAMVALASLRTHKETAKERGISLEQIYS

>gi|33635597|emb|CAE21921.1| 50S ribosomal protein L6 [Prochlorococcus marinus str. MIT 9313]

MSRIGKNPIPIPDKVAVTLDGLAVSVKGPKGELSRTLPEGVSVSQVENTIVVTPTSQKRKSRERHGLCRS

LVANMVEGVSKGYTRKLELIGVGSRAQVKGKKLVVSAGFSHPVEMDPPEGVTFAVENNTNVTVSGADKEL

VGNEAAKIRAIRPPEPYKGKGIRYEGERILRKAGKSGKK

>gi|33641135|emb|CAE22265.1| 50S ribosomal protein L7/L12 [Prochlorococcus marinus str. MIT 9313]

MSKKTDDILDSLKTLSLLEASELVKQIEDAFGVSAAASAGVVVAAGGAAGGGAAAEAAEEQTEFDVVLES

FDASAKIKVLKAVREATGLGLGDAKAMVEAAPKTIKEGIAKNDAEALKKAIEEVGGKVSLK

>gi|33641133|emb|CAE22263.1| 50S ribosomal protein L1 [Prochlorococcus marinus str. MIT 9313]

MPKLSKRITGLLAKVEDRVYQPLEAIQLVKENATAKFDETIEAHVRLGIDPKYTDQQLRTTVALPQGTGQ

SVRIAVISRGEKLAEAKTAGAELAGDDDLVESIGKGQMDFDLLIATPDMMPKVAKLGRVLGPRGLMPNPK

AGTVTTDLAAAIKEFKAGKLEFRADRAGIVHVRFGKASFSADALLENLKTLQETIDRNKPSGAKGRYWKS

LYITSTMGPSVEVDVTALQDLEEDA

>gi|33640613|emb|CAE20402.1| 30S ribosomal protein S4 [Prochlorococcus marinus str. MIT 9313]

MSRYRGPRLRITRRLGDLPGLTRKAAKRSHPPGQHGQARRKRSEYAIRLEEKQKLRFNYGISERQLVRYV

KKARAQDGSTGTNLLKLLENRLDNVCFRLGFGPTVPGARQLVNHGHVTVNGRVLDIASYQCKAGDVVAIR

ERKGSKKLAEANLEFPGLANVPPHIELDKAKMSAKIISKCEREWVALEINELLVVEYYSRKV

>gi|33635595|emb|CAE21919.1| 50S ribosomal protein L5 [Prochlorococcus marinus str. MIT 9313]

MSLKQRYRETIQPKLLKDLSLSNIHEVPKVLKITVNRGLGEAAQNAKSLEASITELATITGQKVVVTRAK

KAIAGFKIRQGMPIGCAVTLRGERMYAFLERLINLALPRIRDFRGVSPKSFDGRGNYTLGVREQLIFPEI

SFDKIDAIRGMDITIVTSARTDEEGQSLLREMGMPFRSN

>gi|33635589|emb|CAE21913.1| 30S ribosomal protein S3 [Prochlorococcus marinus str. MIT 9313]

MGHKIHPTGLRLGITQEHRSRWYATSKMYPILLQEDDRIRRFIHKKYGAAGISDVLIARKADQLEVELKT

ARPGVLVGRQGSGIEELRTGIQKTIGDHSRQVRINVVEVERVDADAFLLAEYIAQQLEKRVAFRRTIRMA

VQRAQRAGVLGLKIQVGGRLNGAEIARTEWTREGRVPLHTLRAEIDYATKVASTTYGVLGIKVWIFKGEV

LGDEAQTMPVGASPRRRGNRRPQQFEDRSNEG

>gi|33635588|emb|CAE21912.1| 50S ribosomal protein L22 [Prochlorococcus marinus str. MIT 9313]

MTTSSPTTTTIAKAHGRFIRGSVSKVRRVLDQIRGRTYRDALIMLEFMPYRSTGPITKVLRSAVANAEHN

LGLDPASLVIAQASADMGPSMKRYRPRAQGRAFAIKKQTCHISIAVAAQTDS

>gi|33641303|emb|CAE22433.1| 30S ribosomal protein S6 [Prochlorococcus marinus str. MIT 9313]

MSQQPYYETMYILRPDIPEEEVETHVTKYREMVTEAGAEVLDNQMRGKRRLAYPISNHKEGIYVQLSHNG

NGQQVAVLEKAMRLSEDVIRYLTVKQEGPLPAPRIVPGSEPEPVQQQEAAAVEA

>gi|33641023|emb|CAE21481.1| Ribosomal protein L19 [Prochlorococcus marinus str. MIT 9313]

MTADSKDTSMSEDNTETATAIENSSAMVTDVTSKSAPNVRLSPDALIKEFEASQQKSDLNDIYVGDTVRV

GVRISEGNKERIQPYEGVVIAKRHGGIHETITVRRIFQGIGVERVFMLHSPQVASIKVERRGKVRRAKLF

YLRERVGKATRVKQRFDR

>gi|33640742|emb|CAE21197.1| 30S Ribosomal protein S21 [Prochlorococcus marinus str. MIT 9313]

MAQVTVGENEGVESALRRFKRAVSKAGIFSDLKRIRHHETPVEKYKRKAQQRRRSRRR

>gi|33640442|emb|CAE20231.1| 50S ribosomal protein L35 [Prochlorococcus marinus str. MIT 9313]

MPKLKTRKAAAKRFKATVTGKFMRRRAFRNHLLDHKSPKLKRHLATKAVVDERDAENVRLMLPYA

>gi|33640441|emb|CAE20230.1| 50S ribosomal protein L20 [Prochlorococcus marinus str. MIT 9313]

MARVKRGNVARKRRNKILRLARGFQGSNGSLFRTANQRVMKALCNAYRDRRRRKRDFRRLWIARINAAAR

INGVSYSRLIGGLKKADVRINRKMLAQLAVMDPKSFTSVVTSAKS

>gi|33635634|emb|CAE21958.1| 30S ribosomal protein S10 [Prochlorococcus marinus str. MIT 9313]

MSTAIAQQKIRIRLKAFDRRMLDLSCDKIIETADNTAATAIGPIPLPTKRKIYCVLCSPHVDKDSREHFE

TRTHRRIIDIYNPSAKTIDALMKLDLPSGVDIEVKL

>gi|33635631|emb|CAE21955.1| 30S ribosomal protein S7 [Prochlorococcus marinus str. MIT 9313]

MSRRNAAEKRPVLPDPQFNNRLATMMISRLMKHGKKSTAQRILAQAFGLINERTGGDPIELFETAVKNAT

PLVEVRARRVGGATYQVPMEVRQERGTAMALRWLVNFSRARNGRSMSQKLAAELMDAANEAGSAVRKREE

THKMAEANKAFAHYRY

>gi|33635630|emb|CAE21954.1| 30S ribosomal protein S12 [Prochlorococcus marinus str. MIT 9313]

MPTIQQLIRTERQHLTRKTKSPALRACPERRGVCTRVYTSTPKKPNSALRKVARVRLTSGFEVTAYIPGI

GHNLQEHSVVLIRGGRVKDLPGVRYHIIRGTLDTAGVKDRSQSRSKYGAKASKQD

>gi|33635611|emb|CAE21935.1| 50S ribosomal protein L31 [Prochlorococcus marinus str. MIT 9313]

MPKPDIHPNWYPDAKVICNGEVVMTTGSTQPELHVDVWSGNHPFFTGTQKILDTEGRVDRFMRKYGMGSA

DAAADEKKPDAKNNNKDNTSKED

>gi|33635610|emb|CAE21934.1| 30S ribosomal protein S9 [Prochlorococcus marinus str. MIT 9313]

MSSSNNSVVYWGTGRRKTSVARVRLVPGSGTITINGRPGDHYLNFNPAYLAAVKAPLQTLGLNEQYDVLV

NVHGGGLTGQADAIKQGAARALCELSADNRKPLKTEGHLSRDPRAKERRKYGLKKARKAPQFSKR

>gi|33635609|emb|CAE21933.1| 50S ribosomal protein L13 [Prochlorococcus marinus str. MIT 9313]

MNKTSVPSIDSIERQWFLVDAENQTLGRLATEVASVLRGKNKPSFTPHLDTGDFVVVVNADKIRVSGNKA

NQKLYRRHSGRPGGMKVETFQALQDRLPERIVEKAIKGMLPHNALGRQLFRKLKVYRGPEHPHSAQRPQT

LQLNPAASSQ

>gi|33635607|emb|CAE21931.1| 50S ribosomal protein L17 [Prochlorococcus marinus str. MIT 9313]

MRHQCRVPKLGRPTDQRKAMLRGLTTQLIREGRVTTTKARAKALRDEAERMITLAKNGSLASRRRAIGYI

YDKQLVHALFDKAQDRYGDRQGGYTRIIRTVPRRGDNAEMAIIELV

>gi|33635605|emb|CAE21929.1| 30S ribosomal protein S11 [Prochlorococcus marinus str. MIT 9313]

MAKPTKKTGSKKTKRNVPNGVAHIQSTFNNTIVSIADTAGEVIAWSSAGASGFKGARKGTPFAAQTAAEA

AARRALEQGMRQIEVLVRGPGSGRETAIRALQVAGLEITLIRDVTPLPHNGCRRPKRRRV

>gi|33635604|emb|CAE21928.1| 30S ribosomal protein S13 [Prochlorococcus marinus str. MIT 9313]

MARIAGVDIPRDKRVEVALTYIYGIGLTRAKTILTKSDVNPDIRVKDLEDGDVQKLRTALEAFTIEGDLR

RQEGMALKRLQDIGCLRGRRHRMSLPVRGQRTRTNARTRRGARKTVAGKKK

>gi|33635603|emb|CAE21927.1| 50S Ribosomal protein L36 [Prochlorococcus marinus str. MIT 9313]

MKVRASVKKMCDKCRVIRRHGRVMVICSTPKHKQRQG

>gi|33635600|emb|CAE21924.1| 50S ribosomal protein L15 [Prochlorococcus marinus str. MIT 9313]

MTTLRLDSLKANVGARRRKMRKGRGIAAGQGASCGFGMRGQKSRSGRPTRPGFEGGQMPLYRRVPKLKHF

TTVNSKEFTVVNVAALNELKAGSTINLDTLVKNGVVTSPKYPLKVLGNGELKVKLTIQAAAFTATARSKI

EAAGGTCEILD

>gi|33635598|emb|CAE21922.1| 50S ribosomal protein L18 [Prochlorococcus marinus str. MIT 9313]

MSNLSRKQQTQKRHRRLRRHLKGTAQRPRLAVFRSNNHIYAQVIDDEAQNTLCAASTLDKDLRTSLKADG

SSCDASNAVGDLVAKRALAKGIQQVVFDRGGNLYHGRVKSLADAAREAGLQF

>gi|33635596|emb|CAE21920.1| 30S ribosomal protein S8 [Prochlorococcus marinus str. MIT 9313]

MANHDPISDMLTRIRNASEKRHQTTRVPASRMSRSIAKVLQQEGFISEISEEGEGVRTHLVLELKYSGKH

RHPTIRSMQRVSKPGLRIYKNTRALPKVLGGLGMAIISTSKGVMSDRDARKQGVGGEVLCYVY

>gi|33635594|emb|CAE21918.1| 50S ribosomal protein L24 [Prochlorococcus marinus str. MIT 9313]

MPIATPKQKTTQRIKMRIHKGDTVQVITGKDKGKTGEVLRTLPIENRVIVQGVNIRTRHVKPTQEGESGR

IVTEEASVHASNVMLYSNKKKIASRVELVVEKDGSKKRRLKKTGELID

>gi|33635593|emb|CAE21917.1| 50S Ribosomal protein L14 [Prochlorococcus marinus str. MIT 9313]

MIQQESFLTVADNSGAKRIQCIRVLGSNRRYAHVGDVIVAAVKDAMPNMSVKKSEVVKAVVVRTKATLRR

DTGNSIRFDDNAAVLINEDKNPRGTRVFGPVARELRERNFTKIVSLAPEVI

>gi|33635592|emb|CAE21916.1| 30S Ribosomal protein S17 [Prochlorococcus marinus str. MIT 9313]

MALKERLGTVVSDKMDKTVVVAVENRFPHPIYQKTVSRTTRYKAHDAGNTCRVGDRVRITETRPLSRSKR

WTVAEVLSHSPKAQEVST

>gi|33635587|emb|CAE21911.1| 30S Ribosomal protein S19 [Prochlorococcus marinus str. MIT 9313]

MGRSLKKGPFIADSLLRKLEKQNADDDKSVIKTWSRASTILPMMIGHTIAVHNGRSHVPVFITEQMVGHK

LGEFAPTRTFKGHIKDKKGGR

>gi|33635586|emb|CAE21910.1| 50S ribosomal protein L2 [Prochlorococcus marinus str. MIT 9313]

MAIRTFRPYTPGTRTRVVTDFNEVTGRKPERSLVVAKHRRKGRNNRGVITCRHRGGGHKRLYRIVDFRRN

KHGIPAKVAAIHYDPHRKAHLALLFYTDGEKRYILAPAGIAIGQQLISGPESPIETGNALPLSAIPLGSS

VHNVELYAGRGGQMARTAGSSAQVMAKEGDYVALKLPSTEVRLVRHECYATLGEVGNSEVRNTSLGKAGR

RRWLGRRPQVRGSVMNPCDHPHGGGEGRAPIGRSGPVTPWGKPALGLKTRKRNKPSNRFVLRKRRRTSKR

SRGGRDS

>gi|33635585|emb|CAE21909.1| 50S ribosomal protein L23 [Prochlorococcus marinus str. MIT 9313]

MTERFNDRLADVIRRPLITEKATSALEQNQYTFEVDHRAAKPDIKAAVEQLFDVRVVGISTMNPPRRSRR

VGRFTGKRAQVKKAIVRLAEGNTIQLFPES

>gi|33635584|emb|CAE21908.1| 50S ribosomal protein L4 [Prochlorococcus marinus str. MIT 9313]

MAECVVHDWQGKEAGKASLELKVSKETTAVDLMHRAVLRQQAHSRQGTASTLTRAEVRGGGRKPYKQKGT

GRARQGTIRTPLRPGGGIIFGPKPRTYNLAMNRKERRLALRTALMARIEDVIVVKDFGNSLKVPKTREIS

DALIRLGLAADAKVLIILSTPSEIIRRSVRNLEKVKLIAADQLNVFDLLHANSLVLSEEALAKIQEVYGD

D

>gi|33635583|emb|CAE21907.1| 50S ribosomal protein L3 [Prochlorococcus marinus str. MIT 9313]

MSIGILGKKLGMSQFFDDQGRAIPVTLIEAGPCRITQLKTSEIDGYAAVQIGFGDTREKLINKPSKGHLT

KSGEVLLKHLREYRVEGLEGLELGAAITVGSFEAGQKVDVSGDTMGRGFAGYQKRHGFSRGPMSHGSKNH

REPGSTGAGTTPGRIYPGKRMAGRYGGKKRTTRGLTILKVDSNRNLLVVKGSVPGKPGALLNIRPAKRVG

NKPAQGGK

>gi|33635359|emb|CAE21684.1| 30S Ribosomal protein S20 [Prochlorococcus marinus str. MIT 9313]

MANNKSSKKRVQIAERNRLENKSYKSAMRTLMKRCFSACSHYSQQPGETAKANVKASIDSAFSKIDKAVK

RGVLHRNTAAHQKSRLSAAVKQAIEPAPST

>gi|33635280|emb|CAE21605.1| putative methyltransferase for Ribosomal protein L11 [Prochlorococcus marinus str. MIT 9313]

MNFAAALRWWRLSLPIADELEESLIWKLTDLGLCRLAVQHAPENSERTLLAWLPSSEWSESDRDQLMANL

RPLAEPFGLKLANPTWCEVADEDWSLNWKQDWQPDPVGQRLLILPAWLDLPQEYADRFVVRLDPGSAFGT

GSHPSTRLCLEALERNPPLGLRVADLGCGSGVLGFAALAFGARQVLAADTDCQAVCASRANTELNQLDLD

RLRVVHGSVEALSAQLEGQTVDLLLCNILAPVIEVLAPSFDQLLSANGRGLLSGLLVKQAPRLQMVLEAL

GWRVNCLTEQGCWGLLDVSKR

>gi|33635271|emb|CAE21596.1| 50S ribosomal protein L27 [Prochlorococcus marinus str. MIT 9313]

MAHKKGTGSTRNGRDSNSKRLGVKAYGGETVTAGSILIRQRGTSVLPGVNVGQGKDDTLFALTDGVVAFE

SIRRSLRNRKRISVVASS

>gi|33635270|emb|CAE21595.1| 50S ribosomal protein L21 [Prochlorococcus marinus str. MIT 9313]

MAEKPTAKPKAAEAKDQSDSYAIVEASGQQFLLQPNRYYDLDRLQAAVDDTVTLEKVLLIKDGKNDATVG

QPYVKGASVELKVMDHRRGPKIIVYKMRPKKKTRRKNGHRQELTRVMVQSISIDGKALS

>gi|33635226|emb|CAE21551.1| 30S Ribosomal protein S14 [Prochlorococcus marinus str. MIT 9313]

MAKKSMIARDVKRKKIVERYAAKRAALMEAFNAAKDPMQRLEIHRKIQALPRNSAPNRIRNRCWATGKPR

GVYRDFGLCRNQLRERAHKGELPGVVKSSW

>gi|33635218|emb|CAE21543.1| 50S ribosomal protein L34 [Prochlorococcus marinus str. MIT 9313]

MTKRTFGGTSRKRKRVSGFRVRMRNHTGRRVIRSRRKRGRARLAV

>gi|33634929|emb|CAE20915.1| 30S Ribosomal protein S18 [Prochlorococcus marinus str. MIT 9313]

MSSSFFKKRLSPIKPGDPIDYKDVDLLKKFITDRGKILPRRLTGLTSKQQRDLTNAVKRARIIALLPFVN

PEG

>gi|33634928|emb|CAE20914.1| 50S ribosomal protein L33 [Prochlorococcus marinus str. MIT 9313]

MAKNKGVRIVITLECNECRSNPAKRSPGVSRYTTEKNRRNTTERLEIKKFCPHCNKSTPHKEIK

>gi|33634884|emb|CAE20870.1| 50S ribosomal protein L28 [Prochlorococcus marinus str. MIT 9313]

MSRVCQLTGTRANNGMAVSHSHIRTKKLQQANLQQRRLWWAEGNRWLKLRVSTRALKTIQKKGLGVYAKS

LGIDLNKI

>gi|33634835|emb|CAE20820.1| 30S Ribosomal protein S15 [Prochlorococcus marinus str. MIT 9313]

MSLDTTEKQQLINANQTHGTDTGSVEVQVAMLSERITKLSSHLQENKHDFSSRQGLLKMIGRRKRLLSYV

RGKSEQRYNGLITKLGIRG

>gi|33634773|emb|CAE20759.1| 30S ribosomal protein S2 [Prochlorococcus marinus str. MIT 9313]

MAVVTLSEMMEAGAHFGHQTRRWNPKMSRYIYCARNGVHIIDLVQTAICMNNAYKWTRSSARSGKRFLFV

GTKKQASEVVALEATRCGASYVNQRWLGGMLTNWTTMKARIDRLKDLERMESSGAIAMRPKKEASVLRRE

LERLQKYLGGLKGMRRLPDVVVLVDQRRETNAVLEARKLDIPLVSMLDTNCDPDLCEVPIPCNDDAVRSV

QLVLGRLADAINEGRHGTNDQRGADDSDD

>gi|33634539|emb|CAE20525.1| 30S Ribosomal protein S16 [Prochlorococcus marinus str. MIT 9313]

MIKLRLKRFGKKREASFRLVACNSTSRRDGRPLQELGFYNPRTKETRLDTEALRLRLSQGAQPTDAVRSL

LEKGGLIEKTVRPAEVVGKAKQAEARKAAAKNVAKQAAEAKAEETPADNTEA

>gi|33640695|emb|CAE21150.1| possible Ribosomal protein L36 [Prochlorococcus marinus str. MIT 9313]

MRRKPISDFIFKSALAGILLCTSTISAEAKDKVIEGTTYSYNCREGLWKSKIIGRGVNDKEWITIERLEK

LSGTRQVNVRNDIKKSCQSSSLISKAKRAFGADCELASDSFDPSNWQIDYASRFNAHGNKYHIFAIRYGD

GSRRLCLSKNNFRDNLPIETPGRSLFLHNIERQADSSIIEYEYHPGNGWGYEVKKYRLDLSDPERPSFSV

VDSWIHQR

>gi|33635591|emb|CAE21915.1| 50S ribosomal protein L29 [Prochlorococcus marinus str. MIT 9313]

MAHPKAAEVRKLTDADITEQIDGIRRELFDLRFQQATRQLSNTHRFKEARIKLAQLLTVQKERSRSAASS

>gi|33635590|emb|CAE21914.1| 50S ribosomal protein L16 [Prochlorococcus marinus str. MIT 9313]

MLSPKRVKFRKQQRGRMRGVATRGNTIAFGEFALQAQECGWITSRQIEASRRAMTRYVKRGGKIWIRIFP

DKPVTMRPAETRMGSGKGNPEFWVAVIKPGRILFEMGGAEITEDIAKEAMRLAQYKLPIKTKFIGLDDQE

KVAGSDKPASVPAITAES

>gi|33635509|emb|CAE21833.1| possible Ribosomal protein S14p/S29e [Prochlorococcus marinus str. MIT 9313]

MKLTKLSQSEFDRGCELNNVLKECWKAFYSLTAQMNNVETDSTKYLQLDRLRQTCEKNIDEFKQELDELN

WLIRIGIRD

>gi|33635293|emb|CAE21618.1| possible Ribosomal protein L36 [Prochlorococcus marinus str. MIT 9313]

MTMDRLQRLVLSFYREDPCIEVELEPLLDCRMTRSWGSIRIECVDAEHLEEVSALLSHLRLPLAALGLGR

QIVLRVPGSLQRSYPMHVPFHSDLLA

>gi|33634946|emb|CAE20932.1| 50S ribosomal protein L32 [Prochlorococcus marinus str. MIT 9313]

MAVPKKKTSKGKRNQRHAIWKAKAATAAQRALSIGKSVLSGRAQGFVYPMQESDDDES

>gi|33634738|emb|CAE20724.1| possible Ribosomal protein L11 [Prochlorococcus marinus str. MIT 9313]

MELSIKQSGVLHGMLTGTAISIGIIIFGILLNPFNFHSNLTLLEKSSVLFKSLIILALCLTISIGRLAKH

RFFSPDELDGKGLRTDSDRAILLQSLLQNTLEQSVLAAFVYGTWTFVMPSAWLSVVPLAALSFALGRVLF

FAGYRRGAVGRALGFTMAFYPSVLMLICTVCFIPLSAVTGGC

>gi|311812490|emb|CBX83583.1| unnamed protein product [Prochlorococcus marinus str. MIT 9313]

MQGQTQSISFDGREIRLTTGRYAPQAGGSVMMECGDTAVLVTATRSTGREGIDFLPLICDYEERLYAAGR

IPGSFMRREGRPPERATLIARLIDRPMRPLFPSWMRDDLQIVATCLSLDERVPADVLAVTGASMATLLAS

IPFQGPMAAVRVGLLGDDFVLNPSYREIERGDLDLVVAGTPDGVVMVEAGANQLPQGDVIEAIDFGYEAV

CELIKAQQTILKDAGIKQVQPEPPTQDQDTKLSTYLEKNCSKSIGEVLKQFEQTKAERDSKLDAIKAKTA

EAIDSLKEDDAVRKSVNANSKVLSNNFKALTKKLMREQIIKQGKRVDGRKLDEVRPISSAAGVLPKRVHG

SGLFQRGLTQVLSTATLGTPSDAQEMDDLNPGPEKTYLHHYNFPPYSVGETRPMRSPGRREVGHGSLAER

AIIPVLPPKDSFPYVLRVVSEVLSSNGSTSMGSVCGSTLALMDAGVPLKAPVSGAAMGLIKEDAEIRILT

DIQGIEDFLGDMDFKVAGTKDGITALQMDMKITGLPVKTIAEAVNQARPARIHILEKMLEAIDAPRTTLS

PHAPRLLSFRIDPELIGTVIGPGGRTIKGITERTNTKIDIEDGGIVTIASHDGAAAEAAQRIIEGLTRKV

NEGEVFTGTITRIIPIGAFVEILPGKEGMIHISQLSEARVEKVDDVVKVGDQVTVRIREIDNRGRINLTL

RGVPQNGEETQSEPAPTPVAPLN

>gi|300582223|emb|CBV22375.1| unnamed protein product [Prochlorococcus marinus str. MIT 9313]

MALVLLPGLNNLIEDISEEKKLPTQVVEAALREALLKGYERYRRTLYLGISEDPFEEEYFSNFDVGLELE

DEGYRVLASKIIVEEVESEDHQIALQEVMQVAEDAQIGDTVVLDVTPEKEDFGRMAAATTKQVLAQKLRD

QQRRMIQEEFADLEDPVLTARVIRFERHSVIMAVSSGLGRPEVEAELPRRDQLPNDNYRANATFKVFLKE

VSEVPRRGPQLFVSRSNAGLVVYLFENEVPEIQEGSVRIVAVAREANPPSRSVGPRTKVAVDSIEREVDP

VGACIGARGSRIQQVVNELRGEKIDVIRWSPDPGQYIANSLSPARVEMVRLVDPEGQHAHVLVPPDQLSL

AIGREGQNVRLAARLTGWKIDIKNSQEYDQASEDTTVAELISQREEEEALQRDAEARLAAEQATRAEEDA

RLRELYPLPEDEEEYDQEEPAKTMAEDENESDGQSDDLSNQPDASSEQRSNEESLEEEDRAR

>gi|300575635|emb|CBV26319.1| unnamed protein product [Prochlorococcus marinus str. MIT 9313]

MALVLLPGLNNLIEDISEEKKLPTQVVEAALREALLKGYERYRRTLYLGISEDPFEEEYFSNFDVGLELE

DEGYRVLASKIIVEEVESEDHQIALQEVMQVAEDAQIGDTVVLDVTPEKEDFGRMAAATTKQVLAQKLRD

QQRRMIQEEFADLEDPVLTARVIRFERHSVIMAVSSGLGRPEVEAELPRRDQLPNDNYRANATFKVFLKE

VSEVPRRGPQLFVSRSNAGLVVYLFENEVPEIQEGSVRIVAVAREANPPSRSVGPRTKVAVDSIEREVDP

VGACIGARGSRIQQVVNELRGEKIDVIRWSPDPGQYIANSLSPARVEMVRLVDPEGQHAHVLVPPDQLSL

AIGREGQNVRLAARLTGWKIDIKNSQEYDQASEDTTVAELISQREEEEALQRDAEARLAAEQATRAEEDA

RLRELYPLPEDEEEYDQEEPAKTMAEDENESDGQSDDLSNQPDASSEQRSNEESLEEEDRAR

>gi|300565519|emb|CBV14284.1| unnamed protein product [Prochlorococcus marinus str. MIT 9313]

MALVLLPGLNNLIEDISEEKKLPTQVVEAALREALLKGYERYRRTLYLGISEDPFEEEYFSNFDVGLELE

DEGYRVLASKIIVEEVESEDHQIALQEVMQVAEDAQIGDTVVLDVTPEKEDFGRMAAATTKQVLAQKLRD

QQRRMIQEEFADLEDPVLTARVIRFERHSVIMAVSSGLGRPEVEAELPRRDQLPNDNYRANATFKVFLKE

VSEVPRRGPQLFVSRSNAGLVVYLFENEVPEIQEGSVRIVAVAREANPPSRSVGPRTKVAVDSIEREVDP

VGACIGARGSRIQQVVNELRGEKIDVIRWSPDPGQYIANSLSPARVEMVRLVDPEGQHAHVLVPPDQLSL

AIGREGQNVRLAARLTGWKIDIKNSQEYDQASEDTTVAELISQREEEEALQRDAEARLAAEQATRAEEDA

RLRELYPLPEDEEEYDQEEPAKTMAEDENESDGQSDDLSNQPDASSEQRSNEESLEEEDRAR

>gi|259705035|emb|CBG08853.1| unnamed protein product [Prochlorococcus marinus str. MIT 9313]

MALVLLPGLNNLIEDISEEKKLPTQVVEAALREALLKGYERYRRTLYLGISEDPFEEEYFSNFDVGLELE

DEGYRVLASKIIVEEVESEDHQIALQEVMQVAEDAQIGDTVVLDVTPEKEDFGRMAAATTKQVLAQKLRD

QQRRMIQEEFADLEDPVLTARVIRFERHSVIMAVSSGLGRPEVEAELPRRDQLPNDNYRANATFKVFLKE

VSEVPRRGPQLFVSRSNAGLVVYLFENEVPEIQEGSVRIVAVAREANPPSRSVGPRTKVAVDSIEREVDP

VGACIGARGSRIQQVVNELRGEKIDVIRWSPDPGQYIANSLSPARVEMVRLVDPEGQHAHVLVPPDQLSL

AIGREGQNVRLAARLTGWKIDIKNSQEYDQASEDTTVAELISQREEEEALQRDAEARLAAEQATRAEEDA

RLRELYPLPEDEEEYDQEEPAKTMAEDENESDGQSDDLSNQPDASSEQRSNEESLEEEDRAR

>gi|259700851|emb|CBG00764.1| unnamed protein product [Prochlorococcus marinus str. MIT 9313]

MALVLLPGLNNLIEDISEEKKLPTQVVEAALREALLKGYERYRRTLYLGISEDPFEEEYFSNFDVGLELE

DEGYRVLASKIIVEEVESEDHQIALQEVMQVAEDAQIGDTVVLDVTPEKEDFGRMAAATTKQVLAQKLRD

QQRRMIQEEFADLEDPVLTARVIRFERHSVIMAVSSGLGRPEVEAELPRRDQLPNDNYRANATFKVFLKE

VSEVPRRGPQLFVSRSNAGLVVYLFENEVPEIQEGSVRIVAVAREANPPSRSVGPRTKVAVDSIEREVDP

VGACIGARGSRIQQVVNELRGEKIDVIRWSPDPGQYIANSLSPARVEMVRLVDPEGQHAHVLVPPDQLSL

AIGREGQNVRLAARLTGWKIDIKNSQEYDQASEDTTVAELISQREEEEALQRDAEARLAAEQATRAEEDA

RLRELYPLPEDEEEYDQEEPAKTMAEDENESDGQSDDLSNQPDASSEQRSNEESLEEEDRAR

>gi|259679371|emb|CBG12805.1| unnamed protein product [Prochlorococcus marinus str. MIT 9313]

MALVLLPGLNNLIEDISEEKKLPTQVVEAALREALLKGYERYRRTLYLGISEDPFEEEYFSNFDVGLELE

DEGYRVLASKIIVEEVESEDHQIALQEVMQVAEDAQIGDTVVLDVTPEKEDFGRMAAATTKQVLAQKLRD

QQRRMIQEEFADLEDPVLTARVIRFERHSVIMAVSSGLGRPEVEAELPRRDQLPNDNYRANATFKVFLKE

VSEVPRRGPQLFVSRSNAGLVVYLFENEVPEIQEGSVRIVAVAREANPPSRSVGPRTKVAVDSIEREVDP

VGACIGARGSRIQQVVNELRGEKIDVIRWSPDPGQYIANSLSPARVEMVRLVDPEGQHAHVLVPPDQLSL

AIGREGQNVRLAARLTGWKIDIKNSQEYDQASEDTTVAELISQREEEEALQRDAEARLAAEQATRAEEDA

RLRELYPLPEDEEEYDQEEPAKTMAEDENESDGQSDDLSNQPDASSEQRSNEESLEEEDRAR

>gi|207007207|emb|CAR80125.1| unnamed protein product [Prochlorococcus marinus str. MIT 9313]

MQGQTQSISFDGREIRLTTGRYAPQAGGSVMMECGDTAVLVTATRSTGREGIDFLPLICDYEERLYAAGR

IPGSFMRREGRPPERATLIARLIDRPMRPLFPSWMRDDLQIVATCLSLDERVPADVLAVTGASMATLLAS

IPFQGPMAAVRVGLLGDDFVLNPSYREIERGDLDLVVAGTPDGVVMVEAGANQLPQGDVIEAIDFGYEAV

CELIKAQQTILKDAGIKQVQPEPPTQDQDTKLSTYLEKNCSKSIGEVLKQFEQTKAERDSKLDAIKAKTA

EAIDSLKEDDAVRKSVNANSKVLSNNFKALTKKLMREQIIKQGKRVDGRKLDEVRPISSAAGVLPKRVHG

SGLFQRGLTQVLSTATLGTPSDAQEMDDLNPGPEKTYLHHYNFPPYSVGETRPMRSPGRREVGHGSLAER

AIIPVLPPKDSFPYVLRVVSEVLSSNGSTSMGSVCGSTLALMDAGVPLKAPVSGAAMGLIKEDAEIRILT

DIQGIEDFLGDMDFKVAGTKDGITALQMDMKITGLPVKTIAEAVNQARPARIHILEKMLEAIDAPRTTLS

PHAPRLLSFRIDPELIGTVIGPGGRTIKGITERTNTKIDIEDGGIVTIASHDGAAAEAAQRIIEGLTRKV

NEGEVFTGTITRIIPIGAFVEILPGKEGMIHISQLSEARVEKVDDVVKVGDQVTVRIREIDNRGRINLTL

RGVPQNGEETQSEPAPTPVAPLN

>gi|33640780|emb|CAE21235.1| putative ribosomal-protein-alanine acetyltransferase [Prochlorococcus marinus str. MIT 9313]

MLKHDIQAMEVIHLGPEQINACMELNQLALNGLWSKQQWIQELIDSRSLCMGVLKSSTLLALACGWLVVD

ELHLTAIGVHPQHRRQGLARLLLSKLLEQGQRTGAIHATLEVARNNSAARGLYESCGFKTAGCRHHYYSN

GQDALIQWLSLGKQAEPRQKI

>gi|33635376|emb|CAE21701.1| N utilization substance protein A [Prochlorococcus marinus str. MIT 9313]

MALVLLPGLNNLIEDISEEKKLPTQVVEAALREALLKGYERYRRTLYLGISEDPFEEEYFSNFDVGLELE

DEGYRVLASKIIVEEVESEDHQIALQEVMQVAEDAQIGDTVVLDVTPEKEDFGRMAAATTKQVLAQKLRD

QQRRMIQEEFADLEDPVLTARVIRFERHSVIMAVSSGLGRPEVEAELPRRDQLPNDNYRANATFKVFLKE

VSEVPRRGPQLFVSRSNAGLVVYLFENEVPEIQEGSVRIVAVAREANPPSRSVGPRTKVAVDSIEREVDP

VGACIGARGSRIQQVVNELRGEKIDVIRWSPDPGQYIANSLSPARVEMVRLVDPEGQHAHVLVPPDQLSL

AIGREGQNVRLAARLTGWKIDIKNSQEYDQASEDTTVAELISQREEEEALQRDAEARLAAEQATRAEEDA

RLRELYPLPEDEEEYDQEEPAKTMAEDENESDGQSDDLSNQPDASSEQRSNEESLEEEDRAR

>gi|33635227|emb|CAE21552.1| polyribonucleotide nucleotidyltransferase [Prochlorococcus marinus str. MIT 9313]

MQGQTQSISFDGREIRLTTGRYAPQAGGSVMMECGDTAVLVTATRSTGREGIDFLPLICDYEERLYAAGR

IPGSFMRREGRPPERATLIARLIDRPMRPLFPSWMRDDLQIVATCLSLDERVPADVLAVTGASMATLLAS

IPFQGPMAAVRVGLLGDDFVLNPSYREIERGDLDLVVAGTPDGVVMVEAGANQLPQGDVIEAIDFGYEAV

CELIKAQQTILKDAGIKQVQPEPPTQDQDTKLSTYLEKNCSKSIGEVLKQFEQTKAERDSKLDAIKAKTA

EAIDSLKEDDAVRKSVNANSKVLSNNFKALTKKLMREQIIKQGKRVDGRKLDEVRPISSAAGVLPKRVHG

SGLFQRGLTQVLSTATLGTPSDAQEMDDLNPGPEKTYLHHYNFPPYSVGETRPMRSPGRREVGHGSLAER

AIIPVLPPKDSFPYVLRVVSEVLSSNGSTSMGSVCGSTLALMDAGVPLKAPVSGAAMGLIKEDAEIRILT

DIQGIEDFLGDMDFKVAGTKDGITALQMDMKITGLPVKTIAEAVNQARPARIHILEKMLEAIDAPRTTLS

PHAPRLLSFRIDPELIGTVIGPGGRTIKGITERTNTKIDIEDGGIVTIASHDGAAAEAAQRIIEGLTRKV

NEGEVFTGTITRIIPIGAFVEILPGKEGMIHISQLSEARVEKVDDVVKVGDQVTVRIREIDNRGRINLTL

RGVPQNGEETQSEPAPTPVAPLN

>gi|225785254|emb|CAX32264.1| Hypothetical protein PMT_2742 [Prochlorococcus marinus str. MIT 9313]

MRLAYAWALSFRFELIQIQMSRYHCPFCSSRYQIHQQRADGVMVCGQCGDPLVKVPLIRPTQIIGLVAAA

AFIAPLLLMVFVFIQDEQRPELKRPLSKMAAVSSPVDICSWLS

>gi|33640910|emb|CAE21366.1| possible acetyltransferase [Prochlorococcus marinus str. MIT 9313]

MISSSSLTPEVLEQAYGHRARECPSSNEQINLVFSQDRSFDLVELEQLLEAVGWSRRPMRRVRLALDHTL

LKVGLWRHDPLFPRLVGFARCTGDGVLEATVWDVAIHPIYQGVGLGKHLMDYTLESLKEMGVKRVTLFAD

PGVVDFYERQGWTLEPDGHKCAFWYA

>gi|33640561|emb|CAE20350.1| possible Fe-S oxidoreductase [Prochlorococcus marinus str. MIT 9313]

MTKPALRSDIPMKPTVHKQEKPSVAFAHLGCEKNRVDTEHMLGLLTEAGYSVSSDENDAAVVVVNTCSFI

QDAREESVRTLIGLAEQGKELIIAGCLAQHFQEELLESIPEAKAIVGTGDYQHIVDVLKRVEAGERVNHV

SEFPTFVGDETLPRQRTTDQAVAYLKVAEGCDYRCAFCIIPKLRGDQRSRPVESIVTEAHQLAEQGVQEL

ILISQITTNYGLDLYGKPKFAELLQALGEVDIPWVRVHYAYPTGLTPEVLAAYREVPNVLRYLDLPLQHS

HPEVLRAMNRPWQTDVNERLLDRIREQLPDAVLRTTLIVGFPGETEDHFNHLAAFIERQRFDHVGVFTFS

PEDGTAAADLPDRVDPSIAAARKDRLMALQQPISAERNQRWVGRTIDVLIEQHNPETGAMIGRCDRFAPE

VDGEVLVLPSEKGLQASPGTMVPVFITGSDVYDLTGQLVDTNAMAVTAQTSQ

>gi|33634623|emb|CAE20609.1| lipase family protein [Prochlorococcus marinus str. MIT 9313]

MDKPRRPLVLVHGLWDTPHLFRRLVKALEQHQLPLQIPHLPHRLGAVPLSKLAETLDQLIVEQWGAETVI

DVLGFSMGGVISRIWLQQLGGSRRTHRFLSVGSPQRGTLTAQWIPACLFAGLADMKRGSPLLRKLNGDVS

ALEDIECSSYFCRWDVMVVPGWQAVLPVGEQQAVPVITHQQLMSHPLALKLVISKLLSN

>gi|61215247|sp|Q7V477.1|RL10_PROMM RecName: Full=50S ribosomal protein L10

MGRTLESKQQIVEELKGLLGEAEMALVLDYQGLSIKEMSDLRTRLQASNGVCKVTKNTLMRHAINGNGAW

SNLESLLTGTNAFVLIKGDVGGAVKAVQAFQKDTKKSETKGGLFEGKLLSQGEIKAIGDLPTKEVLMAQI

AGSLNALATKVAVGINEVPSGLARALHQHAESGES

>gi|81712666|sp|Q7V531.1|RL6_PROMM RecName: Full=50S ribosomal protein L6

MSRIGKNPIPIPDKVAVTLDGLAVSVKGPKGELSRTLPEGVSVSQVENTIVVTPTSQKRKSRERHGLCRS

LVANMVEGVSKGYTRKLELIGVGSRAQVKGKKLVVSAGFSHPVEMDPPEGVTFAVENNTNVTVSGADKEL

VGNEAAKIRAIRPPEPYKGKGIRYEGERILRKAGKSGKK

>gi|81576665|sp|Q7V406.1|RL9_PROMM RecName: Full=50S ribosomal protein L9

MAKRVQVVLNEDVLSLGKDGDLVEVAPGYARNFLLPFGKAVPVTPAVMKQVGHRRAKQAEHQAAIKQEAL

DFQTALVTIGRFTVKKQTGEDDVLFGTVTNGDVAEAIETATKKEIDRRNIIVPEIHRTGSYKVQVKLHNE

VNAEINLEVVSY

>gi|73919089|sp|Q7V529.1|RS5_PROMM RecName: Full=30S ribosomal protein S5

MTQPNTQTTPNDVPAAAEGQQEQQQQQRRGGGRERRGGGRRGDRRGQERDSEWQERVVQIRRVSKTVKGG

KKMSFRAIVVVGNERGQVGVGVGKAGDVIGAVRKGVADGKKHLVKVPLTRHNSIPTLSNGRDGAANVLIR

PAAPGTGVIAGGSIRTVLELAGIKNVLAKRLGSKTPLNNARAAMVALASLRTHKETAKERGISLEQIYS

>gi|38605173|sp|Q7V479.1|RL11_PROMM RecName: Full=50S ribosomal protein L11

MAKKVVSVIKLALQAGKANPAPPVGPALGQHGVNIMAFCKEYNARTQDKAGLVIPVEISVFEDRSFTFIT

KTPPASVLITKAAGIEKGSGESAHGKVGSLSRSQLEEIAKTKLPDLNCTSIESAMRIIEGTARNMGVSIS

D

>gi|81576719|sp|Q7V476.1|RL7_PROMM RecName: Full=50S ribosomal protein L7/L12

MSKKTDDILDSLKTLSLLEASELVKQIEDAFGVSAAASAGVVVAAGGAAGGGAAAEAAEEQTEFDVVLES

FDASAKIKVLKAVREATGLGLGDAKAMVEAAPKTIKEGIAKNDAEALKKAIEEVGGKVSLK

>gi|61215701|sp|Q7V537.1|RS3_PROMM RecName: Full=30S ribosomal protein S3

MGHKIHPTGLRLGITQEHRSRWYATSKMYPILLQEDDRIRRFIHKKYGAAGISDVLIARKADQLEVELKT

ARPGVLVGRQGSGIEELRTGIQKTIGDHSRQVRINVVEVERVDADAFLLAEYIAQQLEKRVAFRRTIRMA

VQRAQRAGVLGLKIQVGGRLNGAEIARTEWTREGRVPLHTLRAEIDYATKVASTTYGVLGIKVWIFKGEV

LGDEAQTMPVGASPRRRGNRRPQQFEDRSNEG

>gi|61215699|sp|Q7V478.1|RL1_PROMM RecName: Full=50S ribosomal protein L1

MPKLSKRITGLLAKVEDRVYQPLEAIQLVKENATAKFDETIEAHVRLGIDPKYTDQQLRTTVALPQGTGQ

SVRIAVISRGEKLAEAKTAGAELAGDDDLVESIGKGQMDFDLLIATPDMMPKVAKLGRVLGPRGLMPNPK

AGTVTTDLAAAIKEFKAGKLEFRADRAGIVHVRFGKASFSADALLENLKTLQETIDRNKPSGAKGRYWKS

LYITSTMGPSVEVDVTALQDLEEDA

>gi|51316776|sp|Q7V538.1|RL22_PROMM RecName: Full=50S ribosomal protein L22

MTTSSPTTTTIAKAHGRFIRGSVSKVRRVLDQIRGRTYRDALIMLEFMPYRSTGPITKVLRSAVANAEHN

LGLDPASLVIAQASADMGPSMKRYRPRAQGRAFAIKKQTCHISIAVAAQTDS

>gi|50401254|sp|Q7V533.1|RL5_PROMM RecName: Full=50S ribosomal protein L5

MSLKQRYRETIQPKLLKDLSLSNIHEVPKVLKITVNRGLGEAAQNAKSLEASITELATITGQKVVVTRAK

KAIAGFKIRQGMPIGCAVTLRGERMYAFLERLINLALPRIRDFRGVSPKSFDGRGNYTLGVREQLIFPEI

SFDKIDAIRGMDITIVTSARTDEEGQSLLREMGMPFRSN

>gi|41017827|sp|Q7V8U9.1|RS4_PROMM RecName: Full=30S ribosomal protein S4

MSRYRGPRLRITRRLGDLPGLTRKAAKRSHPPGQHGQARRKRSEYAIRLEEKQKLRFNYGISERQLVRYV

KKARAQDGSTGTNLLKLLENRLDNVCFRLGFGPTVPGARQLVNHGHVTVNGRVLDIASYQCKAGDVVAIR

ERKGSKKLAEANLEFPGLANVPPHIELDKAKMSAKIISKCEREWVALEINELLVVEYYSRKV

>gi|59798837|sp|Q7TUR7.1|RS20_PROMM RecName: Full=30S ribosomal protein S20

MANNKSSKKRVQIAERNRLENKSYKSAMRTLMKRCFSACSHYSQQPGETAKANVKASIDSAFSKIDKAVK

RGVLHRNTAAHQKSRLSAAVKQAIEPAPST

>gi|54036290|sp|Q7V998.1|RL35_PROMM RecName: Full=50S ribosomal protein L35

MPKLKTRKAAAKRFKATVTGKFMRRRAFRNHLLDHKSPKLKRHLATKAVVDERDAENVRLMLPYA

>gi|81712003|sp|Q7TUX0.1|RS21_PROMM RecName: Full=30S ribosomal protein S21

MAQVTVGENEGVESALRRFKRAVSKAGIFSDLKRIRHHETPVEKYKRKAQQRRRSRRR

>gi|67461502|sp|Q7V7P3.1|RL28_PROMM RecName: Full=50S ribosomal protein L28

MSRVCQLTGTRANNGMAVSHSHIRTKKLQQANLQQRRLWWAEGNRWLKLRVSTRALKTIQKKGLGVYAKS

LGIDLNKI

>gi|81835672|sp|Q7V540.1|RL23_PROMM RecName: Full=50S ribosomal protein L23

MTERFNDRLADVIRRPLITEKATSALEQNQYTFEVDHRAAKPDIKAAVEQLFDVRVVGISTMNPPRRSRR

VGRFTGKRAQVKKAIVRLAEGNTIQLFPES

>gi|81834830|sp|Q7TUP4.1|RS17_PROMM RecName: Full=30S ribosomal protein S17

MALKERLGTVVSDKMDKTVVVAVENRFPHPIYQKTVSRTTRYKAHDAGNTCRVGDRVRITETRPLSRSKR

WTVAEVLSHSPKAQEVST

>gi|81712708|sp|Q7V7K4.1|RL33_PROMM RecName: Full=50S ribosomal protein L33

MAKNKGVRIVITLECNECRSNPAKRSPGVSRYTTEKNRRNTTERLEIKKFCPHCNKSTPHKEIK

>gi|81712679|sp|Q7V5W5.1|RL21_PROMM RecName: Full=50S ribosomal protein L21

MAEKPTAKPKAAEAKDQSDSYAIVEASGQQFLLQPNRYYDLDRLQAAVDDTVTLEKVLLIKDGKNDATVG

QPYVKGASVELKVMDHRRGPKIIVYKMRPKKKTRRKNGHRQELTRVMVQSISIDGKALS

>gi|73621816|sp|Q7V519.1|RL31_PROMM RecName: Full=50S ribosomal protein L31

MPKPDIHPNWYPDAKVICNGEVVMTTGSTQPELHVDVWSGNHPFFTGTQKILDTEGRVDRFMRKYGMGSA

DAAADEKKPDAKNNNKDNTSKED

>gi|67461181|sp|Q7V532.1|RS8_PROMM RecName: Full=30S ribosomal protein S8

MANHDPISDMLTRIRNASEKRHQTTRVPASRMSRSIAKVLQQEGFISEISEEGEGVRTHLVLELKYSGKH

RHPTIRSMQRVSKPGLRIYKNTRALPKVLGGLGMAIISTSKGVMSDRDARKQGVGGEVLCYVY

>gi|62287312|sp|Q7V502.1|RS7_PROMM RecName: Full=30S ribosomal protein S7

MSRRNAAEKRPVLPDPQFNNRLATMMISRLMKHGKKSTAQRILAQAFGLINERTGGDPIELFETAVKNAT

PLVEVRARRVGGATYQVPMEVRQERGTAMALRWLVNFSRARNGRSMSQKLAAELMDAANEAGSAVRKREE

THKMAEANKAFAHYRY

>gi|61216285|sp|Q7V3T1.1|RS6_PROMM RecName: Full=30S ribosomal protein S6

MSQQPYYETMYILRPDIPEEEVETHVTKYREMVTEAGAEVLDNQMRGKRRLAYPISNHKEGIYVQLSHNG

NGQQVAVLEKAMRLSEDVIRYLTVKQEGPLPAPRIVPGSEPEPVQQQEAAAVEA

>gi|61215702|sp|Q7V7Z4.1|RS2_PROMM RecName: Full=30S ribosomal protein S2

MAVVTLSEMMEAGAHFGHQTRRWNPKMSRYIYCARNGVHIIDLVQTAICMNNAYKWTRSSARSGKRFLFV

GTKKQASEVVALEATRCGASYVNQRWLGGMLTNWTTMKARIDRLKDLERMESSGAIAMRPKKEASVLRRE

LERLQKYLGGLKGMRRLPDVVVLVDQRRETNAVLEARKLDIPLVSMLDTNCDPDLCEVPIPCNDDAVRSV

QLVLGRLADAINEGRHGTNDQRGADDSDD

>gi|59798838|sp|Q7TUZ8.1|RS18_PROMM RecName: Full=30S ribosomal protein S18

MSSSFFKKRLSPIKPGDPIDYKDVDLLKKFITDRGKILPRRLTGLTSKQQRDLTNAVKRARIIALLPFVN

PEG

>gi|59798836|sp|Q7TUP2.1|RL36_PROMM RecName: Full=50S ribosomal protein L36

MKVRASVKKMCDKCRVIRRHGRVMVICSTPKHKQRQG

>gi|54036401|sp|Q7TUP5.1|RS19_PROMM RecName: Full=30S ribosomal protein S19

MGRSLKKGPFIADSLLRKLEKQNADDDKSVIKTWSRASTILPMMIGHTIAVHNGRSHVPVFITEQMVGHK

LGEFAPTRTFKGHIKDKKGGR

>gi|50403612|sp|Q7TV43.1|RS16_PROMM RecName: Full=30S ribosomal protein S16

MIKLRLKRFGKKREASFRLVACNSTSRRDGRPLQELGFYNPRTKETRLDTEALRLRLSQGAQPTDAVRSL

LEKGGLIEKTVRPAEVVGKAKQAEARKAAAKNVAKQAAEAKAEETPADNTEA

>gi|46577197|sp|Q7V541.1|RL4_PROMM RecName: Full=50S ribosomal protein L4

MAECVVHDWQGKEAGKASLELKVSKETTAVDLMHRAVLRQQAHSRQGTASTLTRAEVRGGGRKPYKQKGT

GRARQGTIRTPLRPGGGIIFGPKPRTYNLAMNRKERRLALRTALMARIEDVIVVKDFGNSLKVPKTREIS

DALIRLGLAADAKVLIILSTPSEIIRRSVRNLEKVKLIAADQLNVFDLLHANSLVLSEEALAKIQEVYGD

D

>gi|46396795|sp|Q7V534.1|RL24_PROMM RecName: Full=50S ribosomal protein L24

MPIATPKQKTTQRIKMRIHKGDTVQVITGKDKGKTGEVLRTLPIENRVIVQGVNIRTRHVKPTQEGESGR

IVTEEASVHASNVMLYSNKKKIASRVELVVEKDGSKKRRLKKTGELID

>gi|42559599|sp|Q7V542.1|RL3_PROMM RecName: Full=50S ribosomal protein L3

MSIGILGKKLGMSQFFDDQGRAIPVTLIEAGPCRITQLKTSEIDGYAAVQIGFGDTREKLINKPSKGHLT

KSGEVLLKHLREYRVEGLEGLELGAAITVGSFEAGQKVDVSGDTMGRGFAGYQKRHGFSRGPMSHGSKNH

REPGSTGAGTTPGRIYPGKRMAGRYGGKKRTTRGLTILKVDSNRNLLVVKGSVPGKPGALLNIRPAKRVG

NKPAQGGK

>gi|42559204|sp|Q7V539.1|RL2_PROMM RecName: Full=50S ribosomal protein L2

MAIRTFRPYTPGTRTRVVTDFNEVTGRKPERSLVVAKHRRKGRNNRGVITCRHRGGGHKRLYRIVDFRRN

KHGIPAKVAAIHYDPHRKAHLALLFYTDGEKRYILAPAGIAIGQQLISGPESPIETGNALPLSAIPLGSS

VHNVELYAGRGGQMARTAGSSAQVMAKEGDYVALKLPSTEVRLVRHECYATLGEVGNSEVRNTSLGKAGR

RRWLGRRPQVRGSVMNPCDHPHGGGEGRAPIGRSGPVTPWGKPALGLKTRKRNKPSNRFVLRKRRRTSKR

SRGGRDS

>gi|39932297|sp|Q7V5W4.1|RL27_PROMM RecName: Full=50S ribosomal protein L27

MAHKKGTGSTRNGRDSNSKRLGVKAYGGETVTAGSILIRQRGTSVLPGVNVGQGKDDTLFALTDGVVAFE

SIRRSLRNRKRISVVASS

>gi|39932087|sp|Q7V520.1|RS9_PROMM RecName: Full=30S ribosomal protein S9

MSSSNNSVVYWGTGRRKTSVARVRLVPGSGTITINGRPGDHYLNFNPAYLAAVKAPLQTLGLNEQYDVLV

NVHGGGLTGQADAIKQGAARALCELSADNRKPLKTEGHLSRDPRAKERRKYGLKKARKAPQFSKR

>gi|39931789|sp|Q7V999.1|RL20_PROMM RecName: Full=50S ribosomal protein L20

MARVKRGNVARKRRNKILRLARGFQGSNGSLFRTANQRVMKALCNAYRDRRRRKRDFRRLWIARINAAAR

INGVSYSRLIGGLKKADVRINRKMLAQLAVMDPKSFTSVVTSAKS

>gi|39931784|sp|Q7V668.1|RL19_PROMM RecName: Full=50S ribosomal protein L19

MTADSKDTSMSEDNTETATAIENSSAMVTDVTSKSAPNVRLSPDALIKEFEASQQKSDLNDIYVGDTVRV

GVRISEGNKERIQPYEGVVIAKRHGGIHETITVRRIFQGIGVERVFMLHSPQVASIKVERRGKVRRAKLF

YLRERVGKATRVKQRFDR

>gi|81712665|sp|Q7V528.1|RL15_PROMM RecName: Full=50S ribosomal protein L15

MTTLRLDSLKANVGARRRKMRKGRGIAAGQGASCGFGMRGQKSRSGRPTRPGFEGGQMPLYRRVPKLKHF

TTVNSKEFTVVNVAALNELKAGSTINLDTLVKNGVVTSPKYPLKVLGNGELKVKLTIQAAAFTATARSKI

EAAGGTCEILD

>gi|81712663|sp|Q7V522.1|RL17_PROMM RecName: Full=50S ribosomal protein L17

MRHQCRVPKLGRPTDQRKAMLRGLTTQLIREGRVTTTKARAKALRDEAERMITLAKNGSLASRRRAIGYI

YDKQLVHALFDKAQDRYGDRQGGYTRIIRTVPRRGDNAEMAIIELV

>gi|81712662|sp|Q7V521.1|RL13_PROMM RecName: Full=50S ribosomal protein L13

MNKTSVPSIDSIERQWFLVDAENQTLGRLATEVASVLRGKNKPSFTPHLDTGDFVVVVNADKIRVSGNKA

NQKLYRRHSGRPGGMKVETFQALQDRLPERIVEKAIKGMLPHNALGRQLFRKLKVYRGPEHPHSAQRPQT

LQLNPAASSQ

>gi|81712004|sp|Q7TV07.1|RS15_PROMM RecName: Full=30S ribosomal protein S15

MSLDTTEKQQLINANQTHGTDTGSVEVQVAMLSERITKLSSHLQENKHDFSSRQGLLKMIGRRKRLLSYV

RGKSEQRYNGLITKLGIRG

>gi|81711999|sp|Q7TUP3.1|RL14_PROMM RecName: Full=50S ribosomal protein L14

MIQQESFLTVADNSGAKRIQCIRVLGSNRRYAHVGDVIVAAVKDAMPNMSVKKSEVVKAVVVRTKATLRR

DTGNSIRFDDNAAVLINEDKNPRGTRVFGPVARELRERNFTKIVSLAPEVI

>gi|81576938|sp|Q7V525.1|RS13_PROMM RecName: Full=30S ribosomal protein S13

MARIAGVDIPRDKRVEVALTYIYGIGLTRAKTILTKSDVNPDIRVKDLEDGDVQKLRTALEAFTIEGDLR

RQEGMALKRLQDIGCLRGRRHRMSLPVRGQRTRTNARTRRGARKTVAGKKK

>gi|81573131|sp|Q7TUT2.1|RS14_PROMM RecName: Full=30S ribosomal protein S14

MAKKSMIARDVKRKKIVERYAAKRAALMEAFNAAKDPMQRLEIHRKIQALPRNSAPNRIRNRCWATGKPR

GVYRDFGLCRNQLRERAHKGELPGVVKSSW

>gi|73621671|sp|Q7V530.1|RL18_PROMM RecName: Full=50S ribosomal protein L18

MSNLSRKQQTQKRHRRLRRHLKGTAQRPRLAVFRSNNHIYAQVIDDEAQNTLCAASTLDKDLRTSLKADG

SSCDASNAVGDLVAKRALAKGIQQVVFDRGGNLYHGRVKSLADAAREAGLQF

>gi|59798849|sp|Q7V524.1|RS11_PROMM RecName: Full=30S ribosomal protein S11

MAKPTKKTGSKKTKRNVPNGVAHIQSTFNNTIVSIADTAGEVIAWSSAGASGFKGARKGTPFAAQTAAEA

AARRALEQGMRQIEVLVRGPGSGRETAIRALQVAGLEITLIRDVTPLPHNGCRRPKRRRV

>gi|52783392|sp|Q7V503.1|RS12_PROMM RecName: Full=30S ribosomal protein S12

MPTIQQLIRTERQHLTRKTKSPALRACPERRGVCTRVYTSTPKKPNSALRKVARVRLTSGFEVTAYIPGI

GHNLQEHSVVLIRGGRVKDLPGVRYHIIRGTLDTAGVKDRSQSRSKYGAKASKQD

>gi|44888413|sp|Q7V4Z9.1|RS10_PROMM RecName: Full=30S ribosomal protein S10

MSTAIAQQKIRIRLKAFDRRMLDLSCDKIIETADNTAATAIGPIPLPTKRKIYCVLCSPHVDKDSREHFE

TRTHRRIIDIYNPSAKTIDALMKLDLPSGVDIEVKL

>gi|71649175|sp|Q7V613.1|RL34_PROMM RecName: Full=50S ribosomal protein L34

MTKRTFGGTSRKRKRVSGFRVRMRNHTGRRVIRSRRKRGRARLAV

>gi|46577391|sp|Q7V5U0.2|RRP3_PROMM RecName: Full=Probable 30S ribosomal protein PSRP-3; AltName: Full=Ycf65-like protein

MRGGSAALATATIDADGVPSGYTPKADEGRFLLKILWLPDNVALAVDQIVGGGPSPLTAYFFWPREDAWE

TLKGELEGKSWITDNERVEVLNKATEVINYWQEEGKGKNLEEAKLKFPDVTFCGTA

>gi|51316779|sp|Q7V7I8.1|RL32_PROMM RecName: Full=50S ribosomal protein L32

MAVPKKKTSKGKRNQRHAIWKAKAATAAQRALSIGKSVLSGRAQGFVYPMQESDDDES

>gi|73917120|sp|Q7V535.1|RL29_PROMM RecName: Full=50S ribosomal protein L29

MAHPKAAEVRKLTDADITEQIDGIRRELFDLRFQQATRQLSNTHRFKEARIKLAQLLTVQKERSRSAASS

>gi|81576939|sp|Q7V536.1|RL16_PROMM RecName: Full=50S ribosomal protein L16

MLSPKRVKFRKQQRGRMRGVATRGNTIAFGEFALQAQECGWITSRQIEASRRAMTRYVKRGGKIWIRIFP

DKPVTMRPAETRMGSGKGNPEFWVAVIKPGRILFEMGGAEITEDIAKEAMRLAQYKLPIKTKFIGLDDQE

KVAGSDKPASVPAITAES

>gi|81577992|sp|Q7V8Z5.1|RIMO_PROMM RecName: Full=Ribosomal protein S12 methylthiotransferase RimO; Short=S12 MTTase; Short=S12 methylthiotransferase; AltName: Full=Ribosomal protein S12 (aspartate-C(3))-methylthiotransferase; AltName: Full=Ribosome maturation factor RimO

MTKPALRSDIPMKPTVHKQEKPSVAFAHLGCEKNRVDTEHMLGLLTEAGYSVSSDENDAAVVVVNTCSFI

QDAREESVRTLIGLAEQGKELIIAGCLAQHFQEELLESIPEAKAIVGTGDYQHIVDVLKRVEAGERVNHV

SEFPTFVGDETLPRQRTTDQAVAYLKVAEGCDYRCAFCIIPKLRGDQRSRPVESIVTEAHQLAEQGVQEL

ILISQITTNYGLDLYGKPKFAELLQALGEVDIPWVRVHYAYPTGLTPEVLAAYREVPNVLRYLDLPLQHS

HPEVLRAMNRPWQTDVNERLLDRIREQLPDAVLRTTLIVGFPGETEDHFNHLAAFIERQRFDHVGVFTFS

PEDGTAAADLPDRVDPSIAAARKDRLMALQQPISAERNQRWVGRTIDVLIEQHNPETGAMIGRCDRFAPE

VDGEVLVLPSEKGLQASPGTMVPVFITGSDVYDLTGQLVDTNAMAVTAQTSQ

>gi|38605164|sp|Q7TUS7.1|PRMA_PROMM RecName: Full=Ribosomal protein L11 methyltransferase; Short=L11 Mtase

MNFAAALRWWRLSLPIADELEESLIWKLTDLGLCRLAVQHAPENSERTLLAWLPSSEWSESDRDQLMANL

RPLAEPFGLKLANPTWCEVADEDWSLNWKQDWQPDPVGQRLLILPAWLDLPQEYADRFVVRLDPGSAFGT

GSHPSTRLCLEALERNPPLGLRVADLGCGSGVLGFAALAFGARQVLAADTDCQAVCASRANTELNQLDLD

RLRVVHGSVEALSAQLEGQTVDLLLCNILAPVIEVLAPSFDQLLSANGRGLLSGLLVKQAPRLQMVLEAL

GWRVNCLTEQGCWGLLDVSKR

>gi|48474357|sp|Q7V4K0.2|RIMM_PROMM RecName: Full=Ribosome maturation factor RimM

MNGDEDWLTVGKVVAAQGMQGELRINPSSDFPERFTLPGQRWLKERNGEPRPIELLTGRQLPGRSLYVVK

FAGVNNRNAAEALVGQKLLVPSSDRPSLAEGEFHLLDLVGLEARLQAEGPAIGHVIDLTTAGNDLLEIEL

LTGRRVLVPFVEAIVPEVQLNQGWLRLTPPPGLLEL

>gi|81577177|sp|Q7V606.1|PNP_PROMM RecName: Full=Polyribonucleotide nucleotidyltransferase; AltName: Full=Polynucleotide phosphorylase; Short=PNPase

MQGQTQSISFDGREIRLTTGRYAPQAGGSVMMECGDTAVLVTATRSTGREGIDFLPLICDYEERLYAAGR

IPGSFMRREGRPPERATLIARLIDRPMRPLFPSWMRDDLQIVATCLSLDERVPADVLAVTGASMATLLAS

IPFQGPMAAVRVGLLGDDFVLNPSYREIERGDLDLVVAGTPDGVVMVEAGANQLPQGDVIEAIDFGYEAV

CELIKAQQTILKDAGIKQVQPEPPTQDQDTKLSTYLEKNCSKSIGEVLKQFEQTKAERDSKLDAIKAKTA

EAIDSLKEDDAVRKSVNANSKVLSNNFKALTKKLMREQIIKQGKRVDGRKLDEVRPISSAAGVLPKRVHG

SGLFQRGLTQVLSTATLGTPSDAQEMDDLNPGPEKTYLHHYNFPPYSVGETRPMRSPGRREVGHGSLAER

AIIPVLPPKDSFPYVLRVVSEVLSSNGSTSMGSVCGSTLALMDAGVPLKAPVSGAAMGLIKEDAEIRILT

DIQGIEDFLGDMDFKVAGTKDGITALQMDMKITGLPVKTIAEAVNQARPARIHILEKMLEAIDAPRTTLS

PHAPRLLSFRIDPELIGTVIGPGGRTIKGITERTNTKIDIEDGGIVTIASHDGAAAEAAQRIIEGLTRKV

NEGEVFTGTITRIIPIGAFVEILPGKEGMIHISQLSEARVEKVDDVVKVGDQVTVRIREIDNRGRINLTL

RGVPQNGEETQSEPAPTPVAPLN

>gi|123200195|gb|ABM71803.1| 30S ribosomal protein S1 protein B, putative Nbp1 [Prochlorococcus marinus str. MIT 9515]

MKGVNDKGAQNDKKIKGQKNDVKKPLQVLHISKKDSEKEKEANFDDNQKLSNGITKDINATKPQIIEAPL

NEDKENHSANINLENKSYQELAKPVNFKDEDQEFIIERKVDEFDFDENAFLEALNENEPIGTTGETIKGK

IIALESDGLYVDIGGKAPGFMPKKECGLGVITNFKEKFPIGLEMEVLVIKEQNADGMVTISSRALILRQS

WEKVENSAKNGELIQVSINGFNRGGLTCDVDGLRGFIPRSQLEDGQDYQSLVNKTLKVAFLEVNPESRKL

VLSEKKALLVSKFSGLKLGQLIEGEVLGIKPYGFFVDLGGASGLLHQSSITNGSIRNLREIFEEGELIKA

LITEIDLERGRIGLNTALLENSPGELIVDKGKVMLEASERALKAKALFDKKNLQNDSQ

>gi|123199946|gb|ABM71554.1| 30S ribosomal protein S1, protein A [Prochlorococcus marinus str. MIT 9515]

MIENSSETIKEISDDKEIENSTVKENTSETPKIEDLSFESKDIPSADSSSSRRNSDLDSAGFTQEDFASL

LGKYDYNFKPGDLVNGTVFALEPKGAMIDIGAKTAAFMPMQEVSINRVEGLSDVLQPSESREFFIMSEEN

EDGQLALSIRRIEYQRAWERVRQLQKEDATIYSEVFATNRGGALVRVEGLRGFIPGSHISARKIKEDLEG

EYLPLKFLEVDEERNRLVLSHRRALVEKKMNRLEVGEVVVGSVKGIKPYGAFIDIGGVSGLLHISEISHE

HIETPHNVLNVTDQMKVMIIDLDSERGRISLSTKALEPEPGDMLTDPQKVFDKAEEMAAKYKQMLLEQTD

ENDEQTADIPESE

>gi|123201462|gb|ABM73070.1| 50S ribosomal protein L9 [Prochlorococcus marinus str. MIT 9515]

MAKRVKVVLTESIATLGRDGDVVEVAPGYARNYLLPFGKASNVTPSILKQIERKRAKEKIAAEKLKQEAI

DFKTALTTIGRFTIKKQVGEDGVLFGTVTNGDVAEAIQSATKKDIDRRDITVPDIHNLGSFVAKIKLHQE

VSAEVNIEVTS

>gi|123201325|gb|ABM72933.1| 50S ribosomal protein L6 [Prochlorococcus marinus str. MIT 9515]

MSRIGKSPVQIPDKVSVDINGLTITVKGPKGELKRLMPEGVDFVQKENQIVVTPSTTKRYSRERHGLCRT

LISNMVQGVTDGYSKKLEIVGVGSRAQVKGKTLVVSAGYSHPVEMTPPDGITYKVESNTNVTVSGIDKEI

VGNEAAKIRSIRPPEPYKGKGIKYQDERIIRKAGKSGKK

>gi|123201323|gb|ABM72931.1| 30S ribosomal protein S5 [Prochlorococcus marinus str. MIT 9515]

MTDTPTKNENQSKADNLPPSNPNDQRKGNRNNDRKRNRRGDSKNERDSEWQERVVQIRRVFKTVKGGKKM

SFRAIVVVGNEKGQVGVGVGKAGDVIGAVRKGVSDGKKHLVRVPLTPNNSIPTLSKGRDGAANVLIRPAA

PGTGVIAGGSIRTVLELAGIKNVLAKRLGSKTPLNNARAAMVALSQLRTHKSASRERGISLEQLYS

>gi|123199834|gb|ABM71442.1| 50S ribosomal protein L11 [Prochlorococcus marinus str. MIT 9515]

MAKKIVAVIKLALQAGKANPAPPVGPALGQHGVNIMAFCKEYNARTQDKAGFVIPVEISVFEDRSFTFIT

KTPPASVLITKAAGIEKGSGESSKGSVGNISKSQLEEIAKTKLPDLNCTSIESAMKVIEGTARNMGISIT

E

>gi|123199832|gb|ABM71440.1| 50S ribosomal protein L10 [Prochlorococcus marinus str. MIT 9515]

MGRTIENKQKIVTELKSLLDDSEMAVVLDYKGLTIKEMSDLRSRLQTNKGICKVTKNSLMRKAIDGNSNW

TDLESLLTGTNAFVLIKEDVGGAVKAIQSFQKETKKSETKGALFEGRLLSQSEIKEIASLPSREVLMAKI

AGALNGVATKIAISINEVPSGLARSLKQHSEKSES

>gi|123201334|gb|ABM72942.1| 50S ribosomal protein L22 [Prochlorococcus marinus str. MIT 9515]

MIKKSEMTKKAIAHGKYIRGSASKVRRVLDQIRGKSYRDALIMLEFMPYRSTDPITKVLRSAVANAEHNL

GMEPSSLVISSASADNGPVMKRFRPRAQGRAFSIKKQTCHISISVESAPNQTNTEAQN

>gi|123201333|gb|ABM72941.1| 30S ribosomal protein S3 [Prochlorococcus marinus str. MIT 9515]

MGNKINPTGLRLGITQEHRSKWFATSKTYPILLQEDYKIRNFIQKKYSSAGISDVLIARKADQLELELKT

ARPGVIVGRQGSGIEELRSGIQKTIGDRTRQVRINVVEVERVDADAYLLAEYIAQQLEKRVAFRRTIRMA

LQRAQRAGVLGLKIQVGGRLNGAEIARSEWTREGRVPLHTLRAEVDYALREANTTYGVLGIKVWVFKGEV

LPKEEQTIPVGAIPRRKGSRKPQQFEDRSSNENS

>gi|123201327|gb|ABM72935.1| 50S ribosomal protein L5 [Prochlorococcus marinus str. MIT 9515]

MTLKTRYKETIRPKLLKDLGLKNIHQVPKVIKVNVNRGLGEAASNSKALEASLNEMATITGQKALVTRSK

KAIAGFKIREGMAIGCTVTLRGDRMYSFLERFINLALPRIRDFRGVNPKSFDGRGNYTLGVKEQLIFPEI

SFDKIDSIRGMDITIVTSASNDQEGKALLKELGMPFSN

>gi|123200072|gb|ABM71680.1| 30S ribosomal protein S4 [Prochlorococcus marinus str. MIT 9515]

MSRYRGPRLRVTRRLGELPGLTRKASKKSNPPGQHGQARRKRSEYAIRLEEKQKLRFNYGVSERQLVRYV

KKARAQEGSTGTNLLRLLENRLDNVCFRLGFGGTIPGSRQLVNHGHVTVNGKVLDIAGYQCKSGDVISIK

EKKASKKLVEGNIEFPGLANVPPHIELDKPKLTGKINGKCDREWVALEINELLVVEYYSRKV

>gi|123199833|gb|ABM71441.1| 50S ribosomal protein L1 [Prochlorococcus marinus str. MIT 9515]

MKKLSKRMTALSTKIEDRTYAPLEALAIIKENANAKFDETIEAHIRLGIDPKYTDQQLRTTVALPHGTGQ

SIKIAVITSGENVAKAKSAGADLFGEEDLVESINKGNMDFDLLIATPDMMPKVAKLGRVLGPRGLMPNPK

AGTVTSDIASAIKEFKAGKLEFRADKAGIVHVRFGKASFTENALFENLKTLQESIDKNKPSGAKGKYWKT

FYVTSTMGPSVQVDINALQDYQPES

>gi|123199831|gb|ABM71439.1| 50S ribosomal protein L7/L12 [Prochlorococcus marinus str. MIT 9515]

MTAKTEEILESLKSLSLLEASELVKQIEEAFGVSAAASAGVVMAAPGAAGGDGDGGAAEEKTEFDVILES

FDAAAKIKVLKVVRNATGLGLGDAKALVESAPKTVKEGIAKADAETLKKEIEEAGGKVTLK

>gi|123201496|gb|ABM73104.1| 30S ribosomal protein S6 [Prochlorococcus marinus str. MIT 9515]

MTDQIYYETMYILRPDIAEEEVKNHIDKYNKLLEEFGATILDSQMRGKRRLAYQIAKHREGIYVQLSHQG

DGQHIFKIEKAMRLSEDVIRYLTVKQEGPLPTPRSSNKSSNQAEKKENENIDSANKSEPKADETDNKKKI

TLESSTPELEEQVKS

>gi|123201451|gb|ABM73059.1| 50S ribosomal protein L20 [Prochlorococcus marinus str. MIT 9515]

MARVKRGNIARKRRNKILNLAKGFRGGNKNLFRTANQRVMKALCNAYRDRRRRKRDFRRLWIARINASAR

INGTNYSKLINGLKTSEIIINRKMLAQLALNDPQSFEKIVSAVSK

>gi|123201450|gb|ABM73058.1| 50S ribosomal protein L35 [Prochlorococcus marinus str. MIT 9515]

MSKLKTRKSAAKRFKATATGKFMRRRAFHNHLLDHKSSKLKRHLSTKAVVDERDADNVRLMIPYA

>gi|123201339|gb|ABM72947.1| 50S ribosomal protein L3 [Prochlorococcus marinus str. MIT 9515]

MSIGILGKKLGMSQLFDDDGNAVPVTLIEAGPCRVTQLKTQSLDGYTAIQIGYGVSKDKHLSKPEKGHLL

KSGKELLKHLKEFKVEENSSYEIGKEITVTNFEVGQKVDISGKSMGRGFSGYQKRHGFSRGPMSHGSKNH

RAPGSTGAGTTPGRIYPGKRMAGRYGGKKITTKGLLVVKIDDQKNLLVVKGSVPGKPGSIVNIRPNNTVG

NKGGTKS

>gi|123201338|gb|ABM72946.1| 50S ribosomal protein L4 [Prochlorococcus marinus str. MIT 9515]

MTTLETLKWDGKKVGKVSIDLKVAKETSSSDLIHRAVLRQLANQRQGTASTLTRSEVRGGGRKPYKQKGT

GRARQGSIRTPLRPGGGVIFGPKPRSYNLDMNRKERRLALRTALMSRVDDIKAVEDFGSTLNQPKTSEII

NGLSRLGIEKTEKVLVILDSPSEVIKKSINNIAKVKLIAADQLNVFDILNANKLVIGQSAINKIQEVYAS

>gi|123201337|gb|ABM72945.1| 50S ribosomal protein L23 [Prochlorococcus marinus str. MIT 9515]

MSKLFESRLADVIRKPVITEKATNALDLNQYTFEVDHRAAKPQIKAAIEALFNVKVIGINTMNPPRRTRR

VGKFSGKRSQVKKAIVRLAEGDKIQLFPES

>gi|123201336|gb|ABM72944.1| 50S ribosomal protein L2 [Prochlorococcus marinus str. MIT 9515]

MAIRKFKPYTPGTRQRVVTDFSEITGSKPERSLIVSKHRNKGRNNRGVITCRHRGGGHKRQYRLVDFRRD

KRNINAKVAAIHYDPHRNARLALLFYEDGEKRYIIAPAGIKVGQNVISGDSVPIEDGNAMPLSVMPLGSS

VHCVELYAGRGAQMVRSAGASAQLMAKEGDYVALKLPSTEVRLVRKECYATLGEVGNAEIRNTSLGKAGR

TRWLGRRPQVRGSVMNPCDHPHGGGEGKAPIGRAGPVTPWGKAALGLKTRKKNKPSNNLVVRRRRRISKR

SRGGRDS

>gi|123201335|gb|ABM72943.1| 30S Ribosomal protein S19 [Prochlorococcus marinus str. MIT 9515]

MGRSLKKGPFIADSLLKKVEKQNTENDKSVIKTWSRSSTILPVMIGHTIAVHNGKAHIPVFITEQMIGHK

LGEFAPTRTYRGHLRDKKGAR

>gi|123201330|gb|ABM72938.1| 30S Ribosomal protein S17 [Prochlorococcus marinus str. MIT 9515]

MALKERIGTVVSDKMDKTVVVAVINRYPHPTYKKIVSKTTRYKAHDPENSCAMGDRVKIKETRPLSAHKR

WAIEEILNKTIKNKEDKK

>gi|123201329|gb|ABM72937.1| 50S Ribosomal protein L14 [Prochlorococcus marinus str. MIT 9515]

MIQQETYLTVADNSGAKRLQCIRVLGSNRRYAHVGDVVVASVKDALPNMGVKKSDVVKAVIVRTRHTLRR

NTGNSIRFDDNAAVLINEDKNPKGTRVFGPVARELRDKNFTKIVSLAPEVI

>gi|123201328|gb|ABM72936.1| 50S ribosomal protein L24 [Prochlorococcus marinus str. MIT 9515]

MLDSIKQKKNSKRIKMRIKTGDLVKVINGKEKGKTGEVLKTIPLENRVVVKGINLRTKHVKPTQEGESGR

ILTEEASLHASNVMFFSKDKNIVSKIEFFIDKEGVKKRKLKKTGELID

>gi|123201326|gb|ABM72934.1| 30S ribosomal protein S8 [Prochlorococcus marinus str. MIT 9515]

MSNHDPISDMLTRIRNASQKKHTSTSIPASRMILSIAKVLQKEGFIADINEEGEGYESKIVLGLKYSGKN

RFPTIRSMQRVSKPGLRVYKNTKGLPKVLGGLGVAIVSTSKGVMSDRDARKQGIGGEVLCYVY

>gi|123201324|gb|ABM72932.1| 50S ribosomal protein L18 [Prochlorococcus marinus str. MIT 9515]

MAKISRKLQTQKRHKRLRRYLIGNKIRPRLAVFRSNNHIYAQVIDDDAQQTICSASTVDKELKEDSDKLS

PNCSSSTIVGKLLAKRAIKKGIKQVIFDRGGNLYHGRVKALADAARDAGLNF

>gi|123201322|gb|ABM72930.1| 50S ribosomal protein L15 [Prochlorococcus marinus str. MIT 9515]

MTSTLNTLKSNLGSRKKKLRKGRGIAAGQGASCGFGMRGQKSRSGRPTRPGFEGGQMPLYRRVPKLKHFE

IINQKNYSIVNLSKLSEFKDNEIVNIDSLVKKKLLFKPKFPLKILGNGVVKVKLKVQAHAFTKVAQEKIE

SAGGSCEVLNNK

>gi|123201319|gb|ABM72927.1| 50S Ribosomal protein L36 [Prochlorococcus marinus str. MIT 9515]

MKVRASVKKMCDKCRVIRRHGRVMVICTASPRHKQRQG

>gi|123201318|gb|ABM72926.1| 30S ribosomal protein S13 [Prochlorococcus marinus str. MIT 9515]

MARIAGIDIPREKRVEIALTYIYGIGLTRSKLILSNTGVNPDIRVKDLSDSDVQKLRVAAEDFTVEGDLR

RQEGMALKRLQDIGCVRGRRHRMSLPVRGQRTRTNARTRRGSRKTVAGRKK

>gi|123201317|gb|ABM72925.1| 30S ribosomal protein S11 [Prochlorococcus marinus str. MIT 9515]

MAAPVKKTGSKKSKKNVPNGVVHIQSTFNNTIVSISDTSGHVISWSSAGASGFKGARKGTPFAAQTAAEA

AAKRALDQGMRQIEVLVRGPGSGRETAIRALQVAGLEITLIRDVTPLPHNGCRRPKRRRV

>gi|123201315|gb|ABM72923.1| 50S ribosomal protein L17 [Prochlorococcus marinus str. MIT 9515]

MRHQLRIPLLSKPADQRKALLRGLTTQLIREGRVTTTKARAKALRNETERMISLAKEGTLAARRRAIGYI

YDKKLVHSLFEKAQERYGDRNGGYTRIVRTVARKGDNAQMAIIELV

>gi|123201313|gb|ABM72921.1| 50S ribosomal protein L13 [Prochlorococcus marinus str. MIT 9515]

MNKTITPSIETIERNWFLVDAKDKTLGRLATEIASVLRGKNKPTFTPHLDTGDFVIVVNAEKVEVTGKKT

SQKLYRRHSGRPGGMKVEKFESLQERIPERIIEQAVKGMLPHNSLGRQQFKKLKVYKGSEHPHAAQNPVS

LNS

>gi|123201312|gb|ABM72920.1| 30S ribosomal protein S9 [Prochlorococcus marinus str. MIT 9515]

MNSQIKNKAVYWGTGRRKTSVARVRLIPGNGQIKINGRSGDDYLNFNPSHLNSVKAPLQTLGLENSYDIF

VNVFGGGLTGQADAIKQGAARALCDLSPDNRKPLKTEGHLSRDPRAKERRKYGLKKARKAPQFSKR

>gi|123201311|gb|ABM72919.1| 50S ribosomal protein L31 [Prochlorococcus marinus str. MIT 9515]

MPKSEIHPKWYPDAKVICNGEVVMTTGSTKPELHVDVWSGNHPFFTGTQKILDTEGRVDRFMKKYGMGSA

DSATSQETKEAKESDK

>gi|123201290|gb|ABM72898.1| 30S ribosomal protein S12 [Prochlorococcus marinus str. MIT 9515]

MPTISQLIGSERKRLTRKTKSPALKSCPERRGVCTRVYTSTPKKPNSALRKVARVRLTSGFEVTAYIPGI

GHNLQEHSVVLLRGGRVKDLPGVRYHIIRGTLDTAGVKDRRQSRSKYGAKAPKNN

>gi|123201289|gb|ABM72897.1| 30S ribosomal protein S7 [Prochlorococcus marinus str. MIT 9515]

MSRRNAAVKRPVLPDPQFNSRLASMMISRLMKHGKKSTAQKILSDAFSLISERTGGNAVELFETAVKNAT

PLVEVRARRVGGATYQVPMEVRQERGTAMALRWLVTFSRGRNGKSMSQKLAGELMDAANETGSAVKKRED

THKMAEANKAFAHYRY

>gi|123201286|gb|ABM72894.1| 30S ribosomal protein S10 [Prochlorococcus marinus str. MIT 9515]

MTTSLAQQKIRIRLKAFDRRMLDLSCDKIIQTADTTAASAIGPIPLPTKRKIYCVLRSPHVDKDSREHFE

TRTHRRIIDIYSPSAKTIDALMKLDLPSGVDIEVKL

>gi|123201266|gb|ABM72874.1| 30s Ribosomal protein S20 [Prochlorococcus marinus str. MIT 9515]

MANNKSAKKRIKVAERNRLVNKSYKSTVKTLTKKTLANCEKYKLDPNSDNKNLVMFSLSEAFSLIDKAVK

KNVLHKNNGANKKSKINKLVKNFLTSK

>gi|123201113|gb|ABM72721.1| putative methyltransferase for Ribosomal protein L11 [Prochlorococcus marinus str. MIT 9515]

MEINYWYKLTFEIEANLEEIIIWKLNELGISSYALEILLNNKNNKKVLIWLPNLNWPKSLRIKLERNIKE

VLDKNNYRTNCFEWIVIEQEDWMSSWKKYWGPELVGKKLLVLPCWLELPEKFKNKKVIKIDPGAAFGTGS

HPTTSLCLEELEKFSLSNKKILDIGSGSGILSIAARYFGASKVYSIDNDYLAINSTESNFRLNFGDLDDL

KTYLGRFDELVSKYSLKNFDLILCNILAEVIKGIIPDIRNCLKINGEVIFSGILNSQKDEIIKLLNASNL

QINDVSSKQGWVCITAQKII

>gi|123201105|gb|ABM72713.1| 50S ribosomal protein L27 [Prochlorococcus marinus str. MIT 9515]

MAHKKGTGSTRNGRDSNSKRLGVKAFGGEKVSAGSIIIRQRGTSFLPGINVGKGKDDTLFALKEGTVSFD

SIKRNLRNRKRVNIIL

>gi|123201104|gb|ABM72712.1| 50S ribosomal protein L21 [Prochlorococcus marinus str. MIT 9515]

MTSSKKSSDNSSSSNDLYAIAETSGQQFWFEVDRYYDIDRLNAKEKDKITIDKILLIKDKENVSIGKPYI

KNAKIELEVVSHKRDKKIIVYKMRPKKKTRRKMGHRQELTRVMVKSISISKSTPKSSPKTEATKKSTSSK

ASKPEN

>gi|123201045|gb|ABM72653.1| 30S Ribosomal protein S16 [Prochlorococcus marinus str. MIT 9515]

MIKLRLKRFGKKKEASFRIVACNSTSRRDGRPLQELGFYNPRTKETRLDTEALRIRLTQGAQPTDVVRTL

LEKGGLLEKKVRPSIAIGKANLEKEKIAKAKSKESESANKEAES

>gi|123200961|gb|ABM72569.1| 30S Ribosomal protein S14 [Prochlorococcus marinus str. MIT 9515]

MIAREVKRKKLVKKYAVKRKSLLDEFNAAKDPMERLEIHRKIQGLPRNSAPTRIRNRCWATGKPRGVYRD

FGLCRNQLRLRAHNGELPGVVKSSW

>gi|123200625|gb|ABM72233.1| 30S Ribosomal protein S15 [Prochlorococcus marinus str. MIT 9515]

MTLDTAEKQKLIESHQVHPTDTGSVEIQVAMISERITKLSEHLQGNIHDYASRQGLLKMIGKRKRLLSYI

KGKDPKNYQDLIKKIGIRG

>gi|123200583|gb|ABM72191.1| 50S ribosomal protein L28 [Prochlorococcus marinus str. MIT 9515]

MSRVCELTGARANNGMAVSHSHIRTKKLQQVNLQKRRLWWQEGKKWINIKISTKALKSIQKVGLDKVAKT

NGVDLNKF

>gi|123200550|gb|ABM72158.1| 50S Ribosomal protein L33 [Prochlorococcus marinus str. MIT 9515]

MQNSFLKLYNRKLYISVLIKMAKKGTRVVVTLECTEARTSSEPRRSNGISRYTTEKNKRNTTERLELKKF

NPHLNKMTIHKEIK

>gi|123200549|gb|ABM72157.1| 30S Ribosomal protein S18 [Prochlorococcus marinus str. MIT 9515]

MPNSIFKKQLSPIKPGDPIDYKDVELLKKFITERGKILPRRMTGLTSKQQRDLTLAVKRARIVALLPFVN

PEG

>gi|123200430|gb|ABM72038.1| 30S ribosomal protein S2 [Prochlorococcus marinus str. MIT 9515]

MAVVSLSEMMEAGAHFGHQTRRWNPKMSKYIYCARNGVHIIDLVKTALCMNNAYKWTRNAAKSGKRFLFV

GTKKQASDVVAQEAVRCGAAYVNQRWLGGMLTNWSTMKARIERLKDLERMESSGAIAMRPKKEAAVLRRE

LERLQKYLGGLKGMRRLPDVVVLVDQRRESNAVLEARKLDISLVSMLDTNCDPDLCEVPIPCNDDAVRSV

QLILGRLADAINEGRKGSNDQRKV

>gi|123200140|gb|ABM71748.1| Ribosomal protein L19 [Prochlorococcus marinus str. MIT 9515]

MAKEKQENESEIINQTDKSTVITVENEKKASISQTEKIHSSSNLIKEFESEQLKKQLPEIYVGDTVKVGV

KITEGNKERVQPYEGVVIAKRHGGLNQTITVRRIFQGIGVERVFMLHSPQVASLKVERRGKVRRAKLFYL

RDRVGKATRVKQRFDR

>gi|123201332|gb|ABM72940.1| 50S ribosomal protein L16 [Prochlorococcus marinus str. MIT 9515]

MLSPKRTKFRKQHRGRMKGIASKGNTIAFGQFALQAQDCGWVTARQIEASRRAMTRYVKRGGKIWIRIFP

DKPVTMRPAETRMGSGKGNPEFWVAVVKPGRILFEMGGEEITEEIAKEAMRLAQYKLPVKTKFISSDKIV

GGDSPVEKAIEKESTEEVKK

>gi|123201331|gb|ABM72939.1| 50S ribosomal protein L29 [Prochlorococcus marinus str. MIT 9515]

MKNSESIKEFKKLNSSEINEKIDQLRKDLFDLRFKQATRQLNETHQFKIIKKQVAQLLTLSKSQSNSQKS

SD

>gi|123200954|gb|ABM72562.1| 50S ribosomal protein L34 [Prochlorococcus marinus str. MIT 9515]

MTKRTFGGTSRKRKRVSGFRVRMRSHTGRRVIKSRRKRGRDRIAV

>gi|123200665|gb|ABM72273.1| 30S Ribosomal protein S21 [Prochlorococcus marinus str. MIT 9515]

MTQVTVGENEGIESALRRFKRQVSKSGIFADLKRLRHHETPIEKYKRKLQQRRKARRR

>gi|123200529|gb|ABM72137.1| 50S ribosomal protein L32 [Prochlorococcus marinus str. MIT 9515]

MAVPKKKKSKSKRNHRHAVWKGKAALAAQKAISLGKSVLTGKAQGFVYPIDEEEESEE

>gi|123201494|gb|ABM73102.1| Molecular chaperone DnaK2, heat shock protein hsp70-2 [Prochlorococcus marinus str. MIT 9515]

MGKVVGIDLGTTNSCVAVMEGGKPTVIANAEGFRTTPSVVAYTKNQDQLVGQIAKRQAVMNPENTFYSAK

RFVGRRVDEVNEESKDVSYGIEKAGSNVKLKCPVLDKQFSPEEVSAQVLRKLSEDAGKYLGENITQAVIT

VPAYFNDSQRQATKDAGKIAGLEVLRIINEPTAAALAYGLDKKSNERILVFDLGGGTFDVSVLEVGDGVF

EVLSTSGDTHLGGDDFDRCIVNHLASVFKSNEGIDLREDKQALQRLTEAAEKAKIELSNATQSEINLPFI

TATPDGPKHLDLNLTRANFEELASKLIDRCRVPVEQALKDAKLSTGEIDEIVMVGGSTRMPAVQELVKRV

TGKDPNQTVNPDEVVAVGAAIQGGVLAGEVKDILLLDVTPLSLGVETLGGVMTKMITRNTTVPTKKSETY

STAVDGQTNVEIHVLQGEREMASDNKSLGTFRLDGIPSAPRGVPQIEVTFDIDANGILSVTAKDKGSGKE

QSISITGASTLSDNEVDKMVKDAESNASVDKEKREKIDLKNQAETLVYQTEKQLGELGDKVDDSAKAKVE

EKSKALKEATSKEDYDSMKKLLEELQQELYAIGSSVYQQPGNQPPAPGGPNANASDDKGPDDDVIDADFT

ETKD

>gi|123201271|gb|ABM72879.1| N utilization substance protein A [Prochlorococcus marinus str. MIT 9515]

MALVILPGLNNLIEDISEEKKLPPHVVESALREALLKGYEKYRRTFYIGVKEDPFDEEYFSNFDVGFDLD

EEGYRILSSKIIVEEVESEDHQISLNEVKQVADDAQIGDTVVLDVTPEKEDFGRMAASTTKQVLAQKLRD

QQRKMIQEEFADLEDPVLTARVIRFERQSVIMGVSSGIGRPEVEAELPKRDQLPNDNYRANATFKVFLKE

VSEIARKGPQLFVSRANAGLVVYLFENEVPEIQEGTVKIVAVSREANPPSRAVGPRTKVAVDSIEREVDP

VGACIGARGARIQQVVNELRGEKIDVIKWSADPIEYILNSLSPAKVDLVRLVDPEGQHAHVLVPPDQLSL

AIGREGQNVRLAARLTGWKIDVKNSHEYNQEAEDAAVAELIVQREEEEHLQREAEQRLEAEQAERAAEDA

RLRELYPLPEDDEEYGSESYEEENLSENSQLENMNPDEIISTEEKAR

>gi|123200779|gb|ABM72387.1| putative ribosomal-protein-alanine acetyltransferase [Prochlorococcus marinus str. MIT 9515]

MISIKEIDHKEFELCFELDANTICLWTRRQWESEFNKKGVKVVGILIGNKIIGIYVVHTIIDEAQINYFS

IKKGFRRKGYGSYLMNYLLKQCEKLNIQKLLLEVSETNLIAEVFYSKFNFLTVGRRKNYYKDGSDAVLKE

KIFIK

>gi|123199619|gb|ABM71227.1| Predicted GTPase [Prochlorococcus marinus str. MIT 9515]

MKVEKKYKGLVIKKFNEFFLVESYQKYNSDSNKNFLCKIRKSVNFRNQLVFVGDEVILSQIDTTRKRAII

ESLVKRKNLLERPSVANISNIYVICSVQEPKLNLSQVNRFLISAEQMGVEVSLVLTKCDLITEQKRILLV

EKFKKWGYQAITLNLNSPKNFTNLMIELKKKKCSIFIGPSGVGKTTLLNMIIPGLNNTTASVSSKIKRGK

NTTRNVELFSLSSKSYIVDTPGFNMQTLEIDIRELPNLYPEIYRQVVIKGIHCKFRNCLHINDQGCNLNK

NFERYTFYKEMIESFKSHYCPIQAD

>gi|123200825|gb|ABM72433.1| Hypothetical protein P9515_12261 [Prochlorococcus marinus str. MIT 9515]

MKVRSSIKKIDQDDQIVKRRGRLYVINKKKPRNKQRQG

>gi|123199715|gb|ABM71323.1| possible Fe-S oxidoreductase [Prochlorococcus marinus str. MIT 9515]

MKQSNKNVKDKNITKVAFSHVGCEKNLVDTEHMQGLLDKEGYEVGNNLEDAKVVVVNTCSFIETAREESI

RKILEFTDQGKQVIVAGCMAQHFKEELLKEIPEIKALVGTGDYQKIAKVMNRVEKGEIVNEVSKVPEFIA

DEKIPRLIDQKKFVAYLRIAEGCDYNCAFCIIPKLRGPQRSRTIESIILEATNLANQGIQEIILISQITT

NYGQDIYGKPSLARLLKELSKVSVPWIRIHYAYPTGLTDEVIKAFKDSNNIVPYFDLPLQHSHSDVLKSM

NRPWQASLNESILSKIRDQIPSAVLRTSLIVGFPGEEQKHFLHLLDFLHKHKFDHVGVFIFSPEEGTSAF

DLPNRVPSEIADARKDNIMSIQQNISKKKNQLYVGTKVKVLVEKISKNNELIGRSYHFAPEIDGNVILSI

KKNVIENNYVGKFVEANICFADEYDLYGEVINIL

>gi|189045478|sp|A2BYS0.1|RS5_PROM5 RecName: Full=30S ribosomal protein S5

MTDTPTKNENQSKADNLPPSNPNDQRKGNRNNDRKRNRRGDSKNERDSEWQERVVQIRRVFKTVKGGKKM

SFRAIVVVGNEKGQVGVGVGKAGDVIGAVRKGVSDGKKHLVRVPLTPNNSIPTLSKGRDGAANVLIRPAA

PGTGVIAGGSIRTVLELAGIKNVLAKRLGSKTPLNNARAAMVALSQLRTHKSASRERGISLEQLYS

>gi|166230912|sp|A2BUI1.1|RL11_PROM5 RecName: Full=50S ribosomal protein L11

MAKKIVAVIKLALQAGKANPAPPVGPALGQHGVNIMAFCKEYNARTQDKAGFVIPVEISVFEDRSFTFIT

KTPPASVLITKAAGIEKGSGESSKGSVGNISKSQLEEIAKTKLPDLNCTSIESAMKVIEGTARNMGISIT

E

>gi|166223843|sp|A2BZ59.1|RL9_PROM5 RecName: Full=50S ribosomal protein L9

MAKRVKVVLTESIATLGRDGDVVEVAPGYARNYLLPFGKASNVTPSILKQIERKRAKEKIAAEKLKQEAI

DFKTALTTIGRFTIKKQVGEDGVLFGTVTNGDVAEAIQSATKKDIDRRDITVPDIHNLGSFVAKIKLHQE

VSAEVNIEVTS

>gi|166221279|sp|A2BYS2.1|RL6_PROM5 RecName: Full=50S ribosomal protein L6

MSRIGKSPVQIPDKVSVDINGLTITVKGPKGELKRLMPEGVDFVQKENQIVVTPSTTKRYSRERHGLCRT

LISNMVQGVTDGYSKKLEIVGVGSRAQVKGKTLVVSAGYSHPVEMTPPDGITYKVESNTNVTVSGIDKEI

VGNEAAKIRSIRPPEPYKGKGIKYQDERIIRKAGKSGKK

>gi|166230049|sp|A2BUH9.1|RL10_PROM5 RecName: Full=50S ribosomal protein L10

MGRTIENKQKIVTELKSLLDDSEMAVVLDYKGLTIKEMSDLRSRLQTNKGICKVTKNSLMRKAIDGNSNW

TDLESLLTGTNAFVLIKEDVGGAVKAIQSFQKETKKSETKGALFEGRLLSQSEIKEIASLPSREVLMAKI

AGALNGVATKIAISINEVPSGLARSLKQHSEKSES

>gi|166222194|sp|A2BUH8.1|RL7_PROM5 RecName: Full=50S ribosomal protein L7/L12

MTAKTEEILESLKSLSLLEASELVKQIEEAFGVSAAASAGVVMAAPGAAGGDGDGGAAEEKTEFDVILES

FDAAAKIKVLKVVRNATGLGLGDAKALVESAPKTVKEGIAKADAETLKKEIEEAGGKVTLK

>gi|166221982|sp|A2BYT1.1|RL22_PROM5 RecName: Full=50S ribosomal protein L22

MIKKSEMTKKAIAHGKYIRGSASKVRRVLDQIRGKSYRDALIMLEFMPYRSTDPITKVLRSAVANAEHNL

GMEPSSLVISSASADNGPVMKRFRPRAQGRAFSIKKQTCHISISVESAPNQTNTEAQN

>gi|166216366|sp|A2BYS4.1|RL5_PROM5 RecName: Full=50S ribosomal protein L5

MTLKTRYKETIRPKLLKDLGLKNIHQVPKVIKVNVNRGLGEAASNSKALEASLNEMATITGQKALVTRSK

KAIAGFKIREGMAIGCTVTLRGDRMYSFLERFINLALPRIRDFRGVNPKSFDGRGNYTLGVKEQLIFPEI

SFDKIDSIRGMDITIVTSASNDQEGKALLKELGMPFSN

>gi|160166295|sp|A2BUI0.1|RL1_PROM5 RecName: Full=50S ribosomal protein L1

MKKLSKRMTALSTKIEDRTYAPLEALAIIKENANAKFDETIEAHIRLGIDPKYTDQQLRTTVALPHGTGQ

SIKIAVITSGENVAKAKSAGADLFGEEDLVESINKGNMDFDLLIATPDMMPKVAKLGRVLGPRGLMPNPK

AGTVTSDIASAIKEFKAGKLEFRADKAGIVHVRFGKASFTENALFENLKTLQESIDKNKPSGAKGKYWKT

FYVTSTMGPSVQVDINALQDYQPES

>gi|152112247|sp|A2BV69.1|RS4_PROM5 RecName: Full=30S ribosomal protein S4

MSRYRGPRLRVTRRLGELPGLTRKASKKSNPPGQHGQARRKRSEYAIRLEEKQKLRFNYGVSERQLVRYV

KKARAQEGSTGTNLLRLLENRLDNVCFRLGFGGTIPGSRQLVNHGHVTVNGKVLDIAGYQCKSGDVISIK

EKKASKKLVEGNIEFPGLANVPPHIELDKPKLTGKINGKCDREWVALEINELLVVEYYSRKV

>gi|152060894|sp|A2BYT0.1|RS3_PROM5 RecName: Full=30S ribosomal protein S3

MGNKINPTGLRLGITQEHRSKWFATSKTYPILLQEDYKIRNFIQKKYSSAGISDVLIARKADQLELELKT

ARPGVIVGRQGSGIEELRSGIQKTIGDRTRQVRINVVEVERVDADAYLLAEYIAQQLEKRVAFRRTIRMA

LQRAQRAGVLGLKIQVGGRLNGAEIARSEWTREGRVPLHTLRAEVDYALREANTTYGVLGIKVWVFKGEV

LPKEEQTIPVGAIPRRKGSRKPQQFEDRSSNENS

>gi|166199808|sp|A2BXQ1.1|RL34_PROM5 RecName: Full=50S ribosomal protein L34

MTKRTFGGTSRKRKRVSGFRVRMRSHTGRRVIKSRRKRGRDRIAV

>gi|226708153|sp|A2BYQ8.1|RL31_PROM5 RecName: Full=50S ribosomal protein L31

MPKSEIHPKWYPDAKVICNGEVVMTTGSTKPELHVDVWSGNHPFFTGTQKILDTEGRVDRFMKKYGMGSA

DSATSQETKEAKESDK

>gi|205831016|sp|A2BXC2.1|RL361_PROM5 RecName: Full=50S ribosomal protein L36 1

MKVRSSIKKIDQDDQIVKRRGRLYVINKKKPRNKQRQG

>gi|166231012|sp|A2BYR0.1|RL13_PROM5 RecName: Full=50S ribosomal protein L13

MNKTITPSIETIERNWFLVDAKDKTLGRLATEIASVLRGKNKPTFTPHLDTGDFVIVVNAEKVEVTGKKT

SQKLYRRHSGRPGGMKVEKFESLQERIPERIIEQAVKGMLPHNSLGRQQFKKLKVYKGSEHPHAAQNPVS

LNS

>gi|218547107|sp|A2BYS5.1|RL24_PROM5 RecName: Full=50S ribosomal protein L24

MLDSIKQKKNSKRIKMRIKTGDLVKVINGKEKGKTGEVLKTIPLENRVVVKGINLRTKHVKPTQEGESGR

ILTEEASLHASNVMFFSKDKNIVSKIEFFIDKEGVKKRKLKKTGELID

>gi|205831046|sp|A2BYR6.1|RL362_PROM5 RecName: Full=50S ribosomal protein L36 2

MKVRASVKKMCDKCRVIRRHGRVMVICTASPRHKQRQG

>gi|166987299|sp|A2BYT4.1|RL23_PROM5 RecName: Full=50S ribosomal protein L23

MSKLFESRLADVIRKPVITEKATNALDLNQYTFEVDHRAAKPQIKAAIEALFNVKVIGINTMNPPRRTRR

VGKFSGKRSQVKKAIVRLAEGDKIQLFPES

>gi|166986916|sp|A2BY51.1|RL21_PROM5 RecName: Full=50S ribosomal protein L21

MTSSKKSSDNSSSSNDLYAIAETSGQQFWFEVDRYYDIDRLNAKEKDKITIDKILLIKDKENVSIGKPYI

KNAKIELEVVSHKRDKKIIVYKMRPKKKTRRKMGHRQELTRVMVKSISISKSTPKSSPKTEATKKSTSSK

ASKPEN

>gi|166234499|sp|A2BYR9.1|RL15_PROM5 RecName: Full=50S ribosomal protein L15

MTSTLNTLKSNLGSRKKKLRKGRGIAAGQGASCGFGMRGQKSRSGRPTRPGFEGGQMPLYRRVPKLKHFE

IINQKNYSIVNLSKLSEFKDNEIVNIDSLVKKKLLFKPKFPLKILGNGVVKVKLKVQAHAFTKVAQEKIE

SAGGSCEVLNNK

>gi|166234344|sp|A2BWS2.1|RS15_PROM5 RecName: Full=30S ribosomal protein S15

MTLDTAEKQKLIESHQVHPTDTGSVEIQVAMISERITKLSEHLQGNIHDYASRQGLLKMIGKRKRLLSYI

KGKDPKNYQDLIKKIGIRG

>gi|166233178|sp|A2BYT6.1|RL3_PROM5 RecName: Full=50S ribosomal protein L3

MSIGILGKKLGMSQLFDDDGNAVPVTLIEAGPCRVTQLKTQSLDGYTAIQIGYGVSKDKHLSKPEKGHLL

KSGKELLKHLKEFKVEENSSYEIGKEITVTNFEVGQKVDISGKSMGRGFSGYQKRHGFSRGPMSHGSKNH

RAPGSTGAGTTPGRIYPGKRMAGRYGGKKITTKGLLVVKIDDQKNLLVVKGSVPGKPGSIVNIRPNNTVG

NKGGTKS

>gi|166231311|sp|A2BYN3.1|RS10_PROM5 RecName: Full=30S ribosomal protein S10

MTTSLAQQKIRIRLKAFDRRMLDLSCDKIIQTADTTAASAIGPIPLPTKRKIYCVLRSPHVDKDSREHFE

TRTHRRIIDIYSPSAKTIDALMKLDLPSGVDIEVKL

>gi|166229587|sp|A2BYQ9.1|RS9_PROM5 RecName: Full=30S ribosomal protein S9

MNSQIKNKAVYWGTGRRKTSVARVRLIPGNGQIKINGRSGDDYLNFNPSHLNSVKAPLQTLGLENSYDIF

VNVFGGGLTGQADAIKQGAARALCDLSPDNRKPLKTEGHLSRDPRAKERRKYGLKKARKAPQFSKR

>gi|166227365|sp|A2BZ93.1|RS6_PROM5 RecName: Full=30S ribosomal protein S6

MTDQIYYETMYILRPDIAEEEVKNHIDKYNKLLEEFGATILDSQMRGKRRLAYQIAKHREGIYVQLSHQG

DGQHIFKIEKAMRLSEDVIRYLTVKQEGPLPTPRSSNKSSNQAEKKENENIDSANKSEPKADETDNKKKI

TLESSTPELEEQVKS

>gi|166226601|sp|A2BWN0.1|RL28_PROM5 RecName: Full=50S ribosomal protein L28

MSRVCELTGARANNGMAVSHSHIRTKKLQQVNLQKRRLWWQEGKKWINIKISTKALKSIQKVGLDKVAKT

NGVDLNKF

>gi|166225296|sp|A2BW77.1|RS2_PROM5 RecName: Full=30S ribosomal protein S2

MAVVSLSEMMEAGAHFGHQTRRWNPKMSKYIYCARNGVHIIDLVKTALCMNNAYKWTRNAAKSGKRFLFV

GTKKQASDVVAQEAVRCGAAYVNQRWLGGMLTNWSTMKARIERLKDLERMESSGAIAMRPKKEAAVLRRE

LERLQKYLGGLKGMRRLPDVVVLVDQRRESNAVLEARKLDISLVSMLDTNCDPDLCEVPIPCNDDAVRSV

QLILGRLADAINEGRKGSNDQRKV

>gi|166225032|sp|A2BY52.1|RL27_PROM5 RecName: Full=50S ribosomal protein L27

MAHKKGTGSTRNGRDSNSKRLGVKAFGGEKVSAGSIIIRQRGTSFLPGINVGKGKDDTLFALKEGTVSFD

SIKRNLRNRKRVNIIL

>gi|166224053|sp|A2BWW2.1|RS21_PROM5 RecName: Full=30S ribosomal protein S21

MTQVTVGENEGIESALRRFKRQVSKSGIFADLKRLRHHETPIEKYKRKLQQRRKARRR

>gi|166223968|sp|A2BYL3.1|RS20_PROM5 RecName: Full=30S ribosomal protein S20

MANNKSAKKRIKVAERNRLVNKSYKSTVKTLTKKTLANCEKYKLDPNSDNKNLVMFSLSEAFSLIDKAVK

KNVLHKNNGANKKSKINKLVKNFLTSK

>gi|166219721|sp|A2BYS7.1|RS17_PROM5 RecName: Full=30S ribosomal protein S17

MALKERIGTVVSDKMDKTVVVAVINRYPHPTYKKIVSKTTRYKAHDPENSCAMGDRVKIKETRPLSAHKR

WAIEEILNKTIKNKEDKK

>gi|166219679|sp|A2BZ48.1|RL20_PROM5 RecName: Full=50S ribosomal protein L20

MARVKRGNIARKRRNKILNLAKGFRGGNKNLFRTANQRVMKALCNAYRDRRRRKRDFRRLWIARINASAR

INGTNYSKLINGLKTSEIIINRKMLAQLALNDPQSFEKIVSAVSK

>gi|166218719|sp|A2BYS1.1|RL18_PROM5 RecName: Full=50S ribosomal protein L18

MAKISRKLQTQKRHKRLRRYLIGNKIRPRLAVFRSNNHIYAQVIDDDAQQTICSASTVDKELKEDSDKLS

PNCSSSTIVGKLLAKRAIKKGIKQVIFDRGGNLYHGRVKALADAARDAGLNF

>gi|166216203|sp|A2BYR2.1|RL17_PROM5 RecName: Full=50S ribosomal protein L17

MRHQLRIPLLSKPADQRKALLRGLTTQLIREGRVTTTKARAKALRNETERMISLAKEGTLAARRRAIGYI

YDKKLVHSLFEKAQERYGDRNGGYTRIVRTVARKGDNAQMAIIELV

>gi|166201334|sp|A2BXZ2.1|RS16_PROM5 RecName: Full=30S ribosomal protein S16

MIKLRLKRFGKKKEASFRIVACNSTSRRDGRPLQELGFYNPRTKETRLDTEALRIRLTQGAQPTDVVRTL

LEKGGLLEKKVRPSIAIGKANLEKEKIAKAKSKESESANKEAES

>gi|166199903|sp|A2BYT2.1|RS19_PROM5 RecName: Full=30S ribosomal protein S19

MGRSLKKGPFIADSLLKKVEKQNTENDKSVIKTWSRSSTILPVMIGHTIAVHNGKAHIPVFITEQMIGHK

LGEFAPTRTYRGHLRDKKGAR

>gi|166199843|sp|A2BYT5.1|RL4_PROM5 RecName: Full=50S ribosomal protein L4

MTTLETLKWDGKKVGKVSIDLKVAKETSSSDLIHRAVLRQLANQRQGTASTLTRSEVRGGGRKPYKQKGT

GRARQGSIRTPLRPGGGVIFGPKPRSYNLDMNRKERRLALRTALMSRVDDIKAVEDFGSTLNQPKTSEII

NGLSRLGIEKTEKVLVILDSPSEVIKKSINNIAKVKLIAADQLNVFDILNANKLVIGQSAINKIQEVYAS

>gi|166199819|sp|A2BZ47.1|RL35_PROM5 RecName: Full=50S ribosomal protein L35

MSKLKTRKSAAKRFKATATGKFMRRRAFHNHLLDHKSSKLKRHLSTKAVVDERDADNVRLMIPYA

>gi|160358607|sp|A2BYT3.1|RL2_PROM5 RecName: Full=50S ribosomal protein L2

MAIRKFKPYTPGTRQRVVTDFSEITGSKPERSLIVSKHRNKGRNNRGVITCRHRGGGHKRQYRLVDFRRD

KRNINAKVAAIHYDPHRNARLALLFYEDGEKRYIIAPAGIKVGQNVISGDSVPIEDGNAMPLSVMPLGSS

VHCVELYAGRGAQMVRSAGASAQLMAKEGDYVALKLPSTEVRLVRKECYATLGEVGNAEIRNTSLGKAGR

TRWLGRRPQVRGSVMNPCDHPHGGGEGKAPIGRAGPVTPWGKAALGLKTRKKNKPSNNLVVRRRRRISKR

SRGGRDS

>gi|158706262|sp|A2BYR5.1|RS13_PROM5 RecName: Full=30S ribosomal protein S13

MARIAGIDIPREKRVEIALTYIYGIGLTRSKLILSNTGVNPDIRVKDLSDSDVQKLRVAAEDFTVEGDLR

RQEGMALKRLQDIGCVRGRRHRMSLPVRGQRTRTNARTRRGSRKTVAGRKK

>gi|156637363|sp|A2BYN7.1|RS12_PROM5 RecName: Full=30S ribosomal protein S12

MPTISQLIGSERKRLTRKTKSPALKSCPERRGVCTRVYTSTPKKPNSALRKVARVRLTSGFEVTAYIPGI

GHNLQEHSVVLLRGGRVKDLPGVRYHIIRGTLDTAGVKDRRQSRSKYGAKAPKNN

>gi|156630853|sp|A2BWH6.1|RL32_PROM5 RecName: Full=50S ribosomal protein L32

MAVPKKKKSKSKRNHRHAVWKGKAALAAQKAISLGKSVLTGKAQGFVYPIDEEEESEE

>gi|152060808|sp|A2BYR4.1|RS11_PROM5 RecName: Full=30S ribosomal protein S11

MAAPVKKTGSKKSKKNVPNGVVHIQSTFNNTIVSISDTSGHVISWSSAGASGFKGARKGTPFAAQTAAEA

AAKRALDQGMRQIEVLVRGPGSGRETAIRALQVAGLEITLIRDVTPLPHNGCRRPKRRRV

>gi|148841162|sp|A2BYS3.1|RS8_PROM5 RecName: Full=30S ribosomal protein S8

MSNHDPISDMLTRIRNASQKKHTSTSIPASRMILSIAKVLQKEGFIADINEEGEGYESKIVLGLKYSGKN

RFPTIRSMQRVSKPGLRVYKNTKGLPKVLGGLGVAIVSTSKGVMSDRDARKQGIGGEVLCYVY

>gi|218551748|sp|A2BWJ7.2|RL33_PROM5 RecName: Full=50S ribosomal protein L33

MAKKGTRVVVTLECTEARTSSEPRRSNGISRYTTEKNKRNTTERLELKKFNPHLNKMTIHKEIK

>gi|166228461|sp|A2BYN6.1|RS7_PROM5 RecName: Full=30S ribosomal protein S7

MSRRNAAVKRPVLPDPQFNSRLASMMISRLMKHGKKSTAQKILSDAFSLISERTGGNAVELFETAVKNAT

PLVEVRARRVGGATYQVPMEVRQERGTAMALRWLVTFSRGRNGKSMSQKLAGELMDAANETGSAVKKRED

THKMAEANKAFAHYRY

>gi|166220973|sp|A2BWJ6.1|RS18_PROM5 RecName: Full=30S ribosomal protein S18

MPNSIFKKQLSPIKPGDPIDYKDVELLKKFITERGKILPRRMTGLTSKQQRDLTLAVKRARIVALLPFVN

PEG

>gi|166232684|sp|A2BYS6.1|RL14_PROM5 RecName: Full=50S ribosomal protein L14

MIQQETYLTVADNSGAKRLQCIRVLGSNRRYAHVGDVVVASVKDALPNMGVKKSDVVKAVIVRTRHTLRR

NTGNSIRFDDNAAVLINEDKNPKGTRVFGPVARELRDKNFTKIVSLAPEVI

>gi|166228242|sp|A2BYS8.1|RL29_PROM5 RecName: Full=50S ribosomal protein L29

MKNSESIKEFKKLNSSEINEKIDQLRKDLFDLRFKQATRQLNETHQFKIIKKQVAQLLTLSKSQSNSQKS

SD

>gi|166199704|sp|A2BYS9.1|RL16_PROM5 RecName: Full=50S ribosomal protein L16

MLSPKRTKFRKQHRGRMKGIASKGNTIAFGQFALQAQDCGWVTARQIEASRRAMTRYVKRGGKIWIRIFP

DKPVTMRPAETRMGSGKGNPEFWVAVVKPGRILFEMGGEEITEEIAKEAMRLAQYKLPVKTKFISSDKIV

GGDSPVEKAIEKESTEEVKK

>gi|238066582|sp|A2BU62.1|RIMO_PROM5 RecName: Full=Ribosomal protein S12 methylthiotransferase RimO; Short=S12 MTTase; Short=S12 methylthiotransferase; AltName: Full=Ribosomal protein S12 (aspartate-C(3))-methylthiotransferase; AltName: Full=Ribosome maturation factor RimO

MKQSNKNVKDKNITKVAFSHVGCEKNLVDTEHMQGLLDKEGYEVGNNLEDAKVVVVNTCSFIETAREESI

RKILEFTDQGKQVIVAGCMAQHFKEELLKEIPEIKALVGTGDYQKIAKVMNRVEKGEIVNEVSKVPEFIA

DEKIPRLIDQKKFVAYLRIAEGCDYNCAFCIIPKLRGPQRSRTIESIILEATNLANQGIQEIILISQITT

NYGQDIYGKPSLARLLKELSKVSVPWIRIHYAYPTGLTDEVIKAFKDSNNIVPYFDLPLQHSHSDVLKSM

NRPWQASLNESILSKIRDQIPSAVLRTSLIVGFPGEEQKHFLHLLDFLHKHKFDHVGVFIFSPEEGTSAF

DLPNRVPSEIADARKDNIMSIQQNISKKKNQLYVGTKVKVLVEKISKNNELIGRSYHFAPEIDGNVILSI

KKNVIENNYVGKFVEANICFADEYDLYGEVINIL

>gi|166223429|sp|A2BY60.1|PRMA_PROM5 RecName: Full=Ribosomal protein L11 methyltransferase; Short=L11 Mtase

MEINYWYKLTFEIEANLEEIIIWKLNELGISSYALEILLNNKNNKKVLIWLPNLNWPKSLRIKLERNIKE

VLDKNNYRTNCFEWIVIEQEDWMSSWKKYWGPELVGKKLLVLPCWLELPEKFKNKKVIKIDPGAAFGTGS

HPTTSLCLEELEKFSLSNKKILDIGSGSGILSIAARYFGASKVYSIDNDYLAINSTESNFRLNFGDLDDL

KTYLGRFDELVSKYSLKNFDLILCNILAEVIKGIIPDIRNCLKINGEVIFSGILNSQKDEIIKLLNASNL

QINDVSSKQGWVCITAQKII

>gi|166918240|sp|A2BZ91.1|DNAK_PROM5 RecName: Full=Chaperone protein DnaK; AltName: Full=HSP70; AltName: Full=Heat shock 70 kDa protein; AltName: Full=Heat shock protein 70

MGKVVGIDLGTTNSCVAVMEGGKPTVIANAEGFRTTPSVVAYTKNQDQLVGQIAKRQAVMNPENTFYSAK

RFVGRRVDEVNEESKDVSYGIEKAGSNVKLKCPVLDKQFSPEEVSAQVLRKLSEDAGKYLGENITQAVIT

VPAYFNDSQRQATKDAGKIAGLEVLRIINEPTAAALAYGLDKKSNERILVFDLGGGTFDVSVLEVGDGVF

EVLSTSGDTHLGGDDFDRCIVNHLASVFKSNEGIDLREDKQALQRLTEAAEKAKIELSNATQSEINLPFI

TATPDGPKHLDLNLTRANFEELASKLIDRCRVPVEQALKDAKLSTGEIDEIVMVGGSTRMPAVQELVKRV

TGKDPNQTVNPDEVVAVGAAIQGGVLAGEVKDILLLDVTPLSLGVETLGGVMTKMITRNTTVPTKKSETY

STAVDGQTNVEIHVLQGEREMASDNKSLGTFRLDGIPSAPRGVPQIEVTFDIDANGILSVTAKDKGSGKE

QSISITGASTLSDNEVDKMVKDAESNASVDKEKREKIDLKNQAETLVYQTEKQLGELGDKVDDSAKAKVE

EKSKALKEATSKEDYDSMKKLLEELQQELYAIGSSVYQQPGNQPPAPGGPNANASDDKGPDDDVIDADFT

ETKD

>gi|166226464|sp|A2BYY9.1|RIMM_PROM5 RecName: Full=Ribosome maturation factor RimM

MINHDEWLIVGLITSPQGINGKIKVKSLSDFEERFTKPGIRWIQKETESPKKLQLISGFQKPGKESFVIT

FKEIKNRNEAEKLKGYKLLVKVDEIPKLNKNEFHLTELMNLKVKILENNKLKIIGKVVNLENEKNNLLVI

KLLTNDKEVLVPFVKEIIPEIDIKNKFLIITPPPGLLEL

>gi|123960183|gb|ABM74966.1| 30S ribosomal protein S1, protein A [Prochlorococcus marinus str. NATL1A]

MSENPASKIEEKNPEKETSIPEETVSNSTSAEFEENSISELKEDDIPKNIPAADDSSSRINKSDLESAGF

TLDEFASLLSKYDYNFKPGDIVNGTVFALESKGAMIDIGAKTAAFMPMQEVSINRVEGLSDVLQPSEIRE

FFIMTEENEDGQLSLSIRRIEYQRAWERVRQLQKEDATIYSEVFATNRGGALVRVEGLRGFIPGSHISTR

KAKEELVADFLPLKFLEVDEERNRLVLSHRRALVERKMNRLEVGEVVVGAVRGIKPYGAFIDIGGVSGLL

HISEISHEHIETPHSVLNVNDQMKVMIIDLDAERGRISLSTKALEPEPGDMLTDPQKVFDKAEEMAARYK

QMLLEQAEEGEDPIAVMTI

>gi|123960365|gb|ABM75148.1| 30S ribosomal protein S1 protein B, putative Nbp1 [Prochlorococcus marinus str. NATL1A]

MLKSASLKNDRNLLIIKKQLTLDCKNVSLITKGIMAGSDPQPKKATPPRPAINAPRKPLQVMHISRKPEE

EIINEGSSEKEFSEQPKDRRFDEKVLAPKEISYLKKAPEQQKSIGQDSSDDFQSATMEDLLRSENNTNYK

NNIEAFDDQNIFEQKSRTVDEFDFDEDEFLAALEENQPIGTTGEIAKGSVIAVESDGIYVDIGGKAPGFM

PKNECGLGVITNLKERFPKGLKVEVLVTREQNADGMVTISCRALELRKSWDKVQNLAKEGKVIRVKINGF

NRGGVTCDFEGLRGFIPRSQLEDGENHQSLVSKTISTAFLEVNPERRKLVLSEKKAAIASRFSELEIGQL

IEGEILTIKPYGFFVDLRGVSGLLHHSMVTNGSMRSLREVFQPGESIKALITDLDPSRGRIGLNTALLEG

PPGELITDKTKVMEEANERAIKARNSLNKEKVDPQKEKEDINLSS

>gi|123961916|gb|ABM76699.1| 50S ribosomal protein L9 [Prochlorococcus marinus str. NATL1A]

MAKRVQVVLNEDIKSLGNDGDLVEVAPGFARNFLLPNKKALPVTPTVLKQVEHRRAKQAEKEAAKKQEAI

DFQTALTTIGRFTVKKQVGEDGVLFGTVTNGDVAEVVKEATKKDIDRRDISVPEIHGVGKYKVQIKLHNE

VNAEINLEVTSY

>gi|123961762|gb|ABM76545.1| 50S ribosomal protein L6 [Prochlorococcus marinus str. NATL1A]

MSRTGKKPISLPEKVDVKFEGLSITVKGPKGELQRTLPNGVSLSKDENFIFVKPINEKRQSREMHGLCRS

LVANMVEGVSNGFTKKLEIVGVGSRAQVKGKTLVVSAGYSHPVEVVPPEGITFKVENNTNVIVTGPDKEL

VGNEAAKIRAIRPPEPYKGKGIKYQGELIIRKAGKSGKT

>gi|123961760|gb|ABM76543.1| 30S ribosomal protein S5 [Prochlorococcus marinus str. NATL1A]

MTDSNNQSPNKKTSGSSGAPTAADGRQENRRSRGEKRGGRRDRRGQERDSEWQERVVQIRRVSKTVKGGK

KMSFRAIVVVGNEKGQVGVGVGKAGDVIGAVRKGVADGKKHLVRVPLTRNSSIPTLSNGRDGAASVLIRP

AAPGTGVIAGGSIRTVLELAGIKNVLAKRLGSKTPLNNARAAMVALSELRTHKATAKERGISLEQIYS

>gi|123960061|gb|ABM74844.1| 50S ribosomal protein L11 [Prochlorococcus marinus str. NATL1A]

MAKKVVALIKLALQAGKANPAPPVGPALGQHGVNIMAFCKEYNSRTQDKAGFVIPVEISVFEDRSFSFIT

KTPPASVLITKAAGIAKGSGESAKGSAGSISTSQLEEIAKTKLPDLNCSSIESAMKVIEGTAKNMGVSIK

D

>gi|123960059|gb|ABM74842.1| 50S ribosomal protein L10 [Prochlorococcus marinus str. NATL1A]

MGRTLESKKQIVKKIEDLLDNSEMALVLDYKGLSTKEMSDLRSRLQQSDGVCKVTKNTLMRQAIKGKNSW

TGLDSLLTGTNAFVLIKGDVGSAVKAVQAFQKETQKSETKGGLFEGKLLSQDEIKAIAKLPSKEALMGQI

AGALNSITSKIAIGINEVPSGLARSLKQHSESGES

>gi|123961771|gb|ABM76554.1| 50S ribosomal protein L22 [Prochlorococcus marinus str. NATL1A]

MTTSSTKTTAQAHGRYIRGSASKVRRVLDQIRGRTYRDALIMLEFMPYRSTEPITKVLRSAVANAENNLG

LDPSSLMISTATADMGPPMKRYRPRAQGRAFAIKKQTCHISISVSATQSTNSEDSD

>gi|123961770|gb|ABM76553.1| 30S ribosomal protein S3 [Prochlorococcus marinus str. NATL1A]

MGHKIHPTGIRLGITQEHRSKWYAPSKTYPLLLQEDDRIRTFIKKKYVAAGISDVLIARKADQLEVELKT

ARPGVIVGRQGSGIEELRSGIQKTIGDRNRQVRINVVEVEKVDADAYLLAEYIAQQLEKRVAFRRTIRMA

VQRAQRAGVLGLKIQVGGRLNGAEIARSEWTREGRVPLHTLRAEIDYATKVASTTYGVLGIKVWVFKGEV

LPKEEQPLPVGSSPRRSRGNRRPQQFEDRSNESK

>gi|123961764|gb|ABM76547.1| 50S ribosomal protein L5 [Prochlorococcus marinus str. NATL1A]

MSLKTRYRETIRPKLLKDLGLKNVHQVPKVQKVTLNRGLGEAATNSKALEASLKEMATISGQKALVTRAK

KAIATFKIRQGMPIGCSVTLRGDRMYAFLERFINLALPRIRDFRGVSPKSFDGRGNYTIGVKEQLIFPEI

TFDKVDTIRGMDITIVTSASSDEQGKALLSEMGMPFRKK

>gi|123960242|gb|ABM75025.1| 30S ribosomal protein S4 [Prochlorococcus marinus str. NATL1A]

MSRYRGPRLRITRRLGDLPGLTRKAAKRSHPPGQHGQARRKRSEYAIRLEEKQKLRFNYGISERQLVRYV

KKARAQEGSTGTNLLKLLENRLDNVCFRLGFGPTIPGSRQLVNHGHVTVNGKITDIASYQCKAGDVIAIR

DNKASKQLAQANLEFPGLANVPPHLELDKTKLSAKITAKTDREWVAIEINELLVVEYYSRKV

>gi|123960060|gb|ABM74843.1| 50S ribosomal protein L1 [Prochlorococcus marinus str. NATL1A]

MKNFSKRMTTLLSKVEERSYSPIEAIKLVKENANAKFDETIEAHIRLGIDPKYTDQQLRTTVALPSGTGQ

KIRIAVVTRGEKVNEATKAGADLAGEEDLVDSINKGEMNFDLLISTPDMMPKVAKLGRVLGPRGLMPNPK

AGTVTTDLEGAIKEFKAGKLEFRADKAGIVHVRFGKASFSEEALLENLKTLQTTIEKNKPSGAKGKFWRS

FFITSTMGPSVEVDINELQDLQKEK

>gi|123960058|gb|ABM74841.1| 50S ribosomal protein L7/L12 [Prochlorococcus marinus str. NATL1A]

MSAKTDEILDSLKSLSLLEASELVKQIEEAFGVSAAASAGVVMAAPGAAAGGDGADAAEEKTEFEVVLES

FDASSKIKVLKEVRNATGLGLGEAKALVEAAPKTIKEGATKEDAEALKKAIEAVGGKVTLK

>gi|123961962|gb|ABM76745.1| 30S ribosomal protein S6 [Prochlorococcus marinus str. NATL1A]

MSETPYYETMYILRPDIPEEEVDSHLKKYSAILEKSETEVLDSQMRGKRRLAYPIAKHKEGIYVQLSHKG

NGQQVATLERAMRLSEDVIRYITVKQDGPLPTPKPTSKEDETEKEEVKPTEDKTESPAQEEKKEDSKE

>gi|123961904|gb|ABM76687.1| 50S ribosomal protein L20 [Prochlorococcus marinus str. NATL1A]

MARVKRGNVARKRRNKILRLARGFRGGNGTLFRTANQRVMKALCNAYRDRRRRKRDFRRLWIARINAAAR

LNGLSYSKFMGGLKKADIRINRKMLAQLAVIDPKTFTNVAVNSKS

>gi|123961903|gb|ABM76686.1| 50S ribosomal protein L35 [Prochlorococcus marinus str. NATL1A]

MPKLKTRKAAAKRFKATGTGKFMRRRAFHNHLLDHKSPKLKRHLKTKAVVDERDAENVRLMLPYA

>gi|123961776|gb|ABM76559.1| 50S ribosomal protein L3 [Prochlorococcus marinus str. NATL1A]

MSLGILGKKLGMSQLFDDQGRAVPVTLIEAGPCRITQLKSADTDGYAAVQIGFQLIREKLINKPSKGHLA

KSGNDLLRHLREYRVENSSEFELGASITVDDFEKGQKVDVSGDTMGRGFAGYQKRHGFSRGPMSHGSKNH

RLPGSIGAGTTPGRVYPGKRMAGRMGGKKVTTRALEILKIDTNHNLLVVKGSVPGKPGSLLNIRPAKRVG

VSIQQGGE

>gi|123961775|gb|ABM76558.1| 50S ribosomal protein L4 [Prochlorococcus marinus str. NATL1A]

MMTNCTVLDWQGKEAGESSIDLKTAKESSAADLLHRAVLRQQAHSRQGTASTLTRSEVRGGGRKPYKQKG

TGRARQGSIRTPLRPGGGIIFGPKPRKYNLEMNRKERRLALRTALMSRISDAKIIKDFGSKLEVPKTSEI

VALLKRVGIDSDVKILIILNKPSEIIKRSIKNLEKVKLISADQLNVFDLLNANSLVIGEDALSTIKEVYG

ND

>gi|123961774|gb|ABM76557.1| 50S ribosomal protein L23 [Prochlorococcus marinus str. NATL1A]

MTKFFEKRLTDVIKRPLITEKATKALDLNQYTFEVDPRAAKPDIKAAVEKMFDVKVLGISTMNPPRKSRR

VGRFSGKRPQVKKAVVRLAEGNSIQLFPESEEA

>gi|123961773|gb|ABM76556.1| 50S ribosomal protein L2 [Prochlorococcus marinus str. NATL1A]

MAIRSFKPYTPGTRSRVVTDFSEVTSSKPERSLVVSKHRKKGRNNRGVITCRHRGGGHKRLYRIVDFRRN

KHGVSAKVAAIHYDPHRNARLALLFYSDGEKRYILAPADITVGQEVISGPDAPIETGNALPLSSMPLGSS

VHCVELYPGRGGQMVRTAGASAQVMAKEGDYVALKLPSTEVRLVRKECYATLGEVGNSEIRNTSLGKAGR

RRWLGRRPQVRGSVMNPCDHPHGGGEGKAPVGRAGPVTPWGKAALGLKTRKKNKPSNKYVLRKRRKVSKR

SRGGRDS

>gi|123961772|gb|ABM76555.1| 30S Ribosomal protein S19 [Prochlorococcus marinus str. NATL1A]

MGRSLKKGPFISDSLLRKIEKQNSNDDKAVIKTWSRASTILPLMIGHTIAVHNGKSHIPVFITEQMVGHK

LGEFAPTRTYRGHIRDKKGAR

>gi|123961767|gb|ABM76550.1| 30S Ribosomal protein S17 [Prochlorococcus marinus str. NATL1A]

MALKEMVGTVVSDKMQKTVVVAVENRFPHPIYQKIISRTTRYKAHDAENHCKVGDRVRIKESPPISAHKR

WTVTDVLVKGMKSKEAAK

>gi|123961766|gb|ABM76549.1| 50S Ribosomal protein L14 [Prochlorococcus marinus str. NATL1A]

MIQQETFLTVADNSGAKRLQCIRVLGSNRRYAHVGDVIVASVKDAMPNMGVKKSDVVKAVVVRTRATMRR

ETGNSIRFDDNAAVLINDDQNPRGTRVFGPVARELRERNFTKIVSLAPEVI

>gi|123961765|gb|ABM76548.1| 50S ribosomal protein L24 [Prochlorococcus marinus str. NATL1A]

MAAINKTKGSADRIKMKIRKGDTVQVISGKDKGKTGEVLKTLPYENRVLVQGINQRTKHVKPSQEGETGR

IETKEFPLHASNVMIYSTKEKVASKVEIFVDKDGSKKRRLKKTGELID

>gi|123961763|gb|ABM76546.1| 30S ribosomal protein S8 [Prochlorococcus marinus str. NATL1A]

MANHDPISDMLTRIRNASEKRHEKTKVPASRMSLSIAKVLQSEGFIAEINEEGEGFRKQLILGLKYTGKH

RSPIIRSMQRVSRPGLRIYKNTRGLPKVLGGLGIAIISTSNGVMSDRDARKQGVGGEVLCYVC

>gi|123961761|gb|ABM76544.1| 50S ribosomal protein L18 [Prochlorococcus marinus str. NATL1A]

MSKLSRKQQTQKRHKRLRRNLSGTESRPRLAVFRSNNHIYAQIIDDDAQNTICAASTLDKDLKASLKVNA

GSCDASTAVGELVAKKALSKGIKQVIFDRGGNIYHGRVKALAEAARVAGLNF

>gi|123961759|gb|ABM76542.1| 50S ribosomal protein L15 [Prochlorococcus marinus str. NATL1A]

MTIKLESLQSNKGSRRKKMRKGRGIAAGQGASCGFGMRGQKSRSGRPTRPGFEGGQMPLYRRVPKLKHFP

IVNQKNFTVLNVSRLNALKDGTIVNLDLLVKEGILTKPKNPLKILGNGNLEVKLTVQAAAFTASAKKKIE

EVGGSCELYN

>gi|123961756|gb|ABM76539.1| 30S ribosomal protein S13 [Prochlorococcus marinus str. NATL1A]

MARISGIDIPREKRVEVALTYIYGIGLTRAQAILEKSGVNPDIRVKDLDDGDIQKLRAVTEEFTLEGDLR

RQEGMALKRLQDIGCVRGRRHRMSLPVRGQRTRTNARTRRGARKTVAGRKK

>gi|123961755|gb|ABM76538.1| 30S ribosomal protein S11 [Prochlorococcus marinus str. NATL1A]

MATTSKKTGSKKSKRNVPNGVVHIQSTFNNTIVSITDTSGEVISWSSAGASGFKGARKGTPFAAQTAAEL

AARRALEQGMRQIEVLVRGPGSGRETAIRALQVAGLEITLIRDVTPLPHNGCRRPKRRRV

>gi|123961753|gb|ABM76536.1| 50S ribosomal protein L17 [Prochlorococcus marinus str. NATL1A]

MRHQRRIPQLSLPADQRKALLRGLTTQLIREGRVTTTKARAKALRNETERMITLAKDGSLASRRRAIGYV

YDKQLVHALFEKAQERYGDREGGYTRIVRTTPRRGDNSEMAIVELV

>gi|123961751|gb|ABM76534.1| 50S ribosomal protein L13 [Prochlorococcus marinus str. NATL1A]

MNKTSVPSPDSIDRQWFLVDAENQTLGRLATEVASVLRGKTKPNFTPHLDTGDFVIVVNAEKIKVTGKKS

DQKLYRRHSGRPGGMKVETFKALQSRIPERIVEKAIKGMLPHTRLGRQLFTKLKVYKGSDHPHSAQEPKI

LSLNSESVTK

>gi|123961750|gb|ABM76533.1| 30S ribosomal protein S9 [Prochlorococcus marinus str. NATL1A]

MTSSTSKVVYWGTGRRKTSVARVRLTPGKGEIIINGRPGDHYLNFNPAYISAVKAPLKTLGLSESYDVLV

NVYGGGLTGQSDAIKQGAARALCTLSLDNRKSLKLEGHLSRDPRAKERRKYGLKKARKAPQFSKR

>gi|123961749|gb|ABM76532.1| 50S ribosomal protein L31 [Prochlorococcus marinus str. NATL1A]

MPKSDIHPTWYPEAKVICNGEVVMTTGATQPEIQVDVWSGNHPFFTGTQKILDTEGRVDRFMRKYRMASS

DSSEQKDKSSEEKKES

>gi|123961725|gb|ABM76508.1| 30S ribosomal protein S12 [Prochlorococcus marinus str. NATL1A]

MNLVELRPNIKLMPTIQQLIRTERKTLKTKTKSPALRGCPERRGVCTRVYTSTPKKPNSALRKVARVRLT

SGFEVTAYIGGIGHNLQEHSVVLLRGGRVKDLPGVRYHIVRGTLDTAGVKDRRQSRSKYGAKSPKE

>gi|123961724|gb|ABM76507.1| 30S ribosomal protein S7 [Prochlorococcus marinus str. NATL1A]

MSRRNAAEKRPVLPDPQFNNRLASMMVHRLMKHGKKSTAQKILSDAFGLINERTGSDPIELFETAVKNVT

PLVEVRARRVGGATYQVPMEVRQERGIAMALRWLVNFSRSRNGRSMAQKLAGELMDAANEAGNAVRKREE

THKMAEANKAFAHYRY

>gi|123961721|gb|ABM76504.1| 30S ribosomal protein S10 [Prochlorococcus marinus str. NATL1A]

MSTAIAQQKIRIRLKAFDRRMLDLSCDKIIETADTTAASAIGPIPLPTKRKIYCVLRSPHVDKDSREHFE

TRTHRRIIDIYSPSAKTIDALMKLDLPSGVDIEVKL

>gi|123961661|gb|ABM76444.1| 30s Ribosomal protein S20 [Prochlorococcus marinus str. NATL1A]

MANTNSAKKRIQIAERNRLENKNYKSTVRTLMKRCFVACGIFEKEPGDESKADLQKTFNLAFSKIDKAVK

KGVLHKNTGANQKSRLSVALKKVLKEVV

>gi|123961554|gb|ABM76337.1| putative methyltransferase for Ribosomal protein L11 [Prochlorococcus marinus str. NATL1A]

MLVEKTVNLQNSQLSWLRLEQEFSFELEDSFYWLLSKLDIHRFSFEHDPDNNSTQKLFIWLPLNEWSVRD

QEILVQSLISLTEPFDLTLPECKWIQVKDEDWSLSWKKNWKPDPVGKSILILPAWLDVPEKFLERKIIRL

DPGSAFGTGSHPSTRLCLEALDNEPPVGQTIADIGCGSGILSLTALKLGAKSTFSVDTDSLSISATKINS

ALNDCSENLLNVFLGSIEEIEANMPKEKIDLILCNILAPVIKLLGPSFEKIIGHKGKVILSGLLVQQIKE

LQEFFLKLGWQVLEIKKKDQWALMVLTLNLS

>gi|123961545|gb|ABM76328.1| 50S ribosomal protein L27 [Prochlorococcus marinus str. NATL1A]

MAHKKGTGSTRNGRDSNSKRLGVKAYGGEKVTAGSILIRQRGTSVLPGINVGRGKDDTLFALTDGSVHFE

SIRRGLRNRKRINISTAKAV

>gi|123961544|gb|ABM76327.1| 50S ribosomal protein L21 [Prochlorococcus marinus str. NATL1A]

MADKKSSPKKENPQDKTYAIVEASGKQFWLQPNRYYDFDRCQAEVDDVLTLENVLLLNDGKDLKLGKPYV

KDAKVEIKVLEHRRGPKIIVYKMRPKKKTRRKNGHRQELTRVLVQSISIGSNTKKSKAVKTTASKVESD

>gi|123961478|gb|ABM76261.1| 30S Ribosomal protein S16 [Prochlorococcus marinus str. NATL1A]

MIKLRLKRFGKKRETSFRLVACNSTSRRDGRPLQELGFYNPRTKETRLDTEALRTRLGQGAQPTDAVRTL

LEKGGLLEKKVRPAEVLGKQKQEKERSAKKKDATASETSE

>gi|123961438|gb|ABM76221.1| 30S Ribosomal protein S14 [Prochlorococcus marinus str. NATL1A]

MAKKSMIARDVKRKKLVERYAAKRKSLIDAFKSAKDPMERLEIHRKIQALPRNCAPNRIRNRCWATGKPR

GVYRDFGLCRNQLRSRAHNGELPGVVKSSW

>gi|123960788|gb|ABM75571.1| 30S Ribosomal protein S18 [Prochlorococcus marinus str. NATL1A]

MTNSLFKQKLSPIKPGDPIDYKDVELLKKFITDRGKILPRRLTGLTAKQQRDLTTAVKRARIIALLPFVN

PEG

>gi|123960787|gb|ABM75570.1| 50S Ribosomal protein L33 [Prochlorococcus marinus str. NATL1A]

MAKKGTRIVVTLECTESRSVPSSEKRSAGVSRYTTEKNRRNTTERLELKKFCPELNKMTIHREIK

>gi|123960753|gb|ABM75536.1| 50S ribosomal protein L28 [Prochlorococcus marinus str. NATL1A]

MSRVCQLTGTRANNGMSVSHSHIRTKKLQQANLQQRRLWWEEENKWINIRVTTRALKTIQKKGLGKYAKS

LGVDLNKL

>gi|123960711|gb|ABM75494.1| 30S Ribosomal protein S15 [Prochlorococcus marinus str. NATL1A]

MSLGTEEKQNLINTHQVHPTDTGSVEVQVAMLTTRISKLSTHLQGNIHDFSSRQGLLKMIGQRKRLLGYV

RSKSEKRYTELIEKLAIRG

>gi|123960564|gb|ABM75347.1| 30S ribosomal protein S2 [Prochlorococcus marinus str. NATL1A]

MMEAGAHFGHQTRRWNPKMSRYIYSARNGVHIIDLVKTAVCMNSAYKWTRGAARSGKRFLFVGTKKQASE

VVAQEAIRCGASYVNQRWLGGMLTNWTTMKARIDRLKDLERMESSGAIAMRPKKEGAVLRRELERLQKYL

GGLKGMRRLPDVVVLVDQRRETNAVLEARKLDIPLVSMLDTNCDPDLCEIPIPCNDDAVRSVQLVLGRLA

DAINEGRHGPNE

>gi|123960310|gb|ABM75093.1| Ribosomal protein L19 [Prochlorococcus marinus str. NATL1A]

MSVDSKEPSSEEVKIEDESNASEDSGVKTASENKSKGKKISIKNLSPAEIIKTFEDAQLGKDLPDIYVGD

TVRVGVRISEGNKERVQPYEGVVIAKRHGGIHQTITVRRIFQGIGVERIFLVHSPQVASIKVERRGKVRR

AKLFYLRERVGKATRVKQRFDR

>gi|123961769|gb|ABM76552.1| 50S ribosomal protein L16 [Prochlorococcus marinus str. NATL1A]

MLSPKRTKFRKQQRGRMRGVATRGNKIAFGQFALQAQDCGWVTSRQIEASRRAMTRYVKRGGQIWIRIFP

DKPVTMRPAETRMGSGKGNPEFWVAVVKPGRILFEMGGDEITETIAKEAMRLAQYKLPVKTKFISLDEDL

NKGNYKPAKTPVTADDSESSS

>gi|123961768|gb|ABM76551.1| 50S ribosomal protein L29 [Prochlorococcus marinus str. NATL1A]

MSKKTTKDVRNLSDSEMSDKIQNLRKELFDLRFKQATRQLAKTHRFKEARTELAQLLTVSNERSRSNTSS

>gi|123960810|gb|ABM75593.1| 50S ribosomal protein L32 [Prochlorococcus marinus str. NATL1A]

MAVPKKKTSKGKRNQRHATWKGKAAVAAEKALSIGKSVLTGRAQGFVYPMNETSEEEAD

>gi|123961431|gb|ABM76214.1| 50S ribosomal protein L34 [Prochlorococcus marinus str. NATL1A]

MTKRTFGGTSRKRKRVSGFRVRMRSHTGRRVVRTRRKRGRSRLTV

>gi|123961439|gb|ABM76222.1| polyribonucleotide nucleotidyltransferase [Prochlorococcus marinus str. NATL1A]

MQGQTKSVSFDGREIKLTTGRFAPQAGGSVMIECGDTSVLVTATKSSGREGVDFLPLMCDYEERLYAAGR

IPGSFMRREGRPPERATLISRLIDRPMRPLFPGWMRDDIQIVATCLSLDERVPADVLAVTGASMATLMAG

IPFQGPMAAVRVGLLGDDFVLNPSYREIERGDLDLVVAGTPDGVVMVEAGANQLSEQDVIEAIDFGYEAI

TELINAQKEVLKESGIKQEMPKAPEIDDTISTYLDKNCTKSISEVLKNFDQTKEERDNKIEEIKISISAK

IDGLKDDNAVKKSLSLNNKLLENSYKALTKKLMREQIIKEGKRVDGRELNEVRAIEADAAVLPNRVHGSA

LFQRGLTQVLSTATLGTPSDAQEMDDLNPNTDKTYIHHYNFPPYSVGETRPMRTPGRREVGHGALAERAL

IPVLPAKDTFPYVLRVVSEVLSSNGSTSMASVCGSTLALMDAGVPLKAPVGGAAMGLIKEGKEVRILTDI

QGIEDFLGDMDFKVAGTEKGITALQMDMKITGLPIETIGEAINQALPARTHILGKMLDAIETPKDNLSPH

APRLLSFRIDPELIGTVIGPGGRTIKGITERTNTKIDIEDGGIVTIASHDGAAAEEAQRIIEGLTRKVHE

GEIFPGSITRIIPIGAFVEILPGKEGMIHISQLSEARVEKVEDVVKVGDQVTVRVREIDNRGRINLTLRG

VSQNGGMSNYPEPTPTPVAPLT

>gi|123961265|gb|ABM76048.1| putative ribosomal-protein-alanine acetyltransferase [Prochlorococcus marinus str. NATL1A]

MNLKIIQLREMHLNDCVDLDQKSSNGLWSKSQWKKELTDPKRICLGVKELQTKKLLGLCSAWLVIDELHI

TFIAVDPMNQRKGIGKFLLSDLIKRSKSLQINHIFLEVKQNNESAKALYNSMGFKTVGKRSNFYQDGSDA

LLLNKETHYKS

>gi|123959799|gb|ABM74582.1| Predicted GTPases [Prochlorococcus marinus str. NATL1A]

MNKNKSNKLKGIVVALKANFLIVEIDHKNFKDYSFDEFNGKIRLLCIRRSKLNYQGLFIDVGDIVGVESI

DYKNKRAVVSDVEPRQSFLKRPAVANVTLVSICISADEPLFDMEQTSRFLLTAECANIEPLIILTKIDLI

TKNDLILYINKFKSWGYDCIPVSIHNSQGIDSLIERLRKTKLTVLAGPSGVGKTSLINHLIPTVSLPTSS

VSKKLKRGTHTTRHVELFAIGNGSLLADTPGFNRPEIVCEPSDFAFLFPEFRTQLSNSQCKFRNCLHRDE

PGCVIDKDLERYPFYRENLEEMINSPLPYQAG

>gi|123961708|gb|ABM76491.1| Hypothetical protein NATL1_19351 [Prochlorococcus marinus str. NATL1A]

MTQVTVGENEGIESALRRFKRQVSKAGIFGELKRLRHHETPVEKYKRKLQQRRRSRRR

>gi|166230047|sp|A2C032.1|RL10_PROM1 RecName: Full=50S ribosomal protein L10

MGRTLESKKQIVKKIEDLLDNSEMALVLDYKGLSTKEMSDLRSRLQQSDGVCKVTKNTLMRQAIKGKNSW

TGLDSLLTGTNAFVLIKGDVGSAVKAVQAFQKETQKSETKGGLFEGKLLSQDEIKAIAKLPSKEALMGQI

AGALNSITSKIAIGINEVPSGLARSLKQHSESGES

>gi|189045479|sp|A2C4Y3.1|RS5_PROM1 RecName: Full=30S ribosomal protein S5

MTDSNNQSPNKKTSGSSGAPTAADGRQENRRSRGEKRGGRRDRRGQERDSEWQERVVQIRRVSKTVKGGK

KMSFRAIVVVGNEKGQVGVGVGKAGDVIGAVRKGVADGKKHLVRVPLTRNSSIPTLSNGRDGAASVLIRP

AAPGTGVIAGGSIRTVLELAGIKNVLAKRLGSKTPLNNARAAMVALSELRTHKATAKERGISLEQIYS

>gi|166230911|sp|A2C034.1|RL11_PROM1 RecName: Full=50S ribosomal protein L11

MAKKVVALIKLALQAGKANPAPPVGPALGQHGVNIMAFCKEYNSRTQDKAGFVIPVEISVFEDRSFSFIT

KTPPASVLITKAAGIAKGSGESAKGSAGSISTSQLEEIAKTKLPDLNCSSIESAMKVIEGTAKNMGVSIK

D

>gi|166223841|sp|A2C5D9.1|RL9_PROM1 RecName: Full=50S ribosomal protein L9

MAKRVQVVLNEDIKSLGNDGDLVEVAPGFARNFLLPNKKALPVTPTVLKQVEHRRAKQAEKEAAKKQEAI

DFQTALTTIGRFTVKKQVGEDGVLFGTVTNGDVAEVVKEATKKDIDRRDISVPEIHGVGKYKVQIKLHNE

VNAEINLEVTSY

>gi|166221278|sp|A2C4Y5.1|RL6_PROM1 RecName: Full=50S ribosomal protein L6

MSRTGKKPISLPEKVDVKFEGLSITVKGPKGELQRTLPNGVSLSKDENFIFVKPINEKRQSREMHGLCRS

LVANMVEGVSNGFTKKLEIVGVGSRAQVKGKTLVVSAGYSHPVEVVPPEGITFKVENNTNVIVTGPDKEL

VGNEAAKIRAIRPPEPYKGKGIKYQGELIIRKAGKSGKT

>gi|215274851|sp|A2C4Z4.1|RL22_PROM1 RecName: Full=50S ribosomal protein L22

MTTSSTKTTAQAHGRYIRGSASKVRRVLDQIRGRTYRDALIMLEFMPYRSTEPITKVLRSAVANAENNLG

LDPSSLMISTATADMGPPMKRYRPRAQGRAFAIKKQTCHISISVSATQSTNSEDSD

>gi|166222193|sp|A2C031.1|RL7_PROM1 RecName: Full=50S ribosomal protein L7/L12

MSAKTDEILDSLKSLSLLEASELVKQIEEAFGVSAAASAGVVMAAPGAAAGGDGADAAEEKTEFEVVLES

FDASSKIKVLKEVRNATGLGLGEAKALVEAAPKTIKEGATKEDAEALKKAIEAVGGKVTLK

>gi|166216364|sp|A2C4Y7.1|RL5_PROM1 RecName: Full=50S ribosomal protein L5

MSLKTRYRETIRPKLLKDLGLKNVHQVPKVQKVTLNRGLGEAATNSKALEASLKEMATISGQKALVTRAK

KAIATFKIRQGMPIGCSVTLRGDRMYAFLERFINLALPRIRDFRGVSPKSFDGRGNYTIGVKEQLIFPEI

TFDKVDTIRGMDITIVTSASSDEQGKALLSEMGMPFRKK

>gi|160166294|sp|A2C033.1|RL1_PROM1 RecName: Full=50S ribosomal protein L1

MKNFSKRMTTLLSKVEERSYSPIEAIKLVKENANAKFDETIEAHIRLGIDPKYTDQQLRTTVALPSGTGQ

KIRIAVVTRGEKVNEATKAGADLAGEEDLVDSINKGEMNFDLLISTPDMMPKVAKLGRVLGPRGLMPNPK

AGTVTTDLEGAIKEFKAGKLEFRADKAGIVHVRFGKASFSEEALLENLKTLQTTIEKNKPSGAKGKFWRS

FFITSTMGPSVEVDINELQDLQKEK

>gi|152112245|sp|A2C0L5.1|RS4_PROM1 RecName: Full=30S ribosomal protein S4

MSRYRGPRLRITRRLGDLPGLTRKAAKRSHPPGQHGQARRKRSEYAIRLEEKQKLRFNYGISERQLVRYV

KKARAQEGSTGTNLLKLLENRLDNVCFRLGFGPTIPGSRQLVNHGHVTVNGKITDIASYQCKAGDVIAIR

DNKASKQLAQANLEFPGLANVPPHLELDKTKLSAKITAKTDREWVAIEINELLVVEYYSRKV

>gi|152060892|sp|A2C4Z3.1|RS3_PROM1 RecName: Full=30S ribosomal protein S3

MGHKIHPTGIRLGITQEHRSKWYAPSKTYPLLLQEDDRIRTFIKKKYVAAGISDVLIARKADQLEVELKT

ARPGVIVGRQGSGIEELRSGIQKTIGDRNRQVRINVVEVEKVDADAYLLAEYIAQQLEKRVAFRRTIRMA

VQRAQRAGVLGLKIQVGGRLNGAEIARSEWTREGRVPLHTLRAEIDYATKVASTTYGVLGIKVWVFKGEV

LPKEEQPLPVGSSPRRSRGNRRPQQFEDRSNESK

>gi|226708145|sp|A2C4X2.1|RL31_PROM1 RecName: Full=50S ribosomal protein L31

MPKSDIHPTWYPEAKVICNGEVVMTTGATQPEIQVDVWSGNHPFFTGTQKILDTEGRVDRFMRKYRMASS

DSSEQKDKSSEEKKES

>gi|166231010|sp|A2C4X4.1|RL13_PROM1 RecName: Full=50S ribosomal protein L13

MNKTSVPSPDSIDRQWFLVDAENQTLGRLATEVASVLRGKTKPNFTPHLDTGDFVIVVNAEKIKVTGKKS

DQKLYRRHSGRPGGMKVETFKALQSRIPERIVEKAIKGMLPHTRLGRQLFTKLKVYKGSDHPHSAQEPKI

LSLNSESVTK

>gi|229485530|sp|A2C260.1|RL33_PROM1 RecName: Full=50S ribosomal protein L33

MAKKGTRIVVTLECTESRSVPSSEKRSAGVSRYTTEKNRRNTTERLELKKFCPELNKMTIHREIK

>gi|226731531|sp|A2C411.1|RS14_PROM1 RecName: Full=30S ribosomal protein S14

MAKKSMIARDVKRKKLVERYAAKRKSLIDAFKSAKDPMERLEIHRKIQALPRNCAPNRIRNRCWATGKPR

GVYRDFGLCRNQLRSRAHNGELPGVVKSSW

>gi|218547105|sp|A2C4Y8.1|RL24_PROM1 RecName: Full=50S ribosomal protein L24

MAAINKTKGSADRIKMKIRKGDTVQVISGKDKGKTGEVLKTLPYENRVLVQGINQRTKHVKPSQEGETGR

IETKEFPLHASNVMIYSTKEKVASKVEIFVDKDGSKKRRLKKTGELID

>gi|166987296|sp|A2C4Z7.1|RL23_PROM1 RecName: Full=50S ribosomal protein L23

MTKFFEKRLTDVIKRPLITEKATKALDLNQYTFEVDPRAAKPDIKAAVEKMFDVKVLGISTMNPPRKSRR

VGRFSGKRPQVKKAVVRLAEGNSIQLFPESEEA

>gi|166986913|sp|A2C4B7.1|RL21_PROM1 RecName: Full=50S ribosomal protein L21

MADKKSSPKKENPQDKTYAIVEASGKQFWLQPNRYYDFDRCQAEVDDVLTLENVLLLNDGKDLKLGKPYV

KDAKVEIKVLEHRRGPKIIVYKMRPKKKTRRKNGHRQELTRVLVQSISIGSNTKKSKAVKTTASKVESD

>gi|166234498|sp|A2C4Y2.1|RL15_PROM1 RecName: Full=50S ribosomal protein L15

MTIKLESLQSNKGSRRKKMRKGRGIAAGQGASCGFGMRGQKSRSGRPTRPGFEGGQMPLYRRVPKLKHFP

IVNQKNFTVLNVSRLNALKDGTIVNLDLLVKEGILTKPKNPLKILGNGNLEVKLTVQAAAFTASAKKKIE

EVGGSCELYN

>gi|166234342|sp|A2C1Y4.1|RS15_PROM1 RecName: Full=30S ribosomal protein S15

MSLGTEEKQNLINTHQVHPTDTGSVEVQVAMLTTRISKLSTHLQGNIHDFSSRQGLLKMIGQRKRLLGYV

RSKSEKRYTELIEKLAIRG

>gi|166233176|sp|A2C4Z9.1|RL3_PROM1 RecName: Full=50S ribosomal protein L3

MSLGILGKKLGMSQLFDDQGRAVPVTLIEAGPCRITQLKSADTDGYAAVQIGFQLIREKLINKPSKGHLA

KSGNDLLRHLREYRVENSSEFELGASITVDDFEKGQKVDVSGDTMGRGFAGYQKRHGFSRGPMSHGSKNH

RLPGSIGAGTTPGRVYPGKRMAGRMGGKKVTTRALEILKIDTNHNLLVVKGSVPGKPGSLLNIRPAKRVG

VSIQQGGE

>gi|166232682|sp|A2C4Y9.1|RL14_PROM1 RecName: Full=50S ribosomal protein L14

MIQQETFLTVADNSGAKRLQCIRVLGSNRRYAHVGDVIVASVKDAMPNMGVKKSDVVKAVVVRTRATMRR

ETGNSIRFDDNAAVLINDDQNPRGTRVFGPVARELRERNFTKIVSLAPEVI

>gi|166231309|sp|A2C4U4.1|RS10_PROM1 RecName: Full=30S ribosomal protein S10

MSTAIAQQKIRIRLKAFDRRMLDLSCDKIIETADTTAASAIGPIPLPTKRKIYCVLRSPHVDKDSREHFE

TRTHRRIIDIYSPSAKTIDALMKLDLPSGVDIEVKL

>gi|166228459|sp|A2C4U7.1|RS7_PROM1 RecName: Full=30S ribosomal protein S7

MSRRNAAEKRPVLPDPQFNNRLASMMVHRLMKHGKKSTAQKILSDAFGLINERTGSDPIELFETAVKNVT

PLVEVRARRVGGATYQVPMEVRQERGIAMALRWLVNFSRSRNGRSMAQKLAGELMDAANEAGNAVRKREE

THKMAEANKAFAHYRY

>gi|166227363|sp|A2C5I5.1|RS6_PROM1 RecName: Full=30S ribosomal protein S6

MSETPYYETMYILRPDIPEEEVDSHLKKYSAILEKSETEVLDSQMRGKRRLAYPIAKHKEGIYVQLSHKG

NGQQVATLERAMRLSEDVIRYITVKQDGPLPTPKPTSKEDETEKEEVKPTEDKTESPAQEEKKEDSKE

>gi|166226599|sp|A2C226.1|RL28_PROM1 RecName: Full=50S ribosomal protein L28

MSRVCQLTGTRANNGMSVSHSHIRTKKLQQANLQQRRLWWEEENKWINIRVTTRALKTIQKKGLGKYAKS

LGVDLNKL

>gi|166225030|sp|A2C4B8.1|RL27_PROM1 RecName: Full=50S ribosomal protein L27

MAHKKGTGSTRNGRDSNSKRLGVKAYGGEKVTAGSILIRQRGTSVLPGINVGRGKDDTLFALTDGSVHFE

SIRRGLRNRKRINISTAKAV

>gi|166224051|sp|A2C4T1.1|RS21_PROM1 RecName: Full=30S ribosomal protein S21

MTQVTVGENEGIESALRRFKRQVSKAGIFGELKRLRHHETPVEKYKRKLQQRRRSRRR

>gi|166223966|sp|A2C4N4.1|RS20_PROM1 RecName: Full=30S ribosomal protein S20

MANTNSAKKRIQIAERNRLENKNYKSTVRTLMKRCFVACGIFEKEPGDESKADLQKTFNLAFSKIDKAVK

KGVLHKNTGANQKSRLSVALKKVLKEVV

>gi|166220971|sp|A2C261.1|RS18_PROM1 RecName: Full=30S ribosomal protein S18

MTNSLFKQKLSPIKPGDPIDYKDVELLKKFITDRGKILPRRLTGLTAKQQRDLTTAVKRARIIALLPFVN

PEG

>gi|166219719|sp|A2C4Z0.1|RS17_PROM1 RecName: Full=30S ribosomal protein S17

MALKEMVGTVVSDKMQKTVVVAVENRFPHPIYQKIISRTTRYKAHDAENHCKVGDRVRIKESPPISAHKR

WTVTDVLVKGMKSKEAAK

>gi|166219677|sp|A2C5C7.1|RL20_PROM1 RecName: Full=50S ribosomal protein L20

MARVKRGNVARKRRNKILRLARGFRGGNGTLFRTANQRVMKALCNAYRDRRRRKRDFRRLWIARINAAAR

LNGLSYSKFMGGLKKADIRINRKMLAQLAVIDPKTFTNVAVNSKS

>gi|166218717|sp|A2C4Y4.1|RL18_PROM1 RecName: Full=50S ribosomal protein L18

MSKLSRKQQTQKRHKRLRRNLSGTESRPRLAVFRSNNHIYAQIIDDDAQNTICAASTLDKDLKASLKVNA

GSCDASTAVGELVAKKALSKGIKQVIFDRGGNIYHGRVKALAEAARVAGLNF

>gi|166216201|sp|A2C4X6.1|RL17_PROM1 RecName: Full=50S ribosomal protein L17

MRHQRRIPQLSLPADQRKALLRGLTTQLIREGRVTTTKARAKALRNETERMITLAKDGSLASRRRAIGYV

YDKQLVHALFEKAQERYGDREGGYTRIVRTTPRRGDNSEMAIVELV

>gi|166201332|sp|A2C451.1|RS16_PROM1 RecName: Full=30S ribosomal protein S16

MIKLRLKRFGKKRETSFRLVACNSTSRRDGRPLQELGFYNPRTKETRLDTEALRTRLGQGAQPTDAVRTL

LEKGGLLEKKVRPAEVLGKQKQEKERSAKKKDATASETSE

>gi|166199901|sp|A2C4Z5.1|RS19_PROM1 RecName: Full=30S ribosomal protein S19

MGRSLKKGPFISDSLLRKIEKQNSNDDKAVIKTWSRASTILPLMIGHTIAVHNGKSHIPVFITEQMVGHK

LGEFAPTRTYRGHIRDKKGAR

>gi|166199817|sp|A2C5C6.1|RL35_PROM1 RecName: Full=50S ribosomal protein L35

MPKLKTRKAAAKRFKATGTGKFMRRRAFHNHLLDHKSPKLKRHLKTKAVVDERDAENVRLMLPYA

>gi|160358605|sp|A2C4Z6.1|RL2_PROM1 RecName: Full=50S ribosomal protein L2

MAIRSFKPYTPGTRSRVVTDFSEVTSSKPERSLVVSKHRKKGRNNRGVITCRHRGGGHKRLYRIVDFRRN

KHGVSAKVAAIHYDPHRNARLALLFYSDGEKRYILAPADITVGQEVISGPDAPIETGNALPLSSMPLGSS

VHCVELYPGRGGQMVRTAGASAQVMAKEGDYVALKLPSTEVRLVRKECYATLGEVGNSEIRNTSLGKAGR

RRWLGRRPQVRGSVMNPCDHPHGGGEGKAPVGRAGPVTPWGKAALGLKTRKKNKPSNKYVLRKRRKVSKR

SRGGRDS

>gi|158706260|sp|A2C4X9.1|RS13_PROM1 RecName: Full=30S ribosomal protein S13

MARISGIDIPREKRVEVALTYIYGIGLTRAQAILEKSGVNPDIRVKDLDDGDIQKLRAVTEEFTLEGDLR

RQEGMALKRLQDIGCVRGRRHRMSLPVRGQRTRTNARTRRGARKTVAGRKK

>gi|156637361|sp|A2C4U8.2|RS12_PROM1 RecName: Full=30S ribosomal protein S12

MPTIQQLIRTERKTLKTKTKSPALRGCPERRGVCTRVYTSTPKKPNSALRKVARVRLTSGFEVTAYIGGI

GHNLQEHSVVLLRGGRVKDLPGVRYHIVRGTLDTAGVKDRRQSRSKYGAKSPKE

>gi|156630851|sp|A2C283.1|RL32_PROM1 RecName: Full=50S ribosomal protein L32

MAVPKKKTSKGKRNQRHATWKGKAAVAAEKALSIGKSVLTGRAQGFVYPMNETSEEEAD

>gi|152060806|sp|A2C4X8.1|RS11_PROM1 RecName: Full=30S ribosomal protein S11

MATTSKKTGSKKSKRNVPNGVVHIQSTFNNTIVSITDTSGEVISWSSAGASGFKGARKGTPFAAQTAAEL

AARRALEQGMRQIEVLVRGPGSGRETAIRALQVAGLEITLIRDVTPLPHNGCRRPKRRRV

>gi|148841160|sp|A2C4Y6.1|RS8_PROM1 RecName: Full=30S ribosomal protein S8

MANHDPISDMLTRIRNASEKRHEKTKVPASRMSLSIAKVLQSEGFIAEINEEGEGFRKQLILGLKYTGKH

RSPIIRSMQRVSRPGLRIYKNTRGLPKVLGGLGIAIISTSNGVMSDRDARKQGVGGEVLCYVC

>gi|209573695|sp|A2C1I7.2|RS2_PROM1 RecName: Full=30S ribosomal protein S2

MAVVSLSEMMEAGAHFGHQTRRWNPKMSRYIYSARNGVHIIDLVKTAVCMNSAYKWTRGAARSGKRFLFV

GTKKQASEVVAQEAIRCGASYVNQRWLGGMLTNWTTMKARIDRLKDLERMESSGAIAMRPKKEGAVLRRE

LERLQKYLGGLKGMRRLPDVVVLVDQRRETNAVLEARKLDIPLVSMLDTNCDPDLCEIPIPCNDDAVRSV

QLVLGRLADAINEGRHGPNE

>gi|166199806|sp|A2C404.1|RL34_PROM1 RecName: Full=50S ribosomal protein L34

MTKRTFGGTSRKRKRVSGFRVRMRSHTGRRVVRTRRKRGRSRLTV

>gi|166228240|sp|A2C4Z1.1|RL29_PROM1 RecName: Full=50S ribosomal protein L29

MSKKTTKDVRNLSDSEMSDKIQNLRKELFDLRFKQATRQLAKTHRFKEARTELAQLLTVSNERSRSNTSS

>gi|166199702|sp|A2C4Z2.1|RL16_PROM1 RecName: Full=50S ribosomal protein L16

MLSPKRTKFRKQQRGRMRGVATRGNKIAFGQFALQAQDCGWVTSRQIEASRRAMTRYVKRGGQIWIRIFP

DKPVTMRPAETRMGSGKGNPEFWVAVVKPGRILFEMGGDEITETIAKEAMRLAQYKLPVKTKFISLDEDL

NKGNYKPAKTPVTADDSESSS

>gi|254766822|sp|A2BZC2.1|RSGA_PROM1 RecName: Full=Putative ribosome biogenesis GTPase RsgA

MNKNKSNKLKGIVVALKANFLIVEIDHKNFKDYSFDEFNGKIRLLCIRRSKLNYQGLFIDVGDIVGVESI

DYKNKRAVVSDVEPRQSFLKRPAVANVTLVSICISADEPLFDMEQTSRFLLTAECANIEPLIILTKIDLI

TKNDLILYINKFKSWGYDCIPVSIHNSQGIDSLIERLRKTKLTVLAGPSGVGKTSLINHLIPTVSLPTSS

VSKKLKRGTHTTRHVELFAIGNGSLLADTPGFNRPEIVCEPSDFAFLFPEFRTQLSNSQCKFRNCLHRDE

PGCVIDKDLERYPFYRENLEEMINSPLPYQAG

>gi|166226463|sp|A2C552.1|RIMM_PROM1 RecName: Full=Ribosome maturation factor RimM

MFEKDKWMSIGEIVAPQGLRGDLRIKPSSDFPERFTKPGKRWIQKTDELPTEIKLTKGKLIPGKSIYVLS

IEGVSTRSSAEEIIGWKLVIPIDSRPMLSKDEYHYHDLIGLEARSGPSKALIGYVTDLIKGGNDLLEIEL

VEGKKVLVPFVKEIVPEIEIKEKWLLINPPPGLLEL

>gi|187610310|sp|A2C412.1|PNP_PROM1 RecName: Full=Polyribonucleotide nucleotidyltransferase; AltName: Full=Polynucleotide phosphorylase; Short=PNPase

MQGQTKSVSFDGREIKLTTGRFAPQAGGSVMIECGDTSVLVTATKSSGREGVDFLPLMCDYEERLYAAGR

IPGSFMRREGRPPERATLISRLIDRPMRPLFPGWMRDDIQIVATCLSLDERVPADVLAVTGASMATLMAG

IPFQGPMAAVRVGLLGDDFVLNPSYREIERGDLDLVVAGTPDGVVMVEAGANQLSEQDVIEAIDFGYEAI

TELINAQKEVLKESGIKQEMPKAPEIDDTISTYLDKNCTKSISEVLKNFDQTKEERDNKIEEIKISISAK

IDGLKDDNAVKKSLSLNNKLLENSYKALTKKLMREQIIKEGKRVDGRELNEVRAIEADAAVLPNRVHGSA

LFQRGLTQVLSTATLGTPSDAQEMDDLNPNTDKTYIHHYNFPPYSVGETRPMRTPGRREVGHGALAERAL

IPVLPAKDTFPYVLRVVSEVLSSNGSTSMASVCGSTLALMDAGVPLKAPVGGAAMGLIKEGKEVRILTDI

QGIEDFLGDMDFKVAGTEKGITALQMDMKITGLPIETIGEAINQALPARTHILGKMLDAIETPKDNLSPH

APRLLSFRIDPELIGTVIGPGGRTIKGITERTNTKIDIEDGGIVTIASHDGAAAEEAQRIIEGLTRKVHE

GEIFPGSITRIIPIGAFVEILPGKEGMIHISQLSEARVEKVEDVVKVGDQVTVRVREIDNRGRINLTLRG

VSQNGGMSNYPEPTPTPVAPLT

>gi|72003375|gb|AAZ59177.1| SSU ribosomal protein S1P [Prochlorococcus marinus str. NATL2A]

MSENPASKIEEKNPEKETSIPEETVSNATIAEFEENSITELKEDDIPKNIPAADDSSSRINKSDLETAGF

TLDEFASLLSKYDYNFKPGDIVNGTVFALESKGAMIDIGAKTAAFMPMQEVSINRVEGLSDVLQPSEIRE

FFIMTEENEDGQLSLSIRRIEYQRAWERVRQLQKEDATIYSEVFATNRGGALVRVEGLRGFIPGSHISTR

KAKEELVADFLPLKFLEVDEERNRLVLSHRRALVERKMNRLEVGEVVVGAVRGIKPYGAFIDIGGVSGLL

HISEISHEHIETPHSVLNVNDQMKVMIIDLDAERGRISLSTKALEPEPGDMLTDPQKVFDKAEEMAARYK

QMLLEQAEEGEDPIAVMTI

>gi|72003547|gb|AAZ59349.1| SSU ribosomal protein S1P [Prochlorococcus marinus str. NATL2A]

MAGSDPQPKKATPQRPAINAPRKPLQVMHISRKPEEEIINEESSEKEFSEQPKDRRFAEKVLAPKEISYL

KKAPEQQNSIGKDSSDDFQSETMEDLLRSENNTNYKNNIEAFDDQNIFEQKSRTVDEFDFDEDEFLAALE

ENQPIGTTGEIAKGSVIAVESDGIYVDIGGKAPGFMPKNECGLGVITNLKERFPKGLKVEVLVTREQNAD

GMVTISCRALELRKSWDKVQNLAKEGKVIRVKINGFNRGGVTCDFEGLRGFIPRSQLEDGENHQSLVSKT

ISTAFLEVNPERRKLVLSEKKAAIASRFSELEIGQLIEGEILTIKPYGFFVDLRGVSGLLHHSMVTNGSM

RSLREVFQPGESIKALITDLDPSRGRIGLNTALLEGPPGELITDKTKVMEEANERAIKARNSLNKEKVDP

QKEKEDINLSS

>gi|72003257|gb|AAZ59059.1| LSU ribosomal protein L11P [Prochlorococcus marinus str. NATL2A]

MAKKVVALIKLALQAGKANPAPPVGPALGQHGVNIMAFCKEYNSRTQDKAGFVIPVEISVFEDRSFSFIT

KTPPASVLITKAAGIAKGSGESAKGSAGSISTSQLEEIAKTKLPDLNCSSIESAMKVIEGTAKNMGVSIK

D

>gi|72003255|gb|AAZ59057.1| LSU ribosomal protein L10P [Prochlorococcus marinus str. NATL2A]

MGRTLESKKQIVKKIEDLLDNSEMALVLDYKGLSTKEMSDLRSRLQQSDGVCKVTKNTLMRQAIKGKNSW

TGLDSLLTGTNAFVLIKGDVGSAVKAVQAFQKETQKSETKGGLFEGKLLSQDEIKAIAKLPSKEALMGQI

AGALNSITSKIAIGINEVPSGLARSLKQHSENGES

>gi|72002960|gb|AAZ58762.1| LSU ribosomal protein L9P [Prochlorococcus marinus str. NATL2A]

MAKRVQVVLNEDIKSLGNDGDLVEVAPGFARNFLLPNKKALPVTPTVLKQVEHRRAKQAEKEAAKKQEAI

DFQTALTTIGRFTVKKQVGEDGVLFGTVTNGDVAEVVKEATKKDIDRRDISIPEIHGVGKYKVQIKLHNE

VNAEINLEVTSY

>gi|72002803|gb|AAZ58605.1| LSU ribosomal protein L6P [Prochlorococcus marinus str. NATL2A]

MSRTGKKPISLPEKVDVKFEGLSITVKGPKGELKRTLPNGVSLSKDENFIFVKPINEKRQSREMHGLCRS

LVANMVEGVSNGFTKKLEIVGVGSRAQVKGKTLVVSAGYSHPVEVVPPEGITFKVENNTNVIVTGPDKEL

VGNEAAKIRAIRPPEPYKGKGIKYQGELIIRKAGKSGKT

>gi|72002801|gb|AAZ58603.1| SSU ribosomal protein S5P [Prochlorococcus marinus str. NATL2A]

MTDSNNQSPNKKTSGSSSAPPAADGRQENRRSRGEKRGGRRDRRGQERDSEWQERVVQIRRVSKTVKGGK

KMSFRAIVVVGNEKGQVGVGVGKAGDVIGAVRKGVADGKKHLVRVPLTRNSSIPTLSNGRDGAASVLIRP

AAPGTGVIAGGSIRTVLELAGIKNVLAKRLGSKTPLNNARAAMVALSELRTHKATAKERGISLEQIYS

>gi|72003430|gb|AAZ59232.1| SSU ribosomal protein S4P [Prochlorococcus marinus str. NATL2A]

MSRYRGPRLRITRRLGDLPGLTRKAAKRSHPPGQHGQARRKRSEYAIRLEEKQKLRFNYGISERQLVRYV

KKARAQEGSTGTNLLKLLENRLDNVCFRLGFGPTIPGSRQLVNHGHVTVNGRITDIASYQCKAGDVIAIR

DNKASKQLAQANLEFPGLANVPPHLELDKTKLSAKISAKTDREWVAIEINELLVVEYYSRKV

>gi|72003256|gb|AAZ59058.1| LSU ribosomal protein L1P [Prochlorococcus marinus str. NATL2A]

MKNFSKRMTTLLSKVEERSYSPIEAIKLVKENANAKFDETIEAHIRLGIDPKYTDQQLRTTVALPSGTGQ

KIRIAVVTRGEKVNEATKAGADLAGEEDLVDSINKGEMNFDLLISTPDMMPKVAKLGRVLGPRGLMPNPK

AGTVTTDLEGAIKEFKAGKLEFRADKAGIVHVRFGKASFSEEALLENLKTLQTTIEKNKPSGAKGKFWRS

FFITSTMGPSVEVDINELQDLQKEK

>gi|72003254|gb|AAZ59056.1| LSU ribosomal protein L12P [Prochlorococcus marinus str. NATL2A]

MSAKTDEILDSLKSLSLLEASELVKQIEEAFGVSAAASAGVVMAAPGAAAGGDGADAAEEKTEFEVVLES

FEASSKIKVLKEVRNATGLGLGEAKALVEAAPKTIKEGATKEDAEALKKAIEAVGGKVTLK

>gi|72002812|gb|AAZ58614.1| LSU ribosomal protein L22P [Prochlorococcus marinus str. NATL2A]

MTTSSTKTTAQAHGRYIRGSASKVRRVLDQIRGRTYRDALIMLEFMPYRSTEPITKVLRSAVANAENNLG

LDPSSLMISTATADMGPPMKRYRPRAQGRAFAIKKQTCHISISVSATQSTNSEDSD

>gi|72002811|gb|AAZ58613.1| SSU ribosomal protein S3P [Prochlorococcus marinus str. NATL2A]

MGHKIHPTGIRLGITQEHRSKWYAPSKTYPLLLQEDDRIRTFIKKKYLAAGISDVLIARKADQLEVELKT

ARPGVIVGRQGSGIEELRSGIQKTIGDRNRQVRINVVEVEKVDADAYLLAEYIAQQLEKRVAFRRTIRMA

VQRAQRAGVLGLKIQVGGRLNGAEIARSEWTREGRVPLHTLRAEIDYATKVASTTYGVLGIKVWVFKGEV

LPKEEQPLPVGSSPRRSRGNRRPQQFEDRSNEGK

>gi|72002805|gb|AAZ58607.1| LSU ribosomal protein L5P [Prochlorococcus marinus str. NATL2A]

MSLKTRYRETIRPKLLKDLGLKNVHQVPKVQKVTLNRGLGEAATNSKALEASLKEMATISGQKALVTRAK

KAIATFKIRQGMPIGCAVTLRGDRMYAFLERFINLALPRIRDFRGVSPKSFDGRGNYTIGVKEQLIFPEI

TFDKVDTIRGMDITIVTSASSDEQGKALLSEMGMPFRKK

>gi|72002348|gb|AAZ58150.1| [SSU ribosomal protein S18P]-alanine acetyltransferase [Prochlorococcus marinus str. NATL2A]

MNLKIIQLGEMHLNDCVDLDQKSSNGLWSKSQWEKELTDPKRICLGIIELQTKKLLGLCSAWLVIDELHI

TFIAVDPRNQRKGIGKFLLSDLIKRSKSLQINHIFLEVKQNNEPAKALYNSMGFKTVGKRSNFYQDGSDA

LLLNKETNNRS

>gi|72003494|gb|AAZ59296.1| LSU ribosomal protein L19P [Prochlorococcus marinus str. NATL2A]

MSVDSKEPSSQEVKIEDESNASEDSEVKTASETKSKGKKISIQNLSPAEIIKTFEDAQLGKDLPDVYVGD

TVRVGVRISEGNKERVQPYEGVVIAKRHGGIHQTITVRRIFQGIGVERIFLVHSPQVASIKVERRGKVRR

AKLFYLRERVGKATRVKQRFDR

>gi|72003003|gb|AAZ58805.1| SSU ribosomal protein S6P [Prochlorococcus marinus str. NATL2A]

MSEQPYYETMYILRPDIPEEEVDSHLKKYSEILEKSETEVLDSQMRGKRRLAYPIAKHKEGIYVQLSHKG

NGQQVATLERAMRLSEDVIRYITVKQDGPLPTPKPTSKENEPEKEEVKPTEEKTESPSKDEKKEDSKE

>gi|72002948|gb|AAZ58750.1| LSU ribosomal protein L20P [Prochlorococcus marinus str. NATL2A]

MARVKRGNVARKRRNKILRLARGFRGGNGTLFRTANQRVMKALCNAYRDRRRRKRDFRRLWIARINAAAR

LNGLSYSKFMGGLKKADIRINRKMLAQLAVIDPKTFTNVAVNSKS

>gi|72002947|gb|AAZ58749.1| LSU ribosomal protein L35P [Prochlorococcus marinus str. NATL2A]

MPKLKTRKAAAKRFKATGTGKFMRRRAFHNHLLDHKSPKLKRHLKTKAVVDERDAENVRLMLPYA

>gi|72002817|gb|AAZ58619.1| LSU ribosomal protein L3P [Prochlorococcus marinus str. NATL2A]

MSLGILGKKLGMSQLFDDQGRAVPVTLIEAGPCRITQLKSADTDGYAAVQIGFQLIREKLINKPSKGHLA

KSGNDLLRHLREYRVENSSEFELGASITVDDFEKGQKVDISGDTMGRGFAGYQKRHGFSRGPMSHGSKNH

RLPGSIGAGTTPGRVYPGKRMAGRMGGKKVTTRALEILKIDTNHNLLVVKGSVPGKPGSLLNIRPAKRVG

APTQQGGK

>gi|72002816|gb|AAZ58618.1| LSU ribosomal protein L4P [Prochlorococcus marinus str. NATL2A]

MTNCTVLDWQGKEAGESSIDLKTAKESSAADLLHRAVLRQQAHSRQGTASTLTRSEVRGGGRKPYKQKGT

GRARQGSIRTPLRPGGGIIFGPKPRKYNLEMNRKERRLALRTALMSRIPDAKIIKDFGSKLEVPKTSEIV

ALLKRVGIDSDVKILIILNKPSEIIKRSIKNLEKVKLISADQLNVFDLLNANSLVIGEDALSTIKEVYGN

D

>gi|72002815|gb|AAZ58617.1| LSU ribosomal protein L23P [Prochlorococcus marinus str. NATL2A]

MTKFFEKRLTDVIKRPLITEKATKALDLNQYTFEVDPRAAKPDIKAAVEKMFDVKVLGISTMNPPRKSRR

VGRFSGKRPQVKKAVVRLAEGNSIQLFPESEEA

>gi|72002814|gb|AAZ58616.1| LSU ribosomal protein L2P [Prochlorococcus marinus str. NATL2A]

MAIRSFKPYTPGTRSRVVTDFSEVTSSKPERSLVVSKHRKKGRNNRGVITCRHRGGGHKRLYRIVDFRRN

KHGVSAKVAAIHYDPHRNARLALLFYSDGEKRYILAPADITVGQEVISGPDAPIETGNALPLSSMPLGSS

VHCVELYPGRGGQMVRTAGASAQVMAKEGDYVALKLPSTEVRLVRKECYATLGEVGNSEIRNTSLGKAGR

RRWLGRRPQVRGSVMNPCDHPHGGGEGKAPVGRAGPVTPWGKAALGLKTRKKNKPSNKYVLRKRRKVSKR

SRGGRDS

>gi|72002813|gb|AAZ58615.1| SSU ribosomal protein S19P [Prochlorococcus marinus str. NATL2A]

MGRSLKKGPFISDSLLRKIEKQNSNDDKAVIKTWSRASTILPLMIGHTIAVHNGKSHIPVFITEQMVGHK

LGEFAPTRTYRGHIRDKKGAR

>gi|72002808|gb|AAZ58610.1| SSU ribosomal protein S17P [Prochlorococcus marinus str. NATL2A]

MALKEMVGTVVSDKMQKTVVVAVENRFPHPIYQKIISRTTRYKAHDAENHCKVGDRVRIKESPPISAHKR

WTVTDVLVKGMKSKEAAK

>gi|72002807|gb|AAZ58609.1| LSU ribosomal protein L14P [Prochlorococcus marinus str. NATL2A]

MIQQETFLTVADNSGAKRLQCIRVLGSNRRYAHVGDVIVASVKDAMPNMGVKKSDVVKAVVVRTRATMRR

ETGNSIRFDDNAAVLINDDQNPRGTRVFGPVARELRERNFTKIVSLAPEVI

>gi|72002806|gb|AAZ58608.1| LSU ribosomal protein L24P [Prochlorococcus marinus str. NATL2A]

MPAINKTKGSADRIKMKIRKGDTVQVISGKDKGKTGEVLKTLPYENRVLVQGINQRTKHVKPSQEGETGR

IETKEFSLHASNVMIYSTKEKVASKVEIFVDKDGSKKRRLKKTGELID

>gi|72002804|gb|AAZ58606.1| SSU ribosomal protein S8P [Prochlorococcus marinus str. NATL2A]

MANHDPISDMLTRIRNASEKRHEKTKVPASRMSLSIAKVLQSEGFIAEINEEGEGFRKQLILGLKYTGKH

RSPIIRSMQRVSKPGLRIYKNTRGLPKVLGGLGIAIISTSNGVMSDRDARKQGVGGEVLCYVC

>gi|72002802|gb|AAZ58604.1| LSU ribosomal protein L18P [Prochlorococcus marinus str. NATL2A]

MSKLSRKQQTQKRHKRLRRNLSGTESRPRLAVFRSNNHIYAQIIDDDAQNTICAASTLDKDLKASLKVNA

GSCDASTAVGELVAKKALSKGIKQVIFDRGGNIYHGRVKALAEAARVAGLNF

>gi|72002800|gb|AAZ58602.1| LSU ribosomal protein L15P [Prochlorococcus marinus str. NATL2A]

MTIKLESLQSNKGSRRKKMRKGRGIAAGQGASCGFGMRGQKSRSGRPTRPGFEGGQMPLYRRVPKLKHFP

IVNQKNFTVLNVSRLNALKDGTIVNLDLLVKEGILTKPKNPLKILGNGKLEVKLTVQAAAFTASAKNKIE

EVGGSCELYN

>gi|72002797|gb|AAZ58599.1| LSU ribosomal protein L36P [Prochlorococcus marinus str. NATL2A]

MKVRSSVKKISPDDQIVRRRGRIYVINKKRPRNKQRQG

>gi|72002796|gb|AAZ58598.1| SSU ribosomal protein S13P [Prochlorococcus marinus str. NATL2A]

MARISGIDIPREKRVEVALTYIYGIGLTRAQSILEKSGVNPDIRVKDLEDSDIQKLRAVTEEFTLEGDLR

RQEGMALKRLQDIGCVRGRRHRMSLPVRGQRTRTNARTRRGARKTVAGRKK

>gi|72002795|gb|AAZ58597.1| SSU ribosomal protein S11P [Prochlorococcus marinus str. NATL2A]

MATTSKKSGSKKSKRNVPNGVVHIQSTFNNTIVSITDTSGEVISWSSAGASGFKGARKGTPFAAQTAAEL

AARRALEQGMRQIEVLVRGPGSGRETAIRALQVAGLEITLIRDVTPLPHNGCRRPKRRRV

>gi|72002793|gb|AAZ58595.1| LSU ribosomal protein L17P [Prochlorococcus marinus str. NATL2A]

MRHQRRIPQLSLPADQRKALLRGLTTQLIREGRVTTTKARAKALRNETERMITLAKDGSLASRRRAIGYV

YDKQLVHALFEKAQERYGDREGGYTRIVRTTPRRGDNSEMAIVELV

>gi|72002791|gb|AAZ58593.1| LSU ribosomal protein L13P [Prochlorococcus marinus str. NATL2A]

MNKTSVPSPDSIDRQWFLVDAENQTLGRLATEVASVLRGKTKPNFTPHLDTGDFVIVVNAEKIKVTGKKS

DQKLYRRHSGRPGGMKVETFKALQSRIPERIVEKAIKGMLPHTRLGRQLFTKLKVYKGSDHPHSAQEPKI

LSLNSESVTK

>gi|72002790|gb|AAZ58592.1| SSU ribosomal protein S9P [Prochlorococcus marinus str. NATL2A]

MTSSTNKVVYWGTGRRKTSVARVRLTPGKGEIIINGRPGDHYLNFNPAYISAVKAPLKTLGLSESYDVLV

NVYGGGLTGQSDAIKQGAARALCTLSLDNRKPLKLEGHLSRDPRAKERRKYGLKKARKAPQFSKR

>gi|72002789|gb|AAZ58591.1| LSU ribosomal protein L31P [Prochlorococcus marinus str. NATL2A]

MPKSDIHPTWYPEAKVICNGEVVMTTGATQPEIQVDVWSGNHPFFTGTQKILDTEGRVDRFMRKYGMASS

DSSEQKDKSSEEKKES

>gi|72002765|gb|AAZ58567.1| SSU ribosomal protein S12P [Prochlorococcus marinus str. NATL2A]

MPTIQQLIRTERKTLKTKTKSPALRGCPERRGVCTRVYTSTPKKPNSALRKVARVRLTSGFEVTAYIGGI

GHNLQEHSVVLLRGGRVKDLPGVRYHIVRGSLDTAGVKDRRQSRSKYGAKSPKE

>gi|72002764|gb|AAZ58566.1| SSU ribosomal protein S7P [Prochlorococcus marinus str. NATL2A]

MSRRNAAEKRPVLPDPQFNNRLASMMVHRLMKHGKKSTAQKILSDAFGLINERTGSDPIELFETAVKNVT

PLVEVRARRVGGATYQVPMEVRQERGIAMALRWLVNFSRSRNGRSMAQKLAGELMDAANEAGNAVRKREE

THKMAEANKAFAHYRY

>gi|72002761|gb|AAZ58563.1| SSU ribosomal protein S10P [Prochlorococcus marinus str. NATL2A]

MSTAIAQQKIRIRLKAFDRRMLDLSCDKIIETADTTAASAIGPIPLPTKRKIYCVLRSPHVDKDSREHFE

TRTHRRIIDIYSPSAKTIDALMKLDLPSGVDIEVKL

>gi|72002748|gb|AAZ58550.1| SSU ribosomal protein S21P [Prochlorococcus marinus str. NATL2A]

MTQVTVGENEGIESALRRFKRQVSKAGIFGELKRLRHHETPVEKYKRKLQQRRRSRRR

>gi|72002706|gb|AAZ58508.1| SSU ribosomal protein S20P [Prochlorococcus marinus str. NATL2A]

MANTNSAKKRIQIAERNRLENKNYKSTVRTLMKRCFVACGIFEKEPGDESKADLQKTFNLAFSKIDKAVK

KGVLHKNTGANQKSRLSVALKKVLKEVV

>gi|72002614|gb|AAZ58416.1| [LSU ribosomal protein L11P]-lysine N-methyltransferase [Prochlorococcus marinus str. NATL2A]

MLVEKTINLQNSQLSWLRLEQEFSFELEDSFYWLLSKLDIHRFSFEHDPNNNLSKTLFIWLPLNEWSVRD

QEILVQSLISLTEPFDLTLPECKWIQVKDEDWSLSWKKNWKPDPVGKSILILPAWLDVPEKFLERKIIRL

DPGSAFGTGSHPSTRLCLEALDNEPPVGQTIADIGCGSGILSLTALKLGAKSTFSVDTDSLSISATKINS

ALNDVPGNLLNVFLGSIEEIEANMPKEKIDLILCNILAPVIKLLGPSFEKIIGHKGKVILSGLLVQQIKE

LQEFFLELGWQVLEIKKKDQWALMVLTLNLS

>gi|72002605|gb|AAZ58407.1| LSU ribosomal protein L27P [Prochlorococcus marinus str. NATL2A]

MAHKKGTGSTRNGRDSNSKRLGVKAYGGEKVTAGSILIRQRGTSVLPGINVGRGKDDTLFALTDGSVHFE

SIRRGLRNRKRINISTAKAV

>gi|72002604|gb|AAZ58406.1| LSU ribosomal protein L21P [Prochlorococcus marinus str. NATL2A]

MADKKSSPKKENPQDKTYAIVEASGKQFWLQPNRYYDFDRCQAEVDDVLTLENVLLLNDGKDLKLGKPYV

KDAKVEIKVLEHRRGPKIIVYKMRPKKKTRRKNGHRQELTRVLVQSISIGSNTKKSKAVKTTASKVESE

>gi|72002541|gb|AAZ58343.1| SSU ribosomal protein S16P [Prochlorococcus marinus str. NATL2A]

MIKLRLKRFGKKRETSFRLVACNSTSRRDGRPLQELGFYNPRTKETRLDTEALRTRLGQGAQPTDAVRTL

LEKGGLLEKKVRPAEVLGKQKQEKERSAKKKDAAASETSE

>gi|72002498|gb|AAZ58300.1| SSU ribosomal protein S14P [Prochlorococcus marinus str. NATL2A]

MAKKSMIARDVKRKKLVERYAAKRKSLIEAFKSAKDPMERLEIHRKIQALPRNCAPNRIRNRCWATGKPR

GVYRDFGLCRNQLRSRAHNGELPGVVKSSW

>gi|72002044|gb|AAZ57846.1| LSU ribosomal protein L32P [Prochlorococcus marinus str. NATL2A]

MAVPKKKTSKGKRNQRHATWKGKAAVAAEKALSIGKSVLTGRAQGFVYPMNETSEEEAD

>gi|72002030|gb|AAZ57832.1| SSU ribosomal protein S18P [Prochlorococcus marinus str. NATL2A]

MTNSLFKQKLSPIKPGDPIDYKDVELLKKFITDRGKILPRRLTGLTAKQQRDLTTAVKRARIIALLPFVN

PEG

>gi|72002029|gb|AAZ57831.1| LSU ribosomal protein L33P [Prochlorococcus marinus str. NATL2A]

MAKKGTRIVVTLECTESRTVPSSKKRSAGVSRYTTEKNRRNTTERLELKKFCPELNKMTIHREIK

>gi|72001995|gb|AAZ57797.1| LSU ribosomal protein L28P [Prochlorococcus marinus str. NATL2A]

MSRVCQLTGTRANNGMSVSHSHIRTKKLQQANLQQRRLWWEEENKWINIRVTTRALKTIQKKGLGKYAKS

LGVDLNKL

>gi|72001957|gb|AAZ57759.1| SSU ribosomal protein S15P [Prochlorococcus marinus str. NATL2A]

MSLGTEEKQNLINTHQVHPTDTGSAEVQVAMLTTRISKLSTHLQGNIHDFSSRQGLLKMIGQRKRLLGYV

RSKSEKRYTELIEKLAIRG

>gi|72001847|gb|AAZ57649.1| SSU ribosomal protein S2P [Prochlorococcus marinus str. NATL2A]

MMEAGAHFGHQTRRWNPKMSRYIYSARNGVHIIDLVKTAVCMNSAYKWTRGAARSGKRFLFVGTKKQASE

VVAQEAIRCGASYVNQRWLGGMLTNWTTMKARIDRLKDLERMESSGAIAMRPKKEGAVLRRELERLQKYL

GGLKGMRRLPDVVVLVDQRRETNAVLEARKLDIPLVSMLDTNCDPDLCEIPIPCNDDAVRSVQLVLGRLA

DAINEGRHGPNE

>gi|72003421|gb|AAZ59223.1| sigma 54 modulation protein / SSU ribosomal protein S30P [Prochlorococcus marinus str. NATL2A]

MKLLIHGRNLELTQSLRDYTKTKIDKATHNFQEMVQEADVHLSVARNPRVPQQTAEVTVFANGTVIRAQE

RSENLYASIDLVANKLARQLRKYKERHNSHNVHHNQSTKSVQNEDTQNFSSSDHSLTEGKEPHLPSPGVR

RKYFEMTPMNIEQARVQLDLIDHDFYLFREEEGSALRVIYKRNHGGYGVIQEKI

>gi|72002810|gb|AAZ58612.1| LSU ribosomal protein L16P [Prochlorococcus marinus str. NATL2A]

MLSPKRTKFRKQQRGRMRGVATRGNKIAFGQFALQAQDCGWVTSRQIEASRRAMTRYVKRGGQIWIRIFP

DKPVTMRPAETRMGSGKGNPEFWVAVVKPGRILFEMGGDEITEAIAKEAMRLAQYKLPVKTKFISLDEDL

NKGNYKPAKTPVTADDSESSS

>gi|72002809|gb|AAZ58611.1| LSU ribosomal protein L29P [Prochlorococcus marinus str. NATL2A]

MSKTTTKDLRNLSDSEMSDKIQNLRKELFDLRFKQATRQLAKTHRFKEARTELAQLLTVSNERSRSNTSS

>gi|72002491|gb|AAZ58293.1| LSU ribosomal protein L34P [Prochlorococcus marinus str. NATL2A]

MTKRTFGGTSRKRKRVSGFRVRMRSHTGRRVVRTRRKRGRSRLTV

>gi|72003033|gb|AAZ58835.1| GTPase EngC [Prochlorococcus marinus str. NATL2A]

MNKNKSNKLKGIVVALKANFLIVEIDHKNFKDHSFDQFYGKIRLLCIRRSKLNYQGLFIDVGDIVCVESI

DYKNKRAVVSDVEPRQSFLKRPAVANVTLVSICISADEPLFDMEQTSRFLLTAECANIEPLIILTKIDLI

TKNDLILYINKFKSWGYDCIPVSIHNSQGIDSLIERLRKTKLTVLAGPSGVGKTSLINHLIPTVSLPTSS

VSKKLKRGTHTTRHVELFAIGNGSLLADTPGFNRPEIVCEPSDFAFLFPEFRTQLSNSQCKFRNCLHRDE

PGCVIDKDLERYPFYRQNLEEMINSPLPYQAG

>gi|72002499|gb|AAZ58301.1| RNA binding S1:KH [Prochlorococcus marinus str. NATL2A]

MQGQTKSVSFDGREIKLTTGRFAPQAGGSVMIECGDTSVLVTATKSSGREGVDFLPLMCDYEERLYAAGR

IPGSFMRREGRPPERATLISRLIDRPMRPLFPGWMRDDIQIVATCLSLDERVPADVLAVTGASMATLMAG

IPFQGPMAAVRVGLLGDDFVLNPSYREIERGDLDLVVAGTPDGVVMVEAGANQLSEQDVIEAIDFGYEAI

TELINAQKEVLKESGIKQEMPKAPEIDDTISTYLDKNCTKSISEVLKNFDQTKEERDNKIEEIKISISAK

IDGLKDDNAVKKSLSLNNKLLENSYKALTKKLMREQIIKEGKRVDGRELNEVRAIDADAAVLPNRVHGSA

LFQRGLTQVLSTATLGTPSDAQEMDDLNPNTDKTYIHHYNFPPYSVGETRPMRTPGRREVGHGALAERAL

IPVLPAKDTFPYVLRVVSEVLSSNGSTSMASVCGSTLALMDAGVPLKAPVGGAAMGLIKEGKEVRILTDI

QGIEDFLGDMDFKVAGTEKGITALQMDMKITGLPIETIGEAINQALPARTHILGKMLDAIETPKDNLSPH

APRLLSFRIDPELIGTVIGPGGRTIKGITERTNTKIDIEDGGIVTIASHDGAAAEEAQRIIEGLTRKVHE

GEIFPGSITRIIPIGAFVEILPGKEGMIHISQLSEARVEKVEDVVKVGDQVTVRVREIDNRGRINLTLRG

VSQNGGMSNYPEPTPTPVAPLT

>gi|311812668|emb|CBX83672.1| unnamed protein product [Prochlorococcus marinus str. NATL2A]

MQGQTKSVSFDGREIKLTTGRFAPQAGGSVMIECGDTSVLVTATKSSGREGVDFLPLMCDYEERLYAAGR

IPGSFMRREGRPPERATLISRLIDRPMRPLFPGWMRDDIQIVATCLSLDERVPADVLAVTGASMATLMAG

IPFQGPMAAVRVGLLGDDFVLNPSYREIERGDLDLVVAGTPDGVVMVEAGANQLSEQDVIEAIDFGYEAI

TELINAQKEVLKESGIKQEMPKAPEIDDTISTYLDKNCTKSISEVLKNFDQTKEERDNKIEEIKISISAK

IDGLKDDNAVKKSLSLNNKLLENSYKALTKKLMREQIIKEGKRVDGRELNEVRAIDADAAVLPNRVHGSA

LFQRGLTQVLSTATLGTPSDAQEMDDLNPNTDKTYIHHYNFPPYSVGETRPMRTPGRREVGHGALAERAL

IPVLPAKDTFPYVLRVVSEVLSSNGSTSMASVCGSTLALMDAGVPLKAPVGGAAMGLIKEGKEVRILTDI

QGIEDFLGDMDFKVAGTEKGITALQMDMKITGLPIETIGEAINQALPARTHILGKMLDAIETPKDNLSPH

APRLLSFRIDPELIGTVIGPGGRTIKGITERTNTKIDIEDGGIVTIASHDGAAAEEAQRIIEGLTRKVHE

GEIFPGSITRIIPIGAFVEILPGKEGMIHISQLSEARVEKVEDVVKVGDQVTVRVREIDNRGRINLTLRG

VSQNGGMSNYPEPTPTPVAPLT

>gi|207008610|emb|CAR80214.1| unnamed protein product [Prochlorococcus marinus str. NATL2A]

MQGQTKSVSFDGREIKLTTGRFAPQAGGSVMIECGDTSVLVTATKSSGREGVDFLPLMCDYEERLYAAGR

IPGSFMRREGRPPERATLISRLIDRPMRPLFPGWMRDDIQIVATCLSLDERVPADVLAVTGASMATLMAG

IPFQGPMAAVRVGLLGDDFVLNPSYREIERGDLDLVVAGTPDGVVMVEAGANQLSEQDVIEAIDFGYEAI

TELINAQKEVLKESGIKQEMPKAPEIDDTISTYLDKNCTKSISEVLKNFDQTKEERDNKIEEIKISISAK

IDGLKDDNAVKKSLSLNNKLLENSYKALTKKLMREQIIKEGKRVDGRELNEVRAIDADAAVLPNRVHGSA

LFQRGLTQVLSTATLGTPSDAQEMDDLNPNTDKTYIHHYNFPPYSVGETRPMRTPGRREVGHGALAERAL

IPVLPAKDTFPYVLRVVSEVLSSNGSTSMASVCGSTLALMDAGVPLKAPVGGAAMGLIKEGKEVRILTDI

QGIEDFLGDMDFKVAGTEKGITALQMDMKITGLPIETIGEAINQALPARTHILGKMLDAIETPKDNLSPH

APRLLSFRIDPELIGTVIGPGGRTIKGITERTNTKIDIEDGGIVTIASHDGAAAEEAQRIIEGLTRKVHE

GEIFPGSITRIIPIGAFVEILPGKEGMIHISQLSEARVEKVEDVVKVGDQVTVRVREIDNRGRINLTLRG

VSQNGGMSNYPEPTPTPVAPLT

>gi|97181835|sp|Q46HH1.1|RL10_PROMT RecName: Full=50S ribosomal protein L10

MGRTLESKKQIVKKIEDLLDNSEMALVLDYKGLSTKEMSDLRSRLQQSDGVCKVTKNTLMRQAIKGKNSW

TGLDSLLTGTNAFVLIKGDVGSAVKAVQAFQKETQKSETKGGLFEGKLLSQDEIKAIAKLPSKEALMGQI

AGALNSITSKIAIGINEVPSGLARSLKQHSENGES

>gi|118597274|sp|Q46HG9.1|RL11_PROMT RecName: Full=50S ribosomal protein L11

MAKKVVALIKLALQAGKANPAPPVGPALGQHGVNIMAFCKEYNSRTQDKAGFVIPVEISVFEDRSFSFIT

KTPPASVLITKAAGIAKGSGESAKGSAGSISTSQLEEIAKTKLPDLNCSSIESAMKVIEGTAKNMGVSIK

D

>gi|118573634|sp|Q46IS3.1|RL6_PROMT RecName: Full=50S ribosomal protein L6

MSRTGKKPISLPEKVDVKFEGLSITVKGPKGELKRTLPNGVSLSKDENFIFVKPINEKRQSREMHGLCRS

LVANMVEGVSNGFTKKLEIVGVGSRAQVKGKTLVVSAGYSHPVEVVPPEGITFKVENNTNVIVTGPDKEL

VGNEAAKIRAIRPPEPYKGKGIKYQGELIIRKAGKSGKT

>gi|115305542|sp|Q46IB6.1|RL9_PROMT RecName: Full=50S ribosomal protein L9

MAKRVQVVLNEDIKSLGNDGDLVEVAPGFARNFLLPNKKALPVTPTVLKQVEHRRAKQAEKEAAKKQEAI

DFQTALTTIGRFTVKKQVGEDGVLFGTVTNGDVAEVVKEATKKDIDRRDISIPEIHGVGKYKVQIKLHNE

VNAEINLEVTSY

>gi|91207877|sp|Q46IS5.1|RS5_PROMT RecName: Full=30S ribosomal protein S5

MTDSNNQSPNKKTSGSSSAPPAADGRQENRRSRGEKRGGRRDRRGQERDSEWQERVVQIRRVSKTVKGGK

KMSFRAIVVVGNEKGQVGVGVGKAGDVIGAVRKGVADGKKHLVRVPLTRNSSIPTLSNGRDGAASVLIRP

AAPGTGVIAGGSIRTVLELAGIKNVLAKRLGSKTPLNNARAAMVALSELRTHKATAKERGISLEQIYS

>gi|109893782|sp|Q46HH2.1|RL7_PROMT RecName: Full=50S ribosomal protein L7/L12

MSAKTDEILDSLKSLSLLEASELVKQIEEAFGVSAAASAGVVMAAPGAAAGGDGADAAEEKTEFEVVLES

FEASSKIKVLKEVRNATGLGLGEAKALVEAAPKTIKEGATKEDAEALKKAIEAVGGKVTLK

>gi|109893708|sp|Q46IS1.1|RL5_PROMT RecName: Full=50S ribosomal protein L5

MSLKTRYRETIRPKLLKDLGLKNVHQVPKVQKVTLNRGLGEAATNSKALEASLKEMATISGQKALVTRAK

KAIATFKIRQGMPIGCAVTLRGDRMYAFLERFINLALPRIRDFRGVSPKSFDGRGNYTIGVKEQLIFPEI

TFDKVDTIRGMDITIVTSASSDEQGKALLSEMGMPFRKK

>gi|109893219|sp|Q46IR4.1|RL22_PROMT RecName: Full=50S ribosomal protein L22

MTTSSTKTTAQAHGRYIRGSASKVRRVLDQIRGRTYRDALIMLEFMPYRSTEPITKVLRSAVANAENNLG

LDPSSLMISTATADMGPPMKRYRPRAQGRAFAIKKQTCHISISVSATQSTNSEDSD

>gi|91207829|sp|Q46IR5.1|RS3_PROMT RecName: Full=30S ribosomal protein S3

MGHKIHPTGIRLGITQEHRSKWYAPSKTYPLLLQEDDRIRTFIKKKYLAAGISDVLIARKADQLEVELKT

ARPGVIVGRQGSGIEELRSGIQKTIGDRNRQVRINVVEVEKVDADAYLLAEYIAQQLEKRVAFRRTIRMA

VQRAQRAGVLGLKIQVGGRLNGAEIARSEWTREGRVPLHTLRAEIDYATKVASTTYGVLGIKVWVFKGEV

LPKEEQPLPVGSSPRRSRGNRRPQQFEDRSNEGK

>gi|91207368|sp|Q46HH0.1|RL1_PROMT RecName: Full=50S ribosomal protein L1

MKNFSKRMTTLLSKVEERSYSPIEAIKLVKENANAKFDETIEAHIRLGIDPKYTDQQLRTTVALPSGTGQ

KIRIAVVTRGEKVNEATKAGADLAGEEDLVDSINKGEMNFDLLISTPDMMPKVAKLGRVLGPRGLMPNPK

AGTVTTDLEGAIKEFKAGKLEFRADKAGIVHVRFGKASFSEEALLENLKTLQTTIEKNKPSGAKGKFWRS

FFITSTMGPSVEVDINELQDLQKEK

>gi|90101705|sp|Q46LZ9.1|RS4_PROMT RecName: Full=30S ribosomal protein S4

MSRYRGPRLRITRRLGDLPGLTRKAAKRSHPPGQHGQARRKRSEYAIRLEEKQKLRFNYGISERQLVRYV

KKARAQEGSTGTNLLKLLENRLDNVCFRLGFGPTIPGSRQLVNHGHVTVNGRITDIASYQCKAGDVIAIR

DNKASKQLAQANLEFPGLANVPPHLELDKTKLSAKISAKTDREWVAIEINELLVVEYYSRKV

>gi|123620756|sp|Q46IT5.1|RL13_PROMT RecName: Full=50S ribosomal protein L13

MNKTSVPSPDSIDRQWFLVDAENQTLGRLATEVASVLRGKTKPNFTPHLDTGDFVIVVNAEKIKVTGKKS

DQKLYRRHSGRPGGMKVETFKALQSRIPERIVEKAIKGMLPHTRLGRQLFTKLKVYKGSDHPHSAQEPKI

LSLNSESVTK

>gi|123620754|sp|Q46IS9.1|RL36_PROMT RecName: Full=50S ribosomal protein L36

MKVRSSVKKISPDDQIVRRRGRIYVINKKRPRNKQRQG

>gi|148887092|sp|Q46IC9.1|RL35_PROMT RecName: Full=50S ribosomal protein L35

MPKLKTRKAAAKRFKATGTGKFMRRRAFHNHLLDHKSPKLKRHLKTKAVVDERDAENVRLMLPYA

>gi|148878609|sp|Q46IT7.1|RL31_PROMT RecName: Full=50S ribosomal protein L31

MPKSDIHPTWYPEAKVICNGEVVMTTGATQPEIQVDVWSGNHPFFTGTQKILDTEGRVDRFMRKYGMASS

DSSEQKDKSSEEKKES

>gi|123773762|sp|Q46IR9.1|RL14_PROMT RecName: Full=50S ribosomal protein L14

MIQQETFLTVADNSGAKRLQCIRVLGSNRRYAHVGDVIVASVKDAMPNMGVKKSDVVKAVVVRTRATMRR

ETGNSIRFDDNAAVLINDDQNPRGTRVFGPVARELRERNFTKIVSLAPEVI

>gi|123759898|sp|Q46KZ6.1|RS18_PROMT RecName: Full=30S ribosomal protein S18

MTNSLFKQKLSPIKPGDPIDYKDVELLKKFITDRGKILPRRLTGLTAKQQRDLTTAVKRARIIALLPFVN

PEG

>gi|123759852|sp|Q46IR1.1|RL23_PROMT RecName: Full=50S ribosomal protein L23

MTKFFEKRLTDVIKRPLITEKATKALDLNQYTFEVDPRAAKPDIKAAVEKMFDVKVLGISTMNPPRKSRR

VGRFSGKRPQVKKAVVRLAEGNSIQLFPESEEA

>gi|123621283|sp|Q46L31.1|RL28_PROMT RecName: Full=50S ribosomal protein L28

MSRVCQLTGTRANNGMSVSHSHIRTKKLQQANLQQRRLWWEEENKWINIRVTTRALKTIQKKGLGKYAKS

LGVDLNKL

>gi|123621264|sp|Q46KZ7.1|RL33_PROMT RecName: Full=50S ribosomal protein L33

MAKKGTRIVVTLECTESRTVPSSKKRSAGVSRYTTEKNRRNTTERLELKKFCPELNKMTIHREIK

>gi|123620932|sp|Q46JM8.1|RS14_PROMT RecName: Full=30S ribosomal protein S14

MAKKSMIARDVKRKKLVERYAAKRKSLIEAFKSAKDPMERLEIHRKIQALPRNCAPNRIRNRCWATGKPR

GVYRDFGLCRNQLRSRAHNGELPGVVKSSW

>gi|123620867|sp|Q46JC1.1|RL27_PROMT RecName: Full=50S ribosomal protein L27

MAHKKGTGSTRNGRDSNSKRLGVKAYGGEKVTAGSILIRQRGTSVLPGINVGRGKDDTLFALTDGSVHFE

SIRRGLRNRKRINISTAKAV

>gi|123620755|sp|Q46IT3.1|RL17_PROMT RecName: Full=50S ribosomal protein L17

MRHQRRIPQLSLPADQRKALLRGLTTQLIREGRVTTTKARAKALRNETERMITLAKDGSLASRRRAIGYV

YDKQLVHALFEKAQERYGDREGGYTRIVRTTPRRGDNSEMAIVELV

>gi|119367289|sp|Q46IX8.1|RS21_PROMT RecName: Full=30S ribosomal protein S21

MTQVTVGENEGIESALRRFKRQVSKAGIFGELKRLRHHETPVEKYKRKLQQRRRSRRR

>gi|119367140|sp|Q46IR3.1|RS19_PROMT RecName: Full=30S ribosomal protein S19

MGRSLKKGPFISDSLLRKIEKQNSNDDKAVIKTWSRASTILPLMIGHTIAVHNGKSHIPVFITEQMVGHK

LGEFAPTRTYRGHIRDKKGAR

>gi|119365970|sp|Q46JC2.1|RL21_PROMT RecName: Full=50S ribosomal protein L21

MADKKSSPKKENPQDKTYAIVEASGKQFWLQPNRYYDFDRCQAEVDDVLTLENVLLLNDGKDLKLGKPYV

KDAKVEIKVLEHRRGPKIIVYKMRPKKKTRRKNGHRQELTRVLVQSISIGSNTKKSKAVKTTASKVESE

>gi|116256023|sp|Q46GU7.1|RL19_PROMT RecName: Full=50S ribosomal protein L19

MSVDSKEPSSQEVKIEDESNASEDSEVKTASETKSKGKKISIQNLSPAEIIKTFEDAQLGKDLPDVYVGD

TVRVGVRISEGNKERVQPYEGVVIAKRHGGIHQTITVRRIFQGIGVERIFLVHSPQVASIKVERRGKVRR

AKLFYLRERVGKATRVKQRFDR

>gi|115504929|sp|Q46IS4.1|RL18_PROMT RecName: Full=50S ribosomal protein L18

MSKLSRKQQTQKRHKRLRRNLSGTESRPRLAVFRSNNHIYAQIIDDDAQNTICAASTLDKDLKASLKVNA

GSCDASTAVGELVAKKALSKGIKQVIFDRGGNIYHGRVKALAEAARVAGLNF

>gi|115502685|sp|Q46IS6.1|RL15_PROMT RecName: Full=50S ribosomal protein L15

MTIKLESLQSNKGSRRKKMRKGRGIAAGQGASCGFGMRGQKSRSGRPTRPGFEGGQMPLYRRVPKLKHFP

IVNQKNFTVLNVSRLNALKDGTIVNLDLLVKEGILTKPKNPLKILGNGKLEVKLTVQAAAFTASAKNKIE

EVGGSCELYN

>gi|115305726|sp|Q46IS2.1|RS8_PROMT RecName: Full=30S ribosomal protein S8

MANHDPISDMLTRIRNASEKRHEKTKVPASRMSLSIAKVLQSEGFIAEINEEGEGFRKQLILGLKYTGKH

RSPIIRSMQRVSKPGLRIYKNTRGLPKVLGGLGIAIISTSNGVMSDRDARKQGVGGEVLCYVC

>gi|115305713|sp|Q46IW2.1|RS7_PROMT RecName: Full=30S ribosomal protein S7

MSRRNAAEKRPVLPDPQFNNRLASMMVHRLMKHGKKSTAQKILSDAFGLINERTGSDPIELFETAVKNVT

PLVEVRARRVGGATYQVPMEVRQERGIAMALRWLVNFSRSRNGRSMAQKLAGELMDAANEAGNAVRKREE

THKMAEANKAFAHYRY

>gi|115305699|sp|Q46J20.1|RS20_PROMT RecName: Full=30S ribosomal protein S20

MANTNSAKKRIQIAERNRLENKNYKSTVRTLMKRCFVACGIFEKEPGDESKADLQKTFNLAFSKIDKAVK

KGVLHKNTGANQKSRLSVALKKVLKEVV

>gi|115305644|sp|Q46IW5.1|RS10_PROMT RecName: Full=30S ribosomal protein S10

MSTAIAQQKIRIRLKAFDRRMLDLSCDKIIETADTTAASAIGPIPLPTKRKIYCVLRSPHVDKDSREHFE

TRTHRRIIDIYSPSAKTIDALMKLDLPSGVDIEVKL

>gi|115305507|sp|Q46KY2.1|RL32_PROMT RecName: Full=50S ribosomal protein L32

MAVPKKKTSKGKRNQRHATWKGKAAVAAEKALSIGKSVLTGRAQGFVYPMNETSEEEAD

>gi|115305491|sp|Q46IR2.1|RL2_PROMT RecName: Full=50S ribosomal protein L2

MAIRSFKPYTPGTRSRVVTDFSEVTSSKPERSLVVSKHRKKGRNNRGVITCRHRGGGHKRLYRIVDFRRN

KHGVSAKVAAIHYDPHRNARLALLFYSDGEKRYILAPADITVGQEVISGPDAPIETGNALPLSSMPLGSS

VHCVELYPGRGGQMVRTAGASAQVMAKEGDYVALKLPSTEVRLVRKECYATLGEVGNSEIRNTSLGKAGR

RRWLGRRPQVRGSVMNPCDHPHGGGEGKAPVGRAGPVTPWGKAALGLKTRKKNKPSNKYVLRKRRKVSKR

SRGGRDS

>gi|109894783|sp|Q46JI5.1|RS16_PROMT RecName: Full=30S ribosomal protein S16

MIKLRLKRFGKKRETSFRLVACNSTSRRDGRPLQELGFYNPRTKETRLDTEALRTRLGQGAQPTDAVRTL

LEKGGLLEKKVRPAEVLGKQKQEKERSAKKKDAAASETSE

>gi|109893619|sp|Q46IR0.1|RL4_PROMT RecName: Full=50S ribosomal protein L4

MTNCTVLDWQGKEAGESSIDLKTAKESSAADLLHRAVLRQQAHSRQGTASTLTRSEVRGGGRKPYKQKGT

GRARQGSIRTPLRPGGGIIFGPKPRKYNLEMNRKERRLALRTALMSRIPDAKIIKDFGSKLEVPKTSEIV

ALLKRVGIDSDVKILIILNKPSEIIKRSIKNLEKVKLISADQLNVFDLLNANSLVIGEDALSTIKEVYGN

D

>gi|109893537|sp|Q46IQ9.1|RL3_PROMT RecName: Full=50S ribosomal protein L3

MSLGILGKKLGMSQLFDDQGRAVPVTLIEAGPCRITQLKSADTDGYAAVQIGFQLIREKLINKPSKGHLA

KSGNDLLRHLREYRVENSSEFELGASITVDDFEKGQKVDISGDTMGRGFAGYQKRHGFSRGPMSHGSKNH

RLPGSIGAGTTPGRVYPGKRMAGRMGGKKVTTRALEILKIDTNHNLLVVKGSVPGKPGSLLNIRPAKRVG

APTQQGGK

>gi|109893306|sp|Q46IS0.1|RL24_PROMT RecName: Full=50S ribosomal protein L24

MPAINKTKGSADRIKMKIRKGDTVQVISGKDKGKTGEVLKTLPYENRVLVQGINQRTKHVKPSQEGETGR

IETKEFSLHASNVMIYSTKEKVASKVEIFVDKDGSKKRRLKKTGELID

>gi|109893134|sp|Q46IC8.1|RL20_PROMT RecName: Full=50S ribosomal protein L20

MARVKRGNVARKRRNKILRLARGFRGGNGTLFRTANQRVMKALCNAYRDRRRRKRDFRRLWIARINAAAR

LNGLSYSKFMGGLKKADIRINRKMLAQLAVIDPKTFTNVAVNSKS

>gi|94730533|sp|Q46IR8.1|RS17_PROMT RecName: Full=30S ribosomal protein S17

MALKEMVGTVVSDKMQKTVVVAVENRFPHPIYQKIISRTTRYKAHDAENHCKVGDRVRIKESPPISAHKR

WTVTDVLVKGMKSKEAAK

>gi|91207930|sp|Q46I73.1|RS6_PROMT RecName: Full=30S ribosomal protein S6

MSEQPYYETMYILRPDIPEEEVDSHLKKYSEILEKSETEVLDSQMRGKRRLAYPIAKHKEGIYVQLSHKG

NGQQVATLERAMRLSEDVIRYITVKQDGPLPTPKPTSKENEPEKEEVKPTEEKTESPSKDEKKEDSKE

>gi|91207715|sp|Q46IT0.1|RS13_PROMT RecName: Full=30S ribosomal protein S13

MARISGIDIPREKRVEVALTYIYGIGLTRAQSILEKSGVNPDIRVKDLEDSDIQKLRAVTEEFTLEGDLR

RQEGMALKRLQDIGCVRGRRHRMSLPVRGQRTRTNARTRRGARKTVAGRKK

>gi|91207668|sp|Q46IT1.1|RS11_PROMT RecName: Full=30S ribosomal protein S11

MATTSKKSGSKKSKRNVPNGVVHIQSTFNNTIVSITDTSGEVISWSSAGASGFKGARKGTPFAAQTAAEL

AARRALEQGMRQIEVLVRGPGSGRETAIRALQVAGLEITLIRDVTPLPHNGCRRPKRRRV

>gi|90111791|sp|Q46IW1.1|RS12_PROMT RecName: Full=30S ribosomal protein S12

MPTIQQLIRTERKTLKTKTKSPALRGCPERRGVCTRVYTSTPKKPNSALRKVARVRLTSGFEVTAYIGGI

GHNLQEHSVVLLRGGRVKDLPGVRYHIVRGSLDTAGVKDRRQSRSKYGAKSPKE

>gi|85701251|sp|Q46L69.1|RS15_PROMT RecName: Full=30S ribosomal protein S15

MSLGTEEKQNLINTHQVHPTDTGSAEVQVAMLTTRISKLSTHLQGNIHDFSSRQGLLKMIGQRKRLLGYV

RSKSEKRYTELIEKLAIRG

>gi|209573696|sp|Q46LH9.2|RS2_PROMT RecName: Full=30S ribosomal protein S2

MAVVSLSEMMEAGAHFGHQTRRWNPKMSRYIYSARNGVHIIDLVKTAVCMNSAYKWTRGAARSGKRFLFV

GTKKQASEVVAQEAIRCGASYVNQRWLGGMLTNWTTMKARIDRLKDLERMESSGAIAMRPKKEGAVLRRE

LERLQKYLGGLKGMRRLPDVVVLVDQRRETNAVLEARKLDIPLVSMLDTNCDPDLCEIPIPCNDDAVRSV

QLVLGRLADAINEGRHGPNE

>gi|123620937|sp|Q46JN5.1|RL34_PROMT RecName: Full=50S ribosomal protein L34

MTKRTFGGTSRKRKRVSGFRVRMRSHTGRRVVRTRRKRGRSRLTV

>gi|123620751|sp|Q46IR7.1|RL29_PROMT RecName: Full=50S ribosomal protein L29

MSKTTTKDLRNLSDSEMSDKIQNLRKELFDLRFKQATRQLAKTHRFKEARTELAQLLTVSNERSRSNTSS

>gi|85541815|sp|Q46IR6.1|RL16_PROMT RecName: Full=50S ribosomal protein L16

MLSPKRTKFRKQQRGRMRGVATRGNKIAFGQFALQAQDCGWVTSRQIEASRRAMTRYVKRGGQIWIRIFP

DKPVTMRPAETRMGSGKGNPEFWVAVVKPGRILFEMGGDEITEAIAKEAMRLAQYKLPVKTKFISLDEDL

NKGNYKPAKTPVTADDSESSS

>gi|109893031|sp|Q46IK7.1|RIMM_PROMT RecName: Full=Ribosome maturation factor RimM

MFEKDKWMTIGEIVAPQGLRGDLRIKPSSDFPERFTKPGKRWIQKTDELPTEIKLTKGKLIPGKSIYVLS

IEGVSTRSSAEEIIGWKLVIPIDSRPMLSKDEYHYHDLIGLEARRGPSKALIGYVTDLIKGGNDLLEIEL

VEGKKVLVPFVKEIVPEIEIKEKWLLINPPPGLLEL

>gi|123773738|sp|Q46I43.1|RSGA_PROMT RecName: Full=Putative ribosome biogenesis GTPase RsgA

MNKNKSNKLKGIVVALKANFLIVEIDHKNFKDHSFDQFYGKIRLLCIRRSKLNYQGLFIDVGDIVCVESI

DYKNKRAVVSDVEPRQSFLKRPAVANVTLVSICISADEPLFDMEQTSRFLLTAECANIEPLIILTKIDLI

TKNDLILYINKFKSWGYDCIPVSIHNSQGIDSLIERLRKTKLTVLAGPSGVGKTSLINHLIPTVSLPTSS

VSKKLKRGTHTTRHVELFAIGNGSLLADTPGFNRPEIVCEPSDFAFLFPEFRTQLSNSQCKFRNCLHRDE

PGCVIDKDLERYPFYRQNLEEMINSPLPYQAG

>gi|123620931|sp|Q46JM7.1|PNP_PROMT RecName: Full=Polyribonucleotide nucleotidyltransferase; AltName: Full=Polynucleotide phosphorylase; Short=PNPase

MQGQTKSVSFDGREIKLTTGRFAPQAGGSVMIECGDTSVLVTATKSSGREGVDFLPLMCDYEERLYAAGR

IPGSFMRREGRPPERATLISRLIDRPMRPLFPGWMRDDIQIVATCLSLDERVPADVLAVTGASMATLMAG

IPFQGPMAAVRVGLLGDDFVLNPSYREIERGDLDLVVAGTPDGVVMVEAGANQLSEQDVIEAIDFGYEAI

TELINAQKEVLKESGIKQEMPKAPEIDDTISTYLDKNCTKSISEVLKNFDQTKEERDNKIEEIKISISAK

IDGLKDDNAVKKSLSLNNKLLENSYKALTKKLMREQIIKEGKRVDGRELNEVRAIDADAAVLPNRVHGSA

LFQRGLTQVLSTATLGTPSDAQEMDDLNPNTDKTYIHHYNFPPYSVGETRPMRTPGRREVGHGALAERAL

IPVLPAKDTFPYVLRVVSEVLSSNGSTSMASVCGSTLALMDAGVPLKAPVGGAAMGLIKEGKEVRILTDI

QGIEDFLGDMDFKVAGTEKGITALQMDMKITGLPIETIGEAINQALPARTHILGKMLDAIETPKDNLSPH

APRLLSFRIDPELIGTVIGPGGRTIKGITERTNTKIDIEDGGIVTIASHDGAAAEEAQRIIEGLTRKVHE

GEIFPGSITRIIPIGAFVEILPGKEGMIHISQLSEARVEKVEDVVKVGDQVTVRVREIDNRGRINLTLRG

VSQNGGMSNYPEPTPTPVAPLT

>gi|33239981|ref|NP_874923.1| Ribosomal protein S1 [Prochlorococcus marinus subsp. marinus str. CCMP1375]

MAGSGNPQPKRPNPPRQEQSDLRKPLQVMHISRKEERQDIDNLKKVEESRQKSSTISKGAKQPLSPNTSK

PSENIAQNNQTAFDQEYEEGLSMGDLLAQERDSSKNNSTPFESDGFLERSVDDFDFDEGEFLAALDENEP

VGNTGETAKGSVIGVESDGVYVDIGGKAPGFMPKSECGLGVITNLKERFPKGLEVEVLVTREQNADGMVT

ISCRALALRKSWDKVIQLAKEAKVVEVKLNGFNRGGVTCDLEGLRGFIPRSQLNQGDDHESLVGKTINVA

FIEVSPDNRKLILSEKKAATAAKFAELEVGQLIEGKVISVKPYGFFVDLGGISGLLHQSMITNGSLRSLR

EVFNQGDNVKALITDLDPARGRIGLNTALLEGLPGELLIDKEKVLAEAEDRAKKAQGLFKPKEEEVK

>gi|33237507|gb|AAP99575.1| Ribosomal protein S1 [Prochlorococcus marinus subsp. marinus str. CCMP1375]

MAGSGNPQPKRPNPPRQEQSDLRKPLQVMHISRKEERQDIDNLKKVEESRQKSSTISKGAKQPLSPNTSK

PSENIAQNNQTAFDQEYEEGLSMGDLLAQERDSSKNNSTPFESDGFLERSVDDFDFDEGEFLAALDENEP

VGNTGETAKGSVIGVESDGVYVDIGGKAPGFMPKSECGLGVITNLKERFPKGLEVEVLVTREQNADGMVT

ISCRALALRKSWDKVIQLAKEAKVVEVKLNGFNRGGVTCDLEGLRGFIPRSQLNQGDDHESLVGKTINVA

FIEVSPDNRKLILSEKKAATAAKFAELEVGQLIEGKVISVKPYGFFVDLGGISGLLHQSMITNGSLRSLR

EVFNQGDNVKALITDLDPARGRIGLNTALLEGLPGELLIDKEKVLAEAEDRAKKAQGLFKPKEEEVK

>gi|33239803|ref|NP_874745.1| Ribosomal protein S1 [Prochlorococcus marinus subsp. marinus str. CCMP1375]

MSENSSAPVTELEESNADSQITSPENEQNADLADQAATHEESFNTEEIPSADDPSSRVKKYDFDGAGFSL

EEFDSLLGKYDYNFKPGDIVNGTVFALETKGAMIDIGAKTAAFMPMQEVSINRVEGLSDVLQPSEIRQFF

IMSEENEDGQLSLSIRRIEYQRAWERVRQLQKEDATIYSEVFATNRGGALVRVEGLRGFIPGSHISTRKA

KEELVAEYLPLKFLEVDEERNRLVLSHRRALVERKMNRLEVGEVVVGAVRGIKPYGAFIDIGGVSGLLHI

SEISHEHIETPHSVLNVNDQMKVMIIDLDAERGRISLSTKALEPEPGDMLTDPQKVFDKAEEMAAKYKEM

LLEQAEEGENPITSMEV

>gi|33237329|gb|AAP99397.1| Ribosomal protein S1 [Prochlorococcus marinus subsp. marinus str. CCMP1375]

MSENSSAPVTELEESNADSQITSPENEQNADLADQAATHEESFNTEEIPSADDPSSRVKKYDFDGAGFSL

EEFDSLLGKYDYNFKPGDIVNGTVFALETKGAMIDIGAKTAAFMPMQEVSINRVEGLSDVLQPSEIRQFF

IMSEENEDGQLSLSIRRIEYQRAWERVRQLQKEDATIYSEVFATNRGGALVRVEGLRGFIPGSHISTRKA

KEELVAEYLPLKFLEVDEERNRLVLSHRRALVERKMNRLEVGEVVVGAVRGIKPYGAFIDIGGVSGLLHI

SEISHEHIETPHSVLNVNDQMKVMIIDLDAERGRISLSTKALEPEPGDMLTDPQKVFDKAEEMAAKYKEM

LLEQAEEGENPITSMEV

>gi|33241283|ref|NP_876225.1| Ribosomal protein L9 [Prochlorococcus marinus subsp. marinus str. CCMP1375]

MAKRVKVVLKEDILSLGKDGDVVEVAPGYARNFLLSQQKALAVTPSVLKQVEYRLAKKAELEAAKKQEAI

DFETALKTIGRFSIKKQTGEDGVLFGTVTNGDVSEAIQLATQKEIDRRNIIVPEIHETGKYKVQVKLHSE

VTAEINLEVIGN

>gi|33241147|ref|NP_876089.1| Ribosomal protein L6P/L9E [Prochlorococcus marinus subsp. marinus str. CCMP1375]

MSRIGKKPIPVPEKVAVTLDGLLVTVKGPKGELTRTLPEGVTIDQTDGLIIVSADSEKRKSRERHGLSRT

LVANMIEGVNNGYSKQLEIVGVGSRAQVKGKTLVVSAGYSHPVEVIPPEGITFKVENNTNVLVSGIDKEL

VGNEAAKIRAIRPPEPYKGKGIKYLGERILRKAGKSGKK

>gi|33241145|ref|NP_876087.1| Ribosomal protein S5 [Prochlorococcus marinus subsp. marinus str. CCMP1375]

MTETKAKSKSKENSSSPVPAAAEGQQQEQKRGNSRGGERRGRRSDRRNQDRDSEWQERVIQIRRVSKTVK

GGKKMSFRAIVVVGNEKGQVGVGVGKAGDVIGAVRKGVADGKKNLVRVPLTPNSSIPTLSNGRDGAASVL

IRPAAPGTGVIAGGSIRTVLELAGIKNVLAKRLGSKTPLNNARAAMVALSLLRTHKATAKERGISLEQIY

S

>gi|33239682|ref|NP_874624.1| Ribosomal protein L11 [Prochlorococcus marinus subsp. marinus str. CCMP1375]

MAKKITAVIKLALQAGKANPAPPVGPALGQHGVNIMAFCKEYNARTQDKAGFVIPVEISVFEDRSFTFIT

KTPPASVLITKAAGIAKGSGDSAKGQAGSINRAQLEEIAKTKLPDLNCNNIESAMKVIAGTARNMGVSVS

D

>gi|33239680|ref|NP_874622.1| Ribosomal protein L10 [Prochlorococcus marinus subsp. marinus str. CCMP1375]

MGRTLESKKQIVEEIKGLLDKADMALVLDYQGLSIKEMSDLRSRLEQSSGICKVTKNTLMRKAINGDATW

SGLESLLNGTNAFVLVKGDVGSALKAVQAFQKETKKSETKGGLYEGKLLTQDEIKAIAALPSKEALMAQI

AGALNSITTKIAVGVNEIPSGLARSLKQHAENSES

>gi|33238813|gb|AAQ00878.1| Ribosomal protein L9 [Prochlorococcus marinus subsp. marinus str. CCMP1375]

MAKRVKVVLKEDILSLGKDGDVVEVAPGYARNFLLSQQKALAVTPSVLKQVEYRLAKKAELEAAKKQEAI

DFETALKTIGRFSIKKQTGEDGVLFGTVTNGDVSEAIQLATQKEIDRRNIIVPEIHETGKYKVQVKLHSE

VTAEINLEVIGN

>gi|33238677|gb|AAQ00742.1| Ribosomal protein L6P/L9E [Prochlorococcus marinus subsp. marinus str. CCMP1375]

MSRIGKKPIPVPEKVAVTLDGLLVTVKGPKGELTRTLPEGVTIDQTDGLIIVSADSEKRKSRERHGLSRT

LVANMIEGVNNGYSKQLEIVGVGSRAQVKGKTLVVSAGYSHPVEVIPPEGITFKVENNTNVLVSGIDKEL

VGNEAAKIRAIRPPEPYKGKGIKYLGERILRKAGKSGKK

>gi|33238675|gb|AAQ00740.1| Ribosomal protein S5 [Prochlorococcus marinus subsp. marinus str. CCMP1375]

MTETKAKSKSKENSSSPVPAAAEGQQQEQKRGNSRGGERRGRRSDRRNQDRDSEWQERVIQIRRVSKTVK

GGKKMSFRAIVVVGNEKGQVGVGVGKAGDVIGAVRKGVADGKKNLVRVPLTPNSSIPTLSNGRDGAASVL

IRPAAPGTGVIAGGSIRTVLELAGIKNVLAKRLGSKTPLNNARAAMVALSLLRTHKATAKERGISLEQIY

S

>gi|33237207|gb|AAP99276.1| Ribosomal protein L11 [Prochlorococcus marinus subsp. marinus str. CCMP1375]

MAKKITAVIKLALQAGKANPAPPVGPALGQHGVNIMAFCKEYNARTQDKAGFVIPVEISVFEDRSFTFIT

KTPPASVLITKAAGIAKGSGDSAKGQAGSINRAQLEEIAKTKLPDLNCNNIESAMKVIAGTARNMGVSVS

D

>gi|33237205|gb|AAP99274.1| Ribosomal protein L10 [Prochlorococcus marinus subsp. marinus str. CCMP1375]

MGRTLESKKQIVEEIKGLLDKADMALVLDYQGLSIKEMSDLRSRLEQSSGICKVTKNTLMRKAINGDATW

SGLESLLNGTNAFVLVKGDVGSALKAVQAFQKETKKSETKGGLYEGKLLTQDEIKAIAALPSKEALMAQI

AGALNSITTKIAVGVNEIPSGLARSLKQHAENSES

>gi|33241156|ref|NP_876098.1| Ribosomal protein L22 [Prochlorococcus marinus subsp. marinus str. CCMP1375]

MSDLPLAQAHGRFIRGSASKVRRVLDQIRGRTYRDALIMLEFMPYRSTGPITKVLRSAVANAENNLGMDP

SSLIIKTASADMGPSMKRYRPRAQGRAFAIKKQTCHISIAVAPSNQSTTTEASD

>gi|33241155|ref|NP_876097.1| Ribosomal protein S3 [Prochlorococcus marinus subsp. marinus str. CCMP1375]

MGHKIHPNGLRLGITQEHRSRWYASSKTYPLLLQEDDRIRVFIQKKYGAAGISDVLIARKADQLEVELKT

ARPGVIVGRQGSGIEELRSGIQKTIGDRSRQVRINVVEIERVDADAHLLAEYIAQQLEKRVAFRRTIRMA

VQRAQRAGVLGLKIQVGGRLNGAEIARSEWTREGRVPLHTLRAEIDYANKTANTTYGVLGIKVWVFKGEV

LSKEEQPLPVGASPRRKGNRRPQQFEDRSNDGK

>gi|33241149|ref|NP_876091.1| Ribosomal protein L5 [Prochlorococcus marinus subsp. marinus str. CCMP1375]

MSLKNRYRETIRPKLLKDLGFSNLHQVPKVVKINVNRGLGEAAQNSKALEASLSEVSTITGQKALVTRAK

KAIAGFKIRQGMPIGCAVTLRGERMYAFLERLINLALPRIRDFRGVSPKSFDGRGNFTLGVKEQLIFPEI

SFDKIDAIRGMDITIVTSARTDEEGRALLKEMGMPFRSN

>gi|33240808|ref|NP_875750.1| Ribosomal protein S16 [Prochlorococcus marinus subsp. marinus str. CCMP1375]

MIKLRLKRFGKKREASFRLVACNSTSRRDGRPLQELGFYNPRTKETRLDTEALRLRLSQGAQPTDAVRSL

LEKGGLLEKTIRPAELIGKSKQEELRKSEAKTSAKNKKANEEKANEEKVEESETLEASSEA

>gi|33239861|ref|NP_874803.1| Ribosomal protein S4 [Prochlorococcus marinus subsp. marinus str. CCMP1375]

MSRYRGPRLRITRRLGDLPGLTRKAAKRSNPPGQHGNARRKRSEYAIRLEEKQKLRFNYGISERQLVRYV

KKARAMEGSTGTNLLKLLEGRLDNVCFRLGFGPTIPGSRQLVNHGHVTVNGKTLDIASYQCKSGDTIAIR

ERKGSKKLAEGNLEFPGLANVPPHLELEKSKMTAKVTGKCDREWVAIEINELLVVEYYSRKV

>gi|33239681|ref|NP_874623.1| Ribosomal protein L1 [Prochlorococcus marinus subsp. marinus str. CCMP1375]

MTKISKRMKSLSAKVEDKSYAPLEAIQLVKENANAKFDETIEAHIRLGIDPKYTDQQIRTTVSLPKGTGQ

KVRIAVIAKGEKVAEANSAGADLAGEEELIDSISKGEMNFDLLISTPDMMPKVAKLGRVLGPRGLMPNPK

AGTVTTDLISSIKEFKAGKLEFRADRAGIVHVRFGKASFSPEDLLENLKVLHEAIDRNKPSGAKGRYWKS

LYITSTMGPSVEIDIAALQDSKEE

>gi|33239679|ref|NP_874621.1| Ribosomal protein L7/L12 [Prochlorococcus marinus subsp. marinus str. CCMP1375]

MSKKTDEILDSLKSLSLLEASELVKQIEEAFGVSAAASAGVVMAAPGAATGGGEAAAEEKTEFDVVLESF

DASAKIKVLKEVRNATGLGLGDAKAMVEAAPKTIKEGASKEDAEALKKAIEAVGGKVTLK

>gi|33238686|gb|AAQ00751.1| Ribosomal protein L22 [Prochlorococcus marinus subsp. marinus str. CCMP1375]

MSDLPLAQAHGRFIRGSASKVRRVLDQIRGRTYRDALIMLEFMPYRSTGPITKVLRSAVANAENNLGMDP

SSLIIKTASADMGPSMKRYRPRAQGRAFAIKKQTCHISIAVAPSNQSTTTEASD

>gi|33238685|gb|AAQ00750.1| Ribosomal protein S3 [Prochlorococcus marinus subsp. marinus str. CCMP1375]

MGHKIHPNGLRLGITQEHRSRWYASSKTYPLLLQEDDRIRVFIQKKYGAAGISDVLIARKADQLEVELKT

ARPGVIVGRQGSGIEELRSGIQKTIGDRSRQVRINVVEIERVDADAHLLAEYIAQQLEKRVAFRRTIRMA

VQRAQRAGVLGLKIQVGGRLNGAEIARSEWTREGRVPLHTLRAEIDYANKTANTTYGVLGIKVWVFKGEV

LSKEEQPLPVGASPRRKGNRRPQQFEDRSNDGK

>gi|33238679|gb|AAQ00744.1| Ribosomal protein L5 [Prochlorococcus marinus subsp. marinus str. CCMP1375]

MSLKNRYRETIRPKLLKDLGFSNLHQVPKVVKINVNRGLGEAAQNSKALEASLSEVSTITGQKALVTRAK

KAIAGFKIRQGMPIGCAVTLRGERMYAFLERLINLALPRIRDFRGVSPKSFDGRGNFTLGVKEQLIFPEI

SFDKIDAIRGMDITIVTSARTDEEGRALLKEMGMPFRSN

>gi|33238337|gb|AAQ00403.1| Ribosomal protein S16 [Prochlorococcus marinus subsp. marinus str. CCMP1375]

MIKLRLKRFGKKREASFRLVACNSTSRRDGRPLQELGFYNPRTKETRLDTEALRLRLSQGAQPTDAVRSL

LEKGGLLEKTIRPAELIGKSKQEELRKSEAKTSAKNKKANEEKANEEKVEESETLEASSEA

>gi|33237387|gb|AAP99455.1| Ribosomal protein S4 [Prochlorococcus marinus subsp. marinus str. CCMP1375]

MSRYRGPRLRITRRLGDLPGLTRKAAKRSNPPGQHGNARRKRSEYAIRLEEKQKLRFNYGISERQLVRYV

KKARAMEGSTGTNLLKLLEGRLDNVCFRLGFGPTIPGSRQLVNHGHVTVNGKTLDIASYQCKSGDTIAIR

ERKGSKKLAEGNLEFPGLANVPPHLELEKSKMTAKVTGKCDREWVAIEINELLVVEYYSRKV

>gi|33237206|gb|AAP99275.1| Ribosomal protein L1 [Prochlorococcus marinus subsp. marinus str. CCMP1375]

MTKISKRMKSLSAKVEDKSYAPLEAIQLVKENANAKFDETIEAHIRLGIDPKYTDQQIRTTVSLPKGTGQ

KVRIAVIAKGEKVAEANSAGADLAGEEELIDSISKGEMNFDLLISTPDMMPKVAKLGRVLGPRGLMPNPK

AGTVTTDLISSIKEFKAGKLEFRADRAGIVHVRFGKASFSPEDLLENLKVLHEAIDRNKPSGAKGRYWKS

LYITSTMGPSVEIDIAALQDSKEE

>gi|33237204|gb|AAP99273.1| Ribosomal protein L7/L12 [Prochlorococcus marinus subsp. marinus str. CCMP1375]

MSKKTDEILDSLKSLSLLEASELVKQIEEAFGVSAAASAGVVMAAPGAATGGGEAAAEEKTEFDVVLESF

DASAKIKVLKEVRNATGLGLGDAKAMVEAAPKTIKEGASKEDAEALKKAIEAVGGKVTLK

>gi|566703056|ref|NP_875325.3| Ribosomal protein L28 [Prochlorococcus marinus subsp. marinus str. CCMP1375]

MDRAMSRVCELSGTRANNGMAVSHSHIRTKKLQQANLQKRRLWWEEGKKWLNIRVSTSTLKTIQKKGLDS

YAKSQGIDLKKL

>gi|33241323|ref|NP_876265.1| Ribosomal protein S6 [Prochlorococcus marinus subsp. marinus str. CCMP1375]

MSDPTYYETMYILRPDIPEDEVEGHLKKYSEVLEKAKAKIIDNQMRGKRRLAYTIGKHKEGIYVQLSHTG

NGKHVETLERSMRLSEDVIRYLTVKQYGPLPTKRNTKSQDKEASTTNNENDTKEVKEAKDTKEVKEAKDT

KEVKEAKDTKEVKEAKDTKEVKEAKDTKEVKEAKDTKEVKEAKDTKEVKEAKDTKEVKEAKDTKEVKEEG

>gi|33241271|ref|NP_876213.1| Ribosomal protein L20 [Prochlorococcus marinus subsp. marinus str. CCMP1375]

MSRVKRGNVARKRRNKILRLAKGFQGSNGSLFRTANQRVMKALCNAYRDRKRRKRDFRRLWIARINAAAR

MNGMSYSKLIGNLKKADIRINRKMLAQIAILDPTNFQKVVADVKK

>gi|33241270|ref|NP_876212.1| Ribosomal protein L35 [Prochlorococcus marinus subsp. marinus str. CCMP1375]

MPKLKTRKAAAKRFKVTGTGKFMRRRAFRNHLLDHKSSKLKRHLGTKAVVDERDSENVSLMLPYS

>gi|33241161|ref|NP_876103.1| Ribosomal protein L3 [Prochlorococcus marinus subsp. marinus str. CCMP1375]

MSIGILGKKLGMSQFFDEKGRSIPVTLVEAGPCRITQLKSSATDGYSAVQIAFGAVREKLINKPFQGHLA

KSGDDLLRYMSEYRVKDVGEFQLGAQITVGDFEEGQKVDVSGTSMGRGFSGYQKRHGFSRGPMSHGSKNH

REPGSTGAGTTPGRIYPGKRMAGRYGGKKITTRGLMIMKVDSDKNLLVIKGSLPGKPGSLLNIRPAKCVG

LNSSKGGNK

>gi|33241160|ref|NP_876102.1| Ribosomal protein L4 [Prochlorococcus marinus subsp. marinus str. CCMP1375]

MANCAVLDWQGKEAGKASLNLKVAKDSSAVDLMHRAVLRQQAHSRQGTASTLTRAEVRGGGRKPYKQKGT

GRARQGSIRTPLRPGGGIIFGPKPRQYNLSMNRKERRLALRTALMARFNDVIAVKDFGSKLKVPKTKEIQ

DFLARLDISSNSKVLIILSQPSDIIRRSVRNLEKVKLIAAEHLNVFDLLNANSLIIGEDALGKIKEVYGD

D

>gi|33241159|ref|NP_876101.1| Ribosomal protein L23 [Prochlorococcus marinus subsp. marinus str. CCMP1375]

MTEMFKERLADVIRRPLITEKATKGLDINQYTFEVDHRAAKPDIKAAVEKLFDVKVIGISTMNPPRRSRR

VGRFSGKRAQVKKAIVRLAEGNSIQLFPDSES

>gi|33241158|ref|NP_876100.1| Ribosomal protein L2 [Prochlorococcus marinus subsp. marinus str. CCMP1375]

MAIRTFRPYTPGTRTRVVTDFNEVTGRKPERSLVIAKHRLKGRNNRGVITCRHRGGGHKRQYRLVDFRRN

KHGVPAKVAAIHYDPHRNARLALLFYKDGEKRYILAPAGISIGQEVLSGKDVPIEIGNALPLSSIPLGSS

VHCVELYPGRGGQMVRCAGSSAQLMAKEGEYVALKLPSTEVRLVRGECYATLGEVGNSEIRNTSLGKAGR

RRWLGRRPQVRGSVMNPCDHPHGGGEGRAPVGRAGPVTPWGKPALGLKTRKRNKPSNRFVLRKRRRVSKR

SRGGRDS

>gi|33241157|ref|NP_876099.1| Ribosomal protein S19 [Prochlorococcus marinus subsp. marinus str. CCMP1375]

MGRSLKKGPFIADSLIKKVEKQNSDDDKSVIKTWSRASTILPMMIGHTIAVHNGKTHVPVFITEQMVGHK

LGEFAPTRTYRGHMKDKKGGR

>gi|33241152|ref|NP_876094.1| Ribosomal protein S17 [Prochlorococcus marinus subsp. marinus str. CCMP1375]

MALKERLGTVVSDKMDKTVVVAVVNRFPHSIYQKTVSRTTRYKAHDEENNCKVGDRVRITETRPLSATKR

WSVAEVLRTTKQEKEAVK

>gi|33241151|ref|NP_876093.1| Ribosomal protein L14 [Prochlorococcus marinus subsp. marinus str. CCMP1375]

MIQQETYLTVADNSGAKRLQCIRVLGSNRRYAHVGDVIVAAVKDAVPNMGVKKSDVVKAVVVRTKATLRR

ETGNSIRFDDNAAVLINEDKNPRGTRVFGPVARELRERNYTKIVSLAPEVI

>gi|33241150|ref|NP_876092.1| Ribosomal protein L24 [Prochlorococcus marinus subsp. marinus str. CCMP1375]

MFNLNQTKKASLPIKMRIRKGDTVQVINGKEKGKTGEVLKTLPIENRVIVQGINLRTRHVKPTQEGESGR

IVTEEASLHASNVMVYSTKQKTASRVEVFIDKDGSKKRRLKKTGELID

>gi|33241148|ref|NP_876090.1| Ribosomal protein S8 [Prochlorococcus marinus subsp. marinus str. CCMP1375]

MANHDPISDMLTRIRNASQKRHENTRIPASRMSRSIAKVLQNEGFISQVSEEGEGVKTQLVLELKYSGKH

RHPTIRSMKRVSKPGLRIYKNNRGLPKVLGGLGVAIISTSKGVMSDRDARKQGVGGEVLCYVY

>gi|33241146|ref|NP_876088.1| Ribosomal protein L18 [Prochlorococcus marinus subsp. marinus str. CCMP1375]

MSTLSRKQKTQKRHKRLRRNLSGTDQRPRLAVFRSNNHIYAQVIDDVAQNTLCAASTLEKEFLSNSKVNA

STCAASTAVGEMLAKRALGKGIKQVVFDRGGSLYHGRVKALAEAARQAGLTF

>gi|33241144|ref|NP_876086.1| Ribosomal protein L15 [Prochlorococcus marinus subsp. marinus str. CCMP1375]

MTSISLDSLKPNKGARKRKTRKGRGIAAGQGASCGFGMRGQKSRSGRPTRPGFEGGQMPLYRRVPKLKHF

PLVNSKSFTLINVASLNSLKEGSTVNLDSLVKTGIVTSPKYPLKVLGNGNLKVKLVVQAAAFTASAKTKI

EGAGGSCEVFE

>gi|33241141|ref|NP_876083.1| Ribosomal protein L36 [Prochlorococcus marinus subsp. marinus str. CCMP1375]

MKVRASVKKMCEKCRVIRRHGRVMVICTATQKHKQRQG

>gi|33241140|ref|NP_876082.1| Ribosomal protein S13 [Prochlorococcus marinus subsp. marinus str. CCMP1375]

MARIAGIDLPRDKRVEIALTYIYGIGLTRAQNILAKTGVNPDIRVKDLEDGDVQKLRAATESFTIEGDLR

RQEGMALKRLQDIGCLRGRRHRMSLPVRGQRTRTNARTRRGSRKTVAGRKK

>gi|33241139|ref|NP_876081.1| Ribosomal protein S11 [Prochlorococcus marinus subsp. marinus str. CCMP1375]

MATPAKKTGSKKSKRNVPNGVVHIQSTFNNTIVSITDTNGEVISWSSAGASGFKGARKGTPFAAQTAAEA

AARRALDQGMRQIEVLVRGPGSGRETAIRALQVAGLEITLIRDVTPLPHNGCRRAKRRRV

>gi|33241137|ref|NP_876079.1| Ribosomal protein L17 [Prochlorococcus marinus subsp. marinus str. CCMP1375]

MRHQLRVPQLGRPADQRKALLRGLTTQLIREGRVTTTKTRAKALRDEVERMIGLAKDGSLAARRRAIGYI

YDKKLVHSLFEKAQERYGDRQGGYTRIVRTVPRRGDNAEMAIIELV

>gi|33241135|ref|NP_876077.1| Ribosomal protein L13 [Prochlorococcus marinus subsp. marinus str. CCMP1375]

MNKTITPSIDSINRQWYLVDAENQTLGRLATEVASVLRGKNKPSFTPHLDTGDFVIIVNAEKIKVTGKKG

MQKLYRRHSGRPGGMKVETFNSLQERIPERIVEKAIKGMLPHNALGRQLFRKLKVYKGSEHPHSAQNPQV

LSITTNELVK

>gi|33241134|ref|NP_876076.1| Ribosomal protein S9 [Prochlorococcus marinus subsp. marinus str. CCMP1375]

MNSSSKNAVVYWGTGRRKTSVARVRLIPGTGKITINGRPGDHYLNFNPAYLSAVKAPLHTLGLGEAYDVL

VNVYGGGLTGQSDAIKQGAARALCELSVDNRKPLKTEGHLSRDPRAKERRKYGLKKARKAPQFSKR

>gi|33241133|ref|NP_876075.1| Ribosomal protein L31 [Prochlorococcus marinus subsp. marinus str. CCMP1375]

MPKKDIHPNWYPDAKVICNGEVVMTTGSTQPEIHVDVWSGNHPFFTGTQKILDTEGRVDRFMRKYGMANP

DEDSTKNTKSSKKETSEDSSSKGS

>gi|33241116|ref|NP_876058.1| Ribosomal protein S12 [Prochlorococcus marinus subsp. marinus str. CCMP1375]

MPTIQQLIRTERQRLTRKTKSPALRSCPERRGVCTRVYTSTPKKPNSALRKVARVRLTSGFEVTAYIPGI

GHNLQEHSVVLLRGGRVKDLPGVRYHIIRGTLDTAGVKDRRQARSKYGAKAPKS

>gi|33241115|ref|NP_876057.1| Ribosomal protein S7 [Prochlorococcus marinus subsp. marinus str. CCMP1375]

MSRRNAAEKRPVLPDPQFNNRLATMMVARLMKHGKKSTAQKILSQAFGLINERTGGDPIELFETAIKNAT

PLVEVRARRVGGATYQVPMEVRQERGTAMALRWLVNFSRSRNGRSMAHKLAGELMDAANEAGNAVRKREE

THKMAEANKAFAHYRY

>gi|33241112|ref|NP_876054.1| Ribosomal protein S10 [Prochlorococcus marinus subsp. marinus str. CCMP1375]

MSTAIAQQKIRIRLKAFDRRMLDLSCDKIIETADNTAATAIGPIPLPTKRKIYCVLRSPHVDKDSREHFE

TRTHRRIIDIYSPSAKTIDALMKLDLPSGVDIEVKL

>gi|33241091|ref|NP_876033.1| Ribosomal protein S20 [Prochlorococcus marinus subsp. marinus str. CCMP1375]

MANNNSAKKRIQIAERNRIQNRTYKSAMRTLMKRCFEACGAYSEKPSEDAKKDIQNSMNDAFSKIDKAVK

TGVLHRNTGANQKSRLTSVVKKTIEPVVK

>gi|33240884|ref|NP_875826.1| Ribosomal protein L11 methylase [Prochlorococcus marinus subsp. marinus str. CCMP1375]

MIQSTEFFWWKFELVFPPDVEESFLWFLNMAGIKSYAIERSPDNLQDQTLMVWLPSHEWLKKDREEFENS

LLALNKAFREDVLNTKWEKIIDEDWSSSWKKFWKADPVGSKILILPSWLELPDIYSNRIVIKLDPGSAFG

TGSHPTTRLCLEDLERNPPLGKKVVDIGCGSGVLGIAAIKLGAKEVRAIDIDSLAVRATSENIVLNNLSQ

KQLSVSLGSIENLANQLNPLSADLLICNTLSPVIKELAPYFFKLTHSYSRLCLSGLLVAQVEDITNFLSI

LGWELIDSYSSDNWALIRLCRNHP

>gi|33240875|ref|NP_875817.1| Ribosomal protein L27 [Prochlorococcus marinus subsp. marinus str. CCMP1375]

MAHKKGTGSTRNGRDSNSKRLGVKAYGGEKVTAGSILIRQRGTSVLPGANVGQGKDDTLFALVDGIVNFE

TIKRSLKKRKRISVSLA

>gi|33240874|ref|NP_875816.1| Ribosomal protein L21 [Prochlorococcus marinus subsp. marinus str. CCMP1375]

MSFDFMTPNKTKNSTVGNYAIVETSGTQFWLEADRYYDIDRINANVDETVTLDKVLLINDQKGFAIGKPY

IKGASVQLKVMAHKRGPKIIVYKMRPKKKTRTKNGHRQELTRVMVTSISNGEKPKKATTSAKPNTKKPST

AVKSSKVEKTPE

>gi|33240755|ref|NP_875697.1| Ribosomal protein S14 [Prochlorococcus marinus subsp. marinus str. CCMP1375]

MAKKSMIARDVKRKKLVERYASKRKKLLDQFHSAKDPMERLEIHRKIQALPRNSAPSRMRNRCWATGKPR

GVYRDFGLCRNQLRERAHKGELPGVVKSS

>gi|33240748|ref|NP_875690.1| Ribosomal protein L34 [Prochlorococcus marinus subsp. marinus str. CCMP1375]

MTKRTFGGTSRKRKRVSGFRVRMRTHTGRSVIRSRRKKGRSRIAV

>gi|33240431|ref|NP_875373.1| Ribosomal protein L32 [Prochlorococcus marinus subsp. marinus str. CCMP1375]

MAVPKKKTSKGKRNQRHAVWKAKAGDAAQKALSLGKSILSGRAQGFVYPIQEEDSEE

>gi|33240418|ref|NP_875360.1| Ribosomal protein S18 [Prochlorococcus marinus subsp. marinus str. CCMP1375]

MPNSLFKKKLSPIKPGDPIDYKDVETLKKFITERGKILPRRLTGLTAKQQRDLTTAVKRARIIALLPFVN

PEG

>gi|33240417|ref|NP_875359.1| Ribosomal protein L33 [Prochlorococcus marinus subsp. marinus str. CCMP1375]

MAKKGTRIVVTLECTECRSAPASEKRSPGVSRYTTEKNRRNTSDRLELKKFCPQLNKMTIHKEIK

>gi|33240275|ref|NP_875217.1| Ribosomal protein S2 [Prochlorococcus marinus subsp. marinus str. CCMP1375]

MAVVTLSEMMEAGAHFGHQTRRWNPKMSRYIYCARNGVHIIDLVKTAVCMNNAYKWTRNAAKSGKRFLFV

GTKKQASEVVAQEAARCGASYVNQRWLGGMLTNWTTMKARIDRLKDLERMESSGAIAMRPKKEASVLRHE

LERLQKYLGGLKGMKRLPDVVVLVDQRRETNAVLEARKLDIPLVSMLDTNCDPDLCEVPIPCNDDAVRSV

QLVLGRIADAINEGRHGSNDQRARQKYS

>gi|33240203|ref|NP_875145.1| Ribosomal protein S15 [Prochlorococcus marinus subsp. marinus str. CCMP1375]

MTLNTEAKQKIINKHQTHGTDTGSVEVQVAMLSERINQLSKHLQSNNHDFSSRQGLLKMIGQRKRLLNYV

KKQSESRYSSLVTKLGIRG

>gi|33240041|ref|NP_874983.1| Ribosomal protein S21 [Prochlorococcus marinus subsp. marinus str. CCMP1375]

MTQVIVGENEGVESALRRFKREVSKAGIFNDLKRIRHHETPVEKYKRKQRLKNRTKRRR

>gi|33239924|ref|NP_874866.1| Ribosomal protein L19 [Prochlorococcus marinus subsp. marinus str. CCMP1375]

MTEDLKNTSPSKEESNEIEESSKATPKATRETKPKDSPSKTKLSAQALIAQFEKSQQKKKVPEVYVGDTV

RVGVRISEGNKERVQPYEGVIIAKRHGGLNQTITVRRIFQGVGVERVFMVHSPQVASIKVERRGKVRRAK

LFYLRDRVGKATRVKQRFDR

>gi|33238853|gb|AAQ00918.1| Ribosomal protein S6 [Prochlorococcus marinus subsp. marinus str. CCMP1375]

MSDPTYYETMYILRPDIPEDEVEGHLKKYSEVLEKAKAKIIDNQMRGKRRLAYTIGKHKEGIYVQLSHTG

NGKHVETLERSMRLSEDVIRYLTVKQYGPLPTKRNTKSQDKEASTTNNENDTKEVKEAKDTKEVKEAKDT

KEVKEAKDTKEVKEAKDTKEVKEAKDTKEVKEAKDTKEVKEAKDTKEVKEAKDTKEVKEAKDTKEVKEEG

>gi|33238801|gb|AAQ00866.1| Ribosomal protein L20 [Prochlorococcus marinus subsp. marinus str. CCMP1375]

MSRVKRGNVARKRRNKILRLAKGFQGSNGSLFRTANQRVMKALCNAYRDRKRRKRDFRRLWIARINAAAR

MNGMSYSKLIGNLKKADIRINRKMLAQIAILDPTNFQKVVADVKK

>gi|33238800|gb|AAQ00865.1| Ribosomal protein L35 [Prochlorococcus marinus subsp. marinus str. CCMP1375]

MPKLKTRKAAAKRFKVTGTGKFMRRRAFRNHLLDHKSSKLKRHLGTKAVVDERDSENVSLMLPYS

>gi|33238691|gb|AAQ00756.1| Ribosomal protein L3 [Prochlorococcus marinus subsp. marinus str. CCMP1375]

MSIGILGKKLGMSQFFDEKGRSIPVTLVEAGPCRITQLKSSATDGYSAVQIAFGAVREKLINKPFQGHLA

KSGDDLLRYMSEYRVKDVGEFQLGAQITVGDFEEGQKVDVSGTSMGRGFSGYQKRHGFSRGPMSHGSKNH

REPGSTGAGTTPGRIYPGKRMAGRYGGKKITTRGLMIMKVDSDKNLLVIKGSLPGKPGSLLNIRPAKCVG

LNSSKGGNK

>gi|33238690|gb|AAQ00755.1| Ribosomal protein L4 [Prochlorococcus marinus subsp. marinus str. CCMP1375]

MANCAVLDWQGKEAGKASLNLKVAKDSSAVDLMHRAVLRQQAHSRQGTASTLTRAEVRGGGRKPYKQKGT

GRARQGSIRTPLRPGGGIIFGPKPRQYNLSMNRKERRLALRTALMARFNDVIAVKDFGSKLKVPKTKEIQ

DFLARLDISSNSKVLIILSQPSDIIRRSVRNLEKVKLIAAEHLNVFDLLNANSLIIGEDALGKIKEVYGD

D

>gi|33238689|gb|AAQ00754.1| Ribosomal protein L23 [Prochlorococcus marinus subsp. marinus str. CCMP1375]

MTEMFKERLADVIRRPLITEKATKGLDINQYTFEVDHRAAKPDIKAAVEKLFDVKVIGISTMNPPRRSRR

VGRFSGKRAQVKKAIVRLAEGNSIQLFPDSES

>gi|33238688|gb|AAQ00753.1| Ribosomal protein L2 [Prochlorococcus marinus subsp. marinus str. CCMP1375]

MAIRTFRPYTPGTRTRVVTDFNEVTGRKPERSLVIAKHRLKGRNNRGVITCRHRGGGHKRQYRLVDFRRN

KHGVPAKVAAIHYDPHRNARLALLFYKDGEKRYILAPAGISIGQEVLSGKDVPIEIGNALPLSSIPLGSS

VHCVELYPGRGGQMVRCAGSSAQLMAKEGEYVALKLPSTEVRLVRGECYATLGEVGNSEIRNTSLGKAGR

RRWLGRRPQVRGSVMNPCDHPHGGGEGRAPVGRAGPVTPWGKPALGLKTRKRNKPSNRFVLRKRRRVSKR

SRGGRDS

>gi|33238687|gb|AAQ00752.1| Ribosomal protein S19 [Prochlorococcus marinus subsp. marinus str. CCMP1375]

MGRSLKKGPFIADSLIKKVEKQNSDDDKSVIKTWSRASTILPMMIGHTIAVHNGKTHVPVFITEQMVGHK

LGEFAPTRTYRGHMKDKKGGR

>gi|33238682|gb|AAQ00747.1| Ribosomal protein S17 [Prochlorococcus marinus subsp. marinus str. CCMP1375]

MALKERLGTVVSDKMDKTVVVAVVNRFPHSIYQKTVSRTTRYKAHDEENNCKVGDRVRITETRPLSATKR

WSVAEVLRTTKQEKEAVK

>gi|33238681|gb|AAQ00746.1| Ribosomal protein L14 [Prochlorococcus marinus subsp. marinus str. CCMP1375]

MIQQETYLTVADNSGAKRLQCIRVLGSNRRYAHVGDVIVAAVKDAVPNMGVKKSDVVKAVVVRTKATLRR

ETGNSIRFDDNAAVLINEDKNPRGTRVFGPVARELRERNYTKIVSLAPEVI

>gi|33238680|gb|AAQ00745.1| Ribosomal protein L24 [Prochlorococcus marinus subsp. marinus str. CCMP1375]

MFNLNQTKKASLPIKMRIRKGDTVQVINGKEKGKTGEVLKTLPIENRVIVQGINLRTRHVKPTQEGESGR

IVTEEASLHASNVMVYSTKQKTASRVEVFIDKDGSKKRRLKKTGELID

>gi|33238678|gb|AAQ00743.1| Ribosomal protein S8 [Prochlorococcus marinus subsp. marinus str. CCMP1375]

MANHDPISDMLTRIRNASQKRHENTRIPASRMSRSIAKVLQNEGFISQVSEEGEGVKTQLVLELKYSGKH

RHPTIRSMKRVSKPGLRIYKNNRGLPKVLGGLGVAIISTSKGVMSDRDARKQGVGGEVLCYVY

>gi|33238676|gb|AAQ00741.1| Ribosomal protein L18 [Prochlorococcus marinus subsp. marinus str. CCMP1375]

MSTLSRKQKTQKRHKRLRRNLSGTDQRPRLAVFRSNNHIYAQVIDDVAQNTLCAASTLEKEFLSNSKVNA

STCAASTAVGEMLAKRALGKGIKQVVFDRGGSLYHGRVKALAEAARQAGLTF

>gi|33238674|gb|AAQ00739.1| Ribosomal protein L15 [Prochlorococcus marinus subsp. marinus str. CCMP1375]

MTSISLDSLKPNKGARKRKTRKGRGIAAGQGASCGFGMRGQKSRSGRPTRPGFEGGQMPLYRRVPKLKHF

PLVNSKSFTLINVASLNSLKEGSTVNLDSLVKTGIVTSPKYPLKVLGNGNLKVKLVVQAAAFTASAKTKI

EGAGGSCEVFE

>gi|33238671|gb|AAQ00736.1| Ribosomal protein L36 [Prochlorococcus marinus subsp. marinus str. CCMP1375]

MKVRASVKKMCEKCRVIRRHGRVMVICTATQKHKQRQG

>gi|33238670|gb|AAQ00735.1| Ribosomal protein S13 [Prochlorococcus marinus subsp. marinus str. CCMP1375]

MARIAGIDLPRDKRVEIALTYIYGIGLTRAQNILAKTGVNPDIRVKDLEDGDVQKLRAATESFTIEGDLR

RQEGMALKRLQDIGCLRGRRHRMSLPVRGQRTRTNARTRRGSRKTVAGRKK

>gi|33238669|gb|AAQ00734.1| Ribosomal protein S11 [Prochlorococcus marinus subsp. marinus str. CCMP1375]

MATPAKKTGSKKSKRNVPNGVVHIQSTFNNTIVSITDTNGEVISWSSAGASGFKGARKGTPFAAQTAAEA

AARRALDQGMRQIEVLVRGPGSGRETAIRALQVAGLEITLIRDVTPLPHNGCRRAKRRRV

>gi|33238667|gb|AAQ00732.1| Ribosomal protein L17 [Prochlorococcus marinus subsp. marinus str. CCMP1375]

MRHQLRVPQLGRPADQRKALLRGLTTQLIREGRVTTTKTRAKALRDEVERMIGLAKDGSLAARRRAIGYI

YDKKLVHSLFEKAQERYGDRQGGYTRIVRTVPRRGDNAEMAIIELV

>gi|33238665|gb|AAQ00730.1| Ribosomal protein L13 [Prochlorococcus marinus subsp. marinus str. CCMP1375]

MNKTITPSIDSINRQWYLVDAENQTLGRLATEVASVLRGKNKPSFTPHLDTGDFVIIVNAEKIKVTGKKG

MQKLYRRHSGRPGGMKVETFNSLQERIPERIVEKAIKGMLPHNALGRQLFRKLKVYKGSEHPHSAQNPQV

LSITTNELVK

>gi|33238664|gb|AAQ00729.1| Ribosomal protein S9 [Prochlorococcus marinus subsp. marinus str. CCMP1375]

MNSSSKNAVVYWGTGRRKTSVARVRLIPGTGKITINGRPGDHYLNFNPAYLSAVKAPLHTLGLGEAYDVL

VNVYGGGLTGQSDAIKQGAARALCELSVDNRKPLKTEGHLSRDPRAKERRKYGLKKARKAPQFSKR

>gi|33238663|gb|AAQ00728.1| Ribosomal protein L31 [Prochlorococcus marinus subsp. marinus str. CCMP1375]

MPKKDIHPNWYPDAKVICNGEVVMTTGSTQPEIHVDVWSGNHPFFTGTQKILDTEGRVDRFMRKYGMANP

DEDSTKNTKSSKKETSEDSSSKGS

>gi|33238646|gb|AAQ00711.1| Ribosomal protein S12 [Prochlorococcus marinus subsp. marinus str. CCMP1375]

MPTIQQLIRTERQRLTRKTKSPALRSCPERRGVCTRVYTSTPKKPNSALRKVARVRLTSGFEVTAYIPGI

GHNLQEHSVVLLRGGRVKDLPGVRYHIIRGTLDTAGVKDRRQARSKYGAKAPKS

>gi|33238645|gb|AAQ00710.1| Ribosomal protein S7 [Prochlorococcus marinus subsp. marinus str. CCMP1375]

MSRRNAAEKRPVLPDPQFNNRLATMMVARLMKHGKKSTAQKILSQAFGLINERTGGDPIELFETAIKNAT

PLVEVRARRVGGATYQVPMEVRQERGTAMALRWLVNFSRSRNGRSMAHKLAGELMDAANEAGNAVRKREE

THKMAEANKAFAHYRY

>gi|33238642|gb|AAQ00707.1| Ribosomal protein S10 [Prochlorococcus marinus subsp. marinus str. CCMP1375]

MSTAIAQQKIRIRLKAFDRRMLDLSCDKIIETADNTAATAIGPIPLPTKRKIYCVLRSPHVDKDSREHFE

TRTHRRIIDIYSPSAKTIDALMKLDLPSGVDIEVKL

>gi|33238620|gb|AAQ00686.1| Ribosomal protein S20 [Prochlorococcus marinus subsp. marinus str. CCMP1375]

MANNNSAKKRIQIAERNRIQNRTYKSAMRTLMKRCFEACGAYSEKPSEDAKKDIQNSMNDAFSKIDKAVK

TGVLHRNTGANQKSRLTSVVKKTIEPVVK

>gi|33238413|gb|AAQ00479.1| Ribosomal protein L11 methylase [Prochlorococcus marinus subsp. marinus str. CCMP1375]

MIQSTEFFWWKFELVFPPDVEESFLWFLNMAGIKSYAIERSPDNLQDQTLMVWLPSHEWLKKDREEFENS

LLALNKAFREDVLNTKWEKIIDEDWSSSWKKFWKADPVGSKILILPSWLELPDIYSNRIVIKLDPGSAFG

TGSHPTTRLCLEDLERNPPLGKKVVDIGCGSGVLGIAAIKLGAKEVRAIDIDSLAVRATSENIVLNNLSQ

KQLSVSLGSIENLANQLNPLSADLLICNTLSPVIKELAPYFFKLTHSYSRLCLSGLLVAQVEDITNFLSI

LGWELIDSYSSDNWALIRLCRNHP

>gi|33238404|gb|AAQ00470.1| Ribosomal protein L27 [Prochlorococcus marinus subsp. marinus str. CCMP1375]

MAHKKGTGSTRNGRDSNSKRLGVKAYGGEKVTAGSILIRQRGTSVLPGANVGQGKDDTLFALVDGIVNFE

TIKRSLKKRKRISVSLA

>gi|33238403|gb|AAQ00469.1| Ribosomal protein L21 [Prochlorococcus marinus subsp. marinus str. CCMP1375]

MSFDFMTPNKTKNSTVGNYAIVETSGTQFWLEADRYYDIDRINANVDETVTLDKVLLINDQKGFAIGKPY

IKGASVQLKVMAHKRGPKIIVYKMRPKKKTRTKNGHRQELTRVMVTSISNGEKPKKATTSAKPNTKKPST

AVKSSKVEKTPE

>gi|33238283|gb|AAQ00350.1| Ribosomal protein S14 [Prochlorococcus marinus subsp. marinus str. CCMP1375]

MAKKSMIARDVKRKKLVERYASKRKKLLDQFHSAKDPMERLEIHRKIQALPRNSAPSRMRNRCWATGKPR

GVYRDFGLCRNQLRERAHKGELPGVVKSS

>gi|33238276|gb|AAQ00343.1| Ribosomal protein L34 [Prochlorococcus marinus subsp. marinus str. CCMP1375]

MTKRTFGGTSRKRKRVSGFRVRMRTHTGRSVIRSRRKKGRSRIAV

>gi|33237958|gb|AAQ00026.1| Ribosomal protein L32 [Prochlorococcus marinus subsp. marinus str. CCMP1375]

MAVPKKKTSKGKRNQRHAVWKAKAGDAAQKALSLGKSILSGRAQGFVYPIQEEDSEE

>gi|33237945|gb|AAQ00013.1| Ribosomal protein S18 [Prochlorococcus marinus subsp. marinus str. CCMP1375]

MPNSLFKKKLSPIKPGDPIDYKDVETLKKFITERGKILPRRLTGLTAKQQRDLTTAVKRARIIALLPFVN

PEG

>gi|33237944|gb|AAQ00012.1| Ribosomal protein L33 [Prochlorococcus marinus subsp. marinus str. CCMP1375]

MAKKGTRIVVTLECTECRSAPASEKRSPGVSRYTTEKNRRNTSDRLELKKFCPQLNKMTIHKEIK

>gi|33237910|gb|AAP99977.1| Ribosomal protein L28 [Prochlorococcus marinus subsp. marinus str. CCMP1375]

MDRAMSRVCELSGTRANNGMAVSHSHIRTKKLQQANLQKRRLWWEEGKKWLNIRVSTSTLKTIQKKGLDS

YAKSQGIDLKKL

>gi|33237802|gb|AAP99869.1| Ribosomal protein S2 [Prochlorococcus marinus subsp. marinus str. CCMP1375]

MAVVTLSEMMEAGAHFGHQTRRWNPKMSRYIYCARNGVHIIDLVKTAVCMNNAYKWTRNAAKSGKRFLFV

GTKKQASEVVAQEAARCGASYVNQRWLGGMLTNWTTMKARIDRLKDLERMESSGAIAMRPKKEASVLRHE

LERLQKYLGGLKGMKRLPDVVVLVDQRRETNAVLEARKLDIPLVSMLDTNCDPDLCEVPIPCNDDAVRSV

QLVLGRIADAINEGRHGSNDQRARQKYS

>gi|33237730|gb|AAP99797.1| Ribosomal protein S15 [Prochlorococcus marinus subsp. marinus str. CCMP1375]

MTLNTEAKQKIINKHQTHGTDTGSVEVQVAMLSERINQLSKHLQSNNHDFSSRQGLLKMIGQRKRLLNYV

KKQSESRYSSLVTKLGIRG

>gi|33237567|gb|AAP99635.1| Ribosomal protein S21 [Prochlorococcus marinus subsp. marinus str. CCMP1375]

MTQVIVGENEGVESALRRFKREVSKAGIFNDLKRIRHHETPVEKYKRKQRLKNRTKRRR

>gi|33237450|gb|AAP99518.1| Ribosomal protein L19 [Prochlorococcus marinus subsp. marinus str. CCMP1375]

MTEDLKNTSPSKEESNEIEESSKATPKATRETKPKDSPSKTKLSAQALIAQFEKSQQKKKVPEVYVGDTV

RVGVRISEGNKERVQPYEGVIIAKRHGGLNQTITVRRIFQGVGVERVFMVHSPQVASIKVERRGKVRRAK

LFYLRDRVGKATRVKQRFDR

>gi|33241154|ref|NP_876096.1| Ribosomal protein L16/L10E [Prochlorococcus marinus subsp. marinus str. CCMP1375]

MLSPKRTKFRKQQRGRMRGVATRGNKIAFGQFALQAQECGWITSRQIEASRRAMTRYVKRGGQIWIRIFP

DKPVTMRPAETRMGSGKGNPEFWVAVVKPGRILFEMGGEEITEEIAKEAMRLAQYKLPIKTKFLALAEGE

KPTQVGKAPPKSSFLPSDETETAAAQAGTEASSASSVTPLES

>gi|33241153|ref|NP_876095.1| Ribosomal protein L29 [Prochlorococcus marinus subsp. marinus str. CCMP1375]

MAESEKLDVSKLTDIEIKEKIDVTRRELFDLRFQRATRQLNETHRFKKARVQLAQLLTAQGERSRSNT

>gi|33238684|gb|AAQ00749.1| Ribosomal protein L16/L10E [Prochlorococcus marinus subsp. marinus str. CCMP1375]

MLSPKRTKFRKQQRGRMRGVATRGNKIAFGQFALQAQECGWITSRQIEASRRAMTRYVKRGGQIWIRIFP

DKPVTMRPAETRMGSGKGNPEFWVAVVKPGRILFEMGGEEITEEIAKEAMRLAQYKLPIKTKFLALAEGE

KPTQVGKAPPKSSFLPSDETETAAAQAGTEASSASSVTPLES

>gi|33238683|gb|AAQ00748.1| Ribosomal protein L29 [Prochlorococcus marinus subsp. marinus str. CCMP1375]

MAESEKLDVSKLTDIEIKEKIDVTRRELFDLRFQRATRQLNETHRFKKARVQLAQLLTAQGERSRSNT

>gi|81712766|sp|Q7V9X6.1|RL6_PROMA RecName: Full=50S ribosomal protein L6

MSRIGKKPIPVPEKVAVTLDGLLVTVKGPKGELTRTLPEGVTIDQTDGLIIVSADSEKRKSRERHGLSRT

LVANMIEGVNNGYSKQLEIVGVGSRAQVKGKTLVVSAGYSHPVEVIPPEGITFKVENNTNVLVSGIDKEL

VGNEAAKIRAIRPPEPYKGKGIKYLGERILRKAGKSGKK

>gi|81663887|sp|Q7V9J9.1|RL9_PROMA RecName: Full=50S ribosomal protein L9

MAKRVKVVLKEDILSLGKDGDVVEVAPGYARNFLLSQQKALAVTPSVLKQVEYRLAKKAELEAAKKQEAI

DFETALKTIGRFSIKKQTGEDGVLFGTVTNGDVSEAIQLATQKEIDRRNIIVPEIHETGKYKVQVKLHSE

VTAEINLEVIGN

>gi|73919088|sp|Q7V9X8.1|RS5_PROMA RecName: Full=30S ribosomal protein S5

MTETKAKSKSKENSSSPVPAAAEGQQQEQKRGNSRGGERRGRRSDRRNQDRDSEWQERVIQIRRVSKTVK

GGKKMSFRAIVVVGNEKGQVGVGVGKAGDVIGAVRKGVADGKKNLVRVPLTPNSSIPTLSNGRDGAASVL

IRPAAPGTGVIAGGSIRTVLELAGIKNVLAKRLGSKTPLNNARAAMVALSLLRTHKATAKERGISLEQIY

S

>gi|61215250|sp|Q7VDY7.1|RL10_PROMA RecName: Full=50S ribosomal protein L10

MGRTLESKKQIVEEIKGLLDKADMALVLDYQGLSIKEMSDLRSRLEQSSGICKVTKNTLMRKAINGDATW

SGLESLLNGTNAFVLVKGDVGSALKAVQAFQKETKKSETKGGLYEGKLLTQDEIKAIAALPSKEALMAQI

AGALNSITTKIAVGVNEIPSGLARSLKQHAENSES

>gi|38605180|sp|Q7VDY5.1|RL11_PROMA RecName: Full=50S ribosomal protein L11

MAKKITAVIKLALQAGKANPAPPVGPALGQHGVNIMAFCKEYNARTQDKAGFVIPVEISVFEDRSFTFIT

KTPPASVLITKAAGIAKGSGDSAKGQAGSINRAQLEEIAKTKLPDLNCNNIESAMKVIAGTARNMGVSVS

D

>gi|81665047|sp|Q7VDY8.1|RL7_PROMA RecName: Full=50S ribosomal protein L7/L12

MSKKTDEILDSLKSLSLLEASELVKQIEEAFGVSAAASAGVVMAAPGAATGGGEAAAEEKTEFDVVLESF

DASAKIKVLKEVRNATGLGLGDAKAMVEAAPKTIKEGASKEDAEALKKAIEAVGGKVTLK

>gi|61215707|sp|Q7VDY6.1|RL1_PROMA RecName: Full=50S ribosomal protein L1

MTKISKRMKSLSAKVEDKSYAPLEAIQLVKENANAKFDETIEAHIRLGIDPKYTDQQIRTTVSLPKGTGQ

KVRIAVIAKGEKVAEANSAGADLAGEEELIDSISKGEMNFDLLISTPDMMPKVAKLGRVLGPRGLMPNPK

AGTVTTDLISSIKEFKAGKLEFRADRAGIVHVRFGKASFSPEDLLENLKVLHEAIDRNKPSGAKGRYWKS

LYITSTMGPSVEIDIAALQDSKEE

>gi|61215704|sp|Q7V9W8.1|RS3_PROMA RecName: Full=30S ribosomal protein S3

MGHKIHPNGLRLGITQEHRSRWYASSKTYPLLLQEDDRIRVFIQKKYGAAGISDVLIARKADQLEVELKT

ARPGVIVGRQGSGIEELRSGIQKTIGDRSRQVRINVVEIERVDADAHLLAEYIAQQLEKRVAFRRTIRMA

VQRAQRAGVLGLKIQVGGRLNGAEIARSEWTREGRVPLHTLRAEIDYANKTANTTYGVLGIKVWVFKGEV

LSKEEQPLPVGASPRRKGNRRPQQFEDRSNDGK

>gi|54039437|sp|Q7VAU5.1|RS16_PROMA RecName: Full=30S ribosomal protein S16

MIKLRLKRFGKKREASFRLVACNSTSRRDGRPLQELGFYNPRTKETRLDTEALRLRLSQGAQPTDAVRSL

LEKGGLLEKTIRPAELIGKSKQEELRKSEAKTSAKNKKANEEKANEEKVEESETLEASSEA

>gi|51316780|sp|Q7V9W7.1|RL22_PROMA RecName: Full=50S ribosomal protein L22

MSDLPLAQAHGRFIRGSASKVRRVLDQIRGRTYRDALIMLEFMPYRSTGPITKVLRSAVANAENNLGMDP

SSLIIKTASADMGPSMKRYRPRAQGRAFAIKKQTCHISIAVAPSNQSTTTEASD

>gi|50401255|sp|Q7V9X4.1|RL5_PROMA RecName: Full=50S ribosomal protein L5

MSLKNRYRETIRPKLLKDLGFSNLHQVPKVVKINVNRGLGEAAQNSKALEASLSEVSTITGQKALVTRAK

KAIAGFKIRQGMPIGCAVTLRGERMYAFLERLINLALPRIRDFRGVSPKSFDGRGNFTLGVKEQLIFPEI

SFDKIDAIRGMDITIVTSARTDEEGRALLKEMGMPFRSN

>gi|41017828|sp|Q7VDG7.1|RS4_PROMA RecName: Full=30S ribosomal protein S4

MSRYRGPRLRITRRLGDLPGLTRKAAKRSNPPGQHGNARRKRSEYAIRLEEKQKLRFNYGISERQLVRYV

KKARAMEGSTGTNLLKLLEGRLDNVCFRLGFGPTIPGSRQLVNHGHVTVNGKTLDIASYQCKSGDTIAIR

ERKGSKKLAEGNLEFPGLANVPPHLELEKSKMTAKVTGKCDREWVAIEINELLVVEYYSRKV

>gi|37154400|sp|O07828.2|RS9_PROMA RecName: Full=30S ribosomal protein S9

MNSSSKNAVVYWGTGRRKTSVARVRLIPGTGKITINGRPGDHYLNFNPAYLSAVKAPLHTLGLGEAYDVL

VNVYGGGLTGQSDAIKQGAARALCELSVDNRKPLKTEGHLSRDPRAKERRKYGLKKARKAPQFSKR

>gi|71649172|sp|Q7VB02.1|RL34_PROMA RecName: Full=50S ribosomal protein L34

MTKRTFGGTSRKRKRVSGFRVRMRTHTGRSVIRSRRKKGRSRIAV

>gi|67461503|sp|Q7VC09.2|RL28_PROMA RecName: Full=50S ribosomal protein L28

MSRVCELSGTRANNGMAVSHSHIRTKKLQQANLQKRRLWWEEGKKWLNIRVSTSTLKTIQKKGLDSYAKS

QGIDLKKL

>gi|59798852|sp|Q7VA27.1|RS20_PROMA RecName: Full=30S ribosomal protein S20

MANNNSAKKRIQIAERNRIQNRTYKSAMRTLMKRCFEACGAYSEKPSEDAKKDIQNSMNDAFSKIDKAVK

TGVLHRNTGANQKSRLTSVVKKTIEPVVK

>gi|59798850|sp|Q7V9Y2.1|RL36_PROMA RecName: Full=50S ribosomal protein L36

MKVRASVKKMCEKCRVIRRHGRVMVICTATQKHKQRQG

>gi|54036291|sp|Q7V9L2.1|RL35_PROMA RecName: Full=50S ribosomal protein L35

MPKLKTRKAAAKRFKVTGTGKFMRRRAFRNHLLDHKSSKLKRHLGTKAVVDERDSENVSLMLPYS

>gi|51316783|sp|Q7VBW1.1|RL32_PROMA RecName: Full=50S ribosomal protein L32

MAVPKKKTSKGKRNQRHAVWKAKAGDAAQKALSLGKSILSGRAQGFVYPIQEEDSEE

>gi|81712820|sp|Q7VCZ9.1|RS21_PROMA RecName: Full=30S ribosomal protein S21

MTQVIVGENEGVESALRRFKREVSKAGIFNDLKRIRHHETPVEKYKRKQRLKNRTKRRR

>gi|81835790|sp|Q7V9X1.1|RS17_PROMA RecName: Full=30S ribosomal protein S17

MALKERLGTVVSDKMDKTVVVAVVNRFPHSIYQKTVSRTTRYKAHDEENNCKVGDRVRITETRPLSATKR

WSVAEVLRTTKQEKEAVK

>gi|81835789|sp|Q7V9W4.1|RL23_PROMA RecName: Full=50S ribosomal protein L23

MTEMFKERLADVIRRPLITEKATKGLDINQYTFEVDHRAAKPDIKAAVEKLFDVKVIGISTMNPPRRSRR

VGRFSGKRAQVKKAIVRLAEGNSIQLFPDSES

>gi|81712807|sp|Q7VBX5.1|RL33_PROMA RecName: Full=50S ribosomal protein L33

MAKKGTRIVVTLECTECRSAPASEKRSPGVSRYTTEKNRRNTSDRLELKKFCPQLNKMTIHKEIK

>gi|81712787|sp|Q7VAN3.1|RL21_PROMA RecName: Full=50S ribosomal protein L21

MSFDFMTPNKTKNSTVGNYAIVETSGTQFWLEADRYYDIDRINANVDETVTLDKVLLINDQKGFAIGKPY

IKGASVQLKVMAHKRGPKIIVYKMRPKKKTRTKNGHRQELTRVMVTSISNGEKPKKATTSAKPNTKKPST

AVKSSKVEKTPE

>gi|73621815|sp|Q7V9Y9.1|RL31_PROMA RecName: Full=50S ribosomal protein L31

MPKKDIHPNWYPDAKVICNGEVVMTTGSTQPEIHVDVWSGNHPFFTGTQKILDTEGRVDRFMRKYGMANP

DEDSTKNTKSSKKETSEDSSSKGS

>gi|67461182|sp|Q7V9X5.1|RS8_PROMA RecName: Full=30S ribosomal protein S8

MANHDPISDMLTRIRNASQKRHENTRIPASRMSRSIAKVLQNEGFISQVSEEGEGVKTQLVLELKYSGKH

RHPTIRSMKRVSKPGLRIYKNNRGLPKVLGGLGVAIISTSKGVMSDRDARKQGVGGEVLCYVY

>gi|62287313|sp|Q7VA03.1|RS7_PROMA RecName: Full=30S ribosomal protein S7

MSRRNAAEKRPVLPDPQFNNRLATMMVARLMKHGKKSTAQKILSQAFGLINERTGGDPIELFETAIKNAT

PLVEVRARRVGGATYQVPMEVRQERGTAMALRWLVNFSRSRNGRSMAHKLAGELMDAANEAGNAVRKREE

THKMAEANKAFAHYRY

>gi|61216288|sp|Q7V9F9.1|RS6_PROMA RecName: Full=30S ribosomal protein S6

MSDPTYYETMYILRPDIPEDEVEGHLKKYSEVLEKAKAKIIDNQMRGKRRLAYTIGKHKEGIYVQLSHTG

NGKHVETLERSMRLSEDVIRYLTVKQYGPLPTKRNTKSQDKEASTTNNENDTKEVKEAKDTKEVKEAKDT

KEVKEAKDTKEVKEAKDTKEVKEAKDTKEVKEAKDTKEVKEAKDTKEVKEAKDTKEVKEAKDTKEVKEEG

>gi|61215706|sp|Q7VCB6.1|RS2_PROMA RecName: Full=30S ribosomal protein S2

MAVVTLSEMMEAGAHFGHQTRRWNPKMSRYIYCARNGVHIIDLVKTAVCMNNAYKWTRNAAKSGKRFLFV

GTKKQASEVVAQEAARCGASYVNQRWLGGMLTNWTTMKARIDRLKDLERMESSGAIAMRPKKEASVLRHE

LERLQKYLGGLKGMKRLPDVVVLVDQRRETNAVLEARKLDIPLVSMLDTNCDPDLCEVPIPCNDDAVRSV

QLVLGRIADAINEGRHGSNDQRARQKYS

>gi|59798853|sp|Q7VBX4.1|RS18_PROMA RecName: Full=30S ribosomal protein S18

MPNSLFKKKLSPIKPGDPIDYKDVETLKKFITERGKILPRRLTGLTAKQQRDLTTAVKRARIIALLPFVN

PEG

>gi|54036407|sp|Q7V9W6.1|RS19_PROMA RecName: Full=30S ribosomal protein S19

MGRSLKKGPFIADSLIKKVEKQNSDDDKSVIKTWSRASTILPMMIGHTIAVHNGKTHVPVFITEQMVGHK

LGEFAPTRTYRGHMKDKKGGR

>gi|46577200|sp|Q7V9W3.1|RL4_PROMA RecName: Full=50S ribosomal protein L4

MANCAVLDWQGKEAGKASLNLKVAKDSSAVDLMHRAVLRQQAHSRQGTASTLTRAEVRGGGRKPYKQKGT

GRARQGSIRTPLRPGGGIIFGPKPRQYNLSMNRKERRLALRTALMARFNDVIAVKDFGSKLKVPKTKEIQ

DFLARLDISSNSKVLIILSQPSDIIRRSVRNLEKVKLIAAEHLNVFDLLNANSLIIGEDALGKIKEVYGD

D

>gi|46396797|sp|Q7V9X3.1|RL24_PROMA RecName: Full=50S ribosomal protein L24

MFNLNQTKKASLPIKMRIRKGDTVQVINGKEKGKTGEVLKTLPIENRVIVQGINLRTRHVKPTQEGESGR

IVTEEASLHASNVMVYSTKQKTASRVEVFIDKDGSKKRRLKKTGELID

>gi|42559600|sp|Q7V9W2.1|RL3_PROMA RecName: Full=50S ribosomal protein L3

MSIGILGKKLGMSQFFDEKGRSIPVTLVEAGPCRITQLKSSATDGYSAVQIAFGAVREKLINKPFQGHLA

KSGDDLLRYMSEYRVKDVGEFQLGAQITVGDFEEGQKVDVSGTSMGRGFSGYQKRHGFSRGPMSHGSKNH

REPGSTGAGTTPGRIYPGKRMAGRYGGKKITTRGLMIMKVDSDKNLLVIKGSLPGKPGSLLNIRPAKCVG

LNSSKGGNK

>gi|42559205|sp|Q7V9W5.1|RL2_PROMA RecName: Full=50S ribosomal protein L2

MAIRTFRPYTPGTRTRVVTDFNEVTGRKPERSLVIAKHRLKGRNNRGVITCRHRGGGHKRQYRLVDFRRN

KHGVPAKVAAIHYDPHRNARLALLFYKDGEKRYILAPAGISIGQEVLSGKDVPIEIGNALPLSSIPLGSS

VHCVELYPGRGGQMVRCAGSSAQLMAKEGEYVALKLPSTEVRLVRGECYATLGEVGNSEIRNTSLGKAGR

RRWLGRRPQVRGSVMNPCDHPHGGGEGRAPVGRAGPVTPWGKPALGLKTRKRNKPSNRFVLRKRRRVSKR

SRGGRDS

>gi|39932303|sp|Q7VAN2.1|RL27_PROMA RecName: Full=50S ribosomal protein L27

MAHKKGTGSTRNGRDSNSKRLGVKAYGGEKVTAGSILIRQRGTSVLPGANVGQGKDDTLFALVDGIVNFE

TIKRSLKKRKRISVSLA

>gi|39931795|sp|Q7VDB0.1|RL19_PROMA RecName: Full=50S ribosomal protein L19

MTEDLKNTSPSKEESNEIEESSKATPKATRETKPKDSPSKTKLSAQALIAQFEKSQQKKKVPEVYVGDTV

RVGVRISEGNKERVQPYEGVIIAKRHGGLNQTITVRRIFQGVGVERVFMVHSPQVASIKVERRGKVRRAK

LFYLRDRVGKATRVKQRFDR

>gi|39931790|sp|Q7V9L1.1|RL20_PROMA RecName: Full=50S ribosomal protein L20

MSRVKRGNVARKRRNKILRLAKGFQGSNGSLFRTANQRVMKALCNAYRDRKRRKRDFRRLWIARINAAAR

MNGMSYSKLIGNLKKADIRINRKMLAQIAILDPTNFQKVVADVKK

>gi|81712817|sp|Q7VCI7.1|RS15_PROMA RecName: Full=30S ribosomal protein S15

MTLNTEAKQKIINKHQTHGTDTGSVEVQVAMLSERINQLSKHLQSNNHDFSSRQGLLKMIGQRKRLLNYV

KKQSESRYSSLVTKLGIRG

>gi|81712770|sp|Q7V9Y8.1|RL13_PROMA RecName: Full=50S ribosomal protein L13

MNKTITPSIDSINRQWYLVDAENQTLGRLATEVASVLRGKNKPSFTPHLDTGDFVIIVNAEKIKVTGKKG

MQKLYRRHSGRPGGMKVETFNSLQERIPERIVEKAIKGMLPHNALGRQLFRKLKVYKGSEHPHSAQNPQV

LSITTNELVK

>gi|81712769|sp|Q7V9Y6.1|RL17_PROMA RecName: Full=50S ribosomal protein L17

MRHQLRVPQLGRPADQRKALLRGLTTQLIREGRVTTTKTRAKALRDEVERMIGLAKDGSLAARRRAIGYI

YDKKLVHSLFEKAQERYGDRQGGYTRIVRTVPRRGDNAEMAIIELV

>gi|81712767|sp|Q7V9X9.1|RL15_PROMA RecName: Full=50S ribosomal protein L15

MTSISLDSLKPNKGARKRKTRKGRGIAAGQGASCGFGMRGQKSRSGRPTRPGFEGGQMPLYRRVPKLKHF

PLVNSKSFTLINVASLNSLKEGSTVNLDSLVKTGIVTSPKYPLKVLGNGNLKVKLVVQAAAFTASAKTKI

EGAGGSCEVFE

>gi|81712765|sp|Q7V9X2.1|RL14_PROMA RecName: Full=50S ribosomal protein L14

MIQQETYLTVADNSGAKRLQCIRVLGSNRRYAHVGDVIVAAVKDAVPNMGVKKSDVVKAVVVRTKATLRR

ETGNSIRFDDNAAVLINEDKNPRGTRVFGPVARELRERNYTKIVSLAPEVI

>gi|81664239|sp|Q7VAZ6.1|RS14_PROMA RecName: Full=30S ribosomal protein S14

MAKKSMIARDVKRKKLVERYASKRKKLLDQFHSAKDPMERLEIHRKIQALPRNSAPSRMRNRCWATGKPR

GVYRDFGLCRNQLRERAHKGELPGVVKSS

>gi|81663961|sp|Q7V9Y3.1|RS13_PROMA RecName: Full=30S ribosomal protein S13

MARIAGIDLPRDKRVEIALTYIYGIGLTRAQNILAKTGVNPDIRVKDLEDGDVQKLRAATESFTIEGDLR

RQEGMALKRLQDIGCLRGRRHRMSLPVRGQRTRTNARTRRGSRKTVAGRKK

>gi|73621670|sp|Q7V9X7.1|RL18_PROMA RecName: Full=50S ribosomal protein L18

MSTLSRKQKTQKRHKRLRRNLSGTDQRPRLAVFRSNNHIYAQVIDDVAQNTLCAASTLEKEFLSNSKVNA

STCAASTAVGEMLAKRALGKGIKQVVFDRGGSLYHGRVKALAEAARQAGLTF

>gi|59798851|sp|Q7V9Y4.1|RS11_PROMA RecName: Full=30S ribosomal protein S11

MATPAKKTGSKKSKRNVPNGVVHIQSTFNNTIVSITDTNGEVISWSSAGASGFKGARKGTPFAAQTAAEA

AARRALDQGMRQIEVLVRGPGSGRETAIRALQVAGLEITLIRDVTPLPHNGCRRAKRRRV

>gi|52783393|sp|Q7VA02.1|RS12_PROMA RecName: Full=30S ribosomal protein S12

MPTIQQLIRTERQRLTRKTKSPALRSCPERRGVCTRVYTSTPKKPNSALRKVARVRLTSGFEVTAYIPGI

GHNLQEHSVVLLRGGRVKDLPGVRYHIIRGTLDTAGVKDRRQARSKYGAKAPKS

>gi|44888418|sp|Q7VA06.1|RS10_PROMA RecName: Full=30S ribosomal protein S10

MSTAIAQQKIRIRLKAFDRRMLDLSCDKIIETADNTAATAIGPIPLPTKRKIYCVLRSPHVDKDSREHFE

TRTHRRIIDIYSPSAKTIDALMKLDLPSGVDIEVKL

>gi|73917119|sp|Q7V9X0.1|RL29_PROMA RecName: Full=50S ribosomal protein L29

MAESEKLDVSKLTDIEIKEKIDVTRRELFDLRFQRATRQLNETHRFKKARVQLAQLLTAQGERSRSNT

>gi|81663960|sp|Q7V9W9.1|RL16_PROMA RecName: Full=50S ribosomal protein L16

MLSPKRTKFRKQQRGRMRGVATRGNKIAFGQFALQAQECGWITSRQIEASRRAMTRYVKRGGQIWIRIFP

DKPVTMRPAETRMGSGKGNPEFWVAVVKPGRILFEMGGEEITEEIAKEAMRLAQYKLPIKTKFLALAEGE

KPTQVGKAPPKSSFLPSDETETAAAQAGTEASSASSVTPLES

>gi|46577392|sp|Q7VA74.1|RRP3_PROMA RecName: Full=Probable 30S ribosomal protein PSRP-3; AltName: Full=Ycf65-like protein

MLGAAEVLASATFDADGVPSGHTPKADEGRFLLKILWLPDNVALAVDQIVGGGTSPLTAYFFWPREDAWE

TLKTELEDKAWITDNERVEVLNKATEVINYWQEEGKGKTLEEAKLKFPEVTFCGTA

>gi|33241096|ref|NP_876038.1| Transcription elongation factor NusA [Prochlorococcus marinus subsp. marinus str. CCMP1375]

MALVLLPGLNNLIEDISEEKKLPAQVVETALREALLKGYERYRKTLYLGINENPFEEEYFSNFDIGLDLD

QEGYRVLASKIIVEEVDSEDHQIALAEVMQVAEDAQAGDTVVLDVTPEKEEFGRMAASTTKQVLSQKLRD

QQRRMIQEEFADLEDPVLTARVIRFERQSVIMAVSSGLGRPEVEAELPRRDQLPNDNYRANATFKVFLKE

VSEVPRRGPQLFVSRANAGLVVYLFENEVPEIQEGSVRIVAVAREANPPSRSVGPRTKVAVDSIEREVDP

VGACIGARGSRIQQVVNELRGEKIDVIRWSTDPIQYICNSLSPARVENVRLVDPDGQHAHVLVPPDQLSL

AIGREGQNVRLAARLTGWKIDIKNSQEYDQSAEDAIVAELITHREEEESLQKEAEDRLAAEQAARAEEDA

RLRELYPLPEDEEEESENIENDTSSLEMNYNDEESKDSYIPEESNSNEDRER

>gi|33240756|ref|NP_875698.1| Ribonuclease PH [Prochlorococcus marinus subsp. marinus str. CCMP1375]

MQGQTTSVSFDGREIRLTTGRYAPQAGGSVLIECGDTAVLVTATQGQGREGADFLPLSCDYEERLYAAGR

IPGSFMRREGRPPERATLISRLIDRPLRPLFPNWMRDDIQVVATCLSLDERVPADILAVTGSSMATLLAG

IPFYGPMAAVRVGLLGDDFVLNPSFREIERGDLDLVVAGTPDGVVMVEAGSNQLTEQDVIEAIDFGYEAV

NELIKAQESILKDSGLTQIKPEKPDLDETVPSYLEKNCTKPISALLKEFDLSKEDRDLKLDEIKTNCAEK

IDSLKDDNAVKKSITTNTKLLGISFKALTKKLMREQIIKDGKRVDGRALDEVREISAEAGILPKRVHGSG

LFQRGLTQVLSTATLGTPSDAQEMDDLNPSPDKTYIHHYNFPPYSVGETRPMRTPGRREVGHGALAERAI

IPVLPPKESFPYVLRVVSEVLSSNGSTSMGSVCGSTIALLDAGVPLKAPVSGAAMGLIKEGEEVRILTDI

QGIEDFLGDMDFKVAGTEKGITALQMDMKMTGLPIKIIGEAINQAKPARTHILEKMVQAIDKPRETLSPH

APRLLSFRIDPELIGTVIGPGGRTIKGITERTNTKIDIEDGGIVTIASHDGVAAEEAQKIIEGLTRKVHE

GEVFTGSITRIIPIGAFVEILPGKEGMIHISQLSEARVEKVEDVVKVGDEVTVRVREIDNRGRINLTLRG

IPQNGDMQYYPQPTPTPVAPLM

>gi|33240556|ref|NP_875498.1| Acetyltransferase, GNAT family [Prochlorococcus marinus subsp. marinus str. CCMP1375]

MQKLDLYLQTKVITIGIRDLKRCIDLDLKTLNGIWNNTQWEKELTDPNRICLGIVKGTDLLALATGWLIM

SDLNITFLAVDPLYQRLGLGIRVLSELINKAKKSGMKIATIDAKETNIAAKALYKKMDFEEVRCRYNLYK

DGNNAIIYQRLFY

>gi|33239471|ref|NP_874413.1| Predicted GTPase [Prochlorococcus marinus subsp. marinus str. CCMP1375]

MLEINKDRIKGIVLALKANYYIVQIDTINLIPELFKKKIGDHNFRLLCTKRSRLSYKGHSVSVGDFVLIE

AIDWTAETGVISFVEPRKNLITRPPVANVTDVIIVVSLLDPSFDLNQVSRFLMKAEETGLKVTIVLTKRD

LIDEKILEKYDKKLQTWGYQPIPISIVNGEGIQKLSARLKSMKLGVLCGPSGVGKSSLINYLLPKISIPI

GKLSKKLKRGRHTTRHVELFSIYSDSFIADTPGFNKPEFYTEPSQVPQLFPELRSQLLIKKCKFRNCMHL

NEPDCAISRDWERYSNYKNFLQEMLNYHH

>gi|33238625|gb|AAQ00691.1| Transcription elongation factor NusA [Prochlorococcus marinus subsp. marinus str. CCMP1375]

MALVLLPGLNNLIEDISEEKKLPAQVVETALREALLKGYERYRKTLYLGINENPFEEEYFSNFDIGLDLD

QEGYRVLASKIIVEEVDSEDHQIALAEVMQVAEDAQAGDTVVLDVTPEKEEFGRMAASTTKQVLSQKLRD

QQRRMIQEEFADLEDPVLTARVIRFERQSVIMAVSSGLGRPEVEAELPRRDQLPNDNYRANATFKVFLKE

VSEVPRRGPQLFVSRANAGLVVYLFENEVPEIQEGSVRIVAVAREANPPSRSVGPRTKVAVDSIEREVDP

VGACIGARGSRIQQVVNELRGEKIDVIRWSTDPIQYICNSLSPARVENVRLVDPDGQHAHVLVPPDQLSL

AIGREGQNVRLAARLTGWKIDIKNSQEYDQSAEDAIVAELITHREEEESLQKEAEDRLAAEQAARAEEDA

RLRELYPLPEDEEEESENIENDTSSLEMNYNDEESKDSYIPEESNSNEDRER

>gi|33238284|gb|AAQ00351.1| Ribonuclease PH [Prochlorococcus marinus subsp. marinus str. CCMP1375]

MQGQTTSVSFDGREIRLTTGRYAPQAGGSVLIECGDTAVLVTATQGQGREGADFLPLSCDYEERLYAAGR

IPGSFMRREGRPPERATLISRLIDRPLRPLFPNWMRDDIQVVATCLSLDERVPADILAVTGSSMATLLAG

IPFYGPMAAVRVGLLGDDFVLNPSFREIERGDLDLVVAGTPDGVVMVEAGSNQLTEQDVIEAIDFGYEAV

NELIKAQESILKDSGLTQIKPEKPDLDETVPSYLEKNCTKPISALLKEFDLSKEDRDLKLDEIKTNCAEK

IDSLKDDNAVKKSITTNTKLLGISFKALTKKLMREQIIKDGKRVDGRALDEVREISAEAGILPKRVHGSG

LFQRGLTQVLSTATLGTPSDAQEMDDLNPSPDKTYIHHYNFPPYSVGETRPMRTPGRREVGHGALAERAI

IPVLPPKESFPYVLRVVSEVLSSNGSTSMGSVCGSTIALLDAGVPLKAPVSGAAMGLIKEGEEVRILTDI

QGIEDFLGDMDFKVAGTEKGITALQMDMKMTGLPIKIIGEAINQAKPARTHILEKMVQAIDKPRETLSPH

APRLLSFRIDPELIGTVIGPGGRTIKGITERTNTKIDIEDGGIVTIASHDGVAAEEAQKIIEGLTRKVHE

GEVFTGSITRIIPIGAFVEILPGKEGMIHISQLSEARVEKVEDVVKVGDEVTVRVREIDNRGRINLTLRG

IPQNGDMQYYPQPTPTPVAPLM

>gi|33238084|gb|AAQ00151.1| Acetyltransferase, GNAT family [Prochlorococcus marinus subsp. marinus str. CCMP1375]

MQKLDLYLQTKVITIGIRDLKRCIDLDLKTLNGIWNNTQWEKELTDPNRICLGIVKGTDLLALATGWLIM

SDLNITFLAVDPLYQRLGLGIRVLSELINKAKKSGMKIATIDAKETNIAAKALYKKMDFEEVRCRYNLYK

DGNNAIIYQRLFY

>gi|33236996|gb|AAP99065.1| Predicted GTPase [Prochlorococcus marinus subsp. marinus str. CCMP1375]

MLEINKDRIKGIVLALKANYYIVQIDTINLIPELFKKKIGDHNFRLLCTKRSRLSYKGHSVSVGDFVLIE

AIDWTAETGVISFVEPRKNLITRPPVANVTDVIIVVSLLDPSFDLNQVSRFLMKAEETGLKVTIVLTKRD

LIDEKILEKYDKKLQTWGYQPIPISIVNGEGIQKLSARLKSMKLGVLCGPSGVGKSSLINYLLPKISIPI

GKLSKKLKRGRHTTRHVELFSIYSDSFIADTPGFNKPEFYTEPSQVPQLFPELRSQLLIKKCKFRNCMHL

NEPDCAISRDWERYSNYKNFLQEMLNYHH

>gi|33240015|ref|NP_874957.1| Acetyltransferase, GNAT family [Prochlorococcus marinus subsp. marinus str. CCMP1375]

MIKKIGERKISFSGWSNGRSGSNDFREMYGSDACECISSNDQYTFVFSQVRTLDLIELEQLLQSVGWSRR

PIRRVKKALDNSLLKVGVWQHDPKFPRLIGFARCTGDEVIQATIWDVAIHPVYQGFGLGKELMSYVLRSL

KDKGIERVVLFADPGVISFYQSQGWTLEPKGNRCAFWYAN

>gi|33239573|ref|NP_874515.1| 2-methylthioadenine synthetase [Prochlorococcus marinus subsp. marinus str. CCMP1375]

MHLGCEKNLVDTEHMMGLLDQGGYSISTNPSEASLVVVNTCSFIQDAREESVRVLVGLAEQDKEIIIAGC

LAQHFQEELLQSIPEAKAIIGTGDYQNILNVLQRIEQGEIVNQVSNNPTFVGDEKLPRFRTTGKAVAYLK

IAEGCDYSCAFCIIPKLRGMQRSRSIESIVAEANQLAKQGVKELILISQITTNYGLDLYGRPCLADLLRE

LGDVEIPWIRVHYAYPTGLTSEVIKAFREVPNLLPYLDLPLQHSHPDVLRLMNRPWQLDLNASLLDRIRS

ELPDAIFRTSLIVGFPGETEEHFNHLVSFVQTQQFDHIGVFTFSSEAGTKAASLANQIPFSVAEARKDKI

ISIQQPIAELKNQNWIGRTVDVLIEREDKDSAEFVGRCARFSPEVDGFVRLQINNTFTNQLNIGMMTPAL

ITGADLYDLTGQVV

>gi|33237541|gb|AAP99609.1| Acetyltransferase, GNAT family [Prochlorococcus marinus subsp. marinus str. CCMP1375]

MIKKIGERKISFSGWSNGRSGSNDFREMYGSDACECISSNDQYTFVFSQVRTLDLIELEQLLQSVGWSRR

PIRRVKKALDNSLLKVGVWQHDPKFPRLIGFARCTGDEVIQATIWDVAIHPVYQGFGLGKELMSYVLRSL

KDKGIERVVLFADPGVISFYQSQGWTLEPKGNRCAFWYAN

>gi|33237098|gb|AAP99167.1| 2-methylthioadenine synthetase [Prochlorococcus marinus subsp. marinus str. CCMP1375]

MHLGCEKNLVDTEHMMGLLDQGGYSISTNPSEASLVVVNTCSFIQDAREESVRVLVGLAEQDKEIIIAGC

LAQHFQEELLQSIPEAKAIIGTGDYQNILNVLQRIEQGEIVNQVSNNPTFVGDEKLPRFRTTGKAVAYLK

IAEGCDYSCAFCIIPKLRGMQRSRSIESIVAEANQLAKQGVKELILISQITTNYGLDLYGRPCLADLLRE

LGDVEIPWIRVHYAYPTGLTSEVIKAFREVPNLLPYLDLPLQHSHPDVLRLMNRPWQLDLNASLLDRIRS

ELPDAIFRTSLIVGFPGETEEHFNHLVSFVQTQQFDHIGVFTFSSEAGTKAASLANQIPFSVAEARKDKI

ISIQQPIAELKNQNWIGRTVDVLIEREDKDSAEFVGRCARFSPEVDGFVRLQINNTFTNQLNIGMMTPAL

ITGADLYDLTGQVV

>gi|311812478|emb|CBX83577.1| unnamed protein product [Prochlorococcus marinus subsp. marinus str. CCMP1375]

MQGQTTSVSFDGREIRLTTGRYAPQAGGSVLIECGDTAVLVTATQGQGREGADFLPLSCDYEERLYAAGR

IPGSFMRREGRPPERATLISRLIDRPLRPLFPNWMRDDIQVVATCLSLDERVPADILAVTGSSMATLLAG

IPFYGPMAAVRVGLLGDDFVLNPSFREIERGDLDLVVAGTPDGVVMVEAGSNQLTEQDVIEAIDFGYEAV

NELIKAQESILKDSGLTQIKPEKPDLDETVPSYLEKNCTKPISALLKEFDLSKEDRDLKLDEIKTNCAEK

IDSLKDDNAVKKSITTNTKLLGISFKALTKKLMREQIIKDGKRVDGRALDEVREISAEAGILPKRVHGSG

LFQRGLTQVLSTATLGTPSDAQEMDDLNPSPDKTYIHHYNFPPYSVGETRPMRTPGRREVGHGALAERAI

IPVLPPKESFPYVLRVVSEVLSSNGSTSMGSVCGSTIALLDAGVPLKAPVSGAAMGLIKEGEEVRILTDI

QGIEDFLGDMDFKVAGTEKGITALQMDMKMTGLPIKIIGEAINQAKPARTHILEKMVQAIDKPRETLSPH

APRLLSFRIDPELIGTVIGPGGRTIKGITERTNTKIDIEDGGIVTIASHDGVAAEEAQKIIEGLTRKVHE

GEVFTGSITRIIPIGAFVEILPGKEGMIHISQLSEARVEKVEDVVKVGDEVTVRVREIDNRGRINLTLRG

IPQNGDMQYYPQPTPTPVAPLM

>gi|300582217|emb|CBV22372.1| unnamed protein product [Prochlorococcus marinus subsp. marinus str. CCMP1375]

MALVLLPGLNNLIEDISEEKKLPAQVVETALREALLKGYERYRKTLYLGINENPFEEEYFSNFDIGLDLD

QEGYRVLASKIIVEEVDSEDHQIALAEVMQVAEDAQAGDTVVLDVTPEKEEFGRMAASTTKQVLSQKLRD

QQRRMIQEEFADLEDPVLTARVIRFERQSVIMAVSSGLGRPEVEAELPRRDQLPNDNYRANATFKVFLKE

VSEVPRRGPQLFVSRANAGLVVYLFENEVPEIQEGSVRIVAVAREANPPSRSVGPRTKVAVDSIEREVDP

VGACIGARGSRIQQVVNELRGEKIDVIRWSTDPIQYICNSLSPARVENVRLVDPDGQHAHVLVPPDQLSL

AIGREGQNVRLAARLTGWKIDIKNSQEYDQSAEDAIVAELITHREEEESLQKEAEDRLAAEQAARAEEDA

RLRELYPLPEDEEEESENIENDTSSLEMNYNDEESKDSYIPEESNSNEDRER

>gi|300575629|emb|CBV26316.1| unnamed protein product [Prochlorococcus marinus subsp. marinus str. CCMP1375]

MALVLLPGLNNLIEDISEEKKLPAQVVETALREALLKGYERYRKTLYLGINENPFEEEYFSNFDIGLDLD

QEGYRVLASKIIVEEVDSEDHQIALAEVMQVAEDAQAGDTVVLDVTPEKEEFGRMAASTTKQVLSQKLRD

QQRRMIQEEFADLEDPVLTARVIRFERQSVIMAVSSGLGRPEVEAELPRRDQLPNDNYRANATFKVFLKE

VSEVPRRGPQLFVSRANAGLVVYLFENEVPEIQEGSVRIVAVAREANPPSRSVGPRTKVAVDSIEREVDP

VGACIGARGSRIQQVVNELRGEKIDVIRWSTDPIQYICNSLSPARVENVRLVDPDGQHAHVLVPPDQLSL

AIGREGQNVRLAARLTGWKIDIKNSQEYDQSAEDAIVAELITHREEEESLQKEAEDRLAAEQAARAEEDA

RLRELYPLPEDEEEESENIENDTSSLEMNYNDEESKDSYIPEESNSNEDRER

>gi|300565513|emb|CBV14281.1| unnamed protein product [Prochlorococcus marinus subsp. marinus str. CCMP1375]

MALVLLPGLNNLIEDISEEKKLPAQVVETALREALLKGYERYRKTLYLGINENPFEEEYFSNFDIGLDLD

QEGYRVLASKIIVEEVDSEDHQIALAEVMQVAEDAQAGDTVVLDVTPEKEEFGRMAASTTKQVLSQKLRD

QQRRMIQEEFADLEDPVLTARVIRFERQSVIMAVSSGLGRPEVEAELPRRDQLPNDNYRANATFKVFLKE

VSEVPRRGPQLFVSRANAGLVVYLFENEVPEIQEGSVRIVAVAREANPPSRSVGPRTKVAVDSIEREVDP

VGACIGARGSRIQQVVNELRGEKIDVIRWSTDPIQYICNSLSPARVENVRLVDPDGQHAHVLVPPDQLSL

AIGREGQNVRLAARLTGWKIDIKNSQEYDQSAEDAIVAELITHREEEESLQKEAEDRLAAEQAARAEEDA

RLRELYPLPEDEEEESENIENDTSSLEMNYNDEESKDSYIPEESNSNEDRER

>gi|259705029|emb|CBG08850.1| unnamed protein product [Prochlorococcus marinus subsp. marinus str. CCMP1375]

MALVLLPGLNNLIEDISEEKKLPAQVVETALREALLKGYERYRKTLYLGINENPFEEEYFSNFDIGLDLD

QEGYRVLASKIIVEEVDSEDHQIALAEVMQVAEDAQAGDTVVLDVTPEKEEFGRMAASTTKQVLSQKLRD

QQRRMIQEEFADLEDPVLTARVIRFERQSVIMAVSSGLGRPEVEAELPRRDQLPNDNYRANATFKVFLKE

VSEVPRRGPQLFVSRANAGLVVYLFENEVPEIQEGSVRIVAVAREANPPSRSVGPRTKVAVDSIEREVDP

VGACIGARGSRIQQVVNELRGEKIDVIRWSTDPIQYICNSLSPARVENVRLVDPDGQHAHVLVPPDQLSL

AIGREGQNVRLAARLTGWKIDIKNSQEYDQSAEDAIVAELITHREEEESLQKEAEDRLAAEQAARAEEDA

RLRELYPLPEDEEEESENIENDTSSLEMNYNDEESKDSYIPEESNSNEDRER

>gi|259700845|emb|CBG00761.1| unnamed protein product [Prochlorococcus marinus subsp. marinus str. CCMP1375]

MALVLLPGLNNLIEDISEEKKLPAQVVETALREALLKGYERYRKTLYLGINENPFEEEYFSNFDIGLDLD

QEGYRVLASKIIVEEVDSEDHQIALAEVMQVAEDAQAGDTVVLDVTPEKEEFGRMAASTTKQVLSQKLRD

QQRRMIQEEFADLEDPVLTARVIRFERQSVIMAVSSGLGRPEVEAELPRRDQLPNDNYRANATFKVFLKE

VSEVPRRGPQLFVSRANAGLVVYLFENEVPEIQEGSVRIVAVAREANPPSRSVGPRTKVAVDSIEREVDP

VGACIGARGSRIQQVVNELRGEKIDVIRWSTDPIQYICNSLSPARVENVRLVDPDGQHAHVLVPPDQLSL

AIGREGQNVRLAARLTGWKIDIKNSQEYDQSAEDAIVAELITHREEEESLQKEAEDRLAAEQAARAEEDA

RLRELYPLPEDEEEESENIENDTSSLEMNYNDEESKDSYIPEESNSNEDRER

>gi|259679365|emb|CBG12802.1| unnamed protein product [Prochlorococcus marinus subsp. marinus str. CCMP1375]

MALVLLPGLNNLIEDISEEKKLPAQVVETALREALLKGYERYRKTLYLGINENPFEEEYFSNFDIGLDLD

QEGYRVLASKIIVEEVDSEDHQIALAEVMQVAEDAQAGDTVVLDVTPEKEEFGRMAASTTKQVLSQKLRD

QQRRMIQEEFADLEDPVLTARVIRFERQSVIMAVSSGLGRPEVEAELPRRDQLPNDNYRANATFKVFLKE

VSEVPRRGPQLFVSRANAGLVVYLFENEVPEIQEGSVRIVAVAREANPPSRSVGPRTKVAVDSIEREVDP

VGACIGARGSRIQQVVNELRGEKIDVIRWSTDPIQYICNSLSPARVENVRLVDPDGQHAHVLVPPDQLSL

AIGREGQNVRLAARLTGWKIDIKNSQEYDQSAEDAIVAELITHREEEESLQKEAEDRLAAEQAARAEEDA

RLRELYPLPEDEEEESENIENDTSSLEMNYNDEESKDSYIPEESNSNEDRER

>gi|207007195|emb|CAR80119.1| unnamed protein product [Prochlorococcus marinus subsp. marinus str. CCMP1375]

MQGQTTSVSFDGREIRLTTGRYAPQAGGSVLIECGDTAVLVTATQGQGREGADFLPLSCDYEERLYAAGR

IPGSFMRREGRPPERATLISRLIDRPLRPLFPNWMRDDIQVVATCLSLDERVPADILAVTGSSMATLLAG

IPFYGPMAAVRVGLLGDDFVLNPSFREIERGDLDLVVAGTPDGVVMVEAGSNQLTEQDVIEAIDFGYEAV

NELIKAQESILKDSGLTQIKPEKPDLDETVPSYLEKNCTKPISALLKEFDLSKEDRDLKLDEIKTNCAEK

IDSLKDDNAVKKSITTNTKLLGISFKALTKKLMREQIIKDGKRVDGRALDEVREISAEAGILPKRVHGSG

LFQRGLTQVLSTATLGTPSDAQEMDDLNPSPDKTYIHHYNFPPYSVGETRPMRTPGRREVGHGALAERAI

IPVLPPKESFPYVLRVVSEVLSSNGSTSMGSVCGSTIALLDAGVPLKAPVSGAAMGLIKEGEEVRILTDI

QGIEDFLGDMDFKVAGTEKGITALQMDMKMTGLPIKIIGEAINQAKPARTHILEKMVQAIDKPRETLSPH

APRLLSFRIDPELIGTVIGPGGRTIKGITERTNTKIDIEDGGIVTIASHDGVAAEEAQKIIEGLTRKVHE

GEVFTGSITRIIPIGAFVEILPGKEGMIHISQLSEARVEKVEDVVKVGDEVTVRVREIDNRGRINLTLRG

IPQNGDMQYYPQPTPTPVAPLM

>gi|300567855|emb|CBV15641.1| unnamed protein product [Prochlorococcus marinus subsp. marinus str. CCMP1375]

MPKKDIHPNWYPDAKVICNGEVVMTTGSTQPEIHVDVWSGNHPFFTGTQKILDTEGRVDRFMRKYGMANP

DEDSTKNTKSSKKETSEDSSSKGS

>gi|259657487|emb|CBG02121.1| unnamed protein product [Prochlorococcus marinus subsp. marinus str. CCMP1375]

MPKKDIHPNWYPDAKVICNGEVVMTTGSTQPEIHVDVWSGNHPFFTGTQKILDTEGRVDRFMRKYGMANP

DEDSTKNTKSSKKETSEDSSSKGS

>gi|81665118|sp|Q7VE92.1|RIMO_PROMA RecName: Full=Ribosomal protein S12 methylthiotransferase RimO; Short=S12 MTTase; Short=S12 methylthiotransferase; AltName: Full=Ribosomal protein S12 (aspartate-C(3))-methylthiotransferase; AltName: Full=Ribosome maturation factor RimO

MHLGCEKNLVDTEHMMGLLDQGGYSISTNPSEASLVVVNTCSFIQDAREESVRVLVGLAEQDKEIIIAGC

LAQHFQEELLQSIPEAKAIIGTGDYQNILNVLQRIEQGEIVNQVSNNPTFVGDEKLPRFRTTGKAVAYLK

IAEGCDYSCAFCIIPKLRGMQRSRSIESIVAEANQLAKQGVKELILISQITTNYGLDLYGRPCLADLLRE

LGDVEIPWIRVHYAYPTGLTSEVIKAFREVPNLLPYLDLPLQHSHPDVLRLMNRPWQLDLNASLLDRIRS

ELPDAIFRTSLIVGFPGETEEHFNHLVSFVQTQQFDHIGVFTFSSEAGTKAASLANQIPFSVAEARKDKI

ISIQQPIAELKNQNWIGRTVDVLIEREDKDSAEFVGRCARFSPEVDGFVRLQINNTFTNQLNIGMMTPAL

ITGADLYDLTGQVV

>gi|6136541|sp|Q51893.1|YC52L_PROMA RecName: Full=Uncharacterized N-acetyltransferase ycf52-like

MIKKIGERKISFSGWSNGRSGSNDFREMYGSDACECISSNDQYTFVFSQVRTLDLIELEQLLQSVGWSRR

PIRRVKKALDNSLLKVGVWQHDPKFPRLIGFARCTGDEVIQATIWDVAIHPVYQGFGLGKELMSYVLRSL

KDKGIERVVLFADPGVISFYQSQGWTLEPKGNRCAFWYAN

>gi|38605176|sp|Q7VAM5.1|PRMA_PROMA RecName: Full=Ribosomal protein L11 methyltransferase; Short=L11 Mtase

MIQSTEFFWWKFELVFPPDVEESFLWFLNMAGIKSYAIERSPDNLQDQTLMVWLPSHEWLKKDREEFENS

LLALNKAFREDVLNTKWEKIIDEDWSSSWKKFWKADPVGSKILILPSWLELPDIYSNRIVIKLDPGSAFG

TGSHPTTRLCLEDLERNPPLGKKVVDIGCGSGVLGIAAIKLGAKEVRAIDIDSLAVRATSENIVLNNLSQ

KQLSVSLGSIENLANQLNPLSADLLICNTLSPVIKELAPYFFKLTHSYSRLCLSGLLVAQVEDITNFLSI

LGWELIDSYSSDNWALIRLCRNHP

>gi|48474803|sp|Q7V9R4.1|RIMM_PROMA RecName: Full=Ribosome maturation factor RimM

MCKKIIWLTIGKIVAPQGLSGKVRINPSSDFPERFIKSGDRWLQYDNEEPQKIQLNSGRQIPGKSIYVVE

FQGIDDREKAKALVGKKLLIDSSHRPTLAPGEFHLLDLLGLKVRLKNDHREIGEVTNLTSAGNDLLEVRL

LSGKKVLVPFVKEIVPEIKLQEGWLMVCPPPGLFDL

>gi|38257396|sp|Q7VEJ4.1|RSGA_PROMA RecName: Full=Putative ribosome biogenesis GTPase RsgA

MLEINKDRIKGIVLALKANYYIVQIDTINLIPELFKKKIGDHNFRLLCTKRSRLSYKGHSVSVGDFVLIE

AIDWTAETGVISFVEPRKNLITRPPVANVTDVIIVVSLLDPSFDLNQVSRFLMKAEETGLKVTIVLTKRD

LIDEKILEKYDKKLQTWGYQPIPISIVNGEGIQKLSARLKSMKLGVLCGPSGVGKSSLINYLLPKISIPI

GKLSKKLKRGRHTTRHVELFSIYSDSFIADTPGFNKPEFYTEPSQVPQLFPELRSQLLIKKCKFRNCMHL

NEPDCAISRDWERYSNYKNFLQEMLNYHH

>gi|81664238|sp|Q7VAZ5.1|PNP_PROMA RecName: Full=Polyribonucleotide nucleotidyltransferase; AltName: Full=Polynucleotide phosphorylase; Short=PNPase

MQGQTTSVSFDGREIRLTTGRYAPQAGGSVLIECGDTAVLVTATQGQGREGADFLPLSCDYEERLYAAGR

IPGSFMRREGRPPERATLISRLIDRPLRPLFPNWMRDDIQVVATCLSLDERVPADILAVTGSSMATLLAG

IPFYGPMAAVRVGLLGDDFVLNPSFREIERGDLDLVVAGTPDGVVMVEAGSNQLTEQDVIEAIDFGYEAV

NELIKAQESILKDSGLTQIKPEKPDLDETVPSYLEKNCTKPISALLKEFDLSKEDRDLKLDEIKTNCAEK

IDSLKDDNAVKKSITTNTKLLGISFKALTKKLMREQIIKDGKRVDGRALDEVREISAEAGILPKRVHGSG

LFQRGLTQVLSTATLGTPSDAQEMDDLNPSPDKTYIHHYNFPPYSVGETRPMRTPGRREVGHGALAERAI

IPVLPPKESFPYVLRVVSEVLSSNGSTSMGSVCGSTIALLDAGVPLKAPVSGAAMGLIKEGEEVRILTDI

QGIEDFLGDMDFKVAGTEKGITALQMDMKMTGLPIKIIGEAINQAKPARTHILEKMVQAIDKPRETLSPH

APRLLSFRIDPELIGTVIGPGGRTIKGITERTNTKIDIEDGGIVTIASHDGVAAEEAQKIIEGLTRKVHE

GEVFTGSITRIIPIGAFVEILPGKEGMIHISQLSEARVEKVEDVVKVGDEVTVRVREIDNRGRINLTLRG

IPQNGDMQYYPQPTPTPVAPLM
